# Synthesis and Receptor Binding Studies of α5 GABA_A_R Selective Novel Imidazodiazepines Targeted for Psychiatric and Cognitive Disorders

**DOI:** 10.3390/molecules28124771

**Published:** 2023-06-14

**Authors:** Dishary Sharmin, Md Yeunus Mian, Michael Marcotte, Thomas D. Prevot, Etienne Sibille, Jeffrey M. Witkin, James M. Cook

**Affiliations:** 1 Department of Chemistry and Biochemistry, Milwaukee Institute of Drug Discovery, University of Wisconsin Milwaukee, Milwaukee, WI 53201, USA; 2 Campbell Family Mental Health Research Institute of CAMH, Toronto, ON M5S 2S1, Canada; 3 Department of Psychiatry, University of Toronto, Toronto, ON M5T 1R8, Canada; 4 Department of Pharmacology and Toxicology, University of Toronto, Toronto, ON M5T 1R8, Canada; 5 Laboratory of Antiepileptic Drug Discovery, Ascension, St. Vincent, Indianapolis, IN 46260, USA

**Keywords:** γ-Aminobutyric acid (GABA), positive allosteric modulator, cognitive disorders, imidazodiazepine, PDSP, off-target profile

## Abstract

GABA mediates inhibitory actions through various GABA_A_ receptor subtypes, including 19 subunits in human GABAAR. Dysregulation of GABAergic neurotransmission is associated with several psychiatric disorders, including depression, anxiety, and schizophrenia. Selective targeting of α2/3 GABAARs can treat mood and anxiety, while α5 GABAA-Rs can treat anxiety, depression, and cognitive performance. GL-II-73 and MP-III-022, α5-positive allosteric modulators have shown promising results in animal models of chronic stress, aging, and cognitive disorders, including MDD, schizophrenia, autism, and Alzheimer’s disease. Described in this article is how small changes in the structure of imidazodiazepine substituents can greatly impact the subtype selectivity of benzodiazepine GABAAR. To investigate alternate and potentially more effective therapeutic compounds, modifications were made to the structure of imidazodiazepine **1** to synthesize different amide analogs. The novel ligands were screened at the NIMH PDSP against a panel of 47 receptors, ion channels, including hERG, and transporters to identify on- and off-target interactions. Any ligands with significant inhibition in primary binding were subjected to secondary binding assays to determine their K_i_ values. The newly synthesized imidazodiazepines were found to have variable affinities for the benzodiazepine site and negligible or no binding to any off-target profile receptors that could cause other physiological problems.

## 1. Introduction

γ-Aminobutyric acid (GABA) acts as an inhibitory neurotransmitter through various subtypes of GABA receptors [1,2,3]. Nineteen subunits of GABAAR have been identified in humans, including α1-6, β1-3, γ1-3, δ, ε, θ, π, and ρ1-3 subunits, although only a few subunit combinations form a functional receptor [4,5]. The GABAAR ion channel comprises α1-6β1-3γ2 subunits in a 2:2:1 stoichiometric ratio to form a fully functional benzodiazepine-mediated chloride ion channel [6]. Dysregulation of GABAergic neurotransmission has been linked to several psychiatric disorders, including schizophrenia, depression, autism, and anxiety [7,8,9,10,11]. Non-selective targeting of GABAAR with different subunits by benzodiazepines, commonly used to treat anxiety disorders, can result in side effects such as sedation, ataxia, [12] and dependence. Therefore, selective targeting of GABAAR, specifically by potentiating α2, α3, and α5 subunits containing GABAAR, can produce the desired therapeutic effects [7,12].

Evidence suggests that the brain’s γ-aminobutyric acid (GABA) system is dysregulated in major depressive disorder (MDD), resulting in altered inhibitory function and an impaired excitation/inhibition balance [13]. Reduced levels of GABA have been observed in the cerebrospinal fluid [14], plasma [15,16,17,18,19,20], and brain regions such as the occipital cortex [21,22,23,24,25], PFC [26], and anterior cingulate cortex (ACC) [21,26,27] of untreated depressed patients [24,25,28]. Currently, there are no available treatment options to correct this imbalance in MDD patients. Selective targeting of α2/3 GABAARs has been shown to be beneficial in treating mood and anxiety disorders [29], while α5 GABAA-Rs are thought to be useful in treating anxiety and cognitive deficits [29,30]. The α5-containing GABAAR subtype is primarily located in the hippocampus and constitutes approximately 5% of all GABAAR α-subtypes in the brain [31,32]. Within the hippocampus, α5-containing GABAARs are enriched and make up nearly 25% of all hippocampal GABAARs [33]. Moreover, there is a higher α5 expression in the ventral subregions compared to the dorsal subregions of the hippocampus, making it an ideal target for manipulation [34,35]. Reports have shown that positive modulation of GABAARs is anxiolytic. A knockdown of α5-GABAARs in healthy animals has been found to induce psychosis-like deficits in sensorimotor gating, while overexpression of the α5 subunit in the ventral hippocampus reversed the dopamine system dysfunction and behavioral deficits displayed in a rodent model of psychosis [36,37]. Selective ligands targeting the α5-GABAAR subtype have exhibited promising procognitive activity in various behavioral assays, including memory and spatial learning [38], as well as in models of cognitive disorders such as Alzheimer’s disease [39], Huntington’s disease [40], Down syndrome [41], schizophrenia [42,43,44,45], and depression [46].

**Figure 1:**
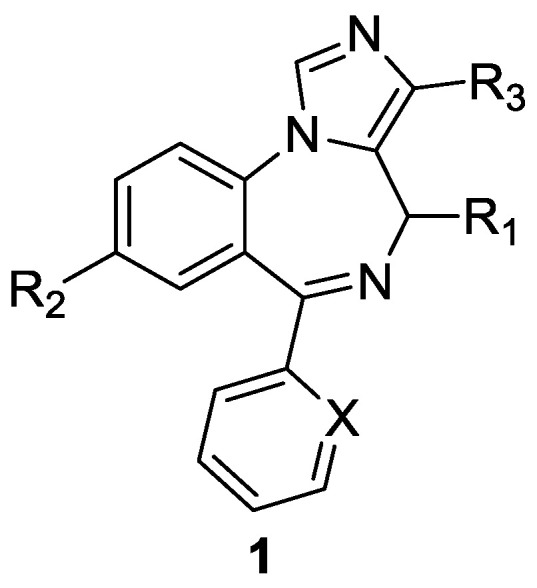
General structure of imidazodiazepines in this research.

The imidazodiazepine is a type of organic compound that features a fused-ring structure comprising two rings—an imidazole ring and a diazepine ring (Figure 1) [47]. The imidazole ring is a five-membered ring consisting of two nitrogen atoms and three carbon atoms, while the diazepine ring is a seven-membered ring that contains five carbon atoms and two nitrogen atoms. The two rings are fused together such that one carbon atom of the diazepine ring is shared with one nitrogen atom of the imidazole ring, forming a unique and versatile molecular scaffold. The biological activity of imidazodiazepines varies depending on the specific substitutions made to the fused-ring structure, allowing for the development of compounds with a range of therapeutic properties [48]. The α5 selective imidazodiazepines are efficacious in reversing symptoms in rodents in rodent models of psychiatric disorders [49]. One of the imidazodiazepines, GL-II-73, which is an α5-positive allosteric modulator (α5-PAM), was developed in Milwaukee and showed improved working memory in a mouse model of chronic stress and aging [49]. Further, another α5-PAM demonstrated improved cognitive functions in old rats compared to other α5-PAMs [50]. GL-II-73 reversed dendritic shrinkage and improved cognitive deficits in old mice [51]. This is a significant finding for MDD, schizophrenia, and Alzheimer’s disease. GL-II-73 also demonstrated pronounced neurotrophic effects in the distal segment of pyramidal neuronal dendrites, which is one of the locations of α5-GABA_A_ receptors [52]. Moreover, GL-II-73 increased spine counts and spine density in both the apical and the basal segment of pyramidal neuron dendrites of the CA1, suggesting its potential as a cognitive enhancer. GL-II-73 has the potential to become a treatment for patients suffering from cognitive decline due to GABAergic downregulation, as it exhibits anxiolytic and antidepressant effects [49]. In addition, the α5-GABAAR subtypeselective ligand MP-III-022, a monomethyl amide, is active in low doses in several psychiatric disorders including autism [53], antipsychotic activity [54], and cognitive disorders [55]. Due to the results obtained with GL-II-73 and MP-III-022 in animal models, several analogs were synthesized to extend the structure–activity relationship and screened for off-target effects in a 47-receptor panel assay.

## 2. Results and Discussion

### 2.1. Chemistry

#### 2.1.1. Amide Analogs of IMDZ 1

**Figure 2:**
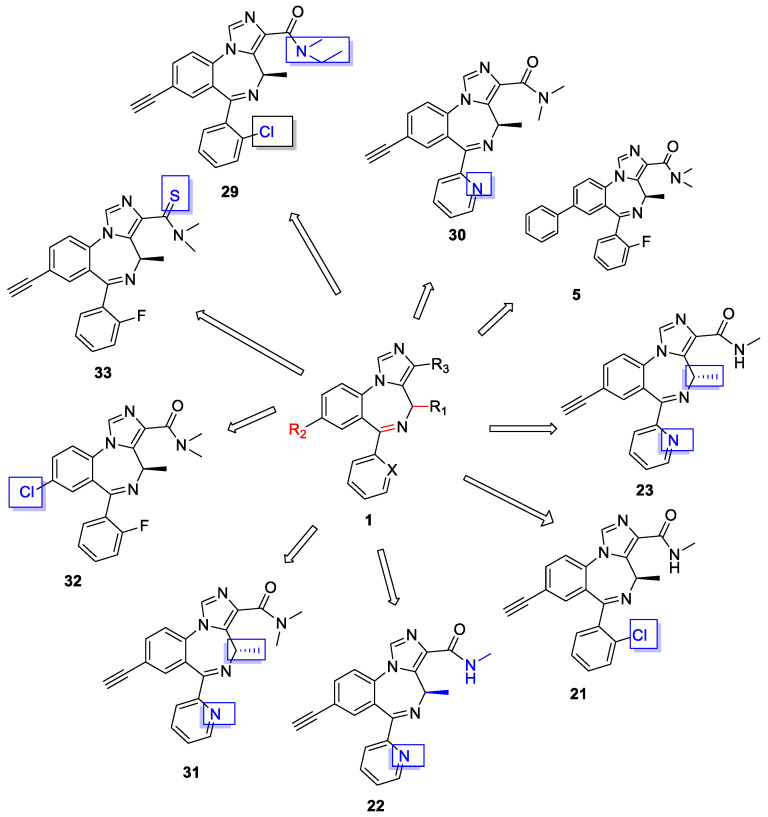
Amide analogs of IMDZ 1 synthesized by modifying different positions of ligand **1**, as illustrated.

Evidence suggested that subtle changes in the structure of imidazodiazepine (IMDZ) substituents can affect the subtype selectivity of BzR/GABA_A_R dramatically [56]. Several modifications were made to the core structure of imidazodiazepine **1** to synthesize different amide analogs in order to obtain alternate active ligands with distinct or improved antipsychotic or procognitive profiles (Figure 2). These novel analogs were characterized and tested in different animal models to evaluate their bioactivity or a lack thereof, as regards the SAR. A number of these newly synthesized analogs showed promising procognitive and anti-depressive activity in the Y-maze assay and forced swim test (unpublished).

Two positions of the imidazodiazepines (the C(8)-acetylene and the C(4) chiral methyl function) played an important role to enhance the α5-subtype selectivity [56,57], therefore a flat phenyl group was introduced at the C-8 position of IMDZ 1 in place of the ethinyl group. The phenyl group is commonly incorporated into different biologically active molecules in order to obtain improved pharmacological properties. The phenyl group may interact similarly to an ethinyl group within the lipophilic binding pocket of the receptor, leading to comparable physiological effects and improved subtype selectivity [58]. Furthermore, the flat phenyl group might slide into the binding pocket and undergo some weak pi-pi interactions for improved bioactivity. Consequently, the C(8)-substituted phenyl analog (**5**) of IMDZ 1, was synthesized based on this hypothesis.

**Scheme 1:**
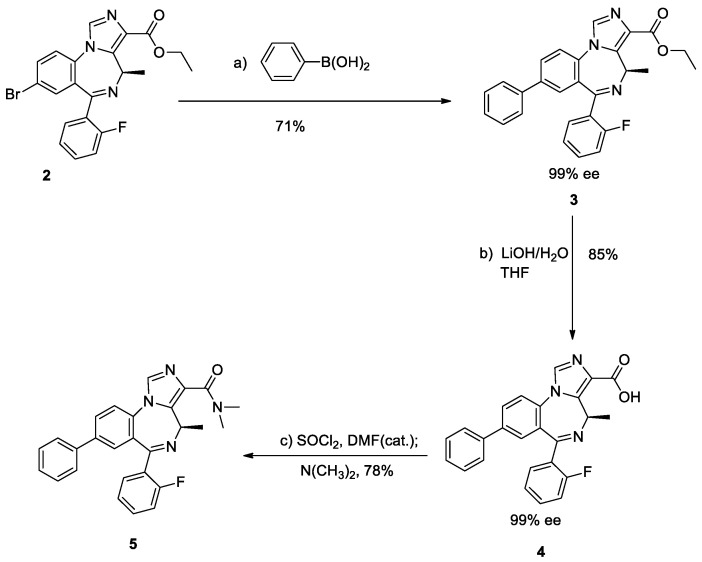
Synthesis of compound **5**. Reagents and conditions: (**a**) Pd(OAc)_2_ (5 mol%), tri-*o*-tolyl phosphine (10 mol%), toluene under argon, 15 min, rt; then K_3_PO_4_ (3 eq), H_2_O (0.2 eq), phenyl boronic acid (3 eq), 100 °C, 12 h (71%, >99% ee); (**b**) LiOH (4 eq), THF:H_2_O (4:1), 50 °C, 2 h; then CH_3_COOH (5.5 eq, pH ~4–5) (85%, >99%ee); (**c**) SOCl_2_ (10 eq), DMF (cat.), DCM, reflux (35–40 °C), 1 h; then *N*,*N*-dimethylamine (2 M solution in THF, 6 eq), DCM, 0 °C to rt, 2 h (78%, >99% ee).

The 8-bromo IMDZ **2** is the precursor for the synthesis of 8-phenyl amide analog (**5**) of IMDZ 1 (**1**). An improved method was developed for the synthesis of bromo imidazodiazepine [59]. The 8-phenyl compound **3** was synthesized from the 8-bromo imidazodiazepine (**2**) by Suzuki coupling [60]. The Suzuki coupling between the phenylboronic acid and the aryl bromide **2** in the presence of a palladium catalyst led to the formation of **3** (Scheme 1). Potassium phosphate, palladium acetate, and tri-o-tolyl phosphine were also used to carry out the coupling reaction. The reaction was completed within 12 h. The product was purified by silica gel flash chromatography (EtOAc:hexane = 1:1). The yield of the coupling reaction was 71% with an optical purity of 99% ee. The coupling reaction was initially attempted using triphenylphosphine as the ligand, which resulted in poor conversion of compound **2** to compound **3** even stiring longer period of time >24 h.

The 8-phenyl imidazodiazepine ethyl ester (**3**) was subjected to base hydrolysis for the conversion of the ester function into the carboxylic acid function. The ester hydrolysis went smoothly to give carboxylic acid, **4** in good yield and optical purity. The ester hydrolysis was also performed using sodium hydroxide as a base. The lithium hydroxide was chosen for its superiority for the hydrolysis of ester functions as sodium hydroxide caused the solution to form a gel of the carboxylic acid during the isolation. It is difficult to isolate the carboxylic acid once it forms a gel. No gel issue was observed in the case of lithium hydroxide. The acid was purified with hot ethanol. The carboxylic acid **4** was then converted into the acid chloride by a process related to Vilsmeier chlorination. The nucleophilic attack of dimethylamine (2 M solution in THF) on the acid chloride intermediate produced compound **5**. The compound was purified on silica gel column chromatography using 100% EtOAc.

**Scheme 2:**
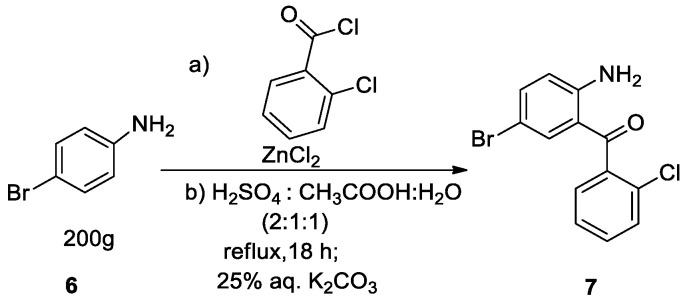
Synthesis of (2-amino-5-bromophenyl)(2-chlorophenyl)methanone **7**. Friedel Crafts acylation of 4-bromo aniline. Reagents and conditions. (**a**) 2-chloro benzoyl chloride (3 eq), 100 °C, 30 min, then anhydrous ZnCl_2_ (2 eq), 220 °C, 3 h; (**b**) H_2_SO_4_: CH_3_COOH: H_2_O (2:1:1), reflux, 18 h; then 25% aq K_2_CO_3_, DCM, 41.5%.

Several other amide analogs were synthesized by using different commercially available benzophenone starting materials. The 2-amino-5-bromophenyl) (2′-fluorophenyl)methanone,2-amino-5-chlorophenyl) (2′-fluorophenyl) methanone, and 2-amino-5-bromophenyl)(pyridin-2-yl)methanone were purchased from chemical vendors while the 2′Cl benzophenone, the 2-(amino-5-bromophenyl) (2′-chlorophenyl) methanone **6** were synthesized, according to the literature method in the lab on a 200 g scale due to its high cost [61,62,63]. This is a one-pot reaction, and this reaction gives a poor yield (41%). Several points need to be followed to improve this reaction. The reaction was performed under neat conditions and 3 equivalents of o-chlorobenzoyl chloride were used to complete this reaction successfully. The first equivalent of o-chlorobenzoyl chloride was used to protect the amine group of 4-bromo aniline and the other two equivalents served as the acylating reagent, as well as the solvent for the process (Scheme 2). The temperature is also an important factor in this reaction. No Friedel-Crafts acylation was observed until the temperature reached 220–230 °C. The formation of an acylium ion was the key intermediate for the successful completion of the reaction [61]. The reagents and apparatus need to be completely anhydrous to perform this reaction, otherwise, trace amounts of moisture will destroy the acylium ion. The benzamide required hydrolysis under harsh conditions, as reported, to deprotect the amine. A mixture of concentrated H_2_SO_4_, CH_3_COOH, and H_2_O was used in a ratio of 2:1:1 to perform this hydrolysis. The reaction mixture was heated to reflux for 18 h and the crude product was purified by an acid-baseextraction, followed by silica gel column chromatography (EtOAc: hexane =1:9).

**Scheme 3:**
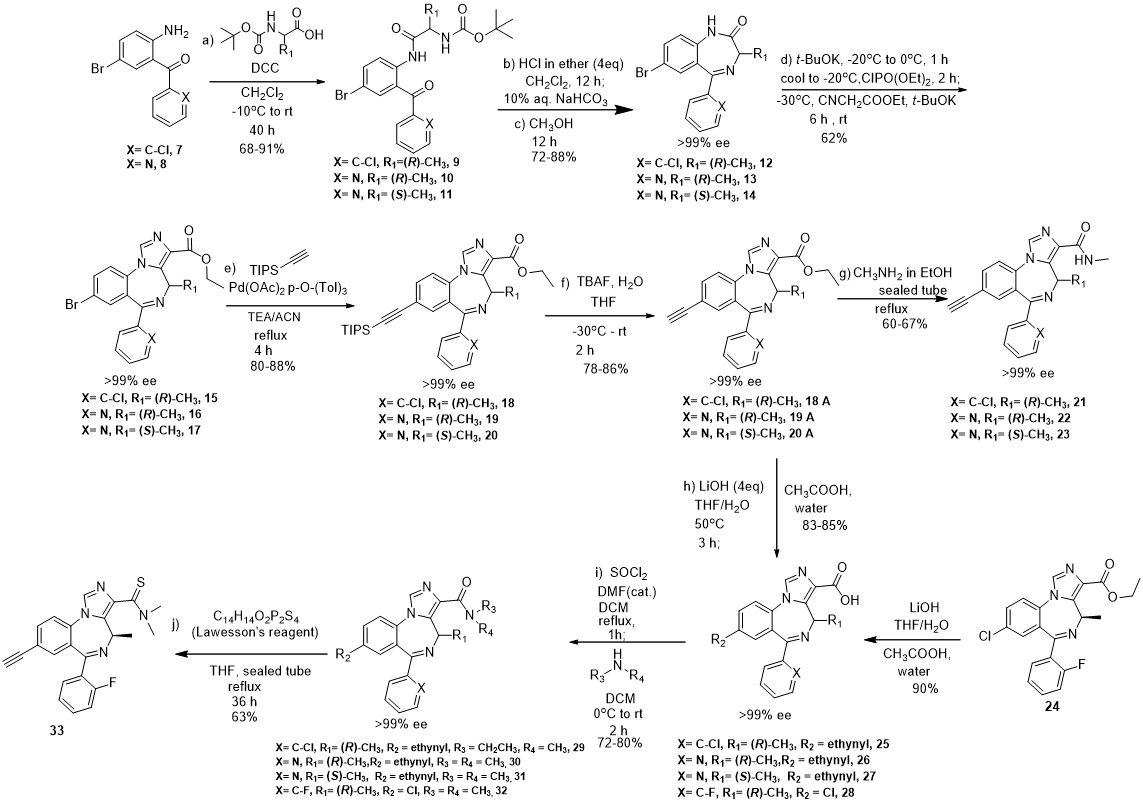
Synthesis of amide analogs of IMDZ 1. Reagent and conditions. (**a**) *N*-Boc-L-Ala-OH or *N*-Boc-D-Ala-OH (1.4 eq), *N*,*N*′-Dicyclohexylcarbodiimide (DCC, 1.6 eq), −10 °C to rt, 40 h, 68–91%; (**b**) 2 M HCl in ether or 4 M HCl in dioxane (4 eq), rt, 12 h; 10% aq, NaHCO_3_ work up; (**c**) MeOH, rt, 12 h, 72–88%, >99% ee; (**d**) t-BuOK (1.3 eq), −20 °C, 1 h, then ClPO(OEt)_2_ (1.4 eq), −20 °C, 2 h, then CNCH_2_COOEt (1.4 eq), −30 °C to rt, 6 h, 62%, >99% ee; (**e**) Pd(OAc)_2_ (5 mol%), tri-*o*-tolyl phosphine (10 mol%), ACN under argon, 15 min, rt, then TEA (3 eq), TIPS-acetylene (1.3 eq), reflux, 4 h, 80–88%, >99% ee; (**f**) TBAF (1 M in THF, 1.3 eq), THF, rt, 2 h, 78–86%, >99% ee; (**g**) CH_3_NH_2_ (88% in EtOH, excess), sealed tube, reflux, 12 h, 60–67%; (**h**) LiOH (4 eq), THF:H_2_O (4:1), 50 °C, 2 h; then CH_3_COOH (5.5 eq, pH ~4–5) (85%, >99%ee); (**i**) SOCl_2_ (10 eq), DMF (cat.), DCM, reflux (35–40 °C), 1 h; then amine (1 M–2 M solution in THF, 6 eq), DCM, 0 °C to rt, 2 h, 70–80%, >99% ee; (**j**) C_14_H_14_O_2_P_2_S_4_ (Lawesson’s reagent, 5 eq), THF, sealed tube, reflux, 36 h, 63%.

The peptide coupling reaction between starting amines (**6** and **8**), and the chiral amino acid was performed in the presence of a peptide coupling reagent, *N*, *N*’-dicyclohexylcarbodiimide (DCC) (Scheme 3). The treatment of **6** and **8** with *R* and *S* amino acids, *N*-Boc-D-Ala-OH and *N*-Boc-Ala-OH individually in the presence of DCC led to the formation of amides **9**–**11** in various yields (72–88%) on different gram scales. An interesting observation was found here. The yield of the peptide coupling reaction for the 2′ *N*-pyridino benzophenone was higher than the 2′Cl benzophenone. These peptide analogs were purified from an EtOAc-hexane reslurry. No column chromatography was involved to purify these analogs. The amide intermediates **9**–**11** underwent acid-mediated Boc-deprotection and subsequent cyclization in methanol under neutral conditions to produce the benzodiazepine intermediates **12**–**14** in good yield, respectively (Scheme 3) in 99% ee. The third step was the synthesis of the imidazodiazepines (**15**–**17**) by installing the imidazole ring on the seven-member ring of benzodiazepines **12**–**14** (Scheme 3). The 1.3 equivalents of potassium *tert*-butoxide in THF solution were used to deprotonate the benzodiazepines, followed by the addition of diethylchlorophosphate to form the iminophosphate intermediate at −30 °C. Then a second portion of base and ethyl isocyanoacetate were added at −20 °C. No chromatography was required to purify the product. Pure imidazodiazepines (**15**–**17**) were obtained by washing the crude solid with *tert* -butyl methyl-ether. The hot *tert* -butyl methyl-ether wash sufficiently removed any traces of byproducts related to the phosphate-related byproducts. The next step in the reaction sequence was the conversion of aryl bromides **15**–**17** into the silyl-protected ethynyl group by a copper-free Sonogashira coupling [64,65]. Pd plays a role in both cycles of the reaction. The copper free Sonogashira coupling of imidazodiazepine intermediate with TIPS-acetylene led to the formation of TIPS-protected imidazodiazepines **18**–**20** in 78–86% yield.

Then the fluoride-mediated deprotection of the silyl protecting group resulted in the formation of 8-ethynyl imidazodiazepines **18**–**20,** respectively. The careful addition of TBAF (1 M solution of THF) at a temperature of −10 °C resulted in successful deprotection of the TIPS group and produced **18**–**20** in various yields. These compounds were purified from a methanol-water mixture by removing the impurities related to tetrabutyl ammonium salts with no chromatography necessary.

The ethyl esters **18**–**20**, and **24** were then hydrolyzed into the corresponding acids **25**–**28** by using a base. Compound **24** was made earlier according to the literature procedure [47]. Previously, NaOH was used as a base to hydrolyze the esters. There was no issue with the hydrolytic capacity of NaOH but a gel was formed during the workup of the reactions mentioned earlier. Acetic acid needed to add to neutralize the excess base and to precipitate the acidic product with continuous stirring. However, the gel formation made the stirring almost impossible. In the new method, LiOH was used as the base. This resolved the gel formation problem; it did not occur. The carboxylic acids were purified from hot ethanol by recrystallization. The acids (**25**–**28**) were converted into acid chlorides, respectively, by a Vilsmeier-related chlorination process. The desired amide analogs were achieved by incorporating various substituted nucleophilic amines. (**29**–**32**) of IMDZ (**1**) with 99% ee. The yield of these amides was in the range of 70–80%. The amides (**29**–**32**) were purified by silica gel column chromatography individually using 100% ethyl acetate.

Thioamides are considered as one of the important bioisosteres of an amide function. The sulfur atom is relatively larger in size and lower in electronegativity than an oxygen atom. Consequently, the sulfur atom is able to accept more charge density transferred from the nitrogen atom in a thioamide, as compared to an amide moiety due to its larger size of the former [66]. Based on these characteristics, a thioamide analog, **33** was prepared. The thioamide **33** was synthesized by using Lawesson’s reagent (LR) [67,68] in refluxing THF for 36 h in a sealed tube, as illustrated in Scheme 3. The LR needs to be handled very carefully in a fume hood because of its unpleasant odor. A reactive dithiophosphine ylide forms from the Lawesson’s reagent in solution at the beginning of the reaction, which is the key for this process. Once the reaction was done, the solvents were removed under reduced pressure, and the contents were loaded onto a column under a fume hood. A mixture of EtOAc:hexane (2:3) was used to remove the excess LR and impurities and this was followed by collection of the pure product from EtOAc:hexane (4:1). The yield of this reaction was 63%.

#### 2.1.2. Oxadiazole Analogs of IMDZ 1

**Scheme 4:**
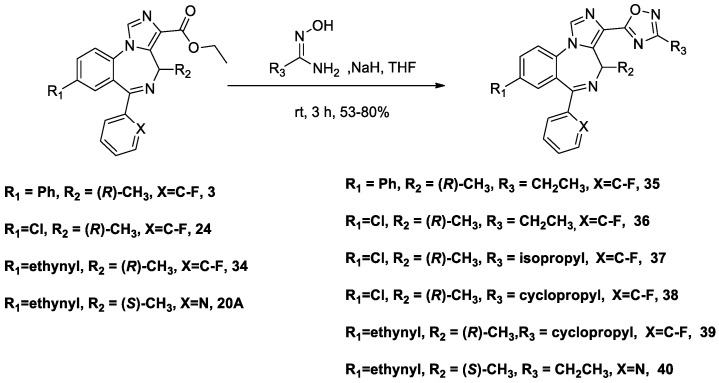
Synthesis of 1,2,4-oxadiazoles. Reagents and conditions. Ethyl, isopropyl or cyclopropyl oximes (4 eq), NaH (60% dispersion in mineral oil, 1.1 eq), THF, molecular sieves (3 Å), 1 h, rt; then add ethyl esters (1 eq), rt, 2 h, 53–80% yield.

Oxadiazole rings play an important role in the field of drug discovery, and they have been introduced in these programs for a variety of reasons. For instance, they contribute to ligand binding as an essential part of the pharmacophore [69]. In many cases, these moieties serve as flat, aromatic-like linkers where the substituents are placed in the proper orientation [70]. Oxadiazoles can also modulate the molecular properties by incorporating them in the periphery of the molecule [71]. Finally, oxadiazoles are also employed as bioisosteric replacements for carbonyl-containing compounds such as esters and amides [72,73,74]. Oxadiazole rings can exist in multiple regioisomeric forms such as the two available 1,2,4-isomers (if asymmetrically substituted), a 1,3,4- isomer and a 1,2,5-isomer, among which the 1,2,5-regioisomer is less common than the other two isomers [75,76]. Hydrogen bond acceptor properties are also exhibited by oxadiazoles and different hydrogen bonding properties are observed by the regioisomers. Taking into consideration these factors, a series of 1,2,4-oxadiazole analogs were synthesized, specifically designed to resemble the structure of esters 18, 19, 20, and amide IMDZ 1 (1). The 1,2,4-oxadiazole analogs of IMDZ 1 were synthesized from the different imidazodiazepine ethyl esters bearing different substituents (Scheme 4). The ethyl esters were treated with different oximes (ethyl, cyclopropyl, and isopropyl) in the presence of sodium hydride in order to form the 1,2,4-oxadiazoles [48].

**Figure 3:**
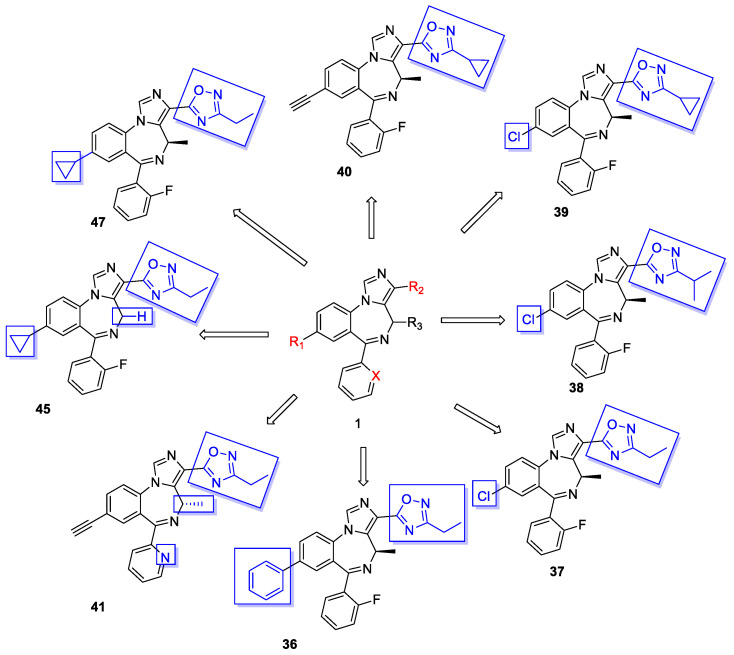
Oxadiazole analogs of IMDZ **1**.

**Figure 4:**
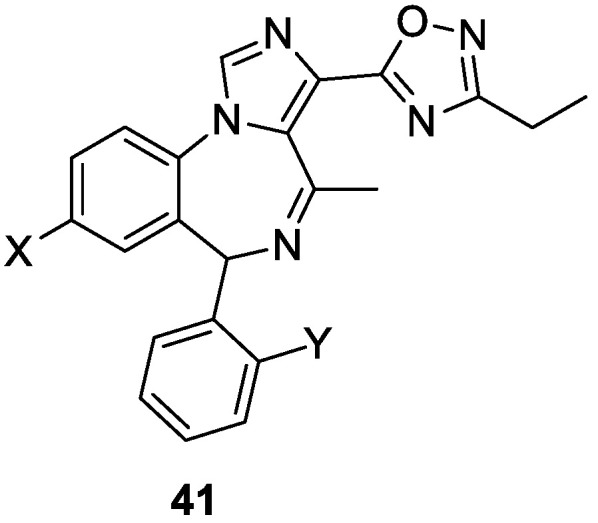
The olefinic migrated product.

The oximes were treated with sodium hydride in THF for an hour and then a solution of the ethyl ester (**3**, **24**, **34**, **and 20 A**) in THF was added to it. Compound **24** and **34** was synthesized according to the procedure outlined in the literature [47] (Supplementary Materials). Caution should be employed regarding the amount of sodium hydride. If the hydride is not very good, the reaction does not go to completion. If the hydride is good and the equivalents are higher than 1.3, an olefinic migration of the C=N double bond can occur and a trisubstituted enamine by-product can form. It is non-polar and easily differentiated from the corresponding oxadizole products on silica gel (TLC). An olefinic migrated by-product was observed in the case of compound **36** (**41**, Figure 3 and Figure 4). That is why the yield for this reaction was only 53%. In other cases, the yield varied from 73 to 80%, respectively.

**Scheme 5:**
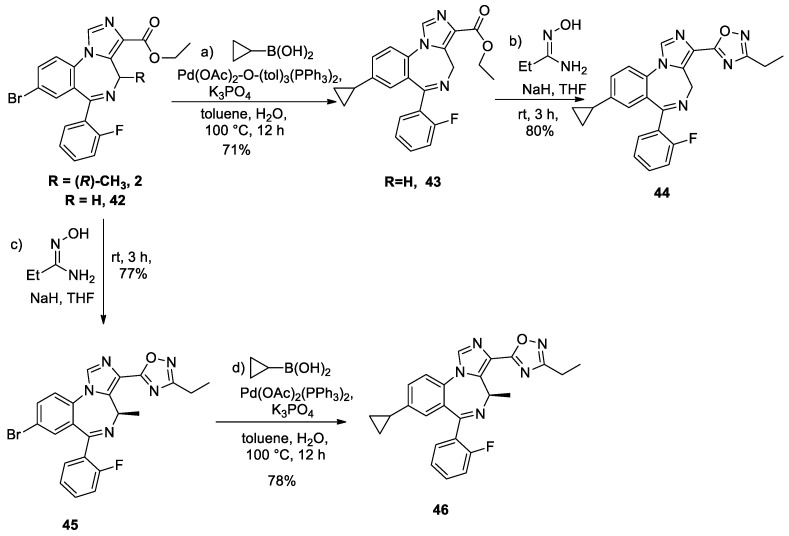
8-Cyclopropyl oxadiazole compounds. Reagents and conditions. (**a**,**d**) Pd(OAc)_2_ (5 mol%), tri-*o*-tolyl phosphine (10 mol%), toluene under argon, 15 min, rt; then K_3_PO_4_ (3 eq), H_2_O (0.2 eq), phenyl brornic acid (3 eq), 100 °C, 12 h, 71–78%; (**b**,**c**) Ethyl oxime (4 eq), NaH (60% dispersion in mineral oil, 1.1 eq), THF, molecular sieve (3 Å), 1 h, rt; then add ethyl esters (1 eq), rt, 2 h, 77–80%.

The 8-cyclopropyl oxadiazole analog, **46** was synthesized from 8-bromo imidazodiazepine ester (**2**) by treatment with NaH in THF with ethyl oxime and this was followed by Suzuki coupling with cyclopropyl boronic acid. A non-chiral version of **46,** compound **44** was prepared in similar fashion (Scheme 5). These ligands are now being tested in the Y-maze assay and FST to determine their procognitive and anti-depressive profile.

### 2.2. Receptor Binding Studies

To determine if any off-target receptor-binding effects are present, all the novel ligands were sent to the National Institute of Mental Health (NIMH), Psychoactive Drug Screening Program (PDSP). The ligands were screened over a panel of 47 receptors, ion channels, and transporters to determine any other possible interactions with the GABAA receptor. The hERG-binding was also determined. Any ligands that showed significant inhibition (over 50%) in the primary binding screens were further tested in the secondary binding assay to obtain a Ki (nM) value. Most of the ligands showed binding affinity at GABAARs but this data did not represent the individual binding affinity of different Bz alpha/beta/gamma2-subtypes but was on synaptosomal membranes. No binding of the test compounds to calcium channels was observed. This evidence suggested no physiological events on cardiac and smooth muscle cells [77], nor NMDA receptors [78] by these analogs. It is necessary to screen the compounds for hERG-binding during the early process of drug discovery. The unintentional binding of many drugs to hERG can cause blockade of hERG K+ channels in the heart, which can cause many side effects including cardiac arrhythmias and sudden death [79]. Hence, it is essential to screen compounds for hERG activity to assess any potential off-target effects of the test compounds.. All of the α5 imidazodiazepines were tested for hERG-binding. The test compounds did not bind to undesirable voltage-sensitive K+ hERG channels (Table 1).

The compounds were also screened at PDSP to determine any binding with peripheral benzodiazepine receptors (PBR). A number of biological processes are associated with PBR. The most prevalent are transportation of anions for mitochondrial transmembrane potential, biosynthesis of heme, regulation of neurological damage, proliferation, cell death (apoptosis), as well as regulation of steroidogenesis [80]. The test compounds were screened in the primary binding assay to determine the % inhibition at PBR. A number of compounds were found to inhibit more than 50% in the primary binding assay. These compounds were then subjected to a secondary screening to determine the K_i_ (nM) value for PBR receptors. Secondary screening results revealed that no ligand was found to bind to PBR at greater than 550 nM, which is considered very weak affinity. The structures of the compounds and binding affinity at PBR are summarized in Table 2.

Along with above mentioned receptors, binding affinity of a few imidazodiazepines was also exhibited at kappa opioid receptors (KOR). Opioid receptors are a family of inhibitory G-coupled protein receptors where opioids act as the ligand. The kappa opioid receptors (KOR), µ and δ opioid receptors (MOR and DOR), and nociception opioid receptor (NOP) fall under the family of opioids receptors [81]. It has been reported that oxadiazole imidazodiazepines can mediate analgesic and anti-inflammatory properties mediated also by the κ opioid receptor (KOR) [82]. The binding at KOR is usually silent. The affinities of the test compounds for KOR, DOR, and MOR were determined using radioligand binding assays at a screening concentration of 10,000 nM in collaboration with the National Institute of Mental Health’s Psychoactive Drug Screening Program (PDSP), as mentioned before. Concentration-dependent experiments were conducted to determine **Ki** values for the compounds that exceeded more than 50% radioligand displacement in primary binding assays. Many of these proposed alpha-5 selective imidazodiazepines exhibited some KOR affinities and the % KOR inhibition and K_i_ values for chiral and achiral imidazodiazepines are summarized in Table 3.

The imidazodiazepine, **40** exhibited the highest affinity for KOR (Ki = 49.8 nM) (Table 3, entry 11). This ligand in the series also exhibited a little affinity for MOR (49% inhibition). It was observed that oxadiazoles bearing different substitutions showed appreciable KOR binding affinity (Table 3, compounds **39, 40, 41, 47,** and **49**). One oxadizole, the imidazodiazepine containing the C-8 phenyl group did not show mentionable binding affinity towards KOR but it showed a reasonable affinity towards DOR (Table 3, compound **38**). Imidazodiazepines containing bromo, chloro- or a cyclopropyl- group at the C-8 position showed superior KOR binding affinity than phenyl or ethyinyl at the C-8 position (Table 3). The BZR binding affinity of the test compounds was also measured using rat brain BzR. A few of the compounds showed good to moderate binding affinity towards these Bz receptors as predicted. The efficacy of imidazodiazepines on the benzodiazepine receptor depends on various factors, including the specific subtype of the receptor they bind to, their chemical structure, and the dose. The data are presented in Table 4.

Only three of the newly synthesized ligands demonstrated promising results in in-vitro assays on BzR. These are currently being investigated in in vivo animal models to characterize their putative anxiolytic, antidepressant, and pro-cognitive effects and identify any possible CNS side effects It is important to note that imidazodiazepines are a distinct class of compounds and should not be confused with benzodiazepines. Unlike benzodiazepines, many imidazodiazepine ligands have shown clinical potential without causing ataxia, sedation, and dependence side effects, often reported with benzodiazepines.

## 3. Materials and Methods

All reactions were performed in round-bottom flasks with magnetic stir bars or overhead stirs under an argon atmosphere. Organic solvents were purified when necessary by standard methods or purchased from Sigma-Aldrich Chemicals. The reagents and other chemicals were purchased from either Sigma-Aldrich, Oakwood Chemical, Alfa Aesar, Matrix Scientific, Admiral Chemical Company, or Acros Organic. The progress of reactions was visualized with TLC plates from Dynamic Adsorbents, Inc. under a UV light. An LCMS 2020 was used to monitor the progress of some reactions. The flash column chromatography was carried out for purification of some analogs on silica gel (230–400 mesh, Dynamic Adsorbents). A normal phase Agilent HPLC was used to determine the ratio of optically active enantiomers, as well as to determine %ee. The ^1^H NMR and ^13^C NMR spectra were obtained on a Bruker Spectrospin 500 MHz instrument in CDCl3 and chemical shifts were reported in δ (ppm). Multiplicities are represented as follows: singlet (s), broad signal (br), doublet (d), triplet (t), quartet (q), dd (doublet of doublets), and multiplet (m). The technique employed for HRMS was carried out on a LCMS-IT-TOF at the Milwaukee Institute for Drug Discovery in the Shimadzu Laboratory for Advanced and Applied Analytical Chemistry.

### 3.1. Synthetic Procedures

#### 3.1.1. Synthesis of Ethyl(R)-6-(2′-fluorophenyl)-4-methyl-8-phenyl-4H-benzo[f]imidazo[1,5-a][1,4]diazepine-3-carboxylate (3)

Pd(OAc)_2_ (0.1 g, 0.45 mmol), tri-(*o*-tolyl) phosphine (0.274 g, 0.9 mmol) were dissolved in toluene (10 mL) and the mixture was stirred for 10 min under an argon atmosphere to generate the Pd(OAc)2-p-*o*-(tol)3-phosphine catalyst in-situ. Then the imidazodiazepine, ethyl(*R*)-8-bromo-6-(2′-fluorophenyl)-4-methyl-4H-benzo [f]imidazo[1,5-a][1,4]diazepine-3-carboxylate (**2**, 2.5 g, 5.63 mmol), phenyl boronic acid (2.05 g, 16.8 mmol), tri-basic potassium phosphate (5.37 g, 25 mmol), water (0.5 mL, 25 mmol), and additional toluene (15 mL) were added sequentially to the previous reaction mixture under argon. The reaction mixture was then stirred at 100 °C for 12 h until the consumption of starting material was confirmed by LCMS 2020 (single quadrupole mass analyzer). The R_f_ value on TLC (silica gel; neutral alumina; 60% ethyl acetate-hexane, R_f_ = 0.4) for both the reactant and product were almost identical. That is why the completion of the reaction required confirmation by LCMS 2020. The reaction mixture was cooled and opened to the air once all the starting material was consumed. The reaction mixture was passed through a pad of celite beads to remove any palladium salts. The filtrate was diluted with water (20 mL) and ethyl acetate (30 mL). The biphasic mixture, which resulted, was allowed to stand to separate. The organic layer was collected and the aq layer was extracted (2 × 10 mL). The combined organic layer was washed with 10% aq NaCl (3 × 10 mL) and dried (Na_2_SO_4_). The solvents were removed under reduced pressure. The orange-colored residue, which resulted, was purified by flash chromatography (silica gel 100 g, 80% EtOAc-hexane). The desired fractions were pooled, and the solvents were removed. The solid residue was dried under vacuum for 2 h to afford an off-white-colored powder of **3** (1.4 g, 71%). **^1^H NMR** (500 MHz, CDCl_3_) δ 7.99 (s, 1H), 7.81 (d, J = 8.1 Hz, 1H), 7.67 (d, J = 8.3 Hz, 1H), 7.64 (t, J = 7.3 Hz, 1H), 7.51–7.41 (m, 6H), 7.39 (t, J = 7.1 Hz, 1H), 7.25 (t, J = 7.5 Hz, 1H), 7.03 (t, J = 9.2 Hz, 1H), 6.73 (q, J = 7.2 Hz, 1H), 4.46–4.35 (m, 2H), 1.43 (t, J = 7.1 Hz, 3H), 1.34 (d, J = 7.3 Hz, 3H).

**^13^C NMR** (126 MHz, CDCl_3_) δ 164.04 (s), 163.09 (s), 160.19 (d, ^1^J_C-F_ = 250.3 Hz), 141.74 (s), 140.47 (s), 138.73 (s), 134.91 (s), 133.70 (s), 131.80 (d, ^3^J_C-F_ = 8.1 Hz), 131.27 (s), 130.41 (s), 129.84 (s), 129.36 (s), 129.07 (s), 128.90 (s), 128.31 (s), 127.09 (s), 124.45 (d, ^4^J_C-F_ = 3.0 Hz), 122.47 (s), 116.15 (d, ^2^J_C-F_ = 21.6 Hz), 60.72 (s), 50.16 (s), 14.85 (s), 14.47 (s). HRMS (ESI/IT-TOF) *m*/*z*: [M + H]+ Calcd for C_27_H_22_N_3_O_2_F 440.1769; found 440.1745; %ee > 99.0% (Chiral pak IBN3-4.6 mm × 150 mm, 3 µm).

#### 3.1.2. Synthesis of (R)-6-(2′-Fluorophenyl)-4-methyl-8-phenyl-4H-benzo[f]imidazo[1,5-a][1,4]diazepine -3-carboxylic acid (4)

The ethyl ester, ethyl(*R*)-6-(2′-fluorophenyl)-4-methyl-8-phenyl-4H-benzo[f]imidazo[1,5-a][1,4]diazepine-3-carboxylate (**3**, 1.3 g, 2.9 mmol) was dissolved in THF (20 mL) and a solution of LiOH (0.28 g, 12 mmol) in water (5 mL) was added dropwise to the former THF solution. The mixture was then stirred for 1 h at 50 °C. The completion of the reaction was confirmed by TLC (silica gel, 100% EtOAc, R_f_ of **3** = 0.5; R_f_ of Li salt of **4** = base line). At the end of the reaction, the mixture was cooled to rt. Then water (20 mL) was added to dilute the mixture and acetic acid (0.82 mL, 14.5 mmol) was added to adjust the pH to 4–5. The THF was removed under reduced pressure and the mixture was kept for 5 h to maximize the precipitation. The solid reside, which resulted, was filtered, washed with water (4 × 10 mL), and dried under vacuum. The residue was then slurried with EtOAc (10 mL) and stirred at 50 °C for 10 min. The mixture was cooled to rt and held there for 3 h. The solid, which formed, was filtered, washed with EtOAc (2 × 10 mL), and dried under vacuum at 40 °C for 2 h to afford a white powder of **4** (1.0 g, 84%). **^1^H NMR** (500 MHz, MeOD) δ 8.45 (s, 1H), 8.02 (s, 2H), 7.63 (t, J = 7.1 Hz, 1H), 7.59–7.50 (m, 3H), 7.45 (t, J = 7.5 Hz, 2H), 7.42–7.37 (m, 2H), 7.33 (t, J = 7.5 Hz, 1H), 7.20 (t, J = 9.3 Hz, 1H), 6.56 (q, J = 7.1 Hz, 1H), 1.21 (d, J = 7.0 Hz, 3H).

**^13^C NMR** (126 MHz, DMSO-d6) δ 164.84 (s), 163.67 (s), 159.91 (d, ^1^J_C-F_ = 248.0 Hz), 140.90 (s), 139.37 (s), 138.42 (s), 136.52 (s), 133.97 (s), 132.47 (s), 131.96 (s), 130.84 (s), 129.67 (s), 129.62 (s), 129.27 (d, ^3^J_C-F_ = 12.5 Hz), 128.76 (s), 128.07 (s), 127.16 (s), 125.11 (d, ^4^J_C-F_ = 2.4 Hz), 123.96 (s), 116.35 (d, ^2^J_C-F_ = 21.3 Hz), 49.82 (s), 15.06 (s).HRMS (ESI/IT-TOF) *m*/*z*: [M—H]- Calcd for C_27_H_22_N_3_O_2_F 410.1310; found 410.1312; %ee > 99.0% (Chiral pak IBN3-4.6 mm × 150 mm, 3 µm).

#### 3.1.3. Synthesis of (R)-6-(2′-Fluorophenyl)-N,N,4-trimethyl-8-phenyl-4H-benzo[f]imidazo[1,5-a][1,4]diazepine-3-carboxamide (5)

A mixture of **4**, (*R*)-6-(2′-fluorophenyl)-4-methyl-8-phenyl-4H-benzo[f]imidazo[1,5-a][1,4]diazepine-3-carboxylic acid (350 mg, 0.85 mmol), thionyl chloride (0.6 mL, 8.5 mmol), a catalytic amount of anhydrous *N*,*N*-dimethyl formamide (0.1 mL, 0.085 mmol) and anhydrous DCM (20 mL) were charged into an oven-dried round-bottom flask under argon. Thesuspension, which resulted, was allowed to reflux at 50 °C for 1 h under argon. The organic solvent and excess thionyl chloride were removed under reduced pressure on a rotary evaporator and the evaporation repeated five times with anhydrous DCM (5 × 10 mL). The resulting yellow residue was dissolved in anhydrous DCM (20 mL) and cooled to 0 °C for 10 min under argon, followed by addition of a solution of *N*,*N*-dimethylamine (4.25 mL, 8.5 mmol). The mixture was then allowed to warm to rt and stirred for an hour. After the completion of the reaction by TLC (silica gel), the mixture was diluted with ice cold water (15 mL) and extracted with DCM (3 x 20 mL). The combined organic layer was washed with brine (20 mL), dried (Na_2_SO_4_) and the residue was purified by silica gel flash chromatography (EtOAc and 1% trimethylamine) to furnish pale yellow solid of **5** (0.29 g, 80%). **^1^H NMR** (500 MHz, CDCl_3_) δ 7.98 (s, 1H), 7.83 (d, J = 7.9 Hz, 1H), 7.68–7.64 (m, 1H), 7.62 (d, J = 8.2 Hz, 1H), 7.52–7.43 (m, 6H), 7.41–7.37 (m, 1H), 7.25 (t, J = 7.5 Hz, 1H), 7.02 (ddd, J = 9.2, 1.6, 0.8 Hz, 1H), 4.40 (q, J = 6.7 Hz, 1H), 3.14 (s, 3H), 3.03 (s, 3H), 1.96 (d, J = 6.6 Hz, 3H)^. **13**^**C NMR** (126 MHz, CDCl_3_) δ 166.37 (s), 163.44 (s), 160.30 (d, ^1^J_C-F_ = 251.4 Hz), 140.39 (s), 138.96 (s), 133.84 (s), 131.93 (d, ^3^J_C-F_ = 8.1 Hz), 131.38 (d, ^4^J_C-F_ = 2.4 Hz), 130.67 (s), 129.52 (s), 129.04 (s), 128.61 (s), 128.21 (s), 127.14 (s), 124.46 (s), 123.07 (s), 116.15 (d, ^2^J_C-F_ = 21.5 Hz), 51.97 (s), 38.46 (s), 34.77 (s), 18.12 (s) HRMS (ESI/IT-TOF) *m*/*z*: [M + H] + Calcd for C_27_H_23_FN_4_O 439.1929; found 439.1908. %ee >99% (HPLC) (Chiral pak IBN3-4.6 mm × 150 mm, 3 µm).

#### 3.1.4. Synthesis of (2-Amino-5-bromophenyl)(2-chlorophenyl)methanone (7)

A round-bottom flask was charged with 2-chlorobenzoyl chloride (515.3 mL, 4069 mmol) and then it was heated to 100–120 ^°C^ under an argon atmosphere. At that point, 4-bromoaniline (200 g, 1163 mmol) was added and the mixture was stirred for 5 min at 100–120 °C. Evolution of HCl (g) occurred and a clear homogenous solution resulted after the evolution of all HCl(g). The mixture was then heated. When the temperature reached 200–210 °C, anhydrous ZnCl_2_ (317 g, 2326 mmol) was added to the reaction mixture. The evolution of HCl (g) was also observed at this point. The mixture was allowed to heat to 200–220 °C for 3 h, at which point the evolution of HCl(g) was completed. The reaction mixture was then cooled to 120 °C and water (400 mL) was added slowly. The mixture was allowed to reflux for 30 min and the hot water was decanted. This step was repeated 5 times. The mixture was then dissolved in a solution of H_2_SO_4_ (400 mL), acetic acid (200 mL), and water (200 mL). The reaction mixture was stirred for 24 h under reflux conditions. The mixture was then cooled to rt and basified slowly by adding aq 25% K_2_CO_3_ solution (3000 mL). Then ethyl acetate (3000 mL) was added, and the mixture was stirred for 10 min. The biphasic mixture, which resulted, was allowed to stand for 20 min to separate the layers. The layers were separated and the aq layer was extracted with ethyl acetate (2 × 400 mL). The combined organic layers were washed with aq 25% K_2_CO_3_ solution (1 × 2000 mL), aq 10 % NaCl (2 × 1000 mL), and dried (MgSO_4_). The ethyl acetate was removed under reduced pressure. The residue was dissolved in DCM (500 mL) and silica gel was added to remove the gummy black residue. The mixture was stirred for 1 h and the silica gel was filtered off, washed with DCM (2 × 400 mL). The solvents were removed under reduced pressure and the residue was purified by column chromatography (silica gel, 100% hexane to 10% EtOAc-hexane). The desired fractions were pooled, and the solvents were removed under reduced pressure. The solid residue was dried under vacuum to afford pure **7** as a yellow-colored powder (126 g, 39%). R_f_ = 0.6 (silica gel, 10% EtOAc-hexane). **^1^H NMR** (500 MHz, CDCl_3_) δ 7.50–7.47 (m, 1H), 7.44 (td, J = 7.7, 1.4 Hz, 1H), 7.40–7.35 (m, 2H), 7.32 (dd, J = 7.5, 1.2 Hz, 1H), 7.28 (d, J = 2.1 Hz, 1H), 6.64 (d, J = 8.8 Hz, 1H), 6.52 (s, 1H). **^13^C NMR** (126 MHz, CDCl_3_) δ 196.36 (s), 150.27 (s), 138.97 (s), 137.90 (s), 136.19 (s), 130.81 (s), 130.73 (s), 130.10 (s), 128.44 (s), 126.84 (s), 118.94 (s), 118.63 (s), 106.70 (s). **HRMS** (ESI/IT-TOF) *m*/*z*: [M + H] Calcd for C_13_H_9_NOClBr 309.9628; found 309.9632.

#### 3.1.5. Synthesis of Tert-butyl(R)-(1-((4-bromo-2-(2′-chlorobenzoyl)phenyl)amino)-1- oxopropan -2-yl) carbamate (9)

The benzophenone **7**, (2-amino-5-bromophenyl)(2′-chlorophenyl)methanone (50 g, 161 mmol), and Boc-D-Ala-OH (60.9 g, 322 mmol) were dissolved in DCM (500 mL) and a solution of DCC (66.43 g, 322 mmol) in DCM (150 mL) was added dropwise to the previous mixture at −10 °C over a period of 30 min. The mixture was then allowed to warm to rt and stirred for 30 h. The white-colored dicyclohexyl urea, which formed, was filtered off and washed with DCM (3 × 100 mL) unless the residue was completely white. The DCM was removed under reduced pressure and the oily mass was purified by column chromatography (silica gel, 3% EtOAc-hexane to 10% EtOAc-hexane). The required fractions were collected, and the solvents were evaporated under reduced pressure. The residue was dried under vacuum for 3 h to afford a light, yellow-colored powder of **9** (50.41 g, 65%). **^1^H NMR** (500 MHz, CDCl_3_) δ 11.90 (s, 1H), 8.77 (d, *J* = 9.1 Hz, 1H), 7.69 (dd, *J* = 9.0, 2.1 Hz, 1H), 7.51–7.49 (m, 2H), 7.46 (d, *J* = 2.0 Hz, 1H), 7.42 (ddd, *J* = 7.6, 5.5, 3.1 Hz, 1H), 7.34–7.31 (m, 1H), 5.20 (q, *J* = 28.7 Hz, 1H), 4.39 (s, 1H), 1.54 (d, *J* = 7.3 Hz, 3H), 1.46 (s, 9H). **^13^C NMR** (126 MHz, CDCl_3_) δ 197.77 (s), 176.81 (s), 172.68 (s), 140.25 (s), 138.36 (s), 137.93 (s), 136.33 (s), 131.66 (s), 131.04 (s), 130.32 (s), 128.78 (s), 126.93 (s), 123.49 (s), 122.57 (s), 114.93 (s), 51.93 (s), 28.31 (s), 18.58 (s), 18.45 (s). **HRMS** (ESI/IT-TOF) *m*/*z*: [M + Na]^+^ Calcd for C_21_H_22_N_2_O_4_ClBr 503.0343; found 503.0343.

#### 3.1.6. Synthesis of Tert-butyl(S)-(1-((4-bromo-2-picolinoylphenyl)amino)-1-oxopropan-2-yl) carbamate (11)

The commercially available benzophenone, (2-amino-5-bromophenyl)(pyridin-2-yl)methanone (**8**, 50 g, 180 mmol) and BOC-L-alanine (44.5 g, 234 mmol) were dissolved in DCM (400 mL), and the mixture was cooled to −10 °C using an ice-methanol bath. A solution of peptide coupling reagent DCC (59.4 g, 288 mmol) in DCM (200 mL) was added dropwise to the reaction mixture over a period of 60 min, while the temperature was maintained at −10 °C to −5 °C. The mixture was then allowed to warm to rt and stirred for 30 hr. The consumption of starting material was monitored by TLC (silica gel, 30% EtOAc-hexane, 1% TEA). Once the reaction was done, the white dicyclohexyl urea byproduct was filtered off and the residue was washed with DCM (3 × 100 mL). The combined DCM layers were removed under reduced pressure and a yellow-colored oil resulted. The oil was slurried with 5% EtOAc-hexane (250 mL) and stirred for 30 min at 55 °C. The yellow-colored oil turned into a white-colored powder floating on the flask during this stirring. The mixture was cooled to rt and held for 2 h. The solid, which formed, was filtered, washed with 5% EtOAc-hexane (3 × 50 mL), and dried under vacuum for 2 h at 40 °C to afford pure **11** as a white powder (69.56 g, 86%). R_f_ = 0.4 (silica gel, 30% EtOAc-hexane); **^1^H NMR** (500 MHz, CDCl_3_) δ 11.39 (s, 1H), 8.74 (d, J = 4.3 Hz, 1H), 8.63 (d, J = 9.0 Hz, 1H), 7.98 (d, J = 2.0 Hz, 1H), 7.96–7.92 (m, 2H), 7.68 (dd, J = 9.0, 2.0 Hz, 1H), 7.53 (dd, J = 8.8, 4.7 Hz, 1H), 5.15 (s, 1H), 4.29 (q, 1H), 1.49 (d, J = 7.2 Hz, 3H), 1.46 (s, 9H); **^13^C NMR** (126 MHz, CDCl_3_) δ 195.40 (s), 172.04 (s), 155.28 (s), 155.06 (s), 148.72 (s), 139.86 (s), 137.31 (s), 136.87 (s), 126.46 (s), 124.79 (s), 123.74 (s), 122.80 (s), 114.82 (s), 80.29 (s), 51.66 (s), 28.31 (s), 18.65 (s); **HRMS (ESI/IT-TOF**) *m*/*z*: [M + H] + Calcd for C_20_H_22_N_3_O_4_Br 448.0866; found 448.0899.

#### 3.1.7. Synthesis of Tert-butyl(R)-(1-((4-bromo-2-picolinoylphenyl)amino)-1-oxopropan-2-yl) carbamate (10)

Compound **10** was synthesized using the identical protocol as compound **11**, employing the same scale, and was obtained as a white powder after isolation (68.2 g, 85.2%). R_f_ = 0.4 (silica gel, 30% EtOAc-hexane).

**^1^H NMR** (500 MHz, CDCl_3_) δ 11.40 (s, 1H), 8.78 (dd, *J* = 25.2, 6.7 Hz, 1H), 8.64 (d, *J* = 9.0 Hz, 1H), 7.99 (d, *J* = 1.8 Hz, 1H), 7.95 (d, *J* = 4.3 Hz, 2H), 7.69 (dd, *J* = 9.0, 1.9 Hz, 1H), 7.54 (dd, *J* = 8.9, 4.6 Hz, 1H), 5.12 (s, 1H), 4.37 (q, *J* = 24.3 Hz, 1H), 1.49 (d, *J* = 7.2 Hz, 3H), 1.46 (s, 9H).

^13^C NMR (126 MHz, CDCl_3_) δ 195.41 (s), 172.01 (s), 155.06 (s), 148.72 (s), 139.86 (s), 137.31 (s), 136.87 (s), 126.45 (s), 124.79 (s), 123.71 (s), 122.80 (s), 114.82 (s), 80.31 (s), 51.66 (s), 28.30 (s), 18.67 (s). **HRMS (ESI/IT-TOF**) *m*/*z*: [M + H] + Calcd for C_20_H_22_N_3_O_4_Br 448.0866; found 448.0877.

#### 3.1.8. Synthesis of (R)-7-Bromo-5-(2′-chlorophenyl)-3-methyl-1,3-dihydro-2H-benzo [e][1,4]diazepin-2-one (12)

The Boc protected amide **9** (50 g, 100 mmol) was dissolved in DCM (500 mL) and 2 M HCl in ether (207.5 mL, 400 mmol) was added dropwise to that mixture at −10 °C. The reaction mixture was stirred for 10 h at rt until the consumption of starting material (TLC) was completed. The completion of the reaction was confirmed by TLC (silica gel, 50% EtOAc-hexane). The reaction mixture was neutralized by adding 10% aq NaHCO_3_ solution (450 mL). The layers were separated, and the organic layers were collected. The aq layer was extracted with DCM (2 × 100 mL). The combined organic layers were washed with 10% aq NaHCO_3_ solution (200 mL), and this was followed by 10% aq NaCl solution (2x 250 mL), and then it was dried (Na_2_SO_4_). The solvents were removed under reduced pressure. The oil, which resulted, was dissolved in anhydrous methanol (300 mL) and the mixture was stirred for 6 h at 40 °C. The consumption of starting material was confirmed by TLC (silica gel, 50% EtOAc-hexane). The methanol was removed under reduced pressure. The gummy mass, which resulted, was dissolved in DCM (400 mL) and water (400 mL) was added. The biphasic mixture, which resulted, was separated and the organic layers were collected. The aq layer was extracted with DCM (2 × 100 mL). The combined organic layers were washed with 10% aq NaCl (2 × 200 mL) and dried (Na_2_SO_4_). The solvents were removed under reduced pressure and the residue was purified by flash chromatography (silica gel, 50% EtOAc-hexane). The desired fractions were collected, and the solvents were removed under reduced pressure. The residue was dried under vacuum for 4 h to afford a light-yellow colored solid of **12** (30.5 g, 81%). R_f_ = 0.4 (silica gel, 50% EtOAc-hexane); **^1^H NMR** (500 MHz, CDCl_3_) δ 9.75 (bs, 1H), 7.58 (d, *J* = 8.6 Hz, 1H), 7.52 (d, *J* = 5.5 Hz, 1H), 7.38 (t, *J* = 4.8 Hz, 3H), 7.22 (s, 1H), 7.12 (d, *J* = 8.6 Hz, 1H), 3.83 (q, *J* = 6.4 Hz, 1H), 1.78 (d, *J* = 6.5 Hz, 3H).^**13**^**C NMR** (126 MHz, CDCl_3_) δ 172.24 (s), 167.27 (s), 138.22 (s), 136.93 (s), 134.72 (s), 133.32 (s), 131.88 (s), 131.13 (s), 130.90 (s), 130.18 (s), 129.86 (s), 127.02 (s), 122.81 (s), 116.46 (s), 58.77 (s), 16.89 (s). **HRMS** (ESI/IT-TOF) *m*/*z*: [M + H] Calcd for C_16_H_12_N_2_OClBr 362.9894; found 362.9895. %ee > 99%

#### 3.1.9. Synthesis of (S)-7-Bromo-3-methyl-5-(pyridin-2′-yl)-1,3-dihydro-2H-benzo[e][1,4]diazepin-2-one (14)

A solution of 4 M HCl in dioxane (153.9 mL, 616 mmol) was added dropwise into a stirred solution of the *N*-Boc protected amide, tert-butyl (*S*)-(1-((4-bromo-2′-picolinoylphenyl)amino) -1-oxopropan-2-yl)carbamate **11** (69 g, 154 mmol) in DCM (500 mL) at −10 °C. The solution was allowed to warm to rt and allowed to stir for 12 h at rt. The consumption of the starting material was confirmed by TLC (silica gel, 60% EtOAc-hexane with 1% TEA). At the end of the reaction progress, the mixture was neutralized with aq 5% NaHCO_3_ solution (600 mL) and the organic layer was extracted with dichloromethane (2 × 500 mL). The combined organic layer was washed with aq 5% NaHCO_3_ solution (250 mL) and aq 10% NaCl solution (2 × 350 mL). The solvents were removed under reduced pressure and the oily mass, which resulted, was dissolved in methanol (500 mL). The mixture was stirred for 20 h at rt until the consumption of starting material was observed (TLC). Once the reaction was done, all the solvents were removed under reduced pressure and the residue was dissolved in DCM (1000 mL), after which water (500 mL) was added to dilute the mixture. The biphasic mixture, which resulted, was allowed to stand for 5 min to separate the layers. The organic layers were collected and the aq layer was extracted with DCM (2 × 250 mL). The combined organic layer was washed with brine (2 × 200 mL) and dried (Na_2_SO_4_). The solvents were removed under reduced pressure and the residue was slurried with 10% EtOAc-hexane (300 mL) and stirred for 30 min at 50 °C. The mixture was cooled to rt and maintained at rt for 3 h to maximize precipitation. The solid residue was washed with 10% EtOAc-hexane (2 × 30 mL) and dried under vacuum at 40 °C for 2 h to afford a white-colored powder of **14** (41.67 g, 82%). R_f_ = 0.5 (silica gel, 60% EtOAc-hexane with 1% TEA). **^1^H NMR** (500 MHz, CDCl_3_) δ 9.76 (bs, 1H), 8.62 (dd, J = 4.7, 0.7 Hz, 1H), 7.98 (d, J = 7.9 Hz, 1H), 7.80 (td, J = 7.7, 1.7 Hz, 1H), 7.54 (dd, J = 8.6, 2.3 Hz, 1H), 7.49 (d, J = 2.2 Hz, 1H), 7.36 (ddd, J = 7.5, 4.8, 1.0 Hz, 1H), 7.05 (d, J = 8.7 Hz, 1H), 3.81 (q, J = 6.4 Hz, 1H), 1.75 (d, J = 6.5 Hz, 3H); **^13^C NMR** (126 MHz, CDCl_3_) δ 172.27 (s), 166.24 (s), 155.89 (s), 148.78 (s), 137.66 (s), 137.01 (s), 134.55 (s), 133.66 (s), 128.16 (s), 124.68 (s), 124.23 (s), 122.98 (s), 115.90 (s), 59.10 (s), 16.92 (s). **HRMS (ESI/IT-TOF)** *m*/*z*: [M + H] + Calcd for C_15_H_12_N_3_OBr 330.0236; found 330.0256. **%**ee >99% (HPLC).

#### 3.1.10. Synthesis of (R)-7-Bromo-3-methyl-5-(pyridin-2′-yl)-1,3-dihydro-2H-benzo[e][1,4]diazepin-2-one (13)

Compound **13** was synthesized by using the same protocol as employed for **14** on an identical scale and isolated as a white-colored powder of **13** (40.5 g, 81%). R_f_ = 0.5 (silica gel, 60% EtOAc-hexane with 1% TEA). **^1^H NMR** (500 MHz, CDCl_3_) δ 9.26 (s, 1H), 8.62 (d, *J* = 4.8 Hz, 1H), 8.03–8.00 (m, 1H), 7.81 (t, *J* = 7.7 Hz, 1H), 7.60–7.55 (m, 1H), 7.52 (s, 1H), 7.36 (dd, *J* = 8.7, 3.7 Hz, 1H), 7.05 (d, *J* = 8.6 Hz, 1H), 3.83 (q,*J* = 11.9 Hz, 1H), 1.77 (d, *J* = 6.5 Hz, 3H).^**13**^**C NMR** (126 MHz, CDCl_3_) δ 172.24 (s), 166.39 (s), 156.07 (s), 148.91 (s), 137.49 (s), 136.85 (s), 134.55 (s), 133.80 (s), 128.32 (s), 124.63 (s), 124.14 (s), 122.88 (s), 116.02 (s), 59.05 (s), 16.93 (s). **HRMS (ESI/IT-TOF)** *m*/*z*: [M + H] + Calcd for C_15_H_12_N_3_OBr 330.0236; found 330. 330.0214. %ee >99% (HPLC).

#### 3.1.11. Synthesis of Ethyl(R)-8-bromo-6-(2′-chlorophenyl)-4-methyl-4H-benzo[f]imidazo[1,5-a][1,4]diazepine-3-carboxylate (15)

The 1,4-benzodiazepine, (*R*)-7-bromo-5-(2′-chlorophenyl)-3-methyl-1,3-dihydro-2H-benzo[e][1,4]diazepin-2-one (**12**, 27 g, 74.2 mmol), was dissolved in anhydrous THF (300 mL) and a solution of potassium *t*-butoxide (10.83 g, 96.5 mmol) in anhydrous THF (60 mL) was added dropwise to that mixture at −20 °C, over a period of 20 min. The reaction mixture was then stirred for 1 h, while maintaining the temperature at −20 °C to −5 °C. Diethyl chlorophosphate (15.01 mL, 104 mmol) was added dropwise over a period of 15 min at−20 °C. The mixture was then allowed to stir for an additional 2 h. The reaction was then cooled to −30 °C and ethyl isocyanoacetate (10.5 mL, 96.5 mmol) was added dropwise to the mixture over a period of 15 min and this was followed by dropwise addition of a solution of *t*-BuOK (10.83 g, 96.5 mmol) in anhydrous THF (60 mL). The reaction was allowed to warm to rt and stirred for 8 h, at which point the reaction progress was deemed to be complete on analysis by TLC (silica gel, 60% EtOAc-hexane). A little starting material was observed on TLC. At the end of the reaction progress, the mixture was quenched by dropwise addition of aq 10% NaHCO_3_ (400 mL) and ethyl acetate (800 mL) was added with stirring. The mixture was allowed to stand for 10 min to separate the layers. The organic layer was separated, and the aqueous layer was extracted with ethyl acetate (2 × 150 mL). The combined organic layers were washed with aq 10% NaHCO_3_ (300 mL), aq 10% NaCl (2 × 300 mL), and dried (Na_2_SO_4_). The solvents were removed under reduced pressure. The black colored semi-solid, which resulted, was slurried with *tert*-butyl methyl ether (150 mL) and the mixture was stirred for 20 min at 50 °C. At that point, hexane (150 mL) was added to that mixture, and it was stirred for an additional 15 min at 50 °C. The mixture was allowed to cool to rt and held there for 5 h. The solid residue was filtered and washed with TBME-hexane (1:1, 3 × 50 mL). The residue was dried under vacuum for 4 h to obtain a light, orange-colored powder of **15** (19 g, 58%). R_f_ = 0.3 (silica gel, 60% EtOAc-hexane); **^1^H NMR** (500 MHz, CDCl_3_) δ 7.91 (s, 1H), 7.71 (d, *J* = 8.0 Hz, 1H), 7.48 (d, *J* = 8.6 Hz, 2H), 7.44–7.34 (m, 4H), 7.27 (d, *J* = 11.2 Hz, 1H), 6.68 (q, *J* = 6.8Hz, 1H), 4.41 (dd, *J* = 14.2, 7.0 Hz, 2H), 1.42 (t, *J* = 7.1 Hz, 4H), 1.33 (d, *J* = 6.7 Hz, 3H); **^13^C NMR** (126 MHz, CDCl_3_) δ 165.10 (s), 162.90 (s), 141.51 (s), 139.33 (s), 134.82 (s), 134.74 (s), 134.16 (s), 132.85 (s), 132.35 (s), 130.93 (s), 130.86 (s), 130.69 (s), 130.16 (s), 129.68 (s), 127.19 (s), 123.55 (s), 120.96 (s), 60.84 (s), 50.15 (s), 15.00 (s), 14.43 (s). **HRMS** (ESI/IT-TOF) *m*/*z*: [M + H] Calcd for C_21_H_17_N_3_O_2_ClBr 458.0265; found 458.0268. %ee > 99%

#### 3.1.12. Synthesis of Ethyl(S)-8-bromo-4-methyl-6-(pyridin-2′-yl)-4H-benzo[f]imidazo[1,5-a][1,4]diazepin-3-carboxylate (17)

A solution of *t*-BuOK (19.44 g, 173 mmol) in anhydrous THF (150 mL) was added dropwise over a period of 20 min to a stirred solution of 1,4-benzodiazepine **14** (44 g, 133 mmol) in anhydrous THF (400 mL) at −20 °C. The mixture was then stirred for 1.5 h, after which diethyl chlorophosphate (26.9 mL, 186.2 mmol) was added dropwise at −20 °C to the reaction mixture. The solution was allowed to warm to rt, and the temperature was held there for 2 h. The consumption of starting material was confirmed by TLC (silica gel, 60% EtOAc-hexane, 1% TEA). A small amount of starting material remained unreacted even after increasing the stirring time. Then the reaction was again cooled to −20 °C and ethyl isocyanoacetate (18.9 mL, 173 mmol) was added, and this was followed by a dropwise addition of a second portion of *t*-BuOK (19.44 g, 173 mmol) in THF (100 mL) solution at −20 °C. The reaction mixture was allowed to warm to rt and stirred for 8 h. The consumption of starting material was determined on analysis by TLC (silica gel, 70% EtOAc-hexane with 1% TEA). The reaction was quenched by addition of a cold 10% aq solution of NaHCO_3_ (300 mL) and extracted with EtOAc (3 × 300 mL). The combined organic layer was washed with brine (2 × 150 mL), dried (Na_2_SO_4_) and the solvent was removed under reduced pressure to afford a brown solid. Then *t*-butyl methyl ether (250 mL) was added to the brown impure solid and stirred for 30 min at 55 °C. The mixture was then cooled to rt, and stirred at rt for 3 h. The residue was filtered and washed with *t*-butyl methyl ether (2 × 40 mL). The solid residue was then slurried again with ethanol (200 mL) and stirred for 30 min at 60 °C. The mixture was cooled to rt and kept at −20 °C in the freezer for 5 h to maximize precipitation. The solid residue was filtered, washed with cold ethanol (2 × 50 mL), and dried under vacuum at 45 °C for 3 h to obtain pure imidazodiazepine **17** as an off-white powder.(34.5 g, 61.5%). R_f_ = 0.4 (70% EtOAc-hexane with 2% TEA). **^1^H NMR** (500 MHz, CDCl3) δ 8.55 (d, J = 4.6 Hz, 1H), 8.03–7.98 (m, 1H), 7.87 (s, 1H), 7.80 (td, J = 7.7, 1.6 Hz, 1H), 7.71 (dd, J = 8.6, 2.1 Hz, 1H), 7.57–7.44 (m, 2H), 7.35 (dd, J = 7.1, 5.4 Hz, 1H), 6.68 (q, J = 7.3 Hz, 1H), 4.38 (dd, J = 11.0 7.1Hz, 2H), 1.39 (t, J = 7.1 Hz, 3H), 1.27 (d, J = 7.4 Hz, 3H). **^13^C NMR** (126 MHz, CDCl_3_) δ 164.55 (s), 162.95 (s), 157.15 (s), 148.52 (s), 141.58 (s), 136.99 (s), 134.96 (s), 134.94 (s), 134.77 (s), 134.67 (s), 134.36 (s), 129.33 (s), 124.73 (s), 124.05 (s), 123.88 (s), 120.35 (s), 60.76 (s), 49.82 (s), 14.57 (s), 14.42 (s). **HRMS (ESI/IT-TOF)** *m*/*z*: [M + H]+ Calcd for C_20_H_17_ N_4_O_2_Br 425.0607; found 425.0640. %ee > 99% (Chiral pak IBN3-4.6 mm × 150 mm, 3 µm).

#### 3.1.13. Synthesis of Ethyl(R)-8-bromo-4-methyl-6-(pyridin-2′-yl)-4H-benzo[f]imidazo[1,5-a][1,4]diazepin-3-carboxylate (16)

Compound **16** was synthesized using the same protocol as compound **17**, on an identical scale, and was isolated as an off-white powder ( 35.5 g, 62.1%). R_f_ = 0.4 (70% EtOAc-hexane with 2% TEA). **^1^H NMR** (500 MHz, CDCl_3_) δ 8.58 (d, *J* = 4.7 Hz, 1H), 8.03 (d, *J* = 7.9 Hz, 1H), 7.87 (s, 1H), 7.83 (td, *J* = 7.9, 1.5 Hz, 1H), 7.74 (dd, *J* = 8.6, 2.0 Hz, 1H), 7.50 (d, *J* = 2.0 Hz, 1H), 7.46 (d, *J* = 8.6 Hz, 1H), 7.38 (dd, *J* = 6.9, 5.1 Hz, 1H), 6.72 (q, *J* = 7.3 Hz, 1H), 4.56–4.29 (m, 3H), 1.43 (t, *J* = 7.1 Hz, 3H), 1.29 (d, *J* = 7.4 Hz, 3H).

**^13^C NMR** (126 MHz, CDCl_3_) δ 164.56 (s), 163.03 (s), 157.28 (s), 148.59 (s), 141.67 (s), 136.96 (s), 135.00 (s), 134.90 (s), 134.73 (s), 129.43 (s), 124.87 (s), 124.71 (s), 124.60 (s), 124.04 (s), 123.83 (s), 120.36 (s), 60.79 (s), 49.86 (s), 14.58 (s), 14.43 (s). **HRMS (ESI/IT-TOF)** *m*/*z*: [M + H]+ Calcd for C_20_H_17_ N_4_O_2_Br 425.0607; found 425.0610. %ee >99% (Chiral pak IBN3-4.6 mm × 150 mm, 3 µm).

#### 3.1.14. Synthesis of Ethyl-(R)-6-(2′-chlorophenyl)-4-methyl-8-((triisopropylsilyl)ethynyl)-4H-benzo[f] imidazo[1,5-a][1,4]diazepine-3-carboxylate (18)

A round bottom flask was charged with tri(o-tolyl)phosphine (1.61 g, 5.3 mmol), Pd(OAc)_2_ (0.59 g, 2.65 mmol), ACN (20 mL), and the mixture was allowed to stir for 10 min under an argon atmosphere. Then, the imidazodiazepine, ethyl(*R*)-8-bromo-6-(2′-chlorophenyl)- 4-methyl -4H-benzo[f]imidazo[1,5-a][1,4]diazepine-3-carboxylate (**15**, 15.2 g, 33.1 mmol), triethylamine (13.9 mL, 9.93 mmol), (triisopropylsilyl)acetylene (8.2 mL, 36.4 mmol) and additional acetonitrile (200 mL) were added sequentially to the former reaction mixture under argon. The reaction mixture was then allowed to stir for 6 h at reflux until the consumption of starting material was confirmed by analysis by TLC (silica gel; 60% ethyl acetate-hexane). The mixture was then cooled to rt and opened to the air to convert the palladium salts to solid PdO. Silica gel (100 g) was then added to the flask, and stirred for 30 min. The contents were filtered through a pad of celite, and the residue was washed with DCM (2 × 150 mL). The solvents were removed under reduced pressure. Then DCM (200 mL) and water (200 mL) were added to the resulting black oily mass to dissolve the contents. The mixture was allowed to stir for 5 min and allowed to stand to separate the layers for 10 min. The layers were separated and the aq layer was extracted with DCM (2 × 50 mL). The combined organic layers were washed with 10% aqueous NaCl solution (2 × 50 mL) and dried (Na_2_SO_4_). The DCM was removed under reduced pressure. The residue was purified by silica gel (300 g) flash chromatography using 50% EtOAc-hexane. The appropriate fractions were pooled, and the solvents were removed under reduced pressure. The semi-solid residue, which resulted, was dried under vacuum for 4 h to afford a yellow-colored oil of **18** (16 g, 86%). R_f_ = 0.6 (silica gel, 60% EtOAc-hexane); **^1^H NMR** (500 MHz, CDCl_3_) δ 7.92 (s, 1H), 7.65 (d, *J* = 7.9 Hz, 1H), 7.52 (d, *J* = 8.3 Hz, 2H), 7.44–7.34 (m, 3H), 7.21–7.12 (m, 1H), 6.66 (q, *J* = 6.6 Hz, 1H), 4.41 (q, *J* = 8.4 Hz, 2H), 1.42 (t, *J* = 7.1 Hz, 3H), 1.32 (d, *J* = 6.7 Hz, 3H), 1.10 (s, 21H). **^13^C NMR** (126 MHz, CDCl_3_) δ 165.82 (s), 162.96 (s), 141.64 (s), 139.63 (s), 135.30 (s), 134.79 (s), 134.52 (s), 133.17 (s), 132.39 (s), 131.81 (s), 130.61 (s), 130.06 (s), 129.58 (s), 129.40 (s), 127.05 (s), 122.99 (s), 121.94 (s), 104.59 (s), 94.25 (s), 60.79 (s), 50.14 (s), 18.59 (s), 14.98 (s), 14.43 (s), 11.22 (s). **HRMS** (ESI/IT-TOF) *m*/*z*: [M + H] Calcd for C_32_H_38_N_3_O_2_SiCl 560.2494; found 560.2500.

#### 3.1.15. Synthesis of Ethyl(S)-4-methyl-6-(pyridin-2′-yl)-8-((triisopropylsilyl)ethynyl)-4Hbenzo [f]imidazo[1,5-a][1,4]diazepine-3-carboxylate (20)

A round bottom flask was charged with tri-(*o*-tolyl) phosphine (3.31 g, 10.88 mmol), Pd(OAc)_2_ (1.22 g, 5.4 mmol), ACN (30 mL), and the mixture was stirred for 10 min under an argon atmosphere to generate the Pd catalyst in situ. Then ethyl (*S*)-8-bromo-4- methyl-6-(pyridin-2′-yl)-4H-benzo[f]imidazo[1,5-a][1,4]diazepin-3-carboxylate (**17**, 29 g, 68 mmol), triethylamine (28.7 mL, 204 mmol), (triisopropylsilyl)acetylene (16.82 mL, 75 mmol) and additional acetonitrile (230 mL) were added sequentially to the previous reaction mixture under argon. The reaction mixture was allowed to stir at reflux for 4 h. At the end of the reaction period the mixture was then cooled to rt, and silica gel (30 g) was added to the flask, and the mixture was allowed to stir for 15 min. The contents were filtered through a pad of celite, and the residue was washed with DCM (3 × 300 mL). The solvents were removed under reduced pressure. After which DCM (400 mL) and water (200 mL) were added to the resulting black oily mass to dissolve the contents. The mixture was stirred for 5 min and then allowed to stand to separate the layers for 10 min. The layers were separated and the aq layer was extracted with DCM (2 × 100 mL). The combined organic layers were washed with 10% aqueous NaCl (2 × 50 mL) and dried (Na_2_SO_4_). The DCM was removed under reduced pressure, and the residue was purified by silica gel (400 g) flash chromatography using 60% EtOAc-hexane with 2% TEA. The desired fractions were collected, and the solvents were removed under reduced pressure. The oil, which resulted, was then dried under vacuum for 4 h to afford an orange-colored oil of **20** (31 g, 86%). R_f_ = 0.6 (silica gel, 70% EtOAc-hexane with 1% TEA). **^1^H NMR** (500 MHz, CDCl_3_) δ 8.57 (d, J = 3.1 Hz, 1H), 7.99 (d, J = 7.9 Hz, 1H), 7.88 (s, 1H), 7.81 (t, J = 7.6 Hz, 1H), 7.69 (dd, J = 22.9, 8.3 Hz, 1H), 7.51 (dd, J = 8.3, 0.5 Hz, 1H), 7.39 (s, 1H), 7.35 (m, 1H), 6.69 (q, J = 7.1 Hz, 1H), 4.39 (m, 2H), 1.40 (t, J = 7.1 Hz, 3H), 1.27 (d, J = 7.3 Hz, 3H), 1.09 (s, 21H). **^13^C NMR** (126 MHz, CDCl_3_) δ 165.28 (s), 162.99 (s), 157.43 (s), 148.50 (s), 141.69 (s), 136.87 (s), 135.39 (s), 135.31 (s), 135.17 (s), 135.00 (s), 129.27 (s), 127.75 (s), 124.60 (s), 124.10 (s), 122.43 (s), 122.28 (s), 104.85 (s), 93.74 (s), 60.72 (s), 49.83 (s), 18.59 (s), 14.55 (s), 14.42 (s), 11.21 (s). %ee > 99%.

#### 3.1.16. Synthesis of Ethyl(R)-4-methyl-6-(pyridin-2′-yl)-8-((triisopropylsilyl)ethynyl)-4Hbenzo [f]imidazo[1,5-a][1,4]diazepine-3-carboxylate (19)

Compound **19** was synthesized by using the same protocol as **20** on an identical scale and isolated as an orange-colored oil of **19** (30 g, 83.5%). R_f_ = 0.6 (silica gel, 70% EtOAc-hexane with 1% TEA ^**1**^**H NMR** (500 MHz, CDCl_3_) δ 8.59 (d, *J* = 4.2 Hz, 1H), 8.01 (d, *J* = 7.9 Hz, 1H), 7.88 (s, 1H), 7.83 (dd, *J* = 11.0, 4.4 Hz, 1H), 7.68 (d, *J* = 8.3 Hz, 1H), 7.51 (d, *J* = 8.4 Hz, 1H), 7.41 (s, 1H), 7.37 (dd, *J* = 6.9, 5.2 Hz, 1H), 6.70 (q, *J* = 7.3 Hz, 1H), 4.49–4.35 (m, 2H), 1.42 (t, *J* = 7.1 Hz, 3H), 1.29 (d, *J* = 7.3 Hz, 3H), 1.11 (s, 21H). **^13^C NMR** (126 MHz, CDCl3) δ 165.26 (s), 163.05 (s), 157.58 (s), 148.54 (s), 141.76 (s), 136.82 (s), 135.40 (s), 135.34 (s), 135.12 (s), 134.95 (s), 129.33 (s), 127.83 (s), 124.55 (s), 124.05 (s), 122.43 (s), 122.24 (s), 104.88 (s), 93.72 (s), 60.73 (s), 49.86 (s), 18.60 (s), 14.55 (s), 14.44 (s), 11.23 (s). HRMS (ESI/IT-TOF) *m*/*z*: [M + H] Calcd for C_25_H_27_N_4_O_2_Si 443.1898; found 443.1901.

#### 3.1.17. Synthesis of Ethyl-(R)-6-(2′-chlorophenyl)-8-ethynyl-4-methyl-4H-benzo[f]imidazo[1,5-a][1,4]diazepine-3-carboxylate (18A)

The tetrabutylammonium fluoride hydrate (1 M solution in THF, 34.27 mL, 34.27 mmol) was added to the stirred solution of TIPS protected intermediate, ethyl-(*R*)-6-(2′-chlorophenyl)- 4-methyl-8-((triisopropylsilyl)ethynyl)-4H-benzo[f]imidazo[1,5-a][1,4]diazepine-3-carboxylate **18** (16 g, 28.6 mmol) in THF (150 mL) and water(2 mL) at −10 °C. The reaction mixture was then allowed to stir for 1 h at rt until the consumption of starting material was indicated by TLC (silica gel, 60% EtOAc- hexane). The reaction solution was then quenched with water (150 mL) and ethyl acetate (250 mL) was added. The organic layer was separated, and the aqueous layer was extracted with ethyl acetate (2 × 100 mL). The combined organic layers were washed with brine (2 × 200 mL), dried (Na_2_SO_4_), and the solvent was removed under reduced pressure. The solid residue, which resulted, was purified by flash chromatography (silica gel, EtOAc/hexanes 6:4) to afford pure ethyl ester **18A** as a white powder (10.2 g, 89 %). R_f_ = 0.3 (silica gel, 70% EtOAc-hexane); **^1^H NMR** (500 MHz, CDCl_3_) δ 7.93 (s, 1H), 7.69 (d, *J* = 8.0 Hz, 1H), 7.52 (dd, *J* = 42.4, 17.0 Hz, 2H), 7.38 (dd, *J* = 11.6, 7.5 Hz, 3H), 7.26 (s, 1H), 6.67 (q, *J* = 7.0 Hz, 1H), 4.41 (m, 2H), 3.15 (s, 1H), 1.42 (t, *J* = 7.1 Hz, 3H), 1.32 (d, *J* = 6.9 Hz, 3H). **^13^C NMR** (126 MHz, CDCl_3_) δ 165.67 (s), 162.92 (s), 141.61 (s), 139.58 (s), 135.17 (s), 135.02 (s), 134.81 (s), 133.75 (s), 133.72 (s), 132.38 (s), 130.71 (s), 130.12 (s), 129.66 (s), 129.45 (s), 127.12 (s), 122.09 (s), 121.62 (s), 81.44 (s), 79.76 (s), 60.83 (s), 50.13 (s), 15.00 (s), 14.43 (s). **HRMS** (ESI/IT-TOF) *m*/*z*: [M + H] Calcd for C_23_H_18_N_3_O_2_Cl 404.1160; found 404.1126. %ee > 99%

#### 3.1.18. Synthesis of Ethyl (S)-8-ethynyl-4-methyl-6-(pyridin-2′-yl)-4H-benzo[f]imidazo[1,5-a][1,4]diazepine-3-carboxylate (20A)

A solution of tetrabutylammonium fluoride hydrate, [1 M in THF (87.7 mL, 87.7 mmoL)] was added dropwise to a stirred solution of the TIPS protected ethyl ester, ethyl (*S*)-4-methyl-6-(pyridin-2′-yl)-8-((triisopropylsilyl)ethynyl)-4Hbenzo[f]imidazo[1,5-a][1,4]diazepine-3-carboxylate (**20**, 44 g, 83.5 mmol), in THF(250 mL) and water (20 mL) at −20 °C. The reaction mixture was allowed to stir for 1 h at rt, at which point, the reaction was completed, and the consumption of starting material was confirmed by analysis by TLC (silica gel; 80% ethyl acetate-hexane with 2% TEA). Water (200 mL) was then added to quench the reaction and the mixture was then diluted with DCM (500 mL). The biphasic mixture, which resulted, was stirred for 5 min, and allowed to stand for 10 min. The organic layer was separated and the aqueous layer was then extracted with DCM (2 × 150 mL). The combined organic layers were washed with a 10% aq sodium chloride solution (2 × 100 mL) and dried (Na_2_SO_4_). The solvents were removed under reduced pressure, which resulted, in an orange-colored residue. The residue was slurried with IPA (300 mL) and allowed to stir for 30 min at 55 °C. Then water (300 mL) was added to the mixture, and it was stirred for an additional 20 min. The mixture was allowed to cool to rt and kept overnight. The solid residue was filtered, washed with IPA-water (1:1, 2 × 50 mL), and dried under vacuum at 45 °C for 3 h to afford the product as a white powder of **20A** (26.60 g, 86%). R_f_ = 0.4 (silica gel; 80% ethyl acetate-hexane with 2% TEA); **^1^H NMR** (500 MHz, CDCl3) δ 8.54 (d, J = 4.6 Hz, 1H), 7.97 (d, J = 7.9 Hz, 1H), 7.89 (s, 1H), 7.79 (td, J = 7.7, 1.6 Hz, 1H), 7.68 (dd, J = 8.3, 1.7 Hz, 1H), 7.54 (d, J = 8.4 Hz, 1H), 7.43 (d, J = 1.6 Hz, 1H), 7.34 (ddd, 1H), 6.68 (q, J = 7.3 Hz, 1H), 4.37 (dd, J = 11.1, 7.1 Hz, 2H), 3.14 (s, 1H), 2.13 (d, J = 6.5 Hz, 1H), 1.39 (t, J = 7.1 Hz, 3H), 1.25 (d, J = 7.4 Hz, 3H). 13C NMR (126 MHz, CDCl3) δ 165.16 (s), 162.96 (s), 157.40 (s), 148.55 (s), 141.66 (s), 136.93 (s), 135.82 (s), 135.09 (s), 135.03 (s), 129.32 (s), 127.85 (s), 124.63 (s), 124.06 (s), 122.44 (s), 120.98 (s), 81.63 (s), 79.52 (s), 60.74 (s), 49.81 (s), 14.57 (s), 14.41 (s). **HRMS (ESI/IT-TOF)** *m*/*z*: [M + H]+ Calcd for C_22_H_18_N_4_O_2_ 371.1505; found 371.1532.

#### 3.1.19. Synthesis of Ethyl (R)-8-ethynyl-4-methyl-6-(pyridin-2′-yl)-4H-benzo[f]imidazo[1,5-a][1,4]diazepine-3-carboxylate (19A)

Compound **19A** was synthesized by using the same protocol as **20A** on an identical scale and isolated as a white powder of **20A** (27.5 g, 89%). R_f_ = 0.4 (silica gel; 80% ethyl acetate-hexane with 2% TEA); **^1^H NMR** (500 MHz, CDCl_3_) δ 8.58 (d, *J* = 4.2 Hz, 1H), 8.00 (d, *J* = 7.9 Hz, 1H), 7.89 (s, 1H), 7.82 (td, *J* = 7.8, 1.5 Hz, 1H), 7.71 (dd, *J* = 8.3, 1.5 Hz, 1H), 7.55 (d, *J* = 8.3 Hz, 1H), 7.47 (t, *J* = 7.3 Hz, 1H), 7.37 (dd, *J* = 6.7, 5.1 Hz, 1H), 6.71 (q, *J* = 7.3 Hz, 1H), 4.41 (m, 2H), 3.16 (s, 1H), 1.42 (t, *J* = 7.1 Hz, 3H), 1.26 (d, *J* = 21.4 Hz, 3H). **^13^C NMR** (126 MHz, CDCl_3_) δ 165.17 (s), 163.04 (s), 157.56 (s), 148.63 (s), 141.76 (s), 136.89 (s), 135.87 (s), 135.06 (s), 134.98 (s), 134.44 (s), 129.41 (s), 127.96 (s), 124.60 (s), 124.04 (s), 122.41 (s), 120.98 (s), 81.70 (s), 79.41 (s), 60.77 (s), 49.85 (s), 14.58 (s), 14.43 (s). **HRMS (ESI/IT-TOF)** *m*/*z*: [M + H]+ Calcd for C_22_H_18_N_4_O_2_ 371.1505; found 371.1502.

#### 3.1.20. Synthesis of (R)-6-(2′-Chlorophenyl)-8-ethynyl-N,4-dimethyl-4H-benzo[f]imidazo[1,5-a][1,4]diazepine- 3-carboxamide (21)

The ethyl ester, ethyl-(*R*)-6-(2′-chlorophenyl)-8-ethynyl-4-methyl-4H-benzo[f]imidazo[1,5-a][1,4]diazepine-3-carboxylate (**18A**, 1.0 g, 2.5 mmol), was charged into a sealed vessel fitted with a septum at −30 °C and then methyl amine (20 mL, 33% wt solution in EtOH) was added. The vessel was sealed with a screwcap and stirred at 50 °C for 18 h. The solution was then cooled to rt, and the methyl amine and ethanol were removed under reduced pressure. The residue, which resulted, was purified by flash column chromatography (silica gel, 80% EtOAc-hexane) to afford the pure methyl amide **21** as an off- white powder (0.54 g, 56 %). R_f_ = 0.2 (silica gel, 90% EtOAc-hexane); **^1^H NMR** (500 MHz, CDCl_3_) δ 7.82 (s, 1H), 7.67 (d, *J* = 7.3 Hz, 1H), 7.53 (d, *J* = 8.3 Hz, 2H), 7.41–7.33 (m, 3H), 7.26 (s, 1H), 7.17 (s, 1H), 6.89 (q, J= 7.2 Hz, 1H), 3.14 (s, 1H), 2.98 (s, 3H), 1.32 (d, *J* = 4.3 Hz, 3H). **^13^C NMR** (126 MHz, CDCl_3_) δ 165.19 (s), 163.29 (s), 139.81 (s), 138.75 (s), 135.26 (s), 134.95 (s), 133.76 (s), 133.34 (s), 132.35 (s), 131.88 (s), 130.88 (s), 130.59 (s), 130.02 (s), 129.57 (s), 129.20 (s), 128.19 (s), 127.08 (s), 121.90 (s), 121.34 (s), 81.56 (s), 79.52 (s), 49.94 (s), 25.62 (s), 15.21 (s). **HRMS** (ESI/IT-TOF) *m*/*z*: [M + H] Calcd for C_22_H_17_N_4_OCl 389.1164; found 389.1131. %ee > 99%

#### 3.1.21. Synthesis of (S)-8-Ethynyl-N,4-dimethyl-6-(pyridin-2′-yl)-4H-benzo[f]imidazo[1,5-a][1,4]diazepine-3-carboxamide (23)

The starting ester ethyl, (*S*)-8-ethynyl-4-methyl-6-(pyridin-2′-yl)-4H-benzo[f]imidazo[1,5-a][1,4]diazepine-3-carboxylate (**20A**, 1 g, 2.69 mmol), was charged into a sealed vessel fitted with a septum at −30 °C, and then methyl amine (20 mL, 33% wt solution in EtOH) was added. The vessel was sealed with a screwcap and stirred at 60 °C for 12 h. The solution was then cooled to rt, and the methyl amine and ethanol were removed under reduced pressure. The residue, which resulted, was purified by flash column chromatography (silica gel, EtOAc) to afford the pure methyl amide **23** as an off- white colored powder (0.63 g, 65 %): ^**1**^**H NMR** (500 MHz, CDCl3) δ 8.56 (d, J = 4.6 Hz, 1H), 8.06 (d, J = 7.9 Hz, 1H), 7.82 (t, J = 7.7 Hz, 1H), 7.76 (s, 1H), 7.70 (d, J = 8.3 Hz, 1H), 7.52 (d, J = 8.3 Hz, 1H), 7.47 (s, 1H), 7.35 (dd, J = 6.7, 5.5 Hz, 1H), 7.17 (s, 1H), 6.90 (q, J = 7.2 Hz, 1H), 3.15 (s, 1H), 2.97 (s, 3H), 1.28 (d, J = 7.2 Hz, 3H). **^13^C NMR** (126 MHz, CDCl3) δ 164.69 (s), 163.40 (s), 157.72 (s), 148.38 (s), 138.74 (s), 136.83 (s), 136.08 (s), 135.95 (s), 134.84 (s), 133.63 (s), 131.57 (s), 128.01 (s), 124.45 (s), 124.07 (s), 122.25 (s), 120.70 (s), 81.84 (s), 79.18 (s), 49.63 (s), 25.57 (s), 14.74 (s). **HRMS** (ESI/IT-TOF) *m*/*z*: [M + H] + Calcd for C_21_ H_17_N_5_O 356.1506; found 356.1474; %ee => 99% (HPLC).

#### 3.1.22. Synthesis of (R)-8-Ethynyl-N,4-dimethyl-6-(pyridin-2′-yl)-4H-benzo[f]imidazo[1,5-a][1,4]diazepine-3-carboxamide (22)

Compound **22** was synthesized by using the same protocol as **23**. **^1^H NMR** (500 MHz, CDCl_3_) δ 8.56 (dd, *J* = 4.7, 0.6 Hz, 1H), 8.06 (d, *J* = 7.9 Hz, 1H), 7.82 (td, *J* = 7.8, 1.7 Hz, 1H), 7.76 (s, 1H), 7.69 (dd, *J* = 8.3, 1.4 Hz, 1H), 7.52 (d, *J* = 8.3 Hz, 1H), 7.47 (d, *J* = 1.1 Hz, 1H), 7.35 (ddd, *J* = 7.4, 4.8, 0.9 Hz, 1H), 7.18 (d, *J* = 4.3 Hz, 1H), 6.90 (q, *J* = 7.3 Hz, 1H), 3.15 (s, 1H), 2.96 (s, 3H), 1.28 (d, *J* = 7.1, 4.2 Hz, 3H). **^13^C NMR** (126 MHz, CDCl_3_) δ 164.72 (s), 163.42 (s), 157.73 (s), 148.39 (s), 138.74 (s), 136.84 (s), 136.08 (s), 135.96 (s), 134.85 (s), 133.65 (s), 131.57 (s), 128.02 (s), 124.47 (s), 124.08 (s), 123.09 (s), 122.26 (s), 81.84 (s), 79.20 (s), 49.64 (s), 25.57 (s), 14.74 (s). **HRMS** (ESI/IT-TOF) *m*/*z*: [M + H] + Calcd for C_21_ H_17_N_5_O 356.1506; found 356.1504; %ee => 99% (HPLC).

#### 3.1.23. Synthesis of (R)-6-(2′-Chlorophenyl)-8-ethynyl-4-methyl-4H-benzo[f]imidazo[1,5-a][1,4]diazepine-3-carboxylic acid (25)

The ethyl ester, ethyl-(*R*)-6-(2′-chlorophenyl)-8-ethynyl-4-methyl-4H-benzo[f]imidazo[1,5-a][1,4]diazepine-3-carboxylate (**18A**, 5.0 g, 12.4 mmol), was dissolved in THF (500 mL) and a solution of lithium hydroxide (1.18 g, 49.6 mmol) was added dropwise to the solution at rt. The reaction mixture was then heated to 55 °C for 0.5 h. The consumption of starting material was confirmed by TLC (silica gel, 100% EtOAc). Water (20 mL) was then added to dilute the mixture, followed by addition of ethyl acetate (3.34 mL, 62 mmol). Then THF was removed under reduced pressure. The mixture was then kept at rt for 2 h. The solid residue, which resulted, was filtered, washed with water (4 × 10 mL), and dried under vacuum. The solid residue was then slurried in 100% EtOAc (20 mL) and stirred for 10 min at 55 ^o^C. The mixture was cooled to rt and maintained at that temperature for 2 h. The residue was filtered, washed with ethyl acetate (2 × 10 mL), and dried under vacuum for 2 h to provide pure acid **25** as an off-white powder (4.18 g, 90%). **^1^H NMR** (300 MHz, d^6^-DMSO) δ 8.38 (s, 1H), 7.87 (dd, *J* = 12.2, 8.2 Hz, 2H), 7.66–7.35 (m, 4H), 7.07 (s, 1H), 6.52 (q, J = 7.2 Hz, 1H), 4.35 (s, 1H), 1.19 (d, *J* = 2.2 Hz, 3H). **^13^C NMR** (75 MHz, DMSO-d^6^) δ 165.21 (s), 164.96 (s), 139.93 (s), 136.39 (s), 135.47 (s), 135.33 (s), 133.05 (s), 132.89 (s), 131.57 (s), 131.46 (s), 130.38 (s), 130.09 (s), 129.12 (s), 127.91 (s), 124.96 (s), 123.89 (s), 120.95 (s), 83.42 (s), 82.02 (s), 49.86 (s), 15.24 (s).). **HRMS** (ESI/IT-TOF) *m*/*z*: [M + H] Calcd for C_21_H_14_N_3_O_2_Cl 376.0847; found 376.0828.

#### 3.1.24. Synthesis of (S)-8-Ethynyl-4-methyl-6-(pyridin-2′-yl)-4H-benzo[f]imidazo[1,5-a][1,4]diazepine- 3-carboxylic acid (27)

The ethyl ester, ethyl (*S*)-8-ethynyl-4-methyl-6-(pyridin-2′-yl)-4H-benzo[f]imidazo[1,5-a][1,4]diazepine-3-carboxylate(**20A**, 3 g, 8.1 mmol), was dissolved in ethanol (30 mL) and a solution of lithium hydroxide (0.766 g, 32 mmol) in water (5 mL) was added dropwise to that mixture. The mixture was then stirred at 50 °C for 1 h at which point the reaction was deemed to be completed on analysis by TLC (silica gel, 100% EtOAc with 2% TEA). The mixture was then cooled to rt, and water (20 mL) was added to dilute the mixture. Then glacial acetic acid (2.5 mL) was added to adjust the pH to 5 (pH paper). Then ethanol was removed under reduced pressure and a white precipitation formed. *Caution: Addition of too much water or acetic acid needs to be avoided, otherwise the acid will go back into the water solution, and it will be difficult to make it precipitate*. The solid residue was filtered, washed with water (3 × 10 mL), and dried under vacuum. The residue was then suspended in 100% EtOAc (10 mL) and stirred for 10 min at 45 °C. The mixture was cooled to rt and held there for 1 h. The solid was filtered, washed (EtOAc, 2 × 3 mL), and dried under vacuum at 40 °C for 1 h to afford a white powder of **27** (2.49 g, 90%). **^1^H NMR** (500 MHz, DMSO-d^6^) δ 8.49 (d, J = 4.2 Hz, 1H), 8.41 (s, 1H), 8.02 (dd, J = 28.9, 7.7 Hz, 1H), 7.92 (ddd, J = 8.4, 6.8, 2.8 Hz, 2H), 7.80 (d, J = 8.4 Hz, 1H), 7.46 (dd, J = 6.7, 5.3 Hz, 1H), 7.34 (s, 1H), 6.54 (q, J = 7.2 Hz, 1H), 4.29 (s, 1H), 1.16 (d, J = 7.3 Hz, 3H). **^13^C NMR** (126 MHz, DMSO-d^6^) δ 165.43 (s), 164.65 (s), 157.59 (s), 148.56 (s), 141.20 (s), 137.54 (s), 136.73 (s), 135.79 (s), 135.46 (s), 135.13 (s), 129.29 (s), 128.08 (s), 125.28 (s), 124.06 (s), 123.80 (s), 120.32 (s), 82.97 (s), 82.31 (s), 49.58 (s), 14.72 (s). **HRMS (ESI/IT-TOF)** *m*/*z*: [M + H]+ Calcd for C_22_H_18_N_4_O_2_ 343.1189; found 343.1208.

#### 3.1.25. Synthesis of (R)-8-Ethynyl-4-methyl-6-(pyridin-2′-yl)-4H-benzo[f]imidazo[1,5-a][1,4]diazepine- 3-carboxylic acid (26)

Compound **26** was synthesized by using the same protocol as **27**. **^1^H NMR** (300 MHz, DMSO-d^6^) δ 8.51 (d, *J* = 4.5 Hz, 1H), 8.38 (s, 1H), 8.00 (d, *J* = 7.4 Hz, 1H), 7.96 (dd, *J* = 7.3, 1.7 Hz, 1H), 7.91 (d, *J* = 3.1 Hz, 1H), 7.88 (s, 1H), 7.80 (d, *J* = 8.2 Hz, 1H), 7.52–7.46 (m, 1H), 7.34 (s, 1H), 6.52 (q, *J* = 7.2 Hz, 1H), 4.34 (s, 1H), 1.16 (d, *J* = 7.2 Hz, 3H). **^13^C NMR** (126 MHz, DMSO-d^6^) δ 165.43(s), 164.72 (s), 157.59(s), 148.55 (s), 141.20 (s), 137.54 (s), 136.75 (s), 135.8 (s), 135.45 (s), 134.94 (s), 129.29 (s), 128.08 (s), 125.34 (s), 124.06 (s), 123.80 (s), 120.32 (s), 82.96 (s), 82.08 (s), 49.58 (s), 14.63 (s). **HRMS (ESI/IT-TOF)** *m*/*z*: [M + H]+ Calcd for C_22_H_18_N_4_O_2_ 343.1189; found 343.1211.

#### 3.1.26. (R)-8-Chloro-6-(2-fluorophenyl)-4-methyl-4H-benzo[f]imidazo[1,5-a][1,4]diazepine-3-carboxylic acid(28)

Ethyl (*R*)-8-chloro-6-(2-fluorophenyl)-4-methyl-4H-benzo[f]imidazo[1,5-a][1,4]diazepine-3-carboxylate (**24,** 6.0 g, 15 mmol) was dissolved in 500 mL of THF, followed by the dropwise addition of a solution of (14.3 g, 60 mmol) of lithium hydroxide at room temperature. The reaction mixture was heated to 55 °C for 1 h, and the consumption of starting material was confirmed by TLC analysis using silica gel and 100% EtOAc. Next, 20 mL of water was added to dilute the mixture, and 4.5 mL (75 mmol) of ethyl acetate was added. The THF solvent was removed under reduced pressure, and the mixture was kept at room temperature for 2 h. The resulting solid residue was filtered, washed with 4 × 10 mL of water, and dried under vacuum. The solid residue was then slurried in 20 mL of 100% EtOAc and stirred at 55 °C for 10 min, followed by cooling to room temperature and maintaining for 2 h. The residue was filtered, washed with 2 × 10 mL of ethyl acetate, and dried under vacuum at 45 °C for 4 h to pure acid **28** as an off-white powder (5.1 g, 91.7%). **^1^H NMR** (500 MHz, DMSO-d^6^) δ 12.72 (bs, 1H), 8.43 (s, 1H), 7.97 (d, *J* = 8.7 Hz, 1H), 7.83 (d, *J* = 8.3 Hz, 1H), 7.56 (dt, *J* = 12.0, 6.4 Hz, 2H), 7.33 (t, *J* = 7.5 Hz, 1H), 7.27–7.18 (m, 1H), 6.52 (q, *J* = 6.5 Hz, 1H), 1.17 (d, *J* = 7.2 Hz, 3H). **^13^C NMR** (126 MHz, DMSO-d^6^) δ 164.62 (s), 162.46 (s), 159.86 (d, ^1^*J_C-F_* = 247.5 Hz), 140.97 (s), 136.76 (s), 133.61 (s), 132.74 (d, ^4^*J_C-F_* = 8.8 Hz), 132.60 (s), 132.11 (s), 131.92 (s), 130.67 (s), 129.53 (s), 129.35 (s), 128.68 (d, ^3^*J_C-F_* = 12.8 Hz), 125.49 (s), 125.18 (d, ^4^*J_C-F_* = 2.4 Hz), 116.43 (d, ^2^*J_C-F_* = 21.2 Hz), 49.84 (s), 15.02 (s).

#### 3.1.27. Synthesis of (R)-6-(2′-Chlorophenyl)-8-ethynyl-N,N,4-trimethyl-4H-benzo[f]imidazo[1,5-a][1,4]diazepine-3-carboxamide (29)

A mixture of the carboxylic acid, (*R*)-6-(2′-chlorophenyl)-4-methyl-8-phenyl-4H-benzo [f]imidazo[1,5-a][1,4]diazepine-3-carboxylic acid, (**25**, 600 mg, 1.59 mmol), thionyl chloride (1.15 mL, 15.9 mmol), a catalytic amount of anhydrous N,N-dimethyl formamide (0.1 mL, 0.159 mmol) and dry DCM (10 mL) were charged into an oven dried round bottom flask under argon and the suspension, which formed, was allowed to reflux at 50 °C for 1 h under argon. The organic solvent and excess thionyl chloride were removed under reduced pressure on a rotary evaporator and a flash evaporation of added solvents was repeated five times with anhydrous DCM (5 × 10 mL). The yellow residue, which resulted, was dissolved in anhydrous DCM (20 mL) and was cooled to 0 °C for 10 min under argon. This was followed by the addition of the nucleophile, N-ethyl-N-methylamine (1.37 mL, 15.9 mmol). The mixture was then allowed to warm to rt and stirred for an hour. After the completion of the reaction as indicated by TLC (silica gel, 100% EtOAc), the mixture was diluted with ice cold water (15 mL) and extracted with DCM (3 x 20 mL). The combined organic layers were washed with brine (20 mL), dried (Na_2_SO_4_), and the residue was purified by silica gel flash chromatography (EtOAc and 1% trimethylamine) to provide a pale, yellow-colored solid of **29** (0.5 g, 80%). **R_f_** = 0.2 (silica gel, 80% EtOAc-hexane); **^1^H NMR** (300 MHz, CDCl_3_) δ 7.89 (d, *J* = 5.0 Hz, 1H), 7.68 (d, *J* = 8.2 Hz, 1H), 7.51 (t, *J* = 7.3 Hz, 2H), 7.40–7.31 (m, 3H), 7.28 (s, 1H), 4.36 (q, J = 6.8 Hz, 1H), 3.75 (dd, *J* = 10.6, 7.3 Hz, 1H), 3.38 (dd, *J* = 11.6, 6.2 Hz, 1H), 3.14 (s, 1H), 3.08 (s, 1H), 2.93 (s, 1H), 1.86 (d, *J* = 4.8 Hz, 3H), 1.22 (t, *J* = 7.1 Hz, 2H). **^13^C NMR** (75 MHz, CDCl_3_) δ 165.27 (s), 165.12 (s), 138.84 (s), 135.32 (s), 135.24 (s), 133.50 (s), 132.44 (s), 132.38 (s), 130.76 (s), 130.68 (s), 130.62 (s), 130.05 (s), 130.01 (s), 128.95 (s), 127.14 (s), 122.64 (s), 121.38 (s), 81.56 (s), 79.52 (s), 52.12 (s), 45.99 (s), 42.02 (s), 36.32 (s), 18.48 (s), 13.63 (s). **HRMS (ESI/IT-TOF)** *m*/*z*: [M + H] Calcd for C_24_H_21_N_4_OCl 417.1476; found 417.1479. %ee > 99%.

#### 3.1.28. Synthesis of (S)-8-Ethynyl-N,N,4-trimethyl-6-(pyridin-2′-yl)-4H-benzo[f]imidazo[1,5-a][1,4]diazepine-3-carboxamide (31)

Thionyl chloride (3.17 mL, 43 mmol) was added dropwise to the stirred solution of (*S*)-8-ethynyl-4-methyl-6-(pyridin-2′-yl)-4H-benzo[f]imidazo[1,5-a][1,4]diazepine-3-carboxylic acid (**27**, 1.5 g, 4.3 mmol), anhydrous *N*,*N*-dimethylformamide (0.1 mL, 0.43 mmol), anhydrous DCM (30 mL) and the mixture was maintained at 35 to 40 °C under an atmosphere of argon. The mixture, which resulted, was stirred for 1 h at reflux temperature. Evolution of SO_2_ (g) and HCl (g) was observed during the addition of thionyl chloride. The consumption of the starting material was confirmed by TLC (silica gel, 100% EtOAc with 2% TEA). To perform TLC, a test tube was filled with 1 mL of methanol and 1 mL of triethylamine. Then, 0.5 mL of the reaction mixture was added to the test tube.The methyl ester, which resulted was analyzed by TLC.. The reaction mixture was cooled at the end of the reaction and the solvents were removed under reduced pressure. The residual thionyl chloride was removed by adding and flash evaporating anhydrous DCM (5 × 10 mL) under vacuum. A yellow-colored residue was obtained. The solid residue was dissolved in anhydrous DCM (20 mL) and the mixture was cooled to 0 °C. A solution of dimethylamine (2 M in THF, 12.9 mL, 25.83 mmol) was added dropwise to the reaction mixture at 0 °C. The mixture was allowed to warm to rt and allowed to stir for 1 h until the consumption of starting material was finished (TLC). The reaction mixture was diluted with aq 10% NaHCO_3_ (50 mL) and additional DCM (50 mL) was added. The biphasic mixture, which resulted, was allowed to stand 3 min to separate the layers. The organic layer was separated and the aq layer was extracted with DCM (2 × 20 mL). The combined organic layers were washed with aq 10% NaHCO_3_ solution (1 × 25 mL), aqueous 10% NaCl solution (2 × 30 mL) and dried (Na_2_SO_4_). The solvents were removed under reduced pressure and the residue was purified by flash chromatography (silica gel, 100% EtOAC with 2% MeOH and 2% TEA). The desired fractions were combined, and the solvents were evaporated under reduced pressure. The solid was dried under vacuum at 40 °C for 1 h to afford a light, yellow-colored powder of **31** (1.08 g, 67.2%). **^1^H NMR** (500 MHz, MeOD) δ 8.52 (d, *J* = 4.6 Hz, 1H), 8.32 (s, 1H), 8.04 (d, *J* = 7.8 Hz, 1H), 7.98 (t, *J* = 7.3 Hz, 1H), 7.82 (s, 2H), 7.54–7.50 (m, 1H), 7.42 (s, 1H), 4.45 (q, *J* = 6.7 Hz, 1H), 3.68 (s, 1H), 3.15 (s, 3H), 2.97 (s, 3H), 1.86 (d, *J* = 6.8 Hz, 3H)). **^13^C NMR** (126 MHz, MeOD) δ 166.73 (s), 165.44 (s), 156.39 (s), 148.17 (s), 137.42 (s), 135.48 (s), 135.22 (s), 135.15 (s), 135.03 (s), 133.95 (s), 130.83 (s), 127.28 (s), 124.98 (s), 124.11 (s), 123.32 (s), 121.34 (s), 81.08 (s), 79.72 (s), 51.89 (s), 37.96 (s), 33.89 (s), 16.88 (s). **HRMS (ESI/IT-TOF)** *m*/*z*: [M + H]^+^ Calcd for C_22_H_19_N_5_O 370.1662; found 370.1629. %ee > 99%.

#### 3.1.29. Synthesis of (S)-8-Ethynyl-N,N,4-trimethyl-6-(pyridin-2′-yl)-4H-benzo[f]imidazo[1,5-a][1,4]diazepine-3-carboxamide (30)

Compound **30** was synthesized by using the same protocol as employed for the synthesis of **31**. ^1^H NMR (500 MHz, MeOD) δ 8.52 (d, *J* = 4.5 Hz, 1H), 8.32 (s, 1H), 8.04 (d, *J* = 7.8 Hz, 1H), 7.98 (t, *J* = 7.4 Hz, 1H), 7.82 (s, 2H), 7.57–7.50 (m, 1H), 7.42 (s, 1H), 4.45 (q, *J* = 6.6 Hz, 1H), 3.68 (s, 1H), 3.15 (s, 3H), 2.97 (s, 3H), 1.86 (d, *J* = 6.7 Hz, 3H). **^13^C NMR** (126 MHz, MeOD) δ 166.73 (s), 165.44 (s), 156.39 (s), 148.17 (s), 137.42 (s), 135.48 (s), 135.22 (s), 135.15 (s), 135.03 (s), 133.95 (s), 130.84 (s), 127.28 (s), 124.98 (s), 124.11 (s), 123.32 (s), 121.34 (s), 81.08 (s), 79.73 (s), 51.89 (s), 37.96 (s), 33.89 (s), 16.88 (s). **HRMS (ESI/IT-TOF)** *m*/*z*: [M + H]^+^ Calcd for C_22_H_19_N_5_O 370.1662; found 370.1624. %ee > 99%.

#### 3.1.30. Synthesis of (R)-8-Chloro-6-(2′-fluorophenyl)-N,N,4-trimethyl-4H-benzo[f]imidazo[1,5-a][1,4]diazepine-3-carboxamide (32)

The carboxylic acid, (*R*)-8-chloro-6-(2′-fluorophenyl)-4-methyl-4H-benzo[f]imidazo[1,5-a][1,4]diazepine-3-carboxylic acid **28** (1.2 g, 3.24 mmol), anhydrous N,N-dimethyl formamide (0.1 mL), and DCM (10 mL) were charged into a round bottom flask and the mixture was heated to 35–40 °C under an argon atmosphere. Then thionyl chloride (2.35 mL, 32.4 mmol) was added dropwise to the mixture. The reaction mixture was allowed to stir for 2 h at 35–40 °C. The consumption of starting material was confirmed by analysis by TLC (silica gel, 60% EtOAc-hexane; sample preparation- 0.2 mL methanol, 0.2 mL TEA and 0.1 mL sample).The LC was for the methyl ester which was resulted from the reaction of acid chloride and methanol. The excess thionyl chloride was removed under reduced pressure by exchanging with DCM (5 × 10 mL), and by flash evaporation (5 times). The solid residue, which resulted, was dissolved in anhydrous DCM (20 mL) and a solution of N,N-dimethylamine (2 M in THF, 9.75 mL, 19.4 mmol) was added dropwise at 0 °C. The mixture was allowed to warm to rt and allowed to stir for 2 h. The reaction mixture was diluted with water (50 mL) and DCM (30 mL). The layers were stirred and then separated. The organic layer was collected, and the aqueous layer was extracted with DCM (2 × 10 mL). The combined organic layers were washed with brine (2 × 20 mL) and dried (Na_2_SO_4_). The residue was purified by flash chromatography (silica gel, EtOAc with 1% MeOH) to afford the pure dimethyl amide **32** as an off-white powder (0.96 g, 75%). R_f_ = 0.2 (silica gel, 80% EtOAc-hexane) **^1^H NMR** (500 MHz, CDCl_3_) δ 7.91 (s, 1H), 7.59 (dt, *J* = 29.4, 13.1 Hz, 2H), 7.49 (d, *J* = 8.4 Hz, 1H), 7.44 (dd, *J* = 12.4, 6.5 Hz, 1H), 7.25 (dd, *J* = 16.0, 8.5 Hz, 2H), 7.02 (t, *J* = 9.2 Hz, 1H), 4.32 (m, 1H), 3.11 (s, 3H), 2.99 (s, 3H), 1.92 (d, *J* = 6.2 Hz, 3H). **^13^C NMR** (126 MHz, CDCl_3_) δ 166.12 (s), 162.16 (s), 160.21 (d, *^1^J_C-F_* = 250.3 Hz), 139.13 (s), 134.71 (s), 133.82 (s), 133.28 (s), 133.16 (s), 132.91 (s), 132.65 (s), 132.23 (d, *^4^J_C-F_* = 8.3 Hz), 132.10 (s), 131.81 (s), 131.30 (d, *^4^J_C-F_* = 2.1 Hz), 130.48 (s), 130.07 (s), 129.84 (s), 127.43 (d, *^3^J_C-F_* = 12.4 Hz), 124.58 (d, *^3^J_C-F_* = 2.5 Hz), 124.15 (s), 116.20 (d, *^2^J_C-F_* = 21.3 Hz), 52.24 (s), 39.09 (s), 35.04 (s), 18.43 (s). **HRMS (ESI/IT-TOF)** *m*/*z*: [M + H] Calcd for C_21_H_18_N_4_OFCl 397.1225; found 397.1259. %ee > 97%

#### 3.1.31. Synthesis of (R)-8-Ethynyl-6-(2′-fluorophenyl)-N,N,4-trimethyl-4H- benzo[f]imidazo[1,5-a][1,4]diazepine-3-carbothioamide (33)

The amide, (*R*)-8-ethynyl-6-(2′-fluorophenyl)-N, N, 4-trimethyl-4H-benzo[f]imidazo[1,5-a][1,4]diazepine-3-carboxamide was suspended in anhydrous THF (13 mL) in a sealed tube and Lawesson’s reagent (6.2 g, 15.5 mmol) was added into it. The tube was tightly sealed, and the mixture was heated to 80 °C for 36 h. The reaction mixture was cooled down to rt, and the solvent was removed under reduced pressure. The dark colored residue, which resulted, was dissolved in DCM, and loaded on a column (silica gel). Then 50% EtOAc-heaxne was passed through the column to remove the first milky fractions of the byproduct of LR reagent. Then the desired thioamide fractions were collected from the elution of 100% EtOAc. The solvents were removed under reduced pressure. The solid residue, which resulted, was dried under vacuum at 40 °C to afford **33** as a yellow-colored powder (670 mg, 67%). R_f_ = 0.4 (silica gel, 90% EtOAc-hexane); **^1^H NMR** (500 MHz, CDCl_3_) δ 7.92 (d, *J* = 56.8 Hz, 1H), 7.71 (d, *J* = 6.7 Hz, 1H), 7.63–7.50 (m, 2H), 7.45 (dd, *J* = 12.9, 6.3 Hz, 1H), 7.41 (s, 1H), 7.26 (t, *J* = 7.4 Hz, 1H), 7.04 (t, *J* = 9.1 Hz, 1H), 4.36–4.23 (m, 1H), 3.85 (s, 1H), 3.60 (s, 2H), 3.39 (s, 1H), 3.19 (s, 1H), 3.15 (s, 1H), 1.92 (d, *J* = 3.6 Hz, 1H), 1.32 (d, *J* = 1.4 Hz, 1H). **^13^C NMR** (126 MHz, CDCl_3_) δ 163.25 (s), 162.69 (s), 160.15 (d, *^1^J_C-F_* = 251.3 Hz), 137.13 (s), 135.44 (s), 135.25 (s), 134.42 (s), 133.73 (s), 132.67 (s), 132.05 (d, *^3^J_C-F_* = 7.2 Hz), 131.27 (s), 129.21 (s), 127.64 (d, *^3^J_C-F_* = 10.5 Hz), 124.50 (d, *^4^J_C-F_* = 2.9 Hz), 122.97 (s), 116.17 (d, *^2^J_C-F_* = 21.4 Hz), 81.52 (s), 79.66 (s), 55.51 (s), 52.23 (s), 44.07 (s), 42.60 (s), 18.37 (s). **HRMS** (ESI/IT-TOF) *m*/*z*: [M + H] Calcd for C_23_H_20_FN_4_S 403.1387; found 403.1385.

#### 3.1.32. Synthesis of (R)-3-Ethyl-5-(6-(2′-fluorophenyl)-4-methyl-8-phenyl-4H-benzo[f]imidazo[1,5-a][1,4]diazepin-3-yl)-1,2,4-oxadiazole (35)

The ethyl ester, ethyl(*R*)-6-(2′-fluorophenyl)-4-methyl-8-phenyl-4H-benzo[f]imidazo[1,5-a][1,4]diazepine-3-carboxylate (**3**, 1.2 g, 2.73 mmol) was dissolved in dry THF (20 mL) under an argon atmosphere. In a separate flask isopropyl oxime, N-hydroxypropionoimidamide (0.96 g, 10.92 mmol) was treated with sodium hydride (60% dispersion in mineral oil, 0.072 g, 3.0 mmol) for an hour with molecular sieves, 3 Å, after which the ethyl ester solution was added dropwise to the other flask and the reaction mixture, which resulted, was allowed to stir at rt for 2 h until the starting material was consumed (TLC, silica gel, EtOAc: hexane = 50:50). The reaction mixture was quenched with a saturated aq NaHCO_3_ solution (10 mL), diluted with water (50 mL), and extracted with EtOAc (3 x 50 mL). The combined organic layers were washed (aq 10% NaCl solution, 2 × 30 mL), dried (Na_2_SO_4_) and the solvents were removed under reduced pressure. The solid residue was purified by flash column chromatography (silica gel, EtOAc: hexane = 50:50) to afford the pure, white oxadiazole, **35** (960 mg, 86%). **^1^H NMR** (300 MHz, CDCl_3_) δ 8.12 (s, 1H), 7.85 (d, *J* = 8.4 Hz, 1H), 7.72 (d, *J* = 8.3 Hz, 1H), 7.65 (t, *J* = 7.1 Hz, 1H), 7.54–739 (m, 7H), 7.24 (d, *J* = 7.5 Hz, 1H), 7.04 (t, *J* = 9.2 Hz, 1H), 6.76 (q, *J* = 7.1 Hz, 1H), 2.84 (q, *J* = 7.6 Hz, 2H), 1.42 (d, *J* = 2.2 Hz, 1H), 1.40 (t, *J* = 6.5 Hz, 3H); **^13^C NMR** (126 MHz, CDCl_3_) δ 171.88 (s), 170.81 (s), 160.17 (d, ^1^*J_C-F_* = 251.3 Hz), 140.75 (s), 139.16 (s), 138.57 (s), 136.37 (s), 133.42 (s), 132.19 (d, ^4^*J_C-F_* = 1.8 Hz), 131.37 (d, ^3^*J_C-F_* = 9.5 Hz), 130.80 (s), 129.67 (d, ^2^*J_C-F_* = 16.9 Hz), 129.20 (s), 129.09 (s), 128.49 (s), 128.40 (s), 127.17 (s), 127.08 (s), 124.87 (s), 124.50 (d, ^3^*J_C-F_* = 3.1 Hz), 122.51 (s), 116.20 (d, ^2^*J_C-F_* = 21.5 Hz), 50.18 (s), 19.78 (s), 14.99 (s), 11.56 (s). **HRMS (ESI/IT-TOF)** *m*/*z*: [M + H] Calcd for C_23_H_20_FN_4_S 464.1881; found 464.1885.

#### 3.1.33. Synthesis of (R)-5-(8-Chloro-6-(2′-fluorophenyl)-4-methyl-4H-benzo[f]imidazo[1,5-a][1,4]diazepin -3-yl)-3-ethyl-1,2,4-oxadiazole (36)

The oxadiazole **36** was synthesized by following the same synthetic protocol for the preparation of **35.** The *N*-hydroxypropionoimidamide (1.78 g, 10 mmol) was treated with sodium hydride (60% dispersion in mineral oil, 0.13 g, 2.7 mmol) for an hour with molecular sieves, 3 Å. This solution was then added to the stirred solution of ethyl ester, ethyl (*R*)-8- chloro-6-(2′-fluorophenyl)-4-methyl-4H-benzo[f]imidazo[1,5-a][1,4]diazepine-3-carboxylate (**24**, 1 g, 2.5 mmol) in dry THF (20 mL). After stirring 2 h, the reaction was quenched with aq NaHCO_3_. Ethyl acetate (60 mL) was added to the mixture and the mixture was stirred and then allowed to stand to separate the layers. The organic layer was collected, and the aqueous layer was extracted with ethyl acetate (2 × 15 mL). The combined organic layers were washed with brine (2 × 30 mL) and dried (Na_2_SO_4_). Two separate spots were observed on TLC (silica gel, 50% EtOAc-hexane). The spots were separated by column chromatography (silica gel, 20% EtOAc-hexane to 60% EtOAc-hexane). The desired fractions of oxadiazole **37** were collected and the solvents were removed under reduced pressure. The residue was dried under vacuum to obtain pure **37** as a white powder (1.1 g, 52%). The other fraction was the olefinic migrated oxadiazole analog of **37**. **^1^H NMR** (500 MHz, CDCl_3_) δ 8.05 (s, 1H), 7.69–7.59 (m, 3H), 7.47 (dd, *J* = 12.7, 6.7 Hz, 1H), 7.28 (dd, *J* = 18.0, 8.9 Hz, 2H), 7.06 (t, *J* = 9.0 Hz, 1H), 6.75 (q, *J* = 6.8 Hz, 1H), 2.83 (dd, *J* = 14.5, 7.1 Hz, 2H), 1.39 (t, *J* = 7.7 Hz, 3H), 1.36 (d, *J* = 10.1 Hz, 3H). **^13^C NMR** (126 MHz, CDCl_3_) δ 171.90 (s), 170.68 (s), 162.93 (s), 160.08 (d, ^1^*J_C-F_* = 251.0 Hz), 139.11 (s), 136.19 (s), 133.50 (s), 132.93 (s), 132.20 (d, ^3^*J_C-F_* = 8.3 Hz), 132.05 (s), 131.15 (s), 130.92 (s), 130.31 (s), 128.36 (d, ^2^*J_C-F_* = 13.0 Hz), 125.05 (s), 124.59 (s), 123.41 (s), 116.27 (d, ^2^*J_C-F_* = 21.4 Hz), 50.27 (s), 19.77 (s), 14.98 (s), 11.54 (s). **HRMS (ESI/IT-TOF)** *m*/*z*: [M + H] Calcd for C_22_H_17_N_5_OFCl 422.1178; found 422.1169.

#### 3.1.34. Synthesis of (R)-5-(8-Chloro-6-(2′-fluorophenyl)-4-methyl-4H-benzo[f]imidazo[1,5-a][1,4]diazepin -3-yl)-3-isopropyl-1,2,4-oxadiazole (37)

The ethyl ester, ethyl (*R*)-8-chloro-6-(2′-fluorophenyl)-4-methyl-4H-benzo[f]imidazo[1,5-a][1,4]diazepine-3-carboxylate (**24**, 1 g, 2.5 mmol) was dissolved in dry THF (20 mL) under an argon atmosphere. The isopropyl oxime, *N*-hydroxyisobutyrimidamide (1.05 g, 10 mmol) was treated with sodium hydride (60% dispersion in mineral oil, 0.065 g, 2.80 mmol) in a separate flask for an hour with molecular sieves, 3 Å, after which the ethyl ester solution was added dropwise to the oxime. The reaction mixture, which resulted, was allowed to stir at rt for 2 h until the starting material was consumed (TLC, silica gel, EtOAc: hexane = 50:50). The reaction mixture was quenched with 100% aq NaHCO_3_ solution (10 mL), diluted with water (50 mL) and extracted with EtOAc (3 x 100 mL). The combined organic layers were washed (aqueous 10% NaCl solution, 2 × 30 mL), dried (Na_2_SO_4_) and solvent was removed under reduced pressure. The solid residue was purified by flash column chromatography (silica gel, EtOAc: hexane= 50:50) to afford the pure, white-colored oxadiazole, **37** (940 mg, 86%). **^1^H NMR** (500 MHz, CDCl_3_) δ 8.05 (s, 1H), 7.69–7.55 (m, 3H), 7.46 (dd, J = 13.2, 7.2 Hz, 1H), 7.27 (dd, J = 17.0, 7.4 Hz, 2H), 7.06 (t, J = 9.0 Hz, 1H), 6.73 (q, J = 7.0 Hz, 1H), 3.18 (dt, J = 13.4, 6.6 Hz, 1H), 1.40 (d, J = 6.9 Hz, 6H), 1.36 (d, J = 7.2 Hz, 2H).**^13^C NMR** (126 MHz, CDCl_3_) δ 175.34 (s), 170.65 (s), 162.02 (d, ^1^J_C-F_ = 235.7 Hz), 139.10 (s), 136.19 (s), 135.51 (s), 133.50 (s), 132.95 (s), 132.21 (d, ^4^J_C-F_ = 8.1 Hz), 132.08 (s), 131.35 (s), 131.12 (s), 130.90 (s), 130.33 (s), 129.80 (s), 128.38 (d, ^3^J_C-F_ = 11.9 Hz), 125.14 (s), 124.62 (s), 124.29 (s), 123.44 (s), 116.29 (d, ^2^J_C-F_ = 21.4 Hz), 50.33 (s), 26.76 (s), 20.62 (s), 20.56 (s), 14.99 (s). **HRMS (ESI/IT-TOF)** *m*/*z*: [M + H] + Calcd for C_23_H_19_N_5_OFCl 436.1334; found 436.1374. % ee > 98% (HPLC).

#### 3.1.35. Synthesis of (R)-5-(8-Chloro-6-(2′-fluorophenyl)-4-methyl-4H-benzo[f]imidazo[1,5-a][1,4]diazepin -3-yl)-3-cyclopropyl-1,2,4-oxadiazole (38)

The *N*’-hydroxycyclopropanecarboximidamide (1.07 g, 10 mmol) was treated with sodium hydride (60% dispersion in mineral oil, 0.066 g, 2.75 mmol) for an hour in the presence of molecular sieves, 3 Å under an argon atmosphere and then the solution of (*R*)-8-chloro-6-(2′-fluorophenyl)- 4-methyl-4H-benzo[f]imidazo[1,5-a][1,4]diazepine-3- carboxylate (**24**, 1 g, 2.5 mmol, 20 mL THF) was added to the reaction mixture dropwise and it was allowed to stir for 2 h until consumption of starting material (TLC). The mixture was then quenched (20 mL saturated aqueous NaHCO_3_ solution), diluted (water, 50 mL) and extracted (EtOAc, 3 × 100 mL). The combined organic layers were washed (10% aq NaCl solution 2 × 50 mL), dried (Na_2_SO_4_), and the solvents were removed under reduced pressure. The residue was purified by silica gel flash chromatography (EtOAc: hexane= 50:50) to furnish pure **38** (white powder, 0.99 g, 91%). **^1^H NMR** (500 MHz, CDCl_3_) δ 8.04 (s, 1H), 7.65 (d, J = 9.1 Hz, 1H), 7.60 (s, 2H), 7.46 (td, J = 7.4, 1.5 Hz, 1H), 7.26 (dd, J = 14.1, 6.4 Hz, 2H), 7.05 (t, J = 9.2 Hz, 1H), 6.66 (q, J = 7.1 Hz, 1H), 4.39 (d, J = 6.2 Hz, 1H), 2.20–2.11 (m, 2H), 1.33 (d, J = 7.2 Hz, 3H), 1.19–1.10 (m, 2H), 1.06 (dd, J = 8.3, 2.2 Hz, 2H). **^13^C NMR** (126 MHz, CDCl_3_) δ 172.64 (s), 170.48 (s), 162.94 (s), 160.07 (d, ^1^J_C-F_ = 249.6 Hz), 139.11 (s), 136.16 (s), 135.48 (s), 133.51 (s), 132.92 (s), 132.55 (d, ^3^J_C-F_ = 8.0 Hz), 132.20 (d, ^4^J_C-F_ = 7.6 Hz), 132.05 (s), 131.10 (s), 130.88 (s), 130.32 (s), 129.80 (s), 128.36 (d, ^2^J_C-F_ = 12.0 Hz), 125.01 (s), 124.61 (s), 124.28 (s), 123.41 (s), 116.28 (d, ^2^J_C-F_ = 21.4 Hz), 50.29 (s), 14.98 (s), 7.76 (s), 6.90 (s). **HRMS (ESI/IT-TOF**) *m*/*z*: [M + H] + Calcd for C_23_ H_17_ N_5_OF Cl 434.1178; found 434.1210. %ee => 98% (HPLC).

#### 3.1.36. Synthesis of (R)-3-Cyclopropyl-5-(8-ethynyl-6-(2′-fluorophenyl)-4-methyl-4H-benzo[f]imidazo[1,5-a][1,4]diazepin-3-yl)-1,2,4-oxadiazole (39)

After treating the *N*’-hydroxycyclopropanecarboximidamide (1.03 g, 10.3 mmol) with sodium hydride (60% dispersion in mineral oil, 0.068 g, 2.75 mmol) for 1 h at rt, the solution was added to the stirred solution of ethyl (*R*)-6-(2′-fluorophenyl)-4-methyl-8-((triisopropylsilyl) ethynyl)-4H- benzo[f]imidazo[1,5-a][1,4]diazepine-3-carboxylate (**34**, 1 g, 2.58 mmol) in anhydrous THF (20 mL). The mixture, which resulted, was then allowed to stir for 2 h at rt until consumption of starting material was observed (TLC). The mixture was quenched with a saturated aqueous solution of NaHCO_3_ (20 mL), diluted with water (10 mL), and extracted with EtOAc (3 × 20 mL). The combined organic layers were washed (10% aq NaCl solution, 2 × 50 mL) and dried (Na_2_SO_4_). The solvents were removed under reduced pressure and the residue was purified by silica gel flash chromatography to provide pure **39** as a white powder (896 mg, 82%). R_f_ = 0.6 (silica gel, 70% EtOAc-hexane). **^1^H NMR** (500 MHz, CDCl_3_) δ 8.07 (s, 1H), 7.75 (dd, J = 14.6, 7.3 Hz, 1H), 7.63 (t, J = 13.9 Hz, 2H), 7.51–7.40 (m, 2H), 7.26 (d, J = 7.5 Hz, 1H), 7.06 (t, J = 9.3 Hz, 1H), 6.68 (q, J = 7.1 Hz, 1H), 3.18 (s, 1H), 2.16 (ddd, J = 16.5, 8.2, 3.9 Hz, 1H), 1.34 (d, J = 7.3 Hz, 2H), 1.16 (dd, J = 11.9, 9.7 Hz, 2H), 1.07 (dd, J = 8.4, 2.3 Hz, 2H). **^13^C NMR** (126 MHz, CDCl_3_) δ 172.67 (s), 170.43 (s), 163.76 (s), 160.10 (d, ^1^*J_C-F_* = 250.9 Hz), 139.08 (s), 136.24 (s), 135.52 (s), 134.24 (s), 133.70 (d, ^2^*J_C-F_* = 20.2 Hz), 132.30 (d, ^3^*J_C-F_* = 8.7 Hz), 131.19 (s), 129.38 (d, ^4^*J_C-F_* = 2.6 Hz), 128.24 (d, ^3^*J_C-F_* = 5.1 Hz), 125.05 (s), 124.58 (d, J = 3.3 Hz), 123.01 (s), 122.24 (s), 121.95 (s), 116.29 (d, ^2^*J_C-F_* = 21.4 Hz), 81.29 (s), 79.97 (s), 50.15 (s), 15.02 (s), 7.76 (s), 6.90 (s). **HRMS (ESI/IT-TOF**) *m*/*z*: [M + H] + Calcd for C_25_H_18_N_5_OF 424.1568; found 424.1573. %ee > 99%.

#### 3.1.37. Synthesis of (S)-3-Ethyl-5-(8-ethynyl-4-methyl-6-(pyridin-2′-yl)-4H-benzo[f]imidazo[1,5-a][1,4]diazepin-3-yl)-1,2,4-oxadiazole (40)

The oxadiazole **40** was prepared from the ethyl ester, ethyl (*S*)-8-ethynyl-4-methyl-6-(pyridin-2′-yl)-4H-benzo[f]imidazo[1,5-a][1,4]diazepine-3-carboxylate (**20A**, 1 g, 2.7 mmol), *N*-hydroxypropionoimidamide (0.95 g, 10.8 mmol), sodium hydride (60% dispersion in mineral oil, 0.072 g, 2.97 mmol) and THF (20 mL) by following the synthetic procedure employed for the synthesis of oxadiazole **40**. The crude residue was purified by flash column chromatography (silica gel, EtOAc/hexane 3:2) to yield pure oxadiazole **40** as a white powder (883 mg, 83 %). R_f_ = 0.6 (silica gel, 70% EtOAc-hexane with 1% TEA). ^**1**^**H NMR** (500 MHz, CDCl_3_) δ 8.61 (d, *J* = 2.4 Hz, 1H), 8.16–7.97 (m, 2H), 7.86 (t, *J* = 7.4 Hz, 1H), 7.78 (dd, *J* = 18.6, 8.3 Hz, 1H), 7.61 (d, *J* = 8.3 Hz, 1H), 7.44 (d, *J* = 36.3 Hz, 2H), 6.74 (q, *J* = 7.1 Hz, 1H), 3.18 (s, 1H), 2.85 (q, *J* = 7.5 Hz, 2H),1.40 (t, *J* = 7.6 Hz, 3H), 1.37 (d, *J* = 7.2 Hz, 3H). **^13^C NMR** (126 MHz, CDCl3) δ 171.92 (s), 170.72 (s), 165.24 (s), 148.48 (s), 137.22 (s), 136.44 (s), 135.99 (s), 135.77 (s), 135.62 (s), 135.33 (s), 127.80 (s), 124.98 (s), 124.85 (s), 124.20 (s), 123.17 (s), 122.42 (s), 121.27 (s), 81.57 (s), 79.64 (s), 50.05 (s), 19.79 (s), 14.72 (s), 11.55 (s). **HRMS (ESI/IT-TOF**) *m*/*z*: [M + H] + Calcd for C_23_H_18_N_6_O 395.1614; found 395.1621.

#### 3.1.38. Synthesis of Ethyl 8-cyclopropyl-6-(2′-fluorophenyl)-4H-benzo[f]imidazo[1,5-a][1,4]diazepine- 3-carboxylate (43)

The Pd(OAc)_2_ (0.11 g, 0.49 mmol),and tri-(o-tolyl) phosphine (0.298 g, 0.98 mmol) were dissolved in toluene (10 mL) and the mixture was stirred for 10 min under an argon atmosphere to generate the Pd(OAc)_2_P(*o*-tol)_3_ catalyst in-situ. Then the imidazodiazepine ethyl 8-bromo-6-(2′-fluorophenyl)-4H-benzo[f]imidazo[1,5-a][1,4]diazepine- 3-carboxylate (**42**, 3 g, 7 mmol), cyclopropyl boronic acid (1.8 g, 21 mmol), tri-basic potassium phosphate (5.93 g, 28 mmol), water (0.5 mL, 25 mmol), and additional toluene (15 mL) were added sequentially to the previous reaction mixture under argon. The reaction mixture was then allowed to stir at 100 °C for 12 h and the consumption of starting material was confirmed on the LCMS 2020 (single quadrupole mass analyzer). The R_f_ value on TLC (silica gel; neutral alumina; 60% ethyl acetate-hexane) for both the starting material and product was almost identical. That is why the completion of the reaction needed to be confirmed by LCMS 2020. The reaction mixture was then cooled and opened to the air once all the starting material was consumed. The reaction mixture was passed through a pad of celite beads to remove any palladium salts. The filtrate was diluted with water (20 mL) and ethyl acetate (30 mL). The biphasic mixture, which resulted, was allowed to stand to separate the layers. The organic layer was separated, and the aqueous layer was extracted (2 × 10 mL). The combined organic layers were washed with a 10% aqueous solution of NaCl (3 × 10 mL) and dried (Na_2_SO_4_). The solvents were removed under reduced pressure. The orange-colored residue, which resulted, was purified by flash chromatography (silica gel, 70% EtOAc-hexane). The desired fractions were pooled, and the solvents were removed under reduced pressure. The solid residue was dried under vacuum at 40 °C for 2 h to afford pure **43** as an off-white powder (1.95 g, 72%). **^1^H NMR** (500 MHz, CDCl_3_) δ 7.93 (s, 1H), 7.63 (td, *J* = 7.5, 1.7 Hz, 1H), 7.48 (d, *J* = 8.3 Hz, 1H), 7.46–7.41 (m, 1H), 7.29 (dd, *J* = 7.9, 2.4 Hz, 1H), 7.24 (t, *J* = 7.5 Hz, 1H), 7.03 (dd, *J* = 15.1, 5.3 Hz, 2H), 6.08 (d, *J* = 3.0 Hz, 1H), 4.43 (q, J= 7.1 Hz, 2H), 4.11 (d, *J* = 10.8 Hz, 1H), 1.95–1.83 (m, 1H), 1.43 (t, *J* = 7.1 Hz, 3H), 1.02 (dd, *J* = 8.4, 1.2 Hz, 2H), 0.85–0.57 (m, 2H). **^13^C NMR** (126 MHz, CDCl_3_) δ 166.40 (s), 163.04 (s), 160.26 (d, ^1^*J_C-F_* = 251.5 Hz), 144.08 (s), 138.45 (s), 134.29 (s), 132.02 (s), 131.85 (s), 131.33 (d, ^4^*J_C-F_* = 2.4 Hz), 129.04 (s), 128.89 (s), 128.76 (s), 128.17 (d, ^2^*J _C-F_* = 12.0 Hz), 127.87 (s), 124.34 (d, ^3^*J_C-F_* = 3.5 Hz), 122.35 (s), 116.07 (d, ^2^*J_C-F_* = 21.7 Hz), 60.67 (s), 44.96 (s), 15.10 (s), 14.48 (s), 9.86 (s). **HRMS (ESI/IT-TOF)** *m*/*z*: [M + H] + Calcd for C_23_H_20_N_3_O_2_F 390.1612; found 390.1617.

#### 3.1.39. Synthesis of 5-(8-Cyclopropyl-6-(2′-fluorophenyl)-4H-benzo[f]imidazo[1,5-a][1,4]diazepin- 3-yl)-3- ethyl-1,2,4-oxadiazole (44)

The ethyl oxime, *N*-hydroxypropionoimidamine (0.9 g, 10.2 mmol) was dissolved in dry THF (20 mL) under argon and treated with sodium hydride (60% dispersion in mineral oil, 0.068 g, 2.86 mmol) for an hour with molecular sieves, 3 Å in a round bottom flask. The 8-cyclopropyl ethyl ester (**43,** 1 g, 2.56 mmol) was dissolved in dry THF (20 mL) in a separate flask at rt under argon and then added to the flask containing the oxime. The reaction mixture, which resulted, was stirred at rt for 2 h until the starting material was consumed, as indicated by analysis by TLC (silica gel, EtOAc: hexane = 50:50). The reaction mixture was quenched with 100% aqueous NaHCO_3_ solution (20 mL). Water (50 mL) was then added, and the product was extracted with EtOAc (3 × 100 mL). The organic layers were combined, washed (aqueous 10% NaCl solution, 2 × 30 mL) and dried (Na_2_SO_4_). The solvent was removed under reduced pressure. The solid, which resulted, was purified by flash column chromatography (silica gel, EtOAc: hexane= 50:50) to afford the pure oxadiazole, **44** (white powder, 850 mg, 80%). **^1^H NMR** (500 MHz, CDCl_3_) δ 8.05 (s, 1H), 7.64 (t, J = 7.5 Hz, 1H), 7.53 (d, J = 8.3 Hz, 1H), 7.47–7.41 (m, 1H), 7.32 (d, J = 8.3 Hz, 1H), 7.24 (t, J = 7.5 Hz, 1H), 7.07 (s, 1H), 7.03 (t, J = 9.4 Hz, 1H), 6.14 (d, J = 10.9 Hz, 1H), 4.23 (d, J = 7.5 Hz, 1H), 2.84 (qd, J = 7.6, 1.1 Hz, 2H), 1.95–1.88 (m, 1H), 1.40 (td, J = 7.5, 1.1 Hz, 3H), 1.03 (d, J = 8.3 Hz, 2H), 0.67 (s, 2H). **^13^C NMR** (126 MHz, CDCl_3_) δ 171.85 (s), 170.94 (s), 166.63 (s), 160.26 (d, ^1^J _C-F_ = 251.6 Hz), 144.35 (s), 136.22 (s), 135.60 (s), 132.10 (d, ^2^J _C-F_ = 8.4 Hz), 131.64 (s), 131.31 (d, ^4^J _C-F_ = 2.3 Hz), 129.04 (s), 128.78 (s), 128.15 (s), 128.06 (s), 128.02 (s), 124.47 (s), 124.36 (d, ^3^J _C-F_ = 3.5 Hz), 122.32 (s), 116.10 (d, ^2^J _C-F_ = 21.6 Hz), 44.82 (s), 19.77 (s), 15.13 (s), 11.57 (s), 9.91 (s). **HRMS** (ESI/IT-TOF) *m*/*z*: [M + H] + Calcd for C_24_ H_20_ N_5_O F 414.1725; found 414.1691.

#### 3.1.40. Synthesis of (R)-5-(8-Bromo-6-(2′-fluorophenyl)-4-methyl-4H-benzo[f]imidazo[1,5-a][1,4]diazepin-3-yl)-3-ethyl-1,2,4-oxadiazole (45)

The *N*-hydroxypropionoimidamine (3.98 g, 45.2 mmol) was treated with sodium hydride (60% dispersion in mineral oil, 0.298 g, 12.4 mmol) for an hour in the presence of molecular sieves (3 Å), in a round bottom flask and this solution was added to the stirred solution of 8-bromo ethyl ester, ethyl (*R*)-8-bromo-6-(2′-fluorophenyl)-4-methyl-4H-benzo[f]imidazo[1,5-a][1,4]diazepine-3-carboxylate (**2,** 5 g, 11.3 mmol) in dry THF (40 mL) in a separate flask under argon. The reaction mixture, which resulted, was stirred at rt for 3 h. The staring material was not consumed fully due to impure NaH. 40% of the starting material remained as indicated by analysis by TLC (silica gel, EtOAc: hexane = 50:50). The reaction mixture was quenched with 100% aqueous NaHCO_3_ solution (20 mL) and the product was extracted with EtOAc (3 x 100 mL). The organic layers were combined, washed (aqueous 10% NaCl solution, 2x 30 mL) and dried (Na_2_SO_4_). The solvent was removed under reduced pressure. The solid, which resulted, was purified by flash column chromatography (silica gel, EtOAc: hexane= 50:50) to afford the pure oxadiazole, **45** as a white powder (2.1 g, 40%). 2 g of starting material were recovered from the reaction. **^1^H NMR** (500 MHz, CDCl_3_) δ 8.05 (s, 1H), 7.78 (dd, *J* = 23.6, 7.8 Hz, 1H), 7.62 (dd, *J* = 24.2, 17.3 Hz, 1H), 7.56–7.41 (m, 3H), 7.27 (dd, *J* = 13.0, 5.4 Hz, 1H), 7.06 (t, *J* = 9.1 Hz, 1H), 6.74 (dd, *J* = 14.2, 7.0 Hz, 1H), 2.83 (dd, *J* = 14.7, 7.3 Hz, 2H), 1.38 (t, *J* = 7.6 Hz, 3H), 1.36 (d, *J* = 7.7 Hz, 3H). **^13^C NMR** (126 MHz, CDCl_3_) δ 171.89 (s), 170.66 (s), 162.84 (s), 160.08 (d, ^1^*J_C-F_* = 251.1 Hz), 139.13 (s), 136.16 (s), 135.31 (d, ^2^*J_C-F_* = 42.5 Hz), 135.01 (s), 133.39 (s), 133.20 (s), 132.21 (d, ^4^*J_C-F_* = 8.3 Hz), 131.14 (s), 128.33 (d, ^3^*J_C-F_* = 12.1 Hz), 125.04 (s), 124.59 (s), 123.61 (s), 121.18 (s), 116.27 (d, ^2^*J_C-F_* = 21.4 Hz), 50.26 (s), 19.76 (s), 14.99 (s), 11.54 (s). **HRMS** (ESI/IT-TOF) *m*/*z*: [M + H] + Calcd for C_22_H_17_N_5_OFBr 466.0673; found 466.0678.

#### 3.1.41. Synthesis of (R)-5-(8-Cyclopropyl-6-(2′-fluorophenyl)-4-methyl-4H-benzo[f]imidazo[1,5-a][1,4]diazepin-3-yl)-3-ethyl-1,2,4-oxadiazole (46)

To a solution of the bromo ethyl oxadiazole (**45**, 1.1 g, 2.38 mmol), tri(*o*-tolyl)phosphine (85.5 mg, 0.28 mmol), cyclopropyl boronic acid (0.724 g, 8.43 mmol) and potassium phosphate (2.56 g, 12.1 mmol) in toluene (30 mL) and water (0.65 mL), Pd(OAc)_2_ (31.5 mg, 0.14 mmol) was added under argon at rt to form a cloudy orange-colored solution. A reflux condenser was attached. The mixture was allowed to stir at rt for 5 min until the color of the solution turned yellow, as an indication of the formation of the Pd complex generated in situ. The mixture was then placed into a pre-heated oil bath at 100 °C. After 2 h the reaction progress was complete on analysis by TLC (silica gel), and it was then cooled to rt. Then water (20 mL) was added, and the mixture was extracted with EtOAc (3 × 25 mL), after which the filtrate was washed with brine (20 mL), dried (Na_2_SO_4_) and concentrated under reduced pressure. The black residue which resulted was purified by a wash column (silica gel, 60% EtOAc-hexane) to afford the desired C(8)-cyclopropyl ethyl oxadiazole (**46**) as a white solid (815.5 mg, 81.6 %): **^1^H NMR** (300 MHz, CDCl_3_) δ 7.98 (s, 1H), 7.46 (d, *J =* 8.3 Hz, 2H), 7.38–7.25 (m, 1H), 7.13 (t, *J =* 7.5 Hz, 2H), 6.93 (t, *J =* 9.1 Hz, 2H), 6.63 (q, *J =* 7.0 Hz, 1H), 2.73 (q, *J =* 7.6 Hz, 2H), 1.84–1.65 (m, 1H), 1.27 (dd, *J =* 14.1, 6.6 Hz, 6H), 0.90 (d, *J =* 8.3 Hz, 2H), 0.54 (dd, *J =* 10.1, 4.8 Hz, 2H); ^13^C NMR (126 MHz, CDCl_3_) δ 171.79 (s), 170.96 (s), 164.35 (s), 160.15 (d, ^1^*J_C-F_* = 249.3 Hz), 144.18 (s), 139.25 (s), 136.27 (s), 131.79 (d, ^4^*J_C-F_* = 3.2 Hz), 131.70 (s), 131.20 (s), 129.21 (s), 129.01 (d, ^3^*J_C-F_* = 5.7 Hz), 128.62 (s), 128.62 (s), 128.06 (s), 124.40 (d, ^2^*J_C-F_* = 12.6 Hz), 121.89 (s), 116.06 (d, ^2^*J_C-F_* = 21.8 Hz), 50.20 (s), 19.74 (s), 15.09 (s), 14.77 (s), 11.54 (s), 9.94 (s), 3.77 (s).; **HRMS** (ESI/IT-TOF) *m*/*z*: [M + H] Calcd for C_25_H_23_FN_5_O 428.1881, found 428.1889.

### 3.2. The Determination of Optical Purity Method 1

The separation of R and S enantiomers was carried out using an Agilent 1100 HPLC system with a Chiral pak IBN3 column (4.6 mm × 150 mm, 3µm particle size) and an isocratic mixture of 90% n-hexane and 10% ethanol as the mobile phase. The quaternary pump, autosampler, and DAD detector were also part of the system. The DAD wavelength range was set at 200–400 nm, and a wavelength of 254 nm was used for relative % area determination. The flow rate for the method was 1 mL/min. To verify the method, a racemic mixture was used. The enantiomeric excess was calculated using the following formula:

% ee = (R − S)/(R + S) × 100.

### 3.3. The Determination of Optical Purity Method 2

The R and S enantiomers of any carboxylic acid compounds were separated using an isocratic mixture of 90% n-hexane and 10% ethanol with 0.5% TFA as the mobile phase on an Agilent 1100 HPLC system comprising of a quaternary pump, autosampler, and a DAD detector. A Chiral pak IBN3 column (4.6 mm × 150 mm, 3µm particle size) was used to resolve the enantiomers. The method was verified using a racemic mixture. The DAD wavelength range was set at 200–400 nm, and a 254 nm wavelength was used for relative % area determination. The flow rate for this method was set at 1 mL/min. The enantiomeric excess was calculated using the formula:

% ee = (R − S)/(R + S) × 100.

### 3.4. Radioligand Binding Assays

The NIMH PDSP assay protocol book [https://kidbdev.med.unc.edu/databases/PDSP%20Protocols%20II%202013-03-28.pdf accessed on 23 April 2023]. describes the protocols for primary and secondary radio-ligand binding assays [83]. These assays were carried out in a final volume of 125 µL per well in the appropriate binding buffer. The concentration of the radioactive ligand was close to the K_d_, with [3H]-U69593 at 0.83 nM, [3H]-DAMGO at 1.20 nM, and [3H]-DADLE at 2.69 nM.

The binding of a ligand to a receptor, ion channel, or transporter was determined in the absence and presence of 10 µM of the appropriate reference compound. Nonspecific binding was also determined in the absence and presence of 10 µM of the appropriate reference compound. Plates were incubated in the dark for 90 min at rt, and the reactions were stopped by vacuum filtration onto 0.3% polyethyleneimine (PEI) soaked 96-well filter mats using a 96-well Filtermateharvester. This was followed by three washes with cold PBS buffer. Radioactivity was then counted on a Microbeta counter. Nonlinear regression was used to analyze the data with N = 6 replicates.

## 4. Conclusions

Our findings indicate that the newly synthesized analogsshowed negligible binding or no binding to any off-target profile receptors at concentrations which could cause other physiological problems. The determination of antidepressant, anxiolytic, and procognitive effects are underway and will be reported in due course. The interest in antidepressant agents with procognitive properties has increased because the related amide (monomethyl amide) has shown activity in the absence of psychosis in a rodent model (in press). Moreover, this ligand (MP-III022) was active in autism and schizophrenia. As mentioned earlier imidazodiazepines are not benzodiazepines and the former are often devoid of the side effects of benzodiazepines.

## 5. Patents

International Patent Application No. PCT//US2022/042832 (Atty. File No. 020871-9178-WO01). Etienne Sibille, Thomas Prevot, and James Cook are listed as authors of the patent. This patent was licensed -to Damona Pharmaceuticals, a company co-founded by Etienne Sibille.

## Figures and Tables

**Table 1:** Binding affinities of imidazodiazepines at hERG channels.

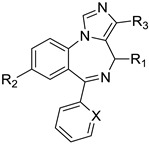 Core Structure of the Novel Imidazodiazepines					
X	R2	R3	R1	Compound	Binding Affinity at hERG % Inhibition (Primary Binding) *
C-F	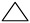	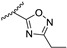	(R)-CH3	**46**	4.34 ± 3.922
C-F	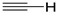	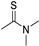	(R)-CH3	**33**	−4.4 ± 4.52
C-Cl	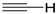	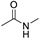	(R)-CH3	**21**	−5.73 ± 11.12
N	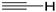	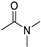	(S)-CH3	**31**	0.95 ± 2.21
N	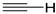	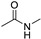	(S)-CH3	**23**	−4.24 ± 3.47
C-Cl	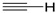	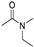	(R)-CH3	**29**	−1.55 ± 10.12
C-F	Cl	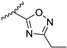	(R)-CH3	**38**	−1.15 ± 19.61
C-F	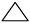	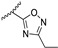	H	**44**	33.24 ± 12.61
C-F	Ph	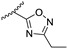	(R)-CH3	**35**	37.34 ± 5.97
C-F	Ph	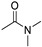	(R)-CH3	**5**	−0.48 ± 4.52
C-F	Cl	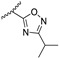	(R)-CH3	**37**	4.21 ± 10.78
C-F	Cl	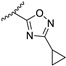	(R)-CH3	**38**	1.86 ± 25.56

* Percent of inhibition at 10,000 nM. N = 5, The concentration of the radioactive ligand was close to the Kd, with [3H]-U69593 at 0.83 nM, [3H]-DAMGO at 1.20 nM, and [3H]-DADLE at 2.69 nM.

**Table 2:** Binding affinities at peripheral benzodiazepine receptors (PBR) for the novel compounds.

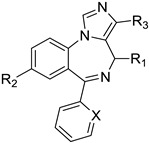 Core Structure of the Novel Imidazodiazepines						
X	R2	R3	R1	Compound	Binding Affinity at PBR	
					% Inhibition (Primary) *	Ki (nM) (Secondary Screen)
C-F	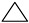	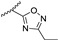	(R)-CH3	**46**	86.38 ± 6.35	627.34 ± 5.72
C-F	Cl	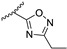	(R)-CH3	**36**	57.19 ± 10.32	982.2 ± 404.51
C-F	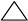	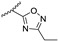	H	**47**	71.03 ± 13.62	1109.99 ± 15.18
C-F	Ph	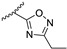	(R)-CH3	**35**	71.76 ± 10.47	1114.56 ± 24.5
C-F	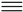	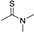	(R)-CH3	**33**	66.42 ± 3.3	1712.3
C-F	Cl	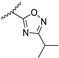	(R)-CH3	**37**	88.03 ± 1.32	838.24 ± 134.66
C-F	Cl	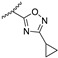	(R)-CH3	**38**	89.95 ± 2.13	617.14 ± 27.07

* Percent of inhibition at 10,000 nM. Ki values for secondary screening are in nM. N = 5 for primary screening. The concentration of the radioactive ligand was close to the Kd, with [3H]-U69593 at 0.83 nM, [3H]-DAMGO at 1.20 nM, and [3H]-DADLE at 2.69 nM. N = 2 for secondary screening.

**Table 3:** Opioid receptor binding of the novel imidazodiazepine.

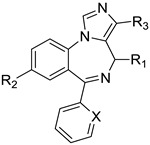 Core Structure of the Novel Imidazodiazepines								
X	R2	R3	R1	Compound	Primary Binding			KOR * (Ki, nM)
					%KOR *	%MOR *	%DOR *	
C-F	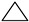	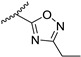	(R)--CH3	**47**	91.32 ± 3.06	25.15 ± 12.76	67.34 ± 7.84	248.03 ± 22.58
C-F	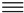	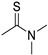	(R)--CH3	**33**	53.57 ± 7.24	−2.4 ± 5.72	13.2 ± 7.83	399.77 ± 81.95
C-Cl	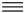	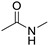	(R)--CH3	**21**	69.57 ± 6.01	−3.57 ± 7.68	28.27 ± 4.67	660.47 ± 159.2
N	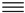	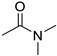	(S)-CH3	**31**	−3.79 ± 4.99	4.55 ± 12.47	6.18 ± 11.18	NA
N	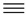	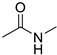	(S)-CH3	**23**	26.87 ± 6.21	5.24 ± 10.73	14.92 ± 8.84	NA
C-Cl	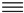	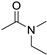	(R)--CH3	**29**	69.11 ± 29.11	7.31 ± 9.54	39.52 ± 3.21	392.26 ± 78.84
C-F	Cl	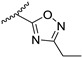	(R)--CH3	**36**	96.42 ± 3.00	19.2 ± 5.47	40.73 ± 10.16	104.01 ± 7.58
C-F	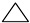	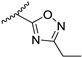	H	**44**	96.26 ± 2.29	26.44 ± 6.01	81.35 ± 4.76	207.18 ± 33.15
C-F	Ph	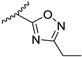	(R)--CH3	**35**	37.97 ± 1.73	14.82 ± 6.32	52.73 ± 4.38	NA
C-F	Ph	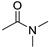	(R)--CH3	**5**	53.58 ± 7.24	−11.18 ± 5.72	42.97 ± 7.83	2144.35 ± 311.03
C-F	Cl	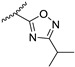	(R)--CH3	**37**	99.21 ± 1.75	48.6 ± 4.17	31.95 ± 9.58	46.33 ± 4.93
C-F	Cl	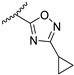	(R)--CH3	**38**	98.86 ± 1.60	30.22 ± 6.46	42.78 ± 8.944	82.24 ± 2.43
C-F	Cl	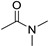	(R)--CH3	**32**	81.41 ± 7.52	11.24 ± 5.47	14.38 ± 7.98	730.77 ± 238.01

* Percent of inhibition at 10,000 nM. Ki values for secondary screening are in nM. N = 5 for primary screening. The concentration of the radioactive ligand was close to the Kd, with [3H]-U69593 at 0.83 nM, [3H]-DAMGO at 1.20 nM, and [3H]-DADLE at 2.69 nM. N = 2 for secondary screening.

**Table 4:** Binding affinities at the BZP rat brain site (PDSP) for the novel compounds.

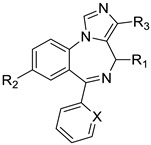 Core Structure of the Novel Imidazodiazepines						
X	R_2_	R_3_	R_1_	Compound	Binding Affinity BZR	
					% Inhibition (Primary)*	Secondary Screening Ki(nM)
C-F	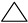	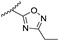	(*R*)--CH_3_	**46**	65.25 ± 5.33	3791.32 ± 632.23
C-F	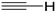	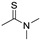	(*R*)--CH_3_	**33**	94.79 ± 1.08	871.14 ± 8.72
C-Cl	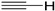	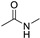	(*R*)--CH_3_	**21**	94.96 ± 2.06	77.65 ± 2.93
N	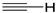	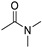	(*S*)-CH_3_	**31**	83.57 ± 1.57	838.37 ± 107.14
N	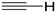	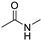	(*S*)-CH_3_	**23**	52.97 ± 6.26	1299.44 ± 393.85
C-Cl	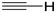	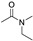	(*R*)--CH_3_	**29**	87.49 ± 2.75	394.44 ± 40.19
C-F	Cl	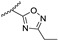	(*R*)--CH_3_	**36**	81.18 ± 3.12	589.72 ± 44.56
C-F	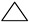	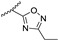	H	**44**	96.52 ± 2.57	193.89 ± 62.71
C-F	Ph	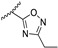	(*R*)--CH_3_	**35**	19.91 ± 7.21	_
C-F	Ph	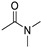	(*R*)--CH_3_	**5**	94.79 ± 1.08	123.2 ± 22.3

* Percent of inhibition at 10,000 nM. Ki values for secondary screening are in nM. N = 5 for primary screening. The concentration of the radioactive ligand was close to the Kd, with [3H]-U69593 at 0.83 nM, [3H]-DAMGO at 1.20 nM, and [3H]-DADLE at 2.69 nM. N = 2 for secondary screening.

## Data Availability

The study data are accessible upon request from the corresponding author. However, data cannot be publicly accessed due to limitations imposed by patent regulations.

## Supplementary Materials

The following supporting information can be downloaded at: https://www.mdpi.com/article/10.3390/molecules28124771/s1, ^1^HNMR and ^13^CNMR spectra; HRMS spectra; PDSP raw data.

## Sample Availability

Samples of the various compounds are available from the authors.
