# Supplementary material for: Synthesis and Receptor Binding Studies of α5 GABAAR Selective Novel Imidazodiazepines Targeted for Psychiatric and Cognitive Disorders
Source: Molecules. 2023 Jun 14;28(12):4771. doi: 10.3390/molecules28124771 (PMC10303271; doi:10.3390/molecules28124771)
Supplement: Supplementary file 1 [file molecules-28-04771-s001.zip › molecules-2353316-supplementary.pdf]

Synthesis and Receptor Binding Studies of α5 GABA$_A$R Selective Novel Imidazodiazepines Targeted for Psychiatric and Cognitive Disorders

Dishary Sharmin [1,*], Md Yeunus Mian [1], Michael Marcotte [2], Thomas D. Prevot [2,3], Etienne Sibille [2,3,4], Jeffrey M. Witkin [1,5] and James M. Cook [1,*]

[1] Department of Chemistry and Biochemistry, Milwaukee Institute of Drug Discovery, University of Wisconsin Milwaukee, Milwaukee, WI 53201, USA; mmian@uwm.edu (M.Y.M.); witkinconsult@gmail.com (J.M.W.)
[2] Campbell Family Mental Health Research Institute of CAMH, Toronto, ON M5S 2S1, Canada;
michael.marcotte@camh.ca (M.M.); thomas.prevot@camh.ca (T.D.P.); etienne.sibille@camh.ca (E.S.)
[3] Department of Psychiatry, University of Toronto, Toronto, ON M5T 1R8, Canada
[4] Department of Pharmacology and Toxicology, University of Toronto, Toronto, ON M5T 1R8, Canada
[5] Laboratory of Antiepileptic Drug Discovery, Ascension, St. Vincent, Indianapolis, IN 46260, USA
* Correspondence: dsharmin@uwm.edu (D.S.); capncook@uwm.edu (J.M.C.)

Table of Contents

Figure S1- Figure S60. ^{1}HNMR and ^{13}C NMR spectra of compounds……………………… pages **S2-S83**

Figure S61 –Figure S85. HRMS spectra of compounds……………………………………… pages **S84-S110**

Table S1- Table S12. PDSP raw data of compounds………………………………………… pages **S110- S132**

Synthetic scheme of 24, 34, and 42……………………………………………………………….. pages **S132-S138**

3

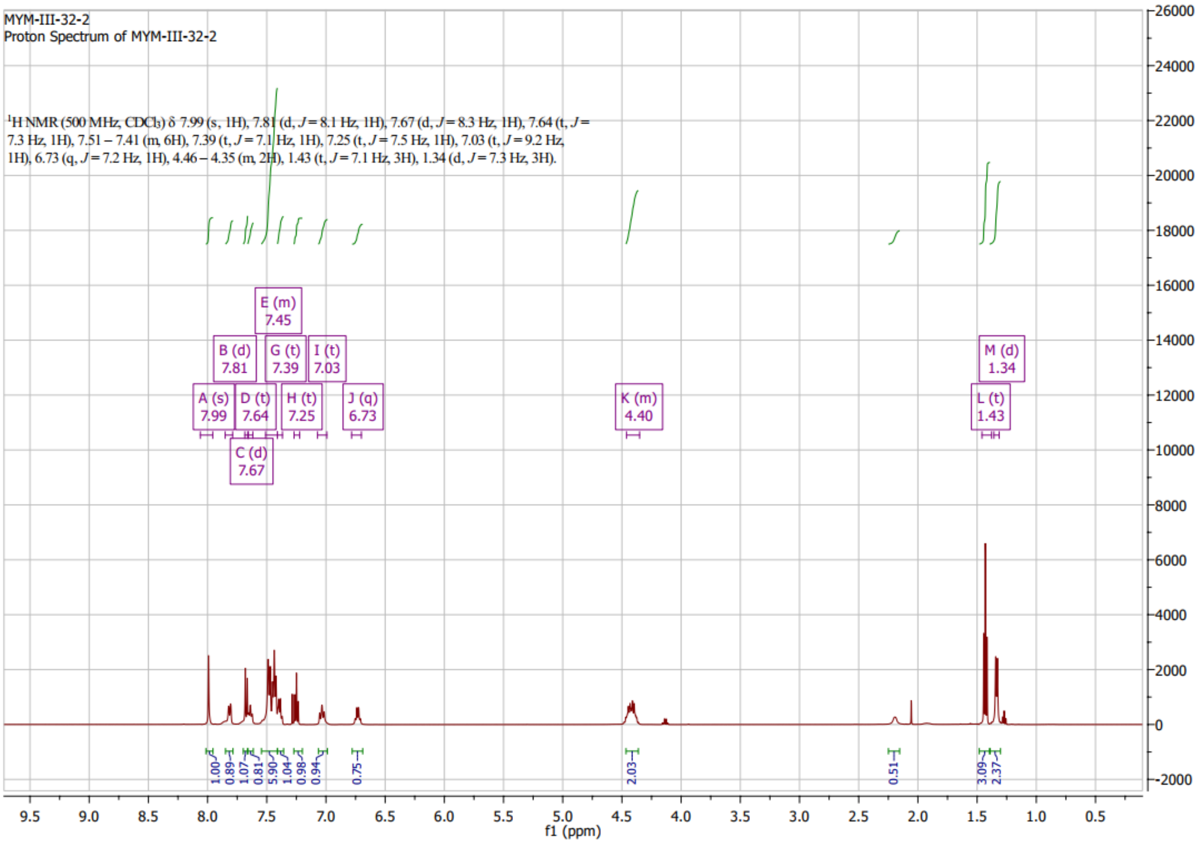

Figure S1. ^{1}HNMR spectrum of compound **3**

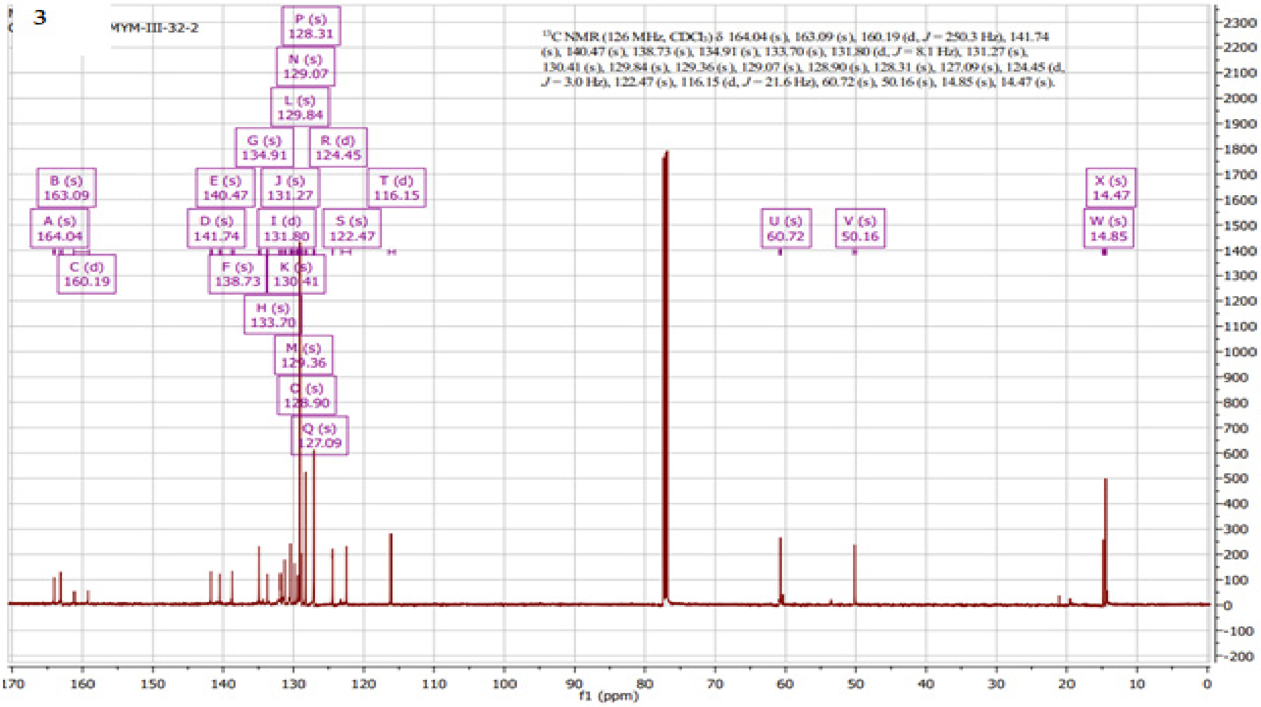

Figure S2. ^{13}CNMR spectrum of compound **3**

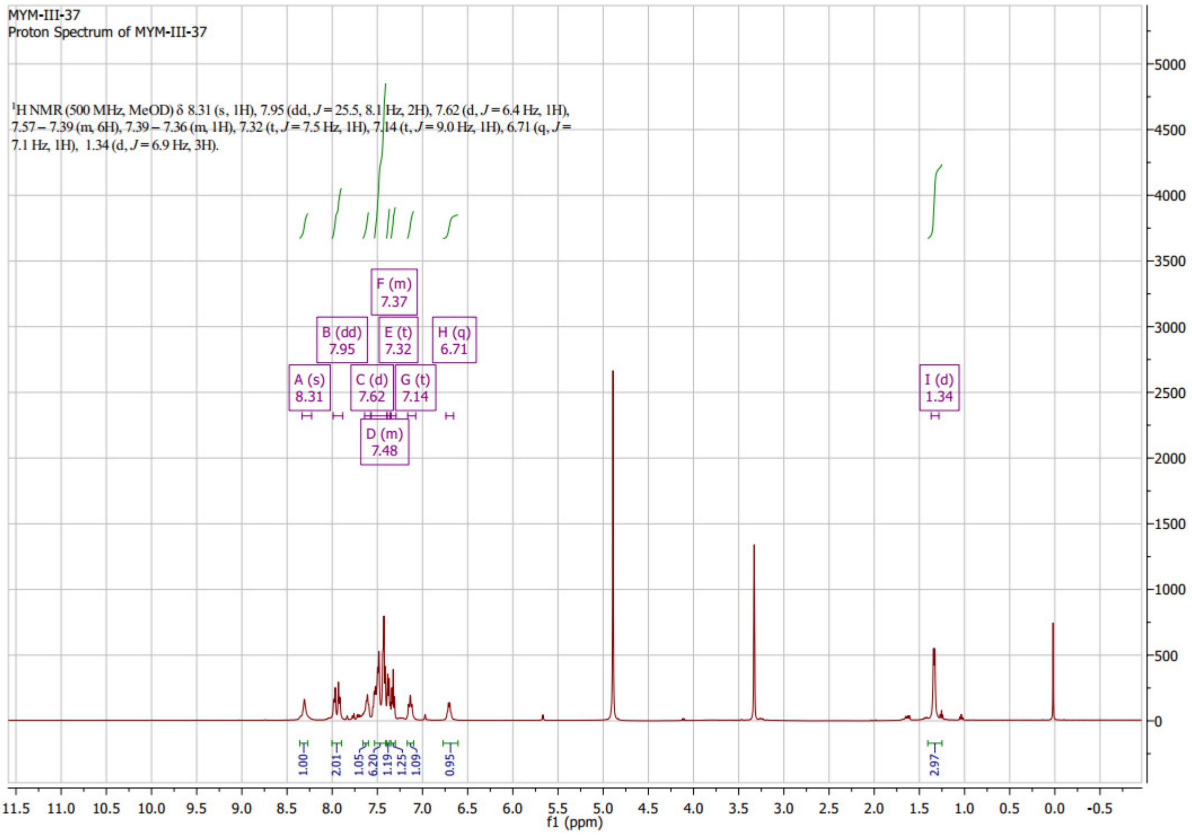

Figure S3. ^{1}HNMR spectrum of compound **4**

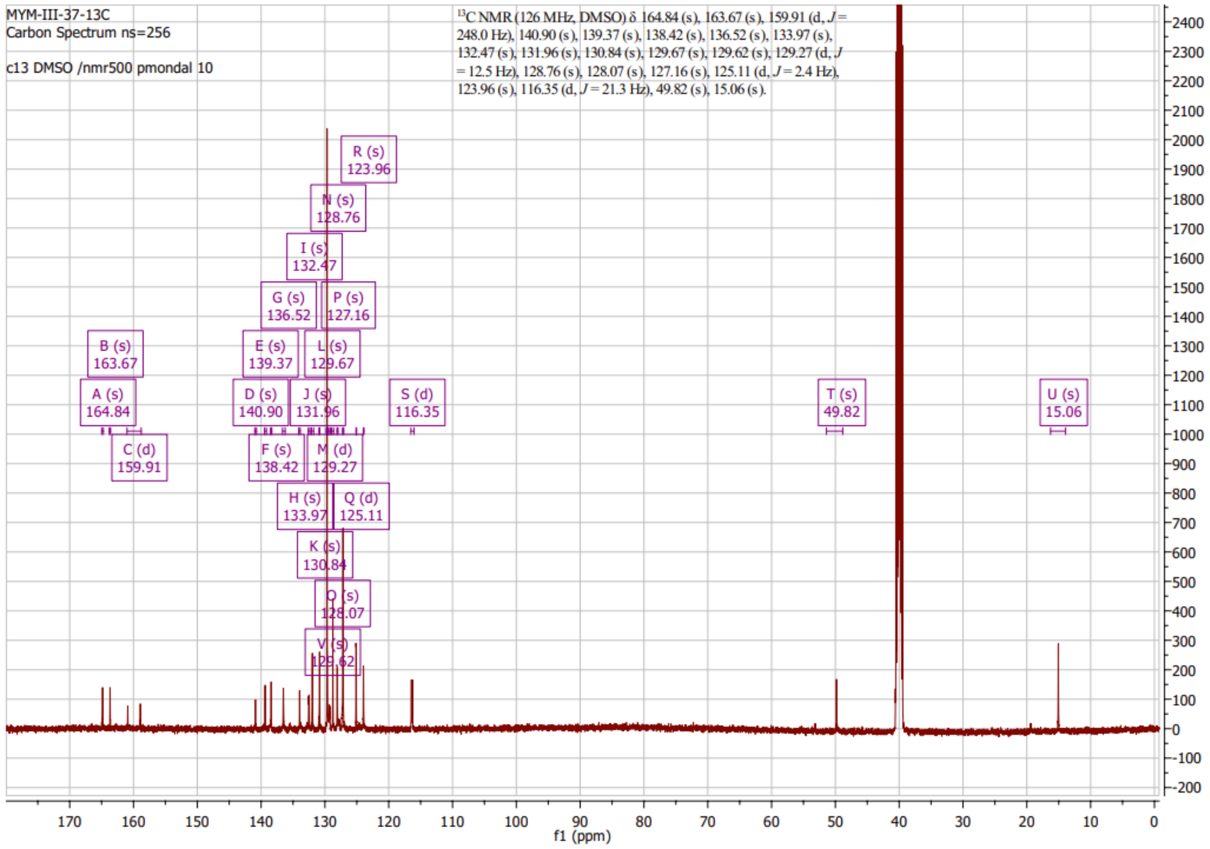

Figure S4. ^{13}CNMR spectrum of compound **4**

5

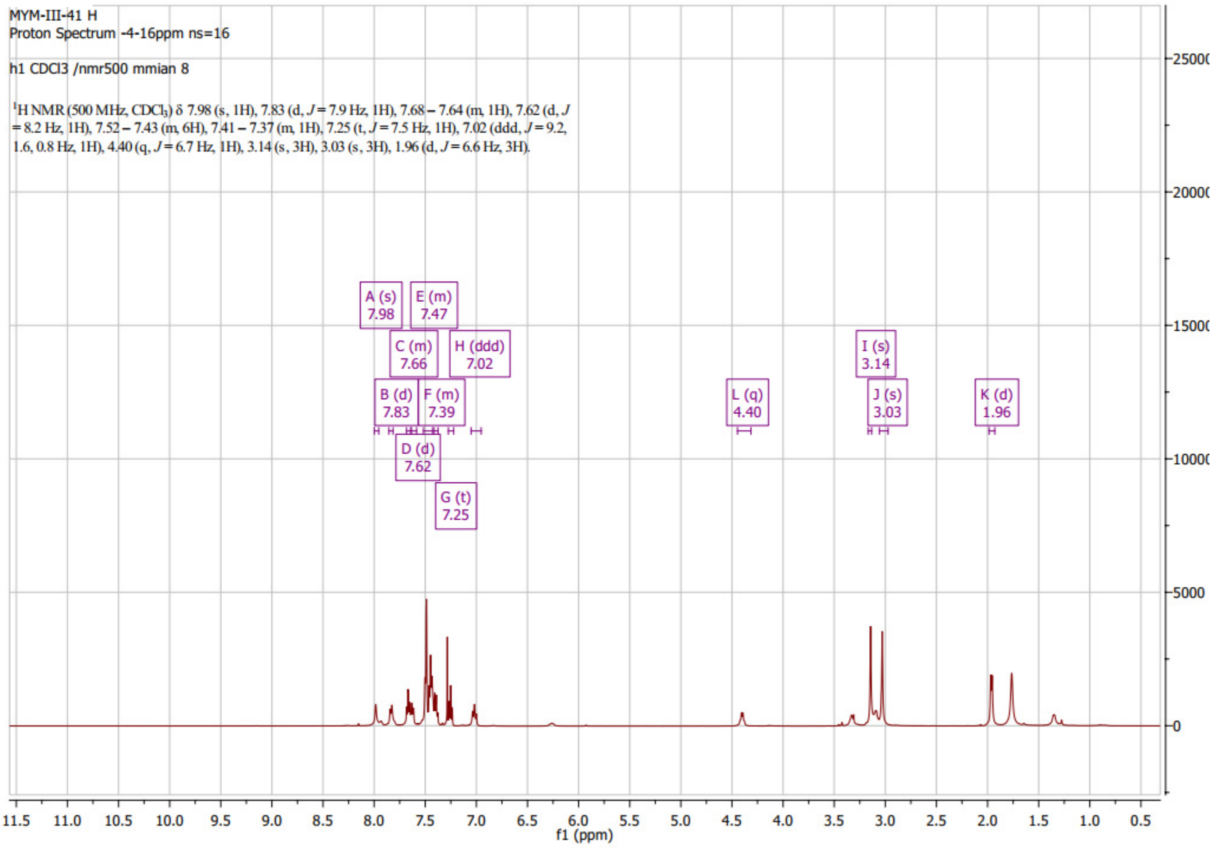

Figure S5. ^{1}HNMR spectrum of compound **5**

5

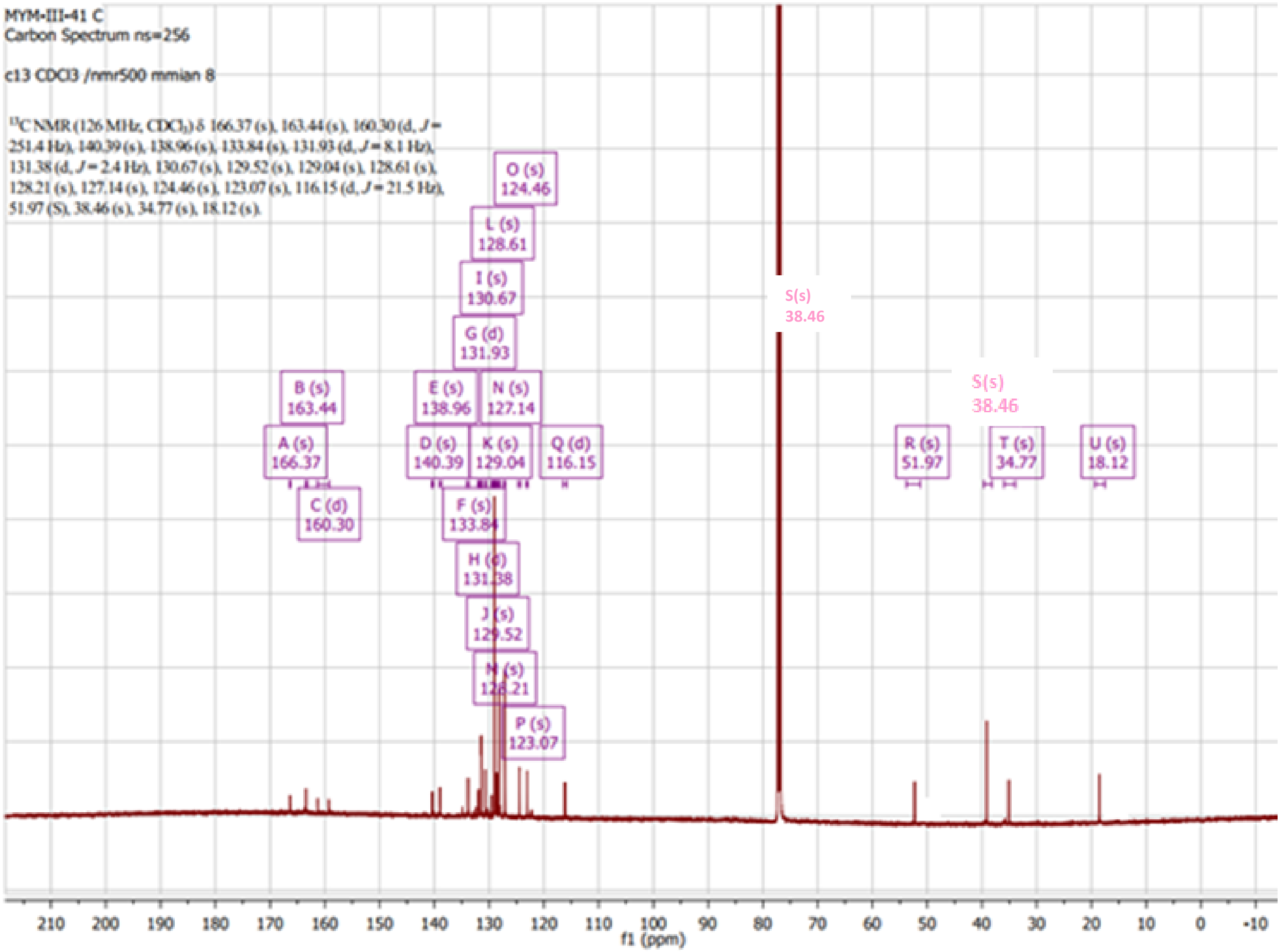

Figure S6. 13CNMR spectrum of compound **5**

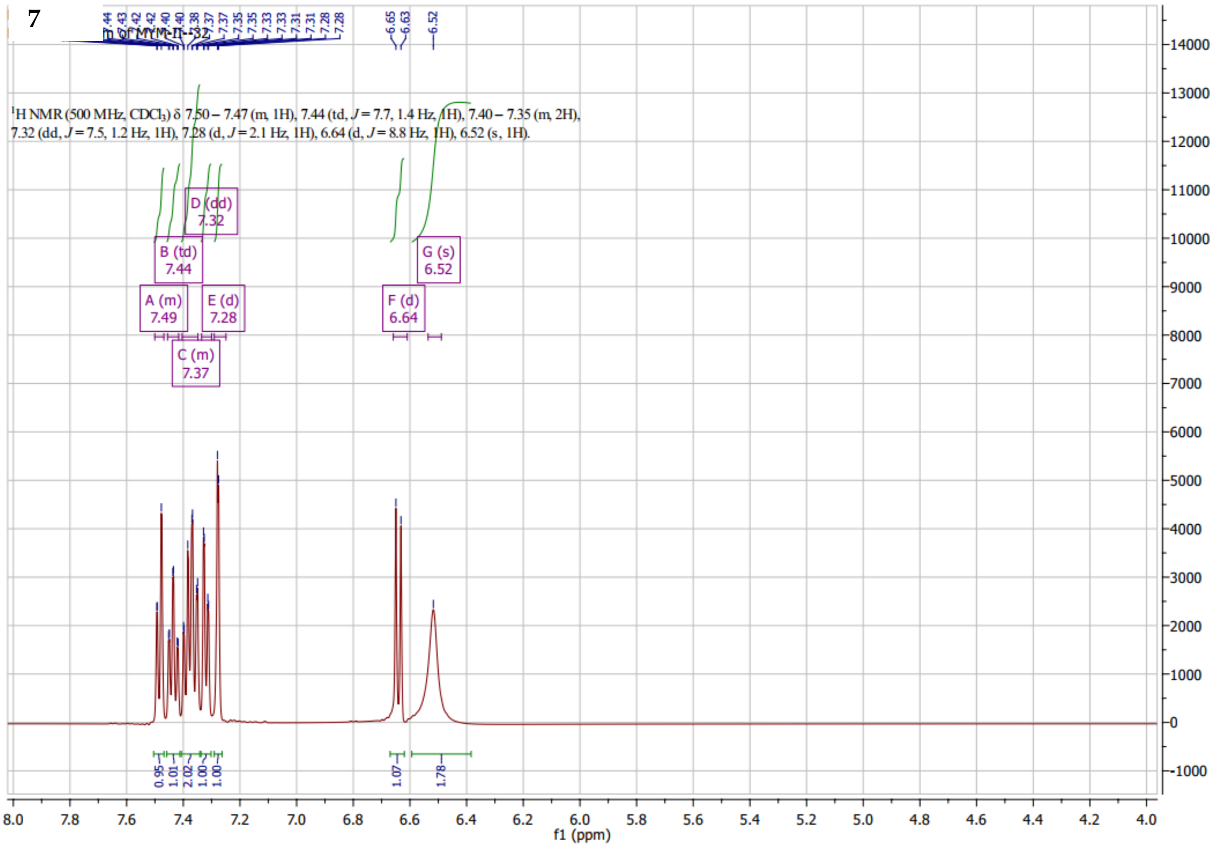

Figure S7. ^{1}HNMR spectrum of compound **7**

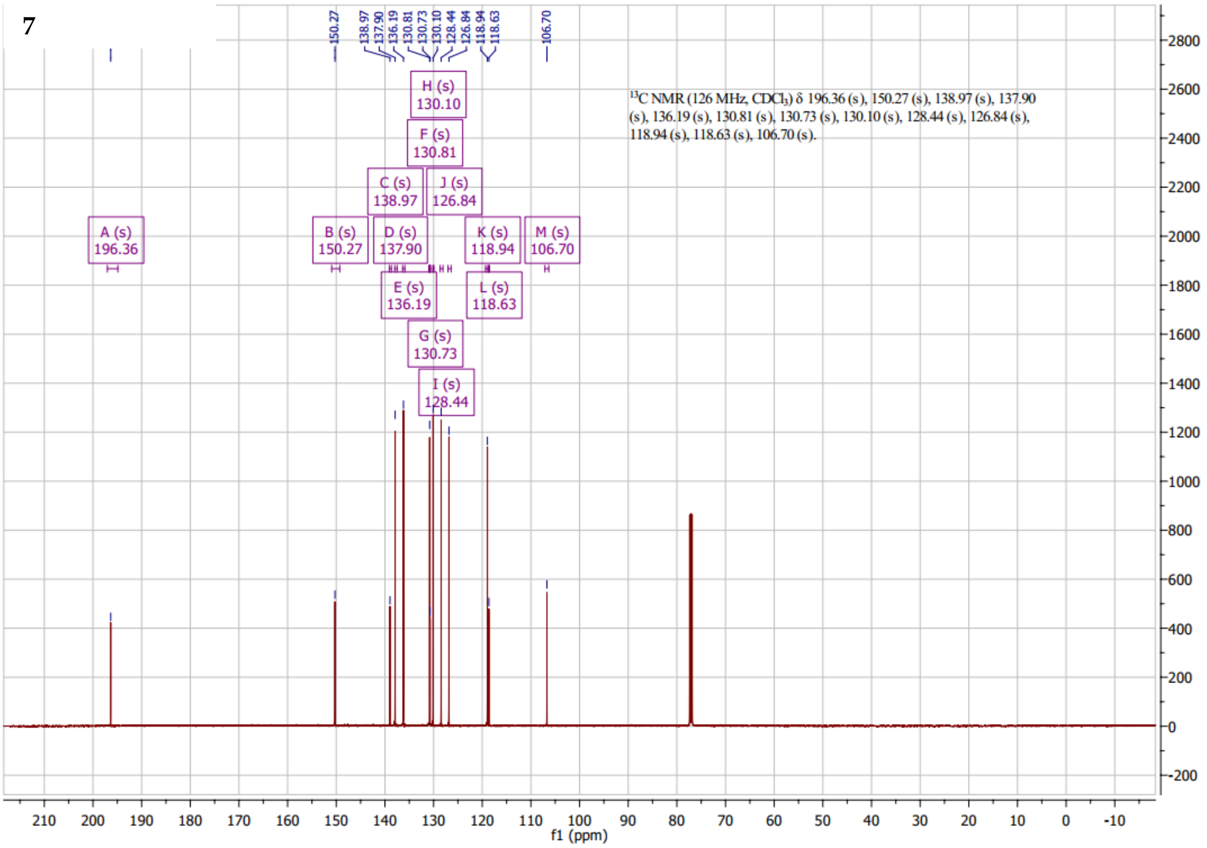

Figure S8. ^{13}CNMR spectrum of compound **7**

9

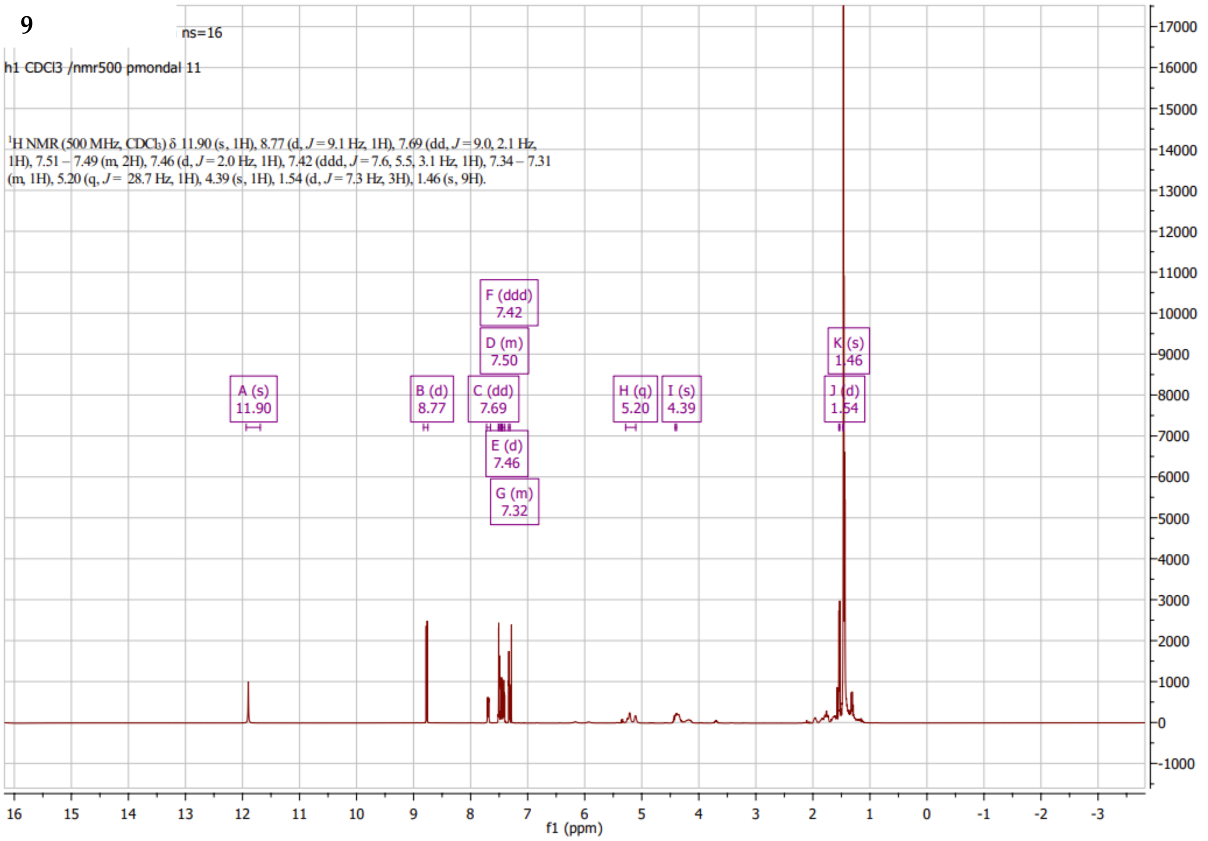

Figure S9. ^{1}HNMR spectrum of compound **9**

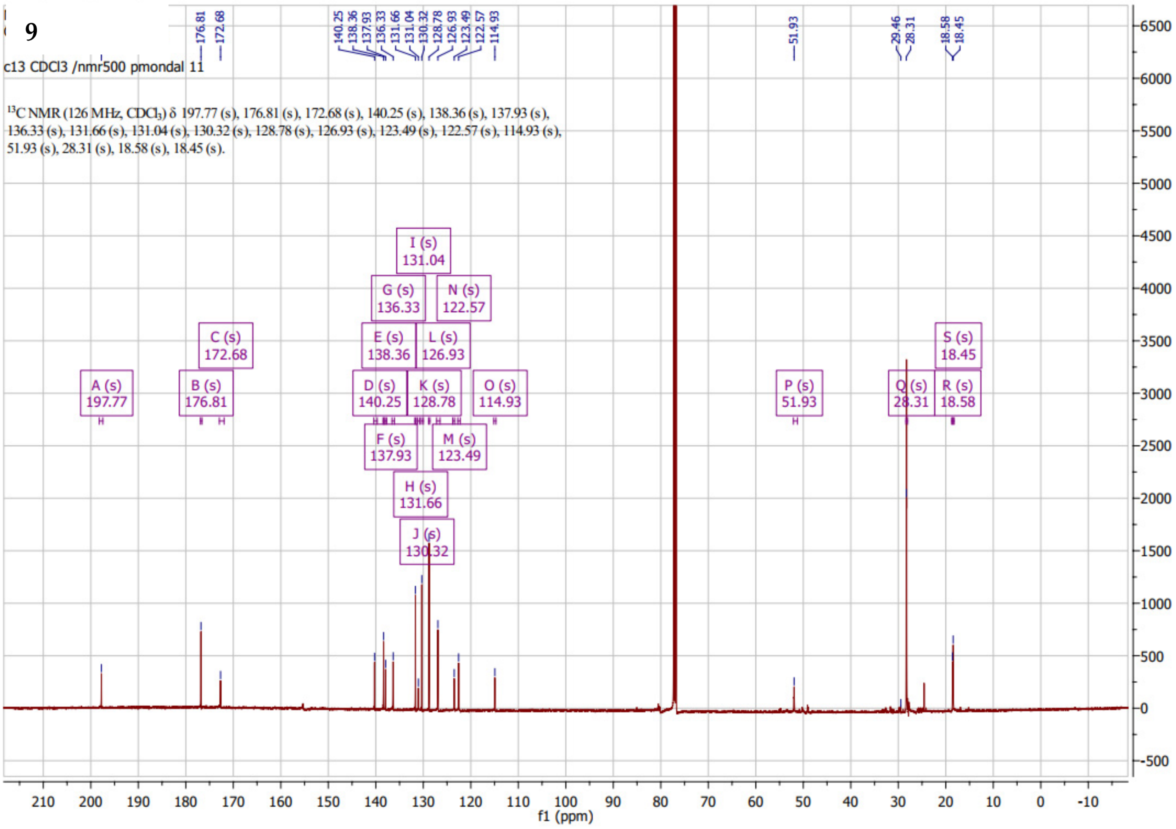

Figure S10. 13CNMR spectrum of compound **9**

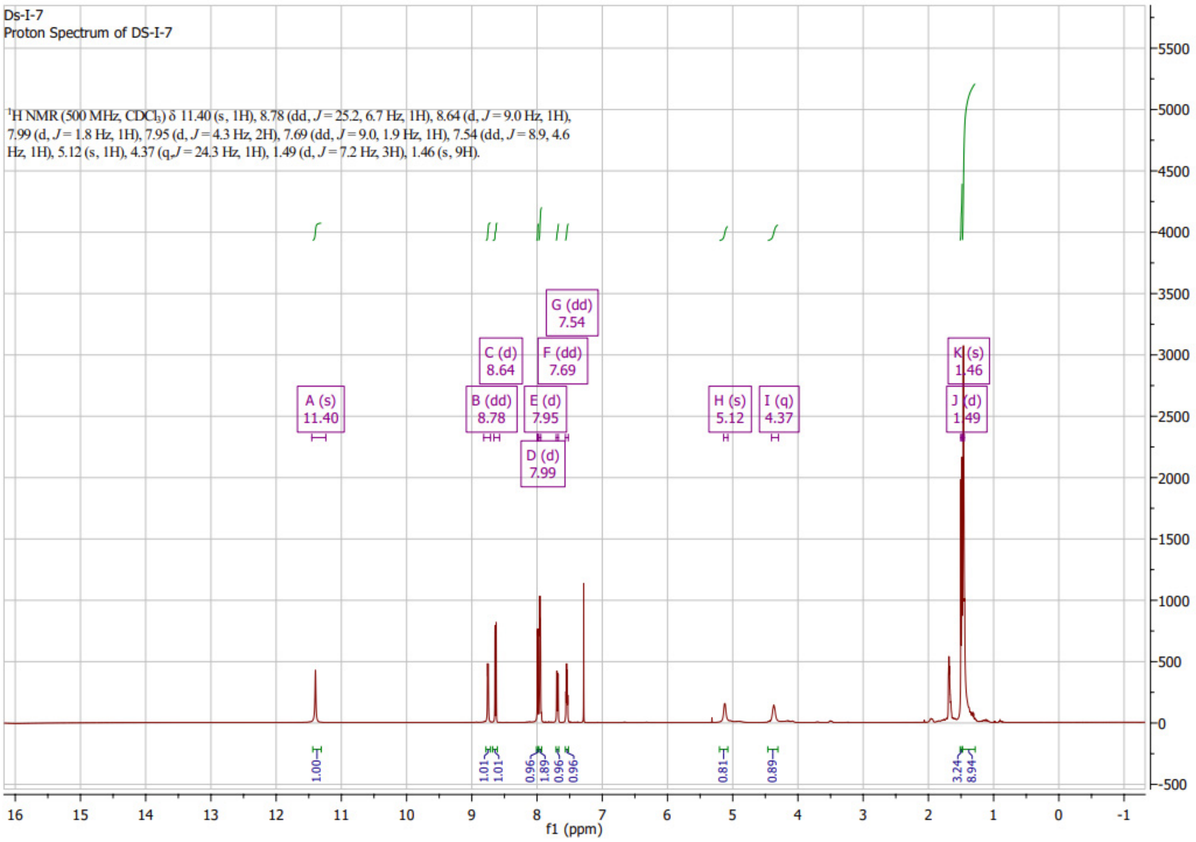

Figure S11. ^{1}HNMR spectrum of compound **10**

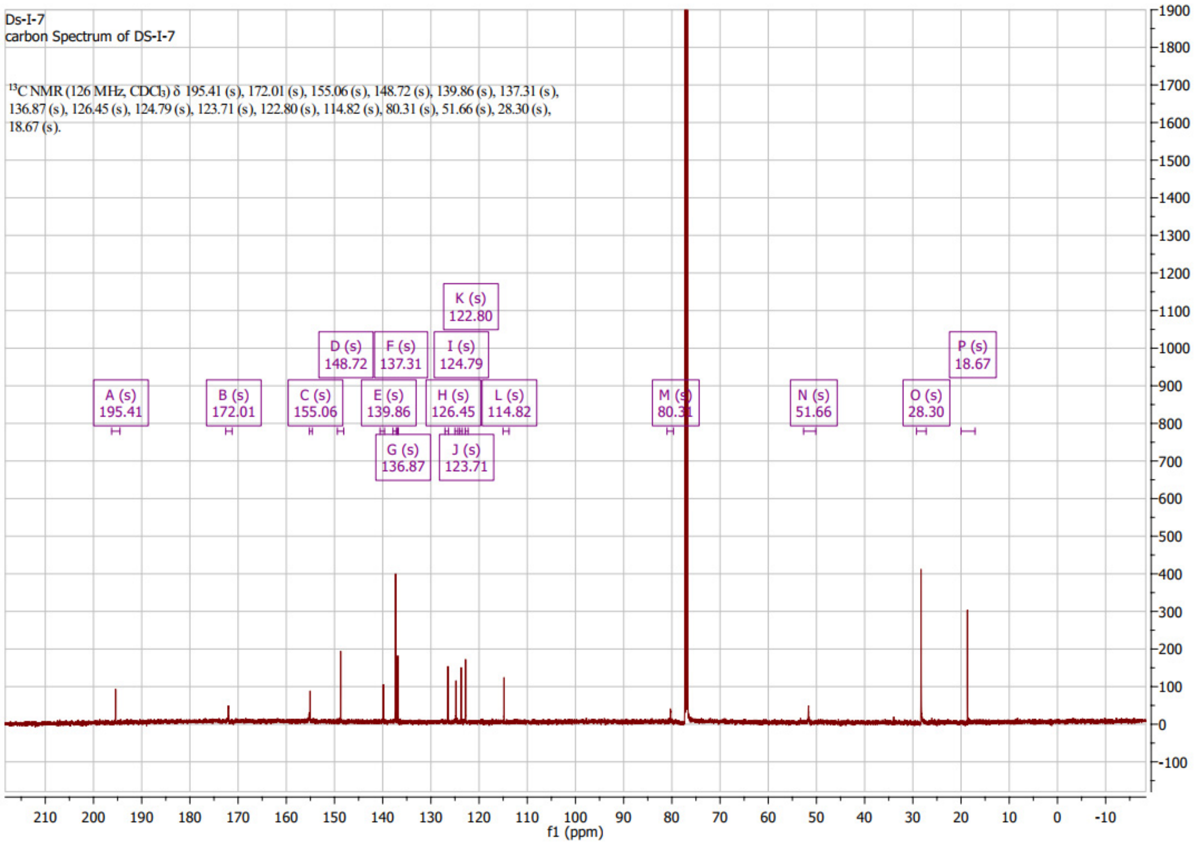

Figure S12. ^{13}CNMR spectrum of compound **10**

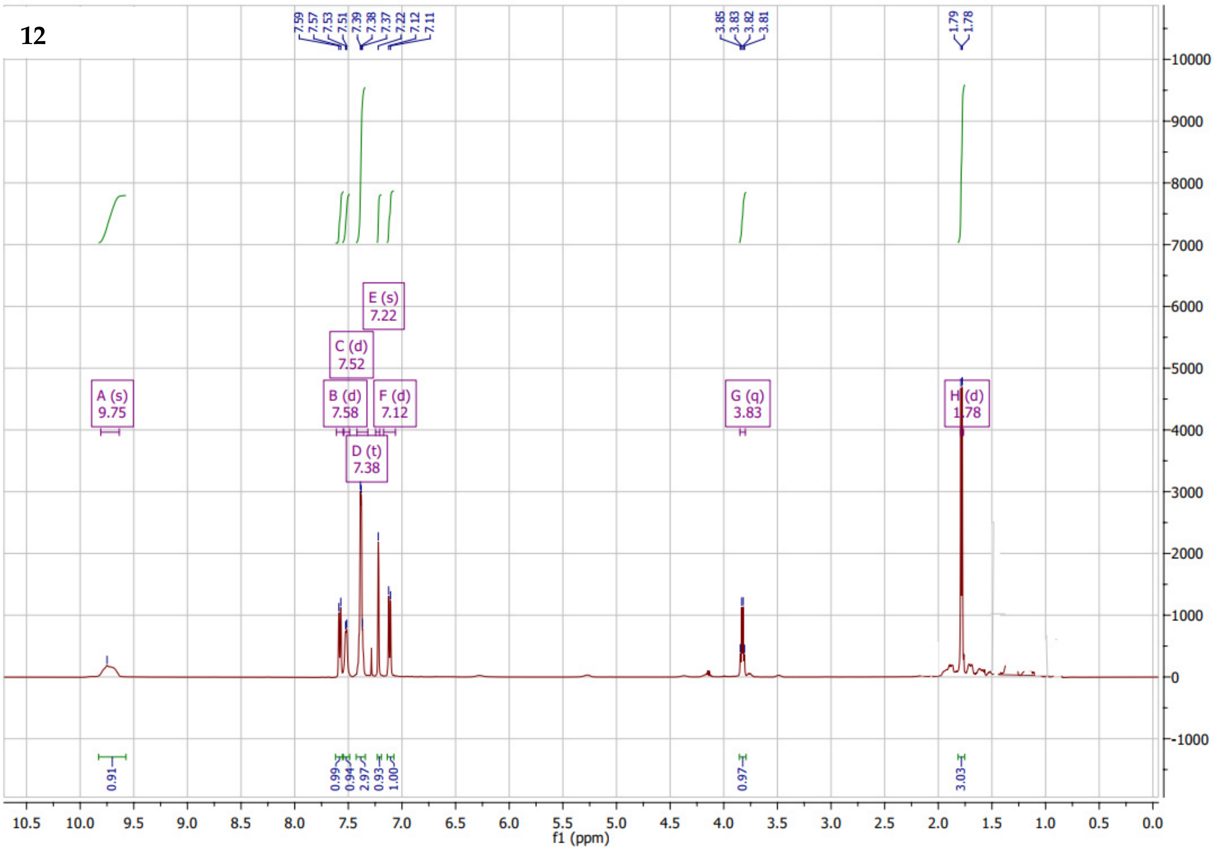

Figure S13. ^{1}HNMR spectrum of compound **12**

12

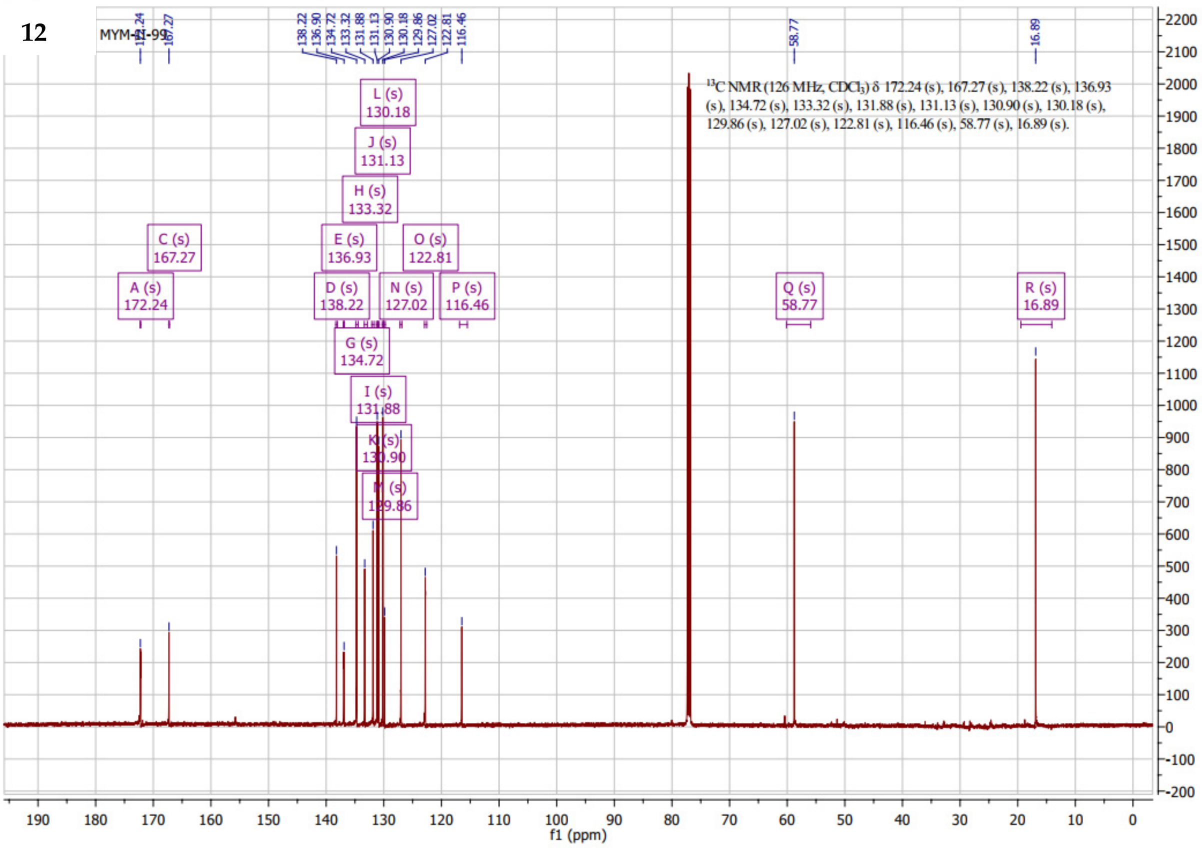

Figure S14. ^{13}CNMR spectrum of compound **12**

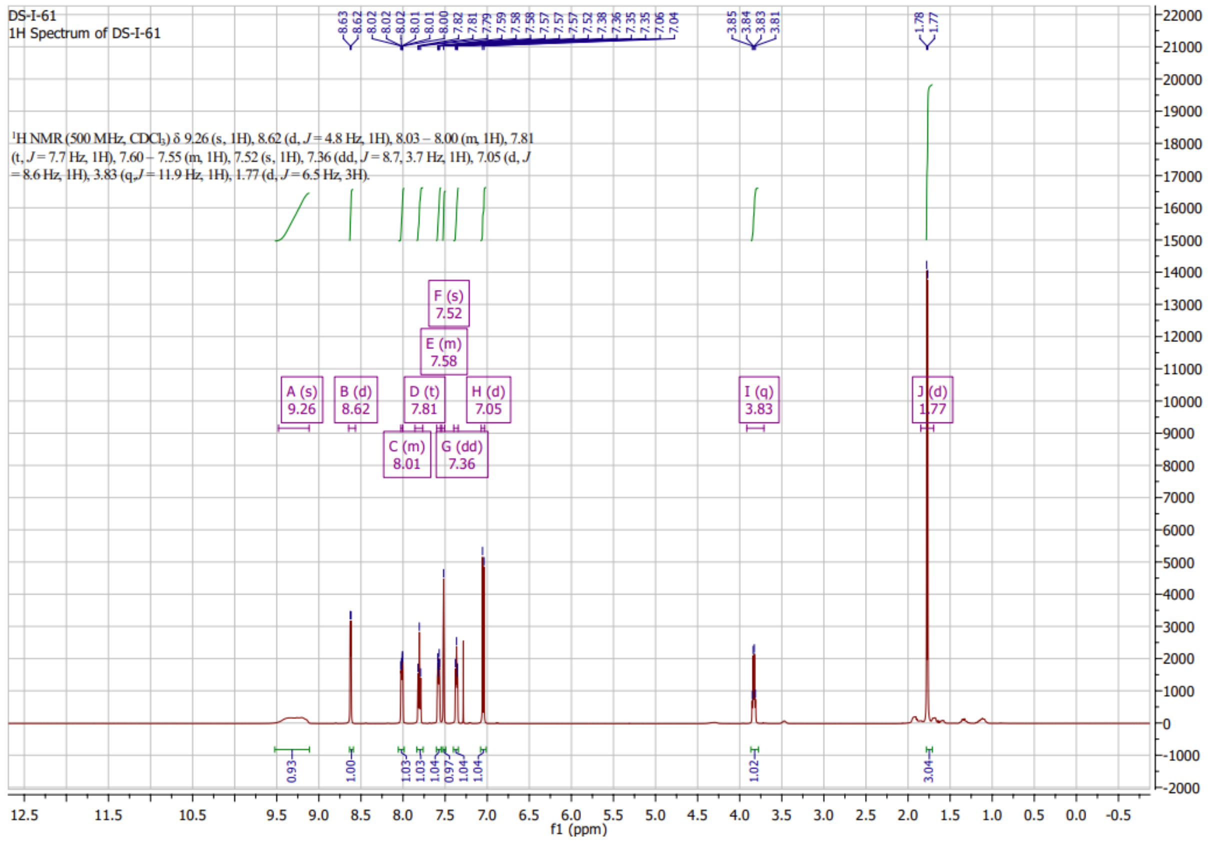

Figure S15. ¹HNMR spectrum of compound **13**

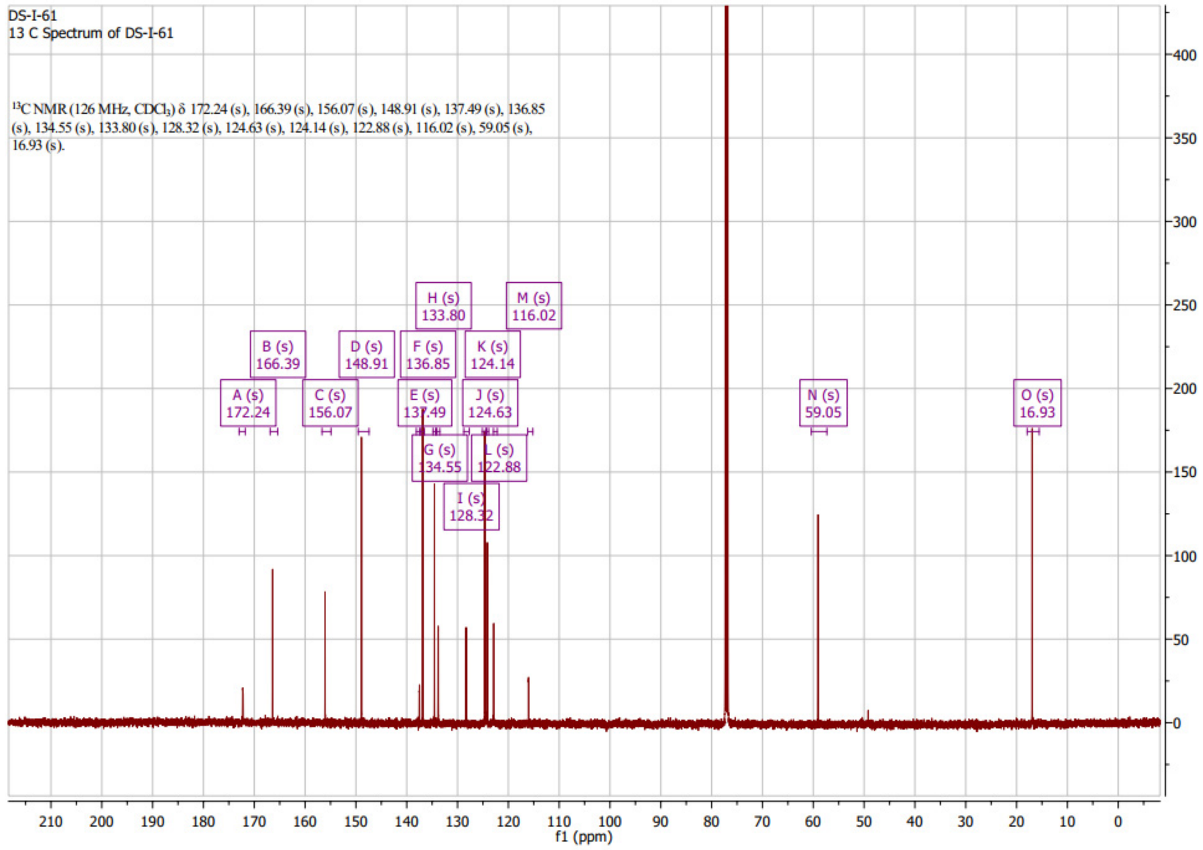

Figure S16. ^{13}CNMR spectrum of compound **13**

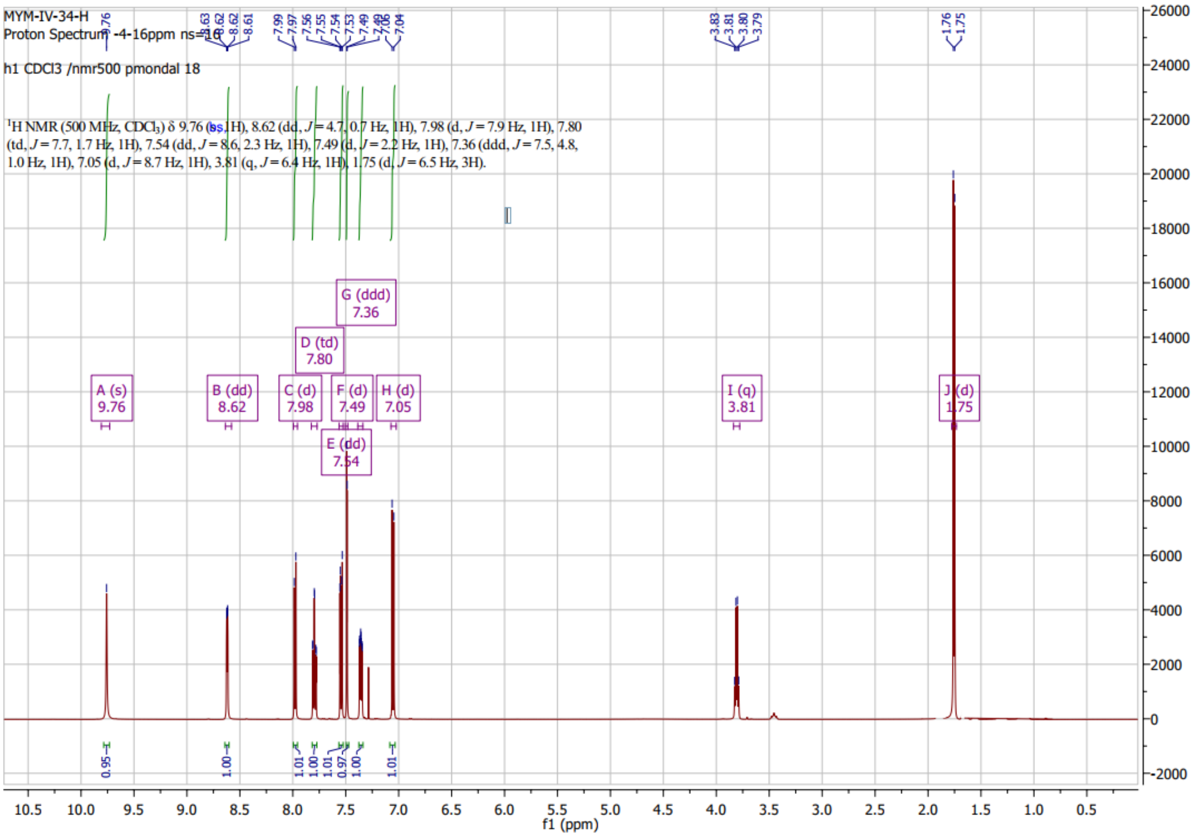

Figure S17. ^{1}HNMR spectrum of compound **14**

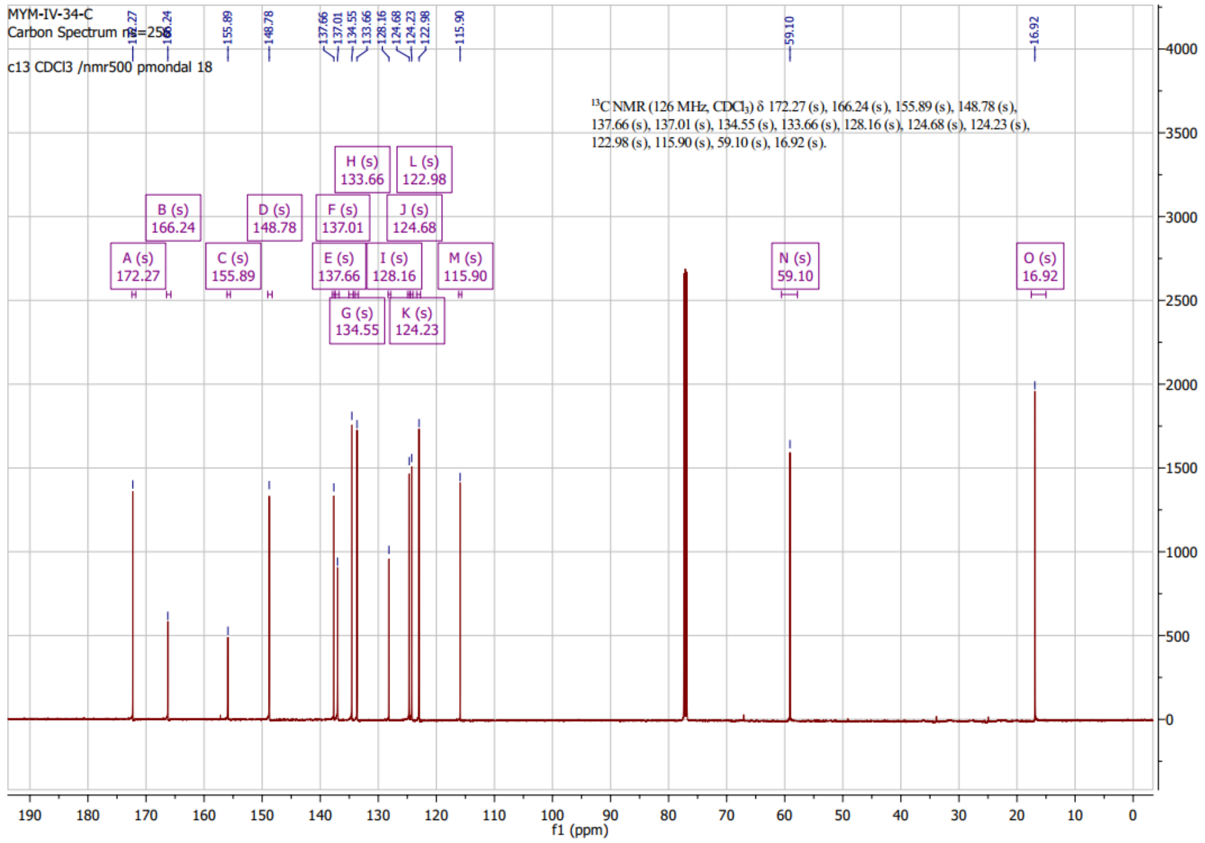

Figure S18. ^{13}CNMR spectrum of compound **14**

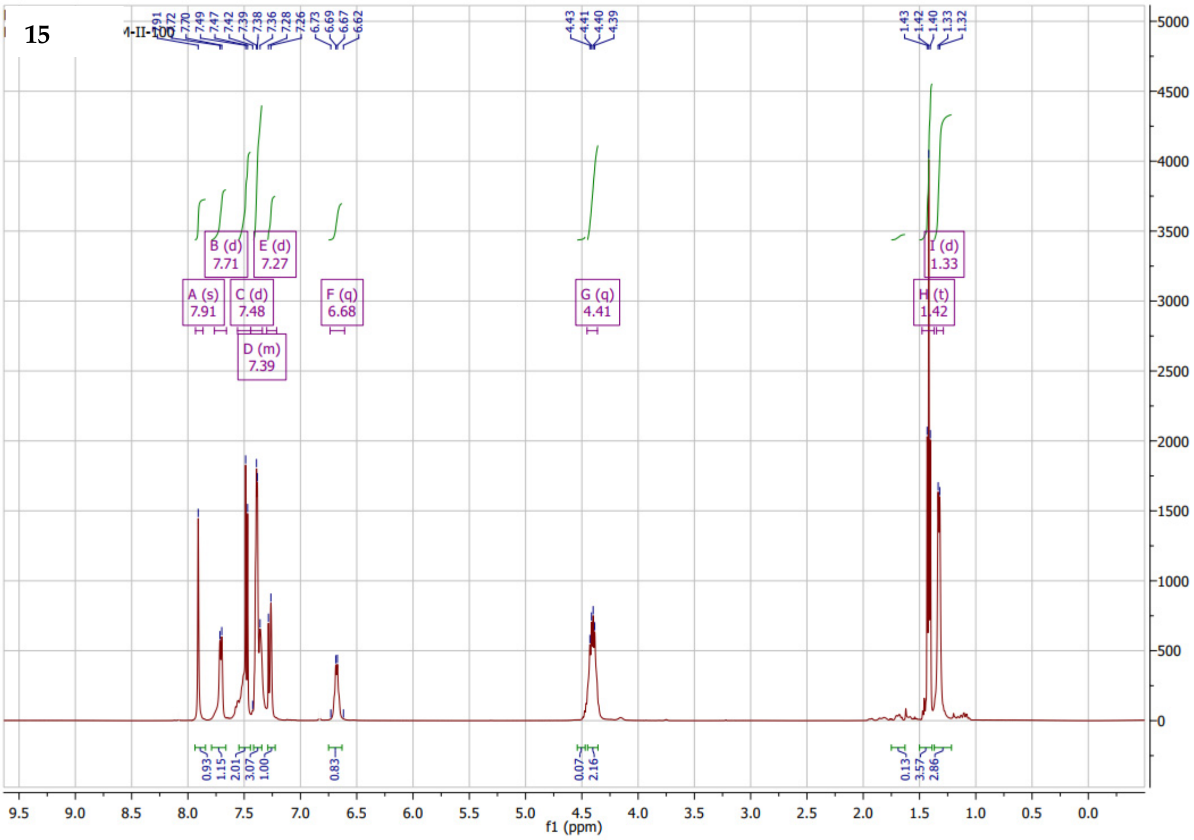

Figure S19. ^{1}HNMR spectrum of compound **15**

15

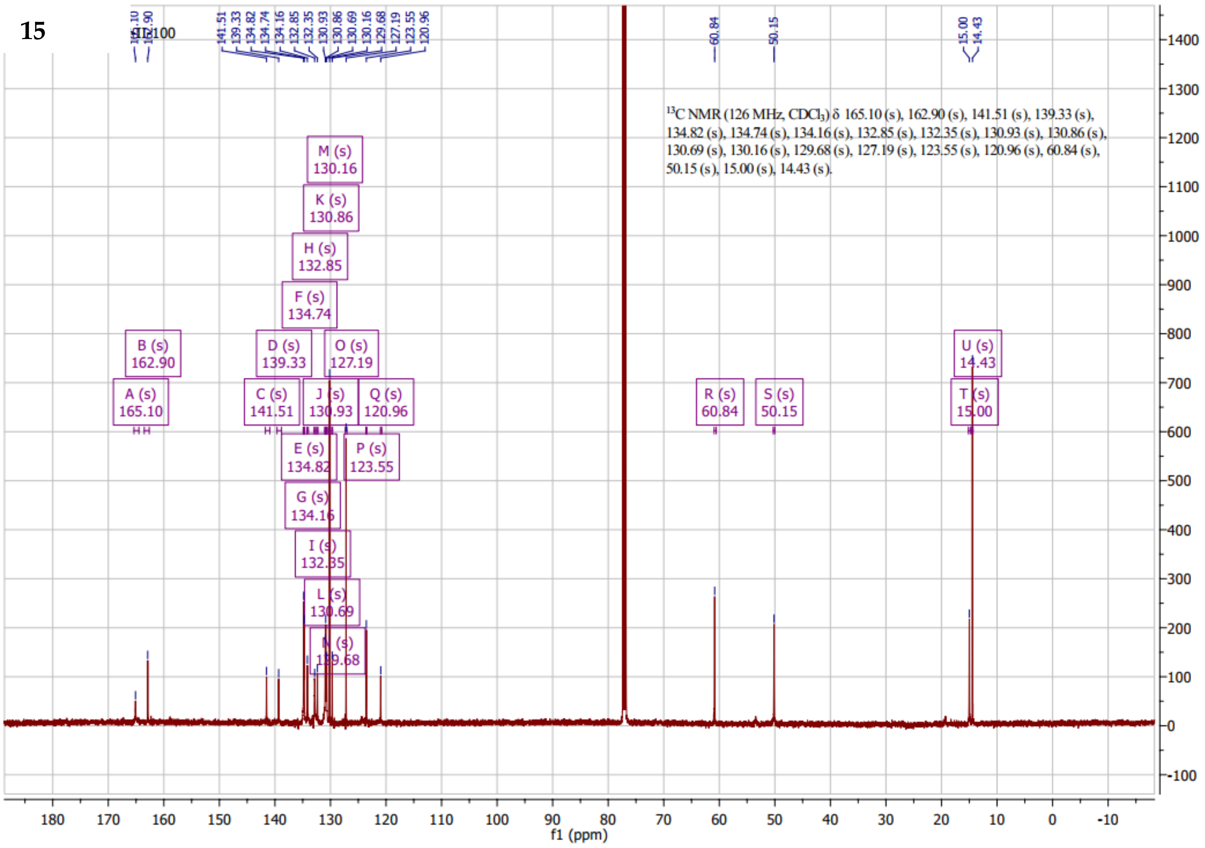

Figure S20. ^{13}CNMR spectrum of compound **15**

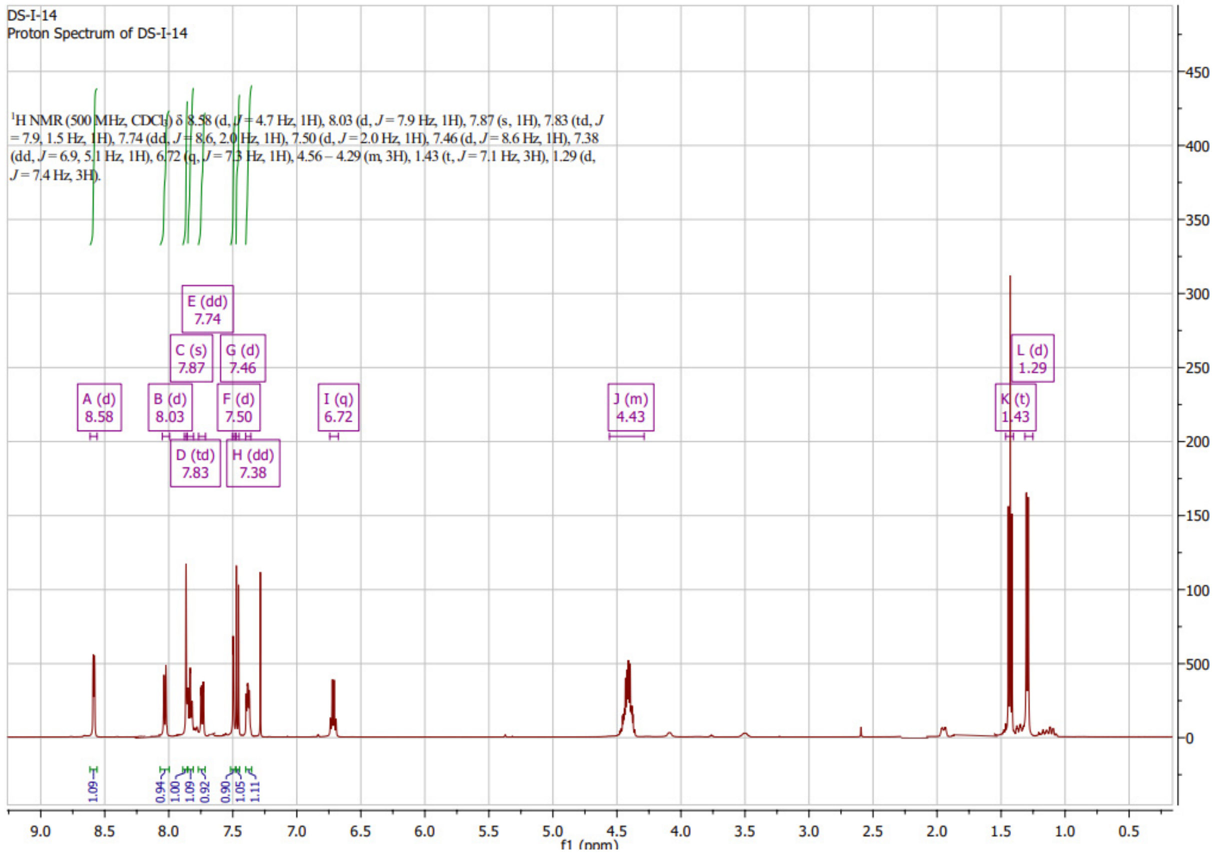

Figure S21. ^{1}HNMR spectrum of compound **16**

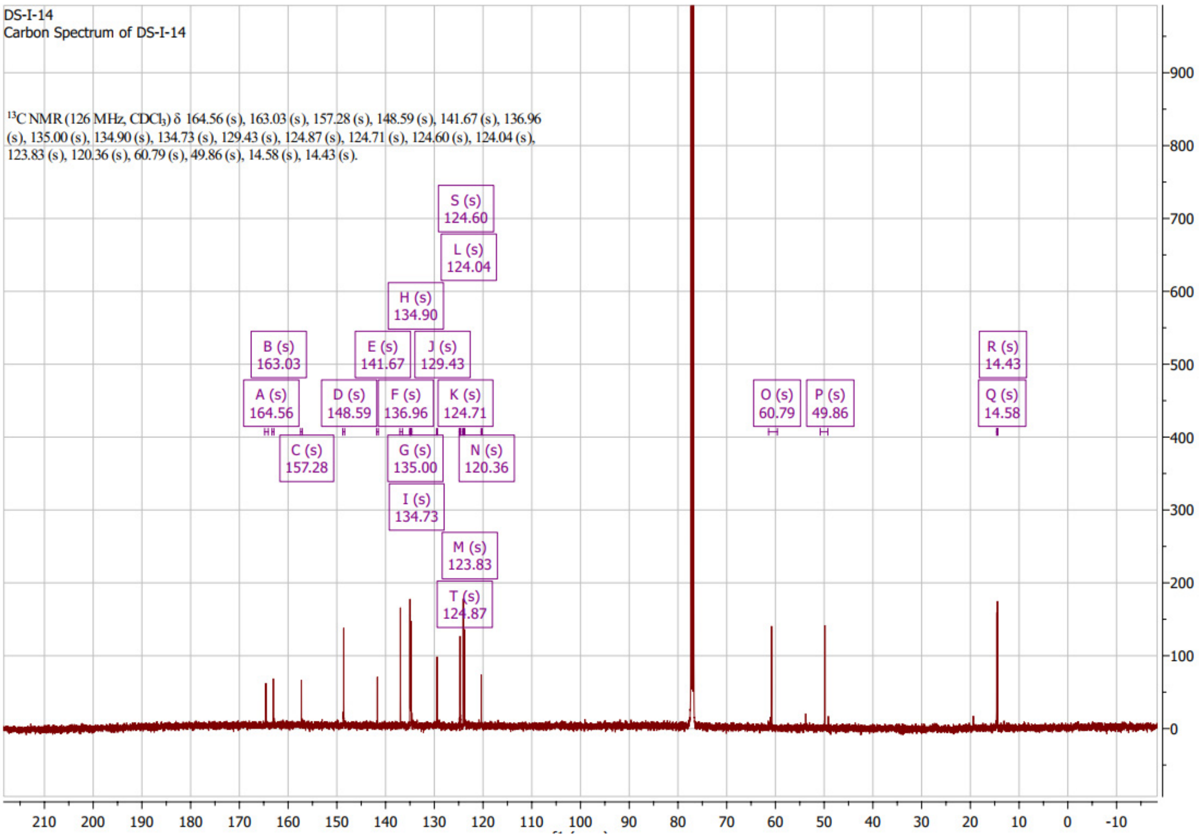

Figure S22. ^{13}CNMR spectrum of compound **16**

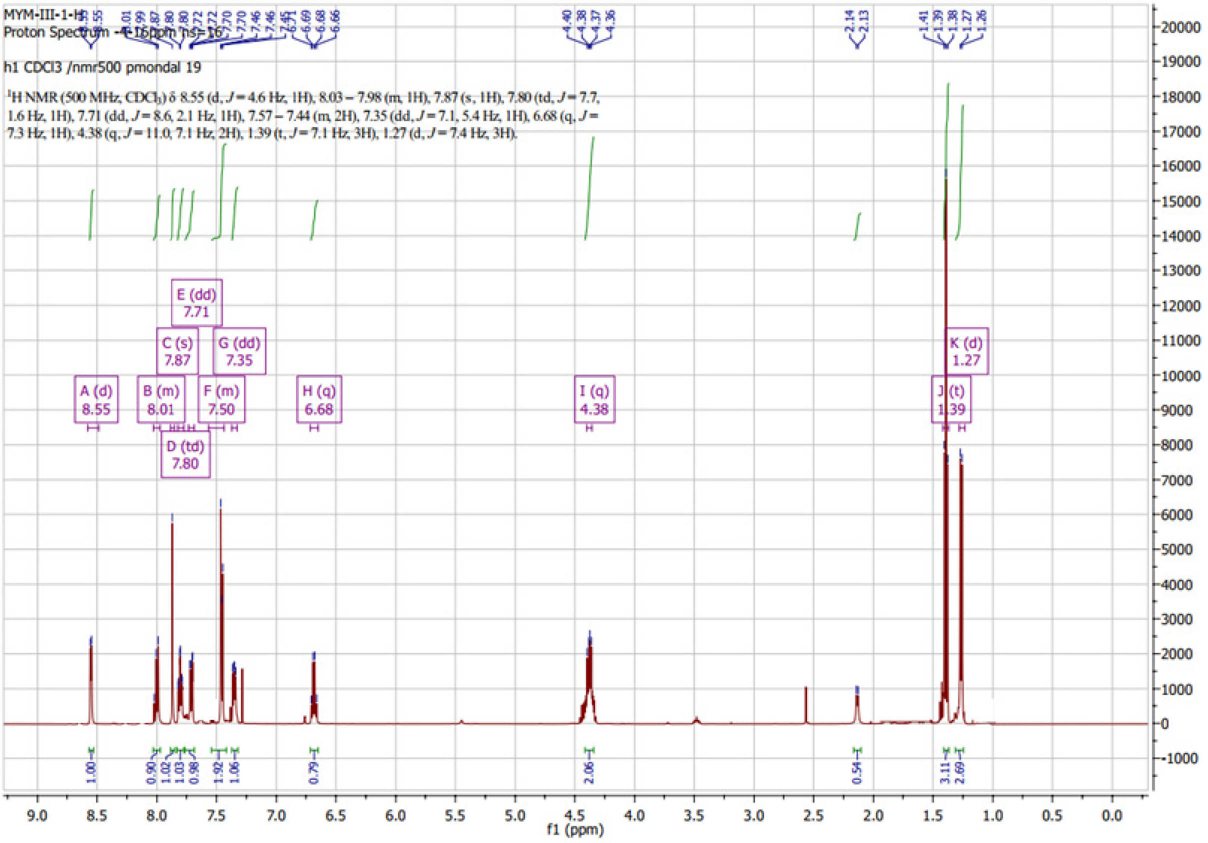

Figure S23. ¹HNMR spectrum of compound **17**

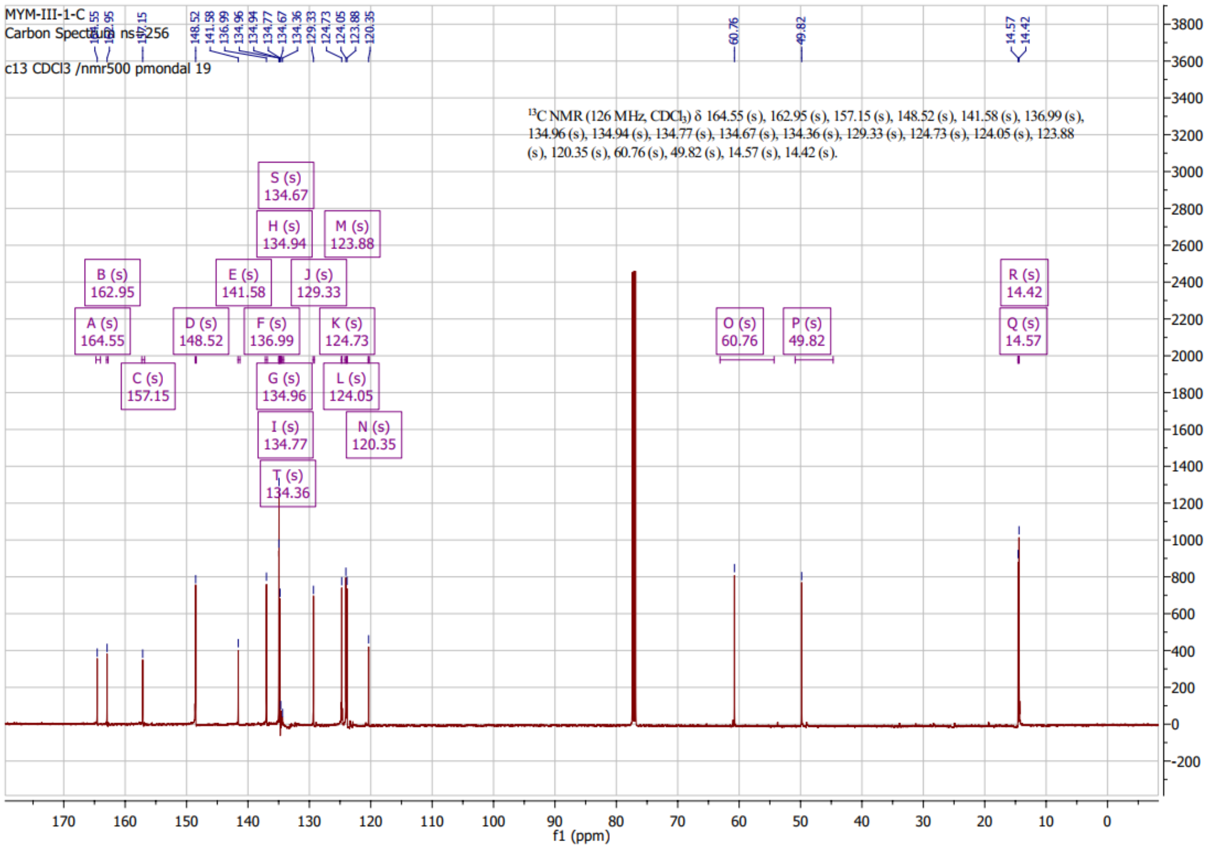

Figure S24. ^{13}CNMR spectrum of compound **17**

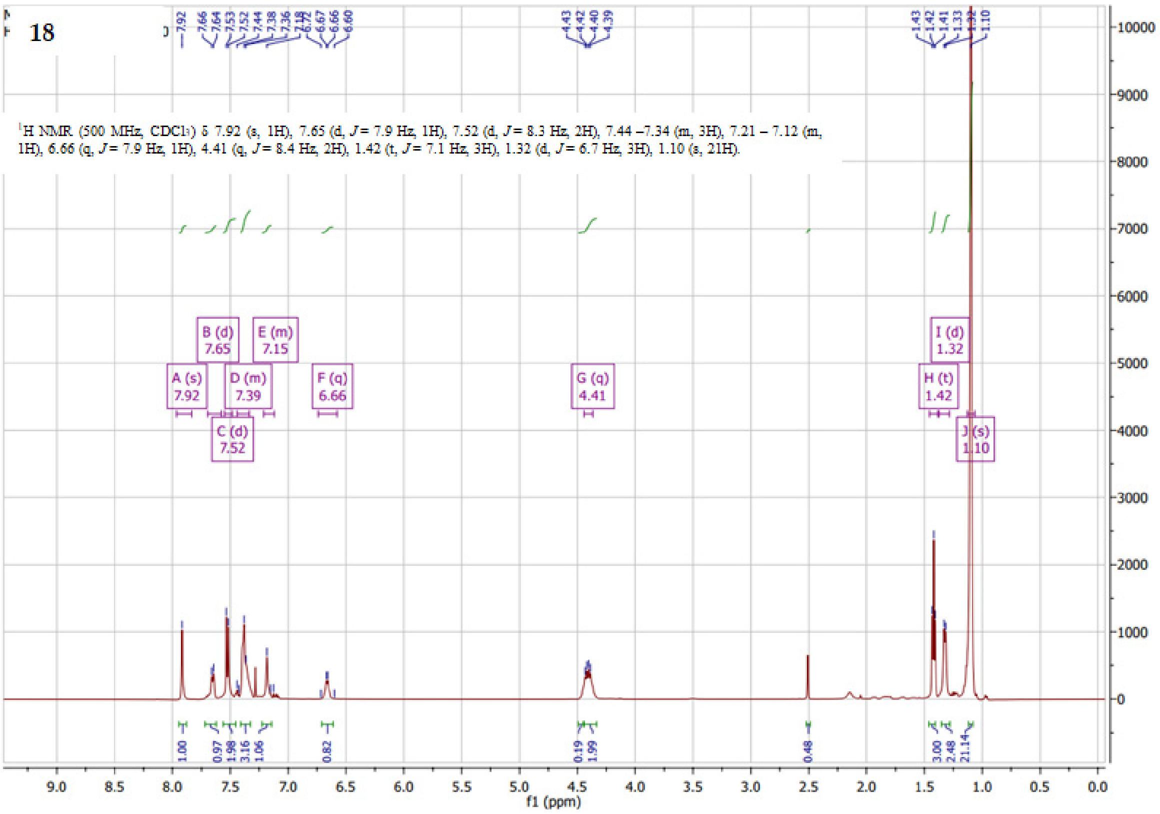

Figure S25. ^{1}HNMR spectrum of compound **18**

18

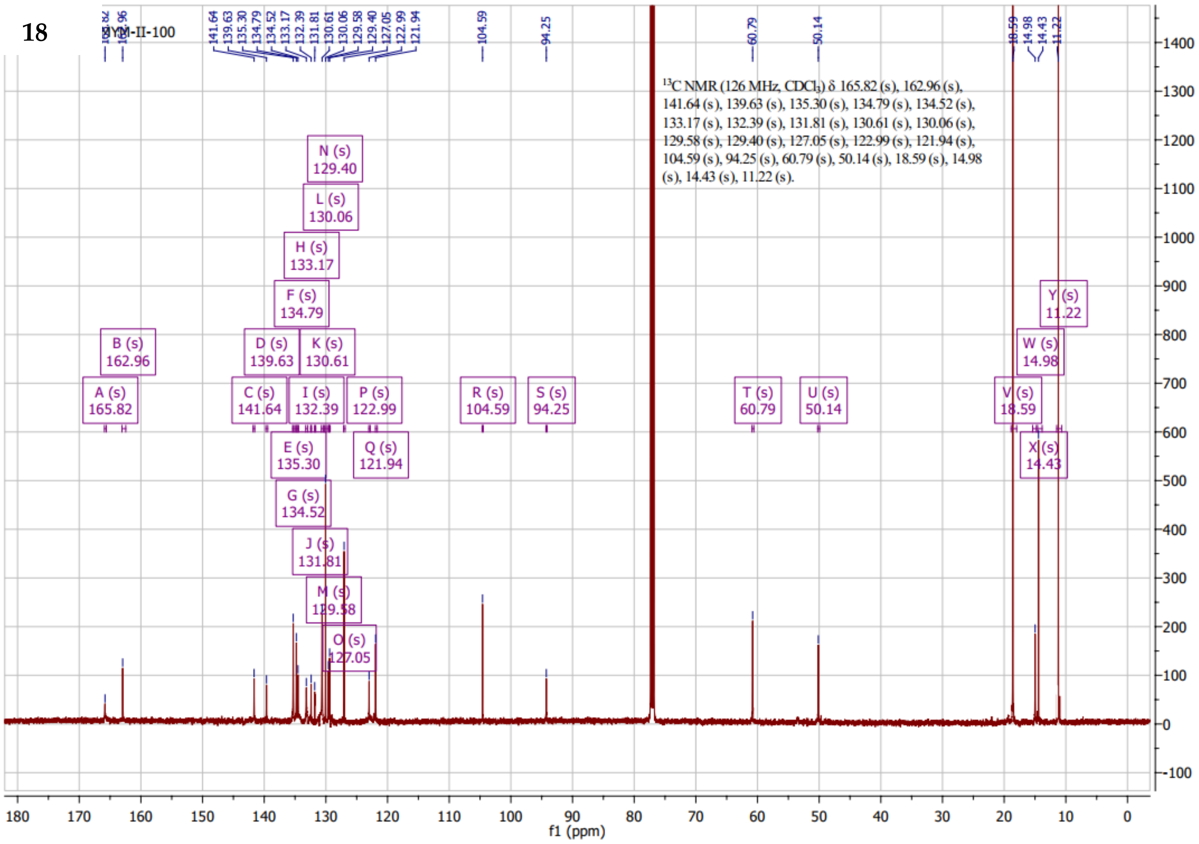

Figure S26. ^{13}CNMR spectrum of compound **18**

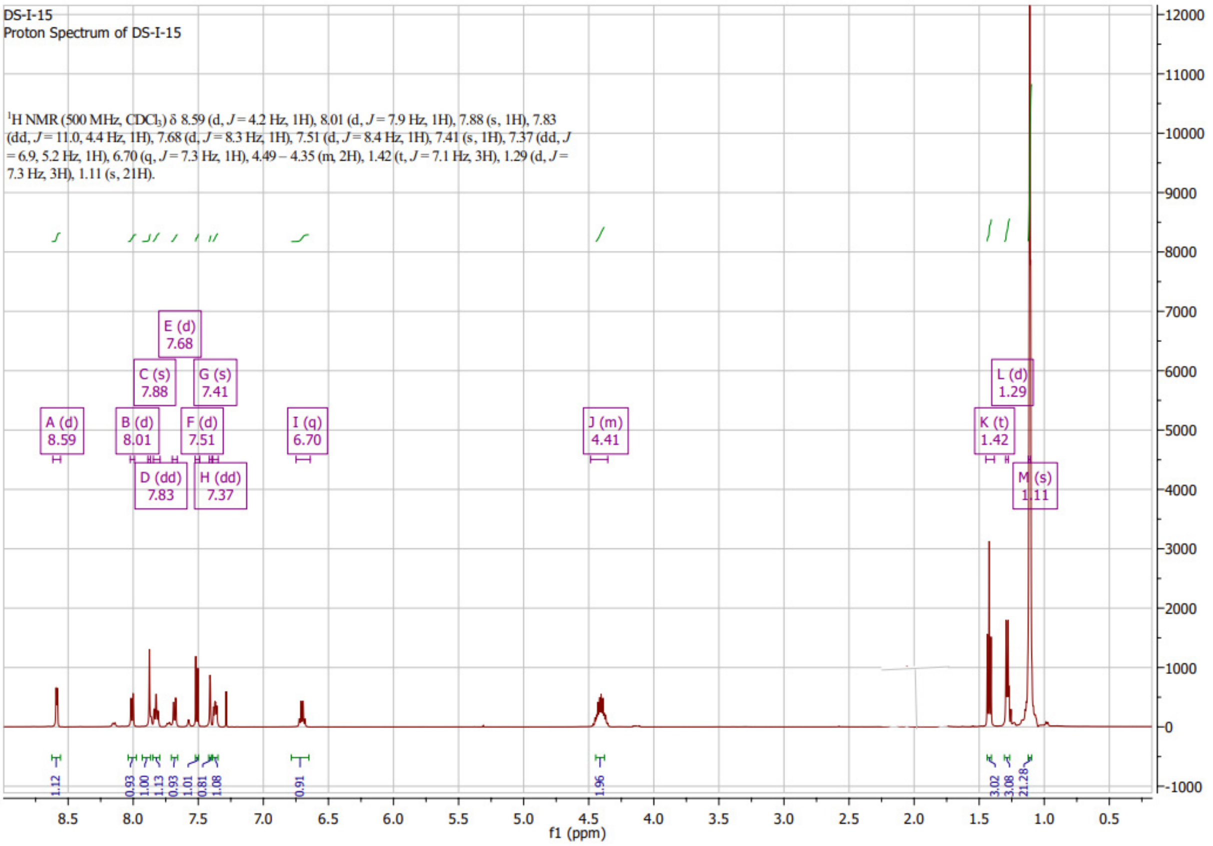

Figure S27. 1HNMR spectrum of compound **19**

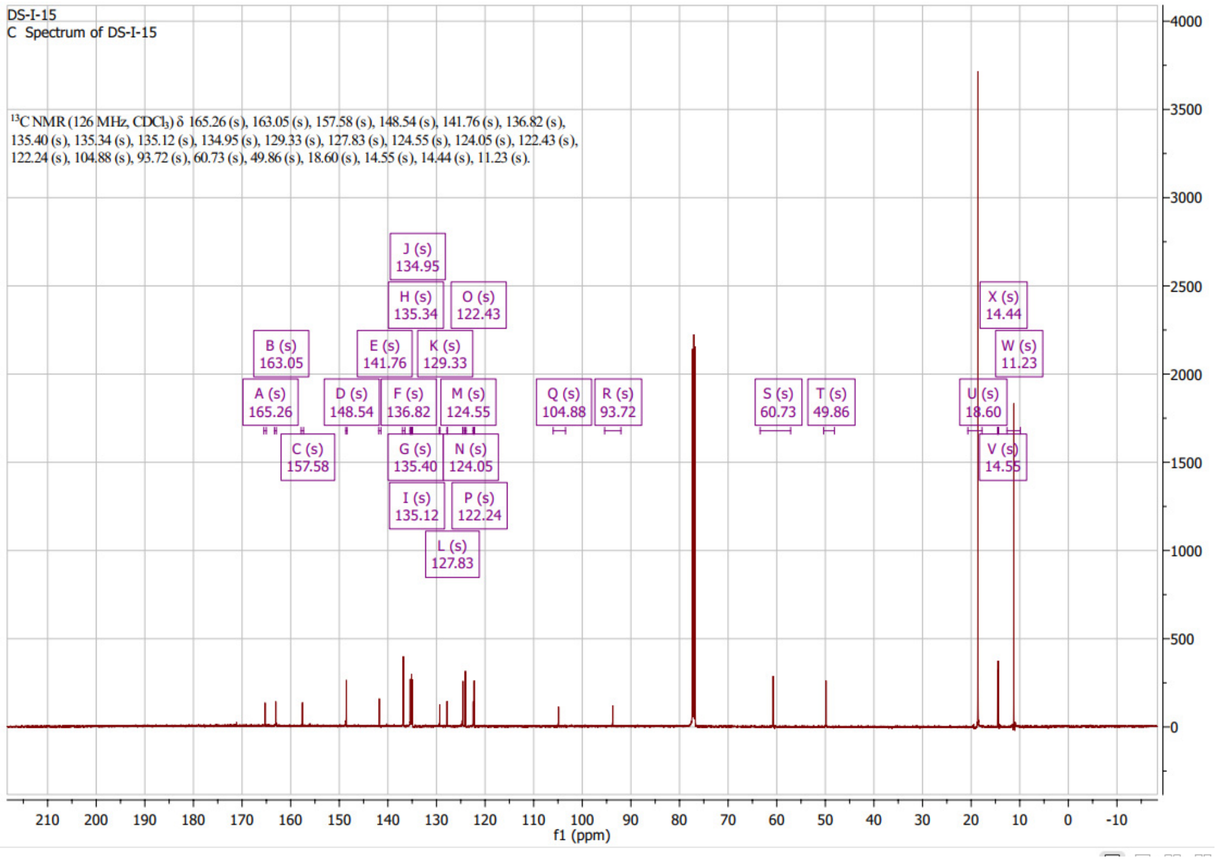

Figure S28. ^{13}CNMR spectrum of compound **19**

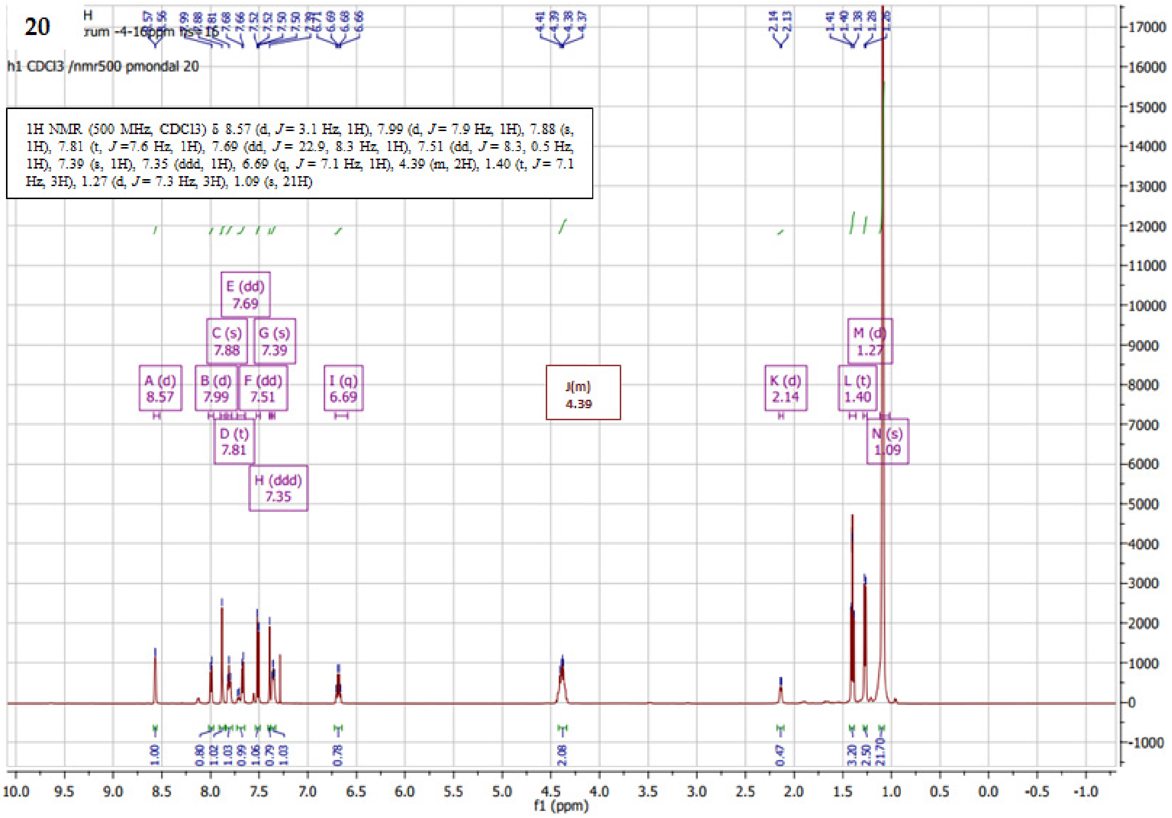

Figure S29. ^{1}HNMR spectrum of compound **20**

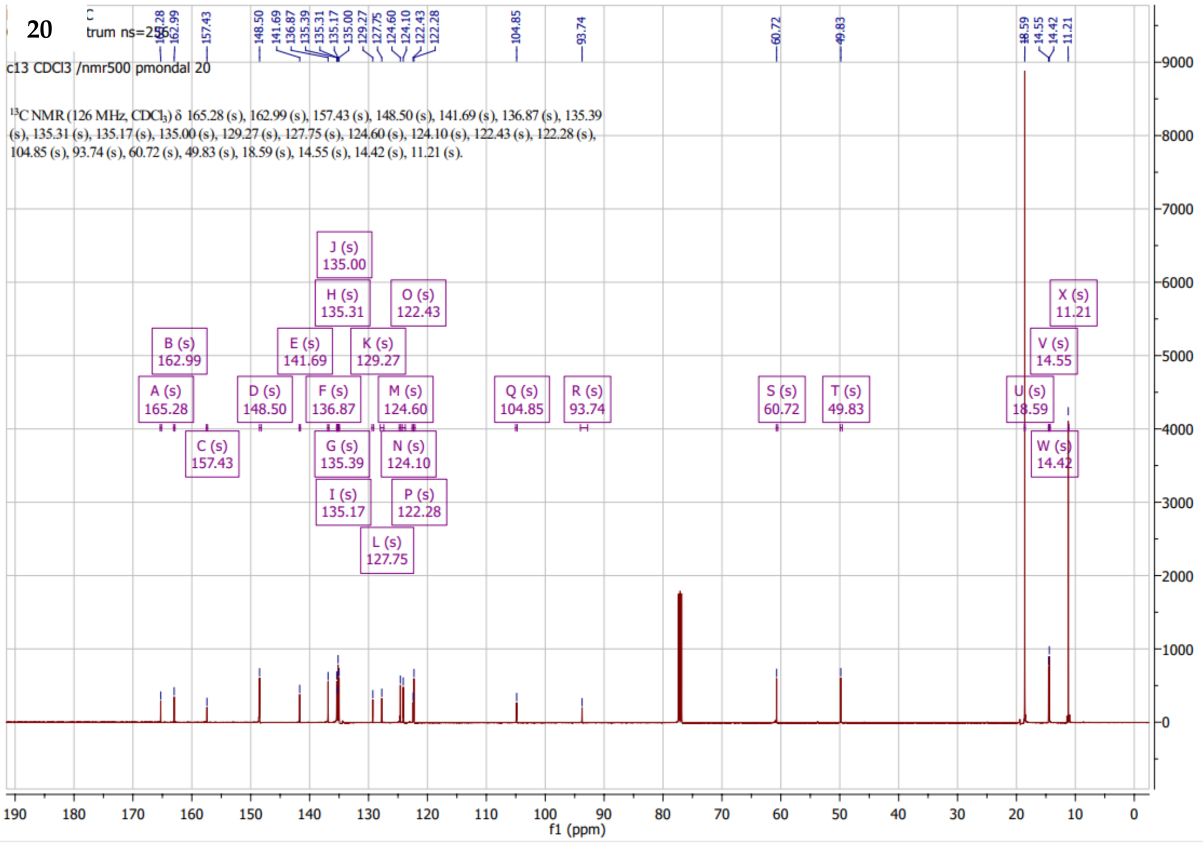

Figure S30. ^{13}CNMR spectrum of compound **20**

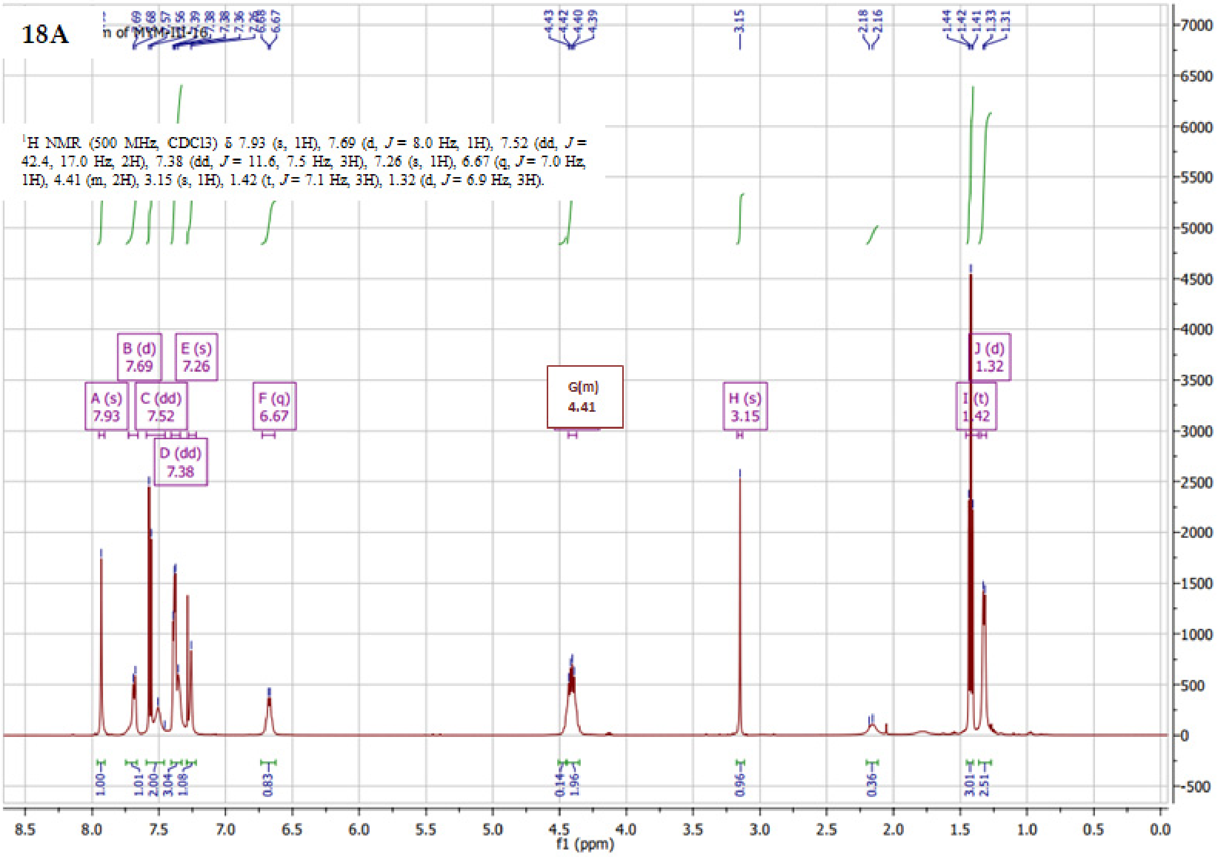

Figure S31. ^{1}HNMR spectrum of compound **18A**

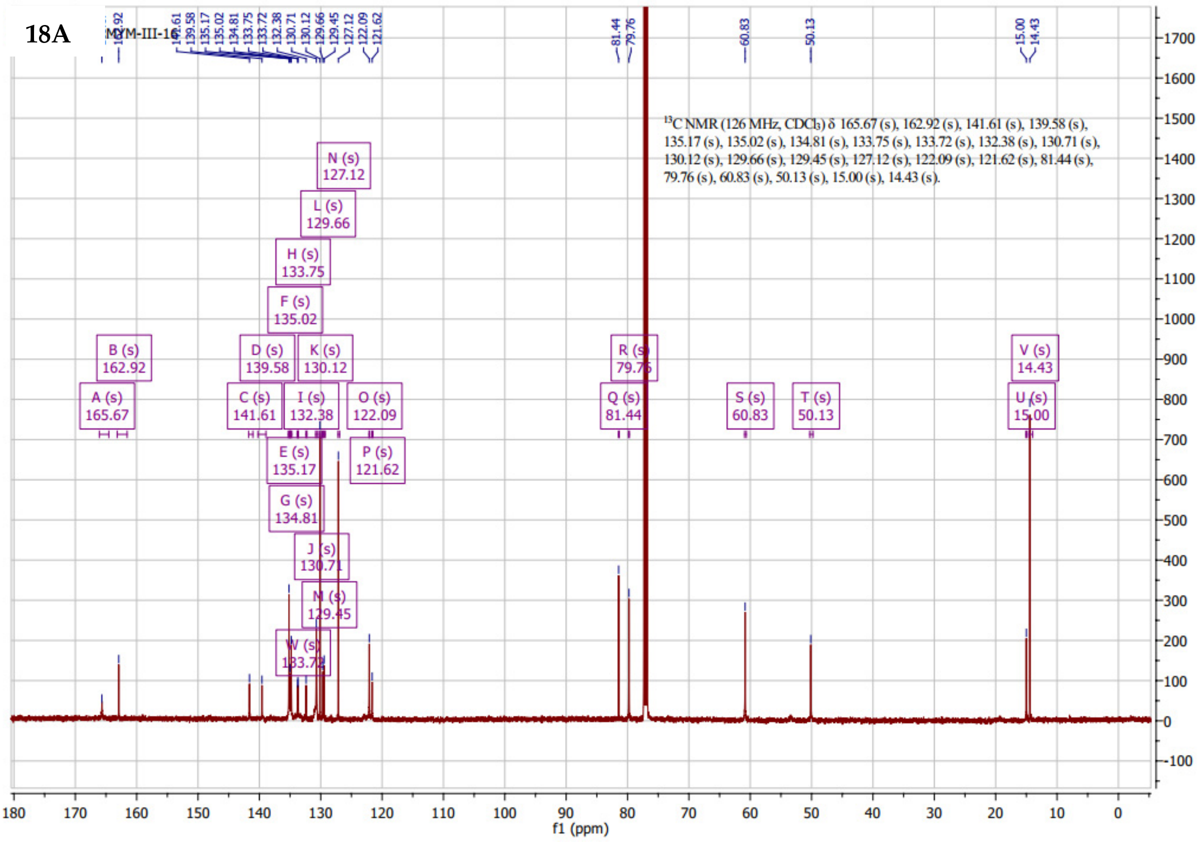

Figure S32. ^{13}CNMR spectrum of compound **18A**

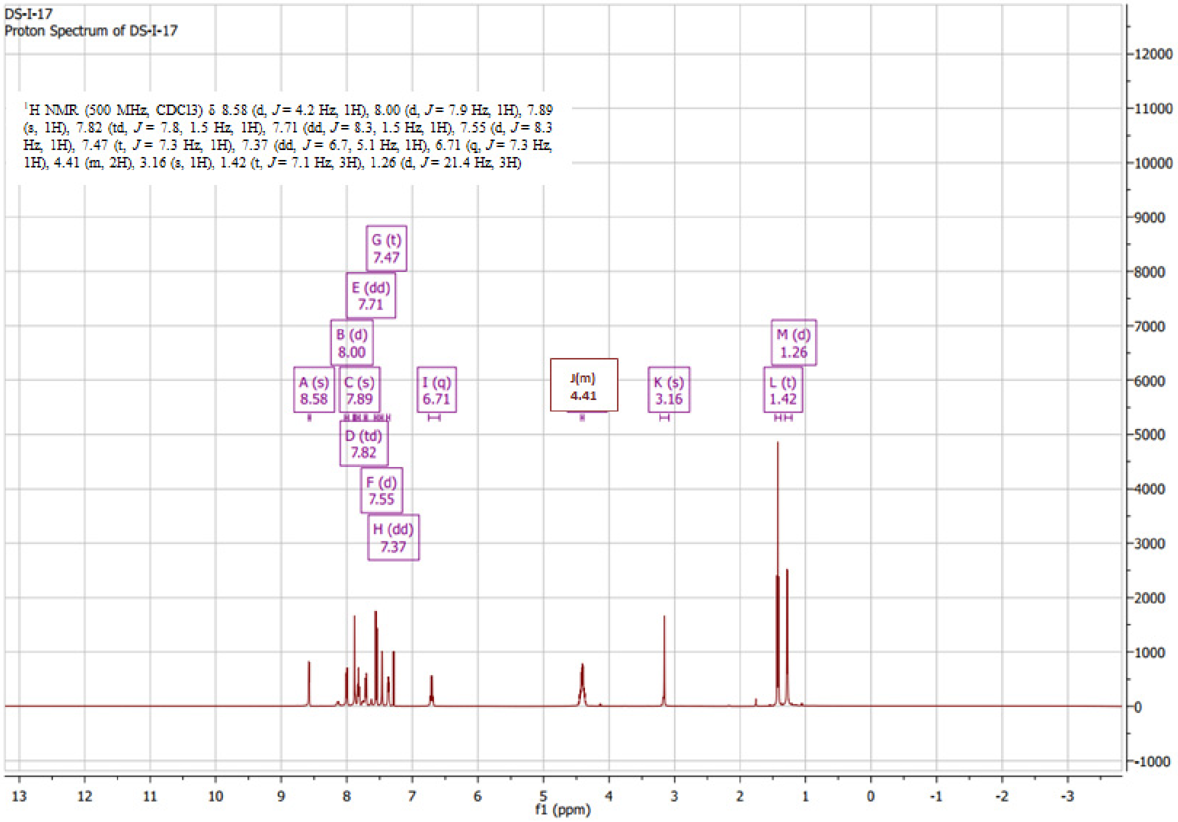

Figure S33. 1HNMR spectrum of compound **19A**

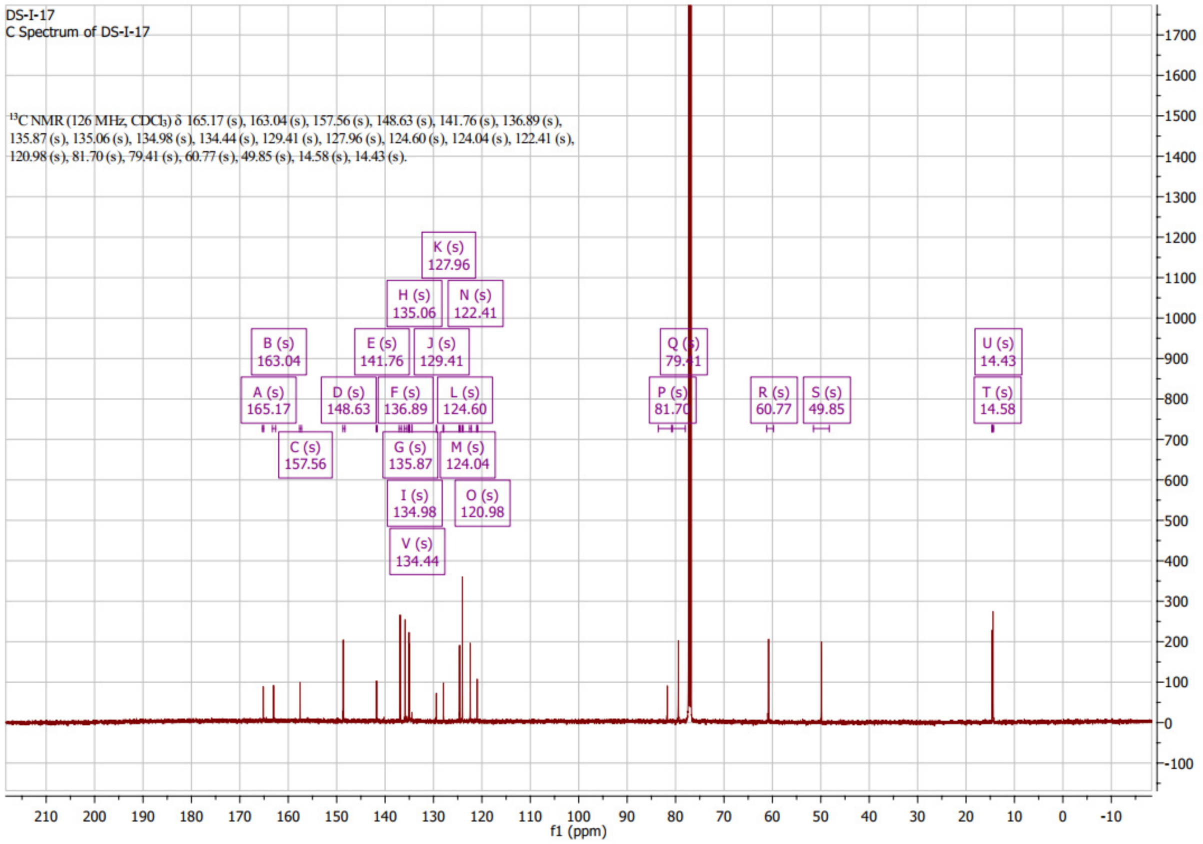

Figure S34. ^{13}CNMR spectrum of compound **19A**

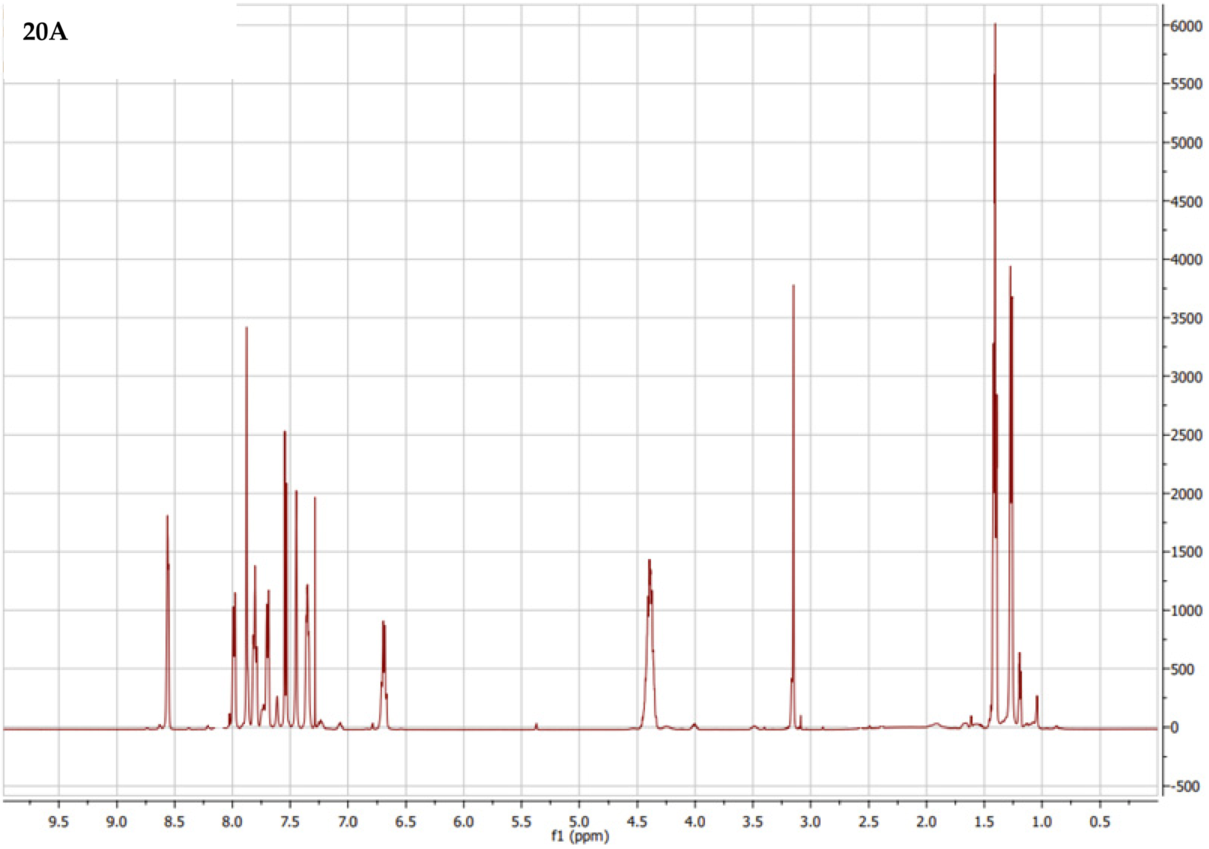

Figure S35. ^{1}HNMR spectrum of compound **20A**

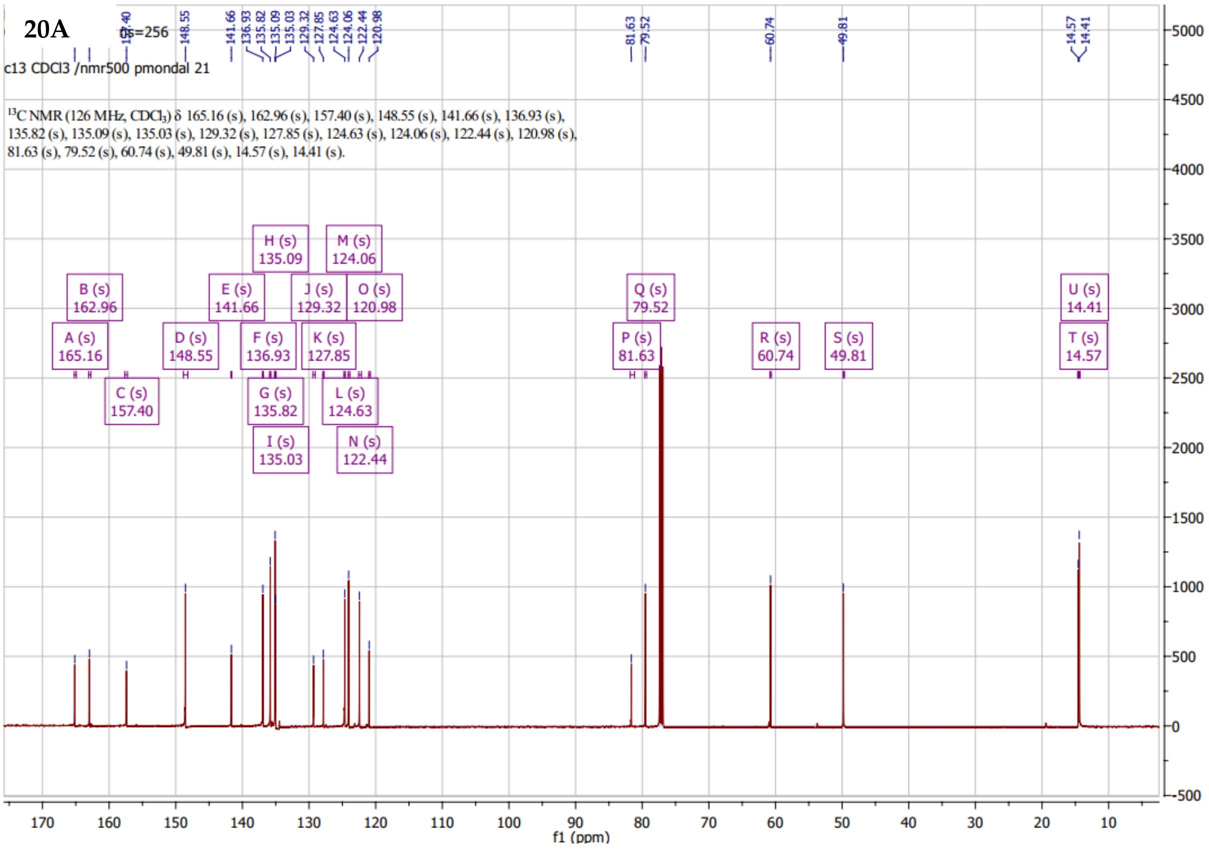

Figure S36. ^{13}CNMR spectrum of compound **20A**

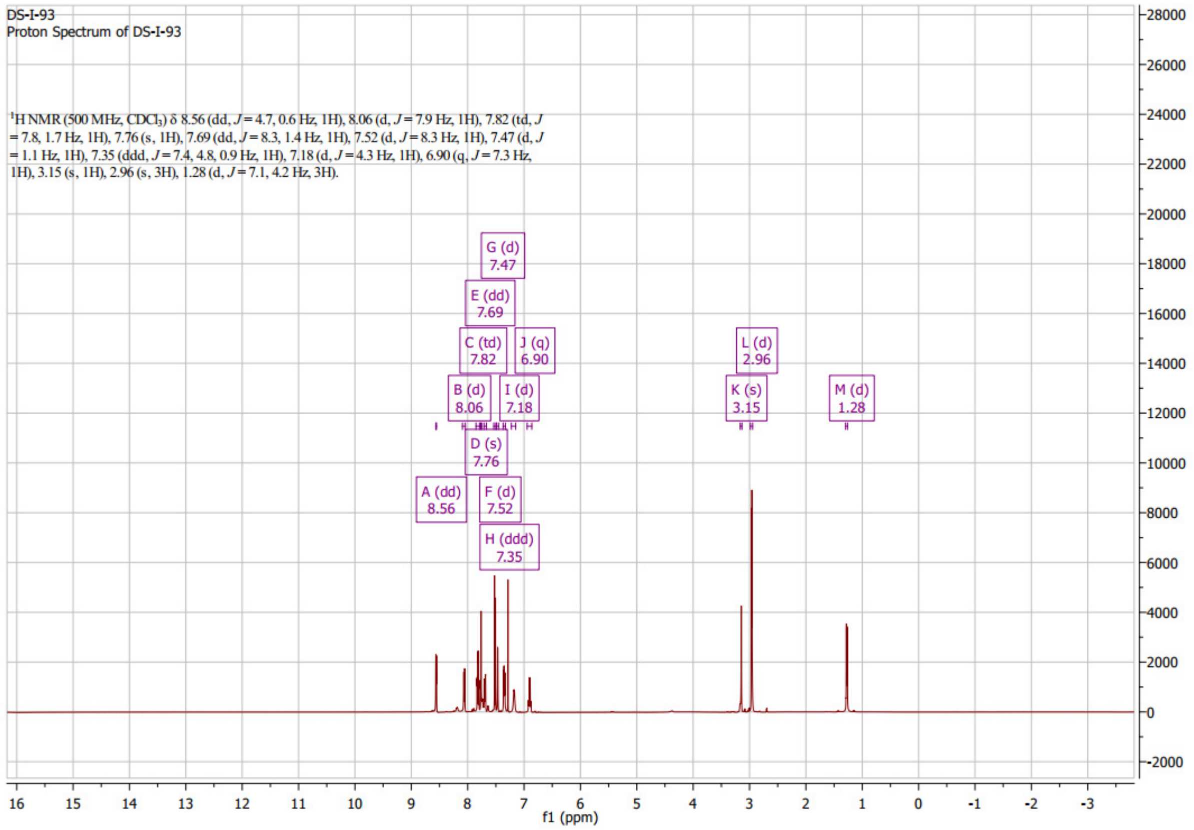

Figure S37. 1HNMR spectrum of compound **22**

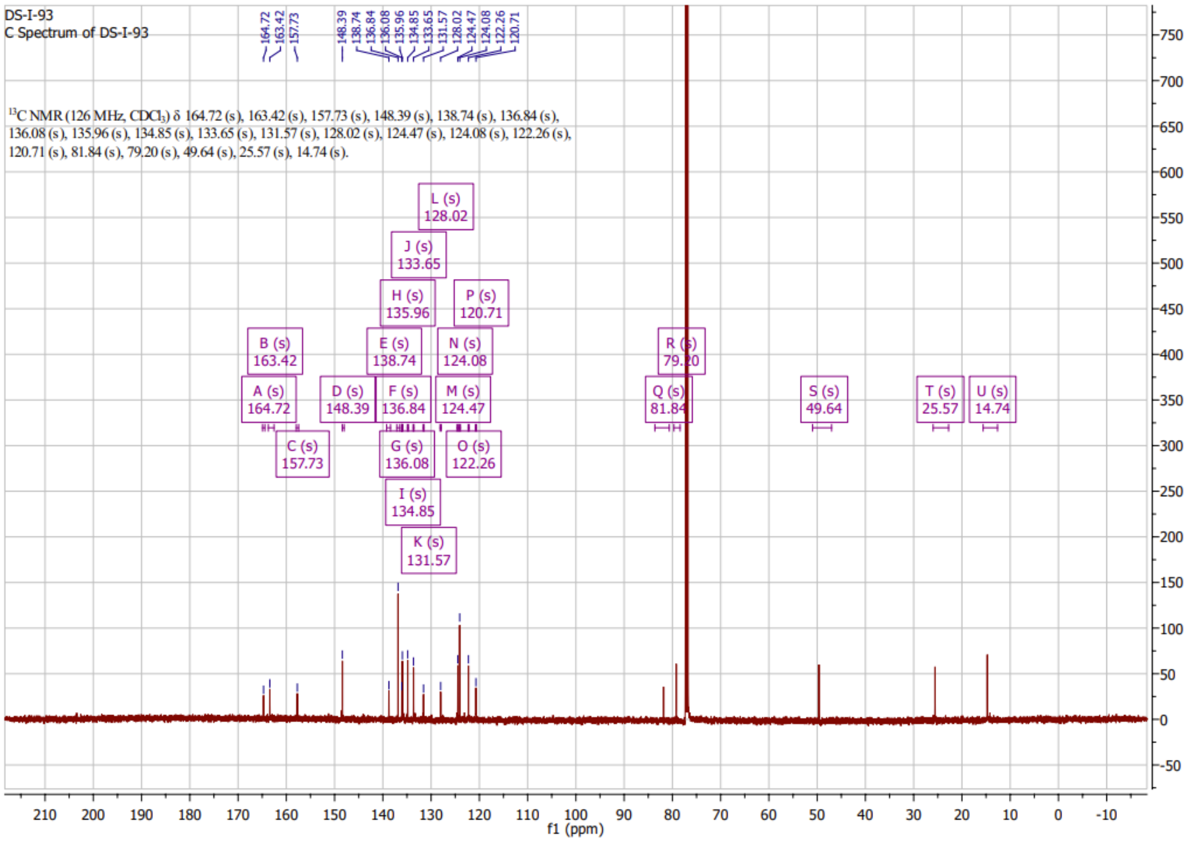

Figure S38. ^{13}CNMR spectrum of compound **22**

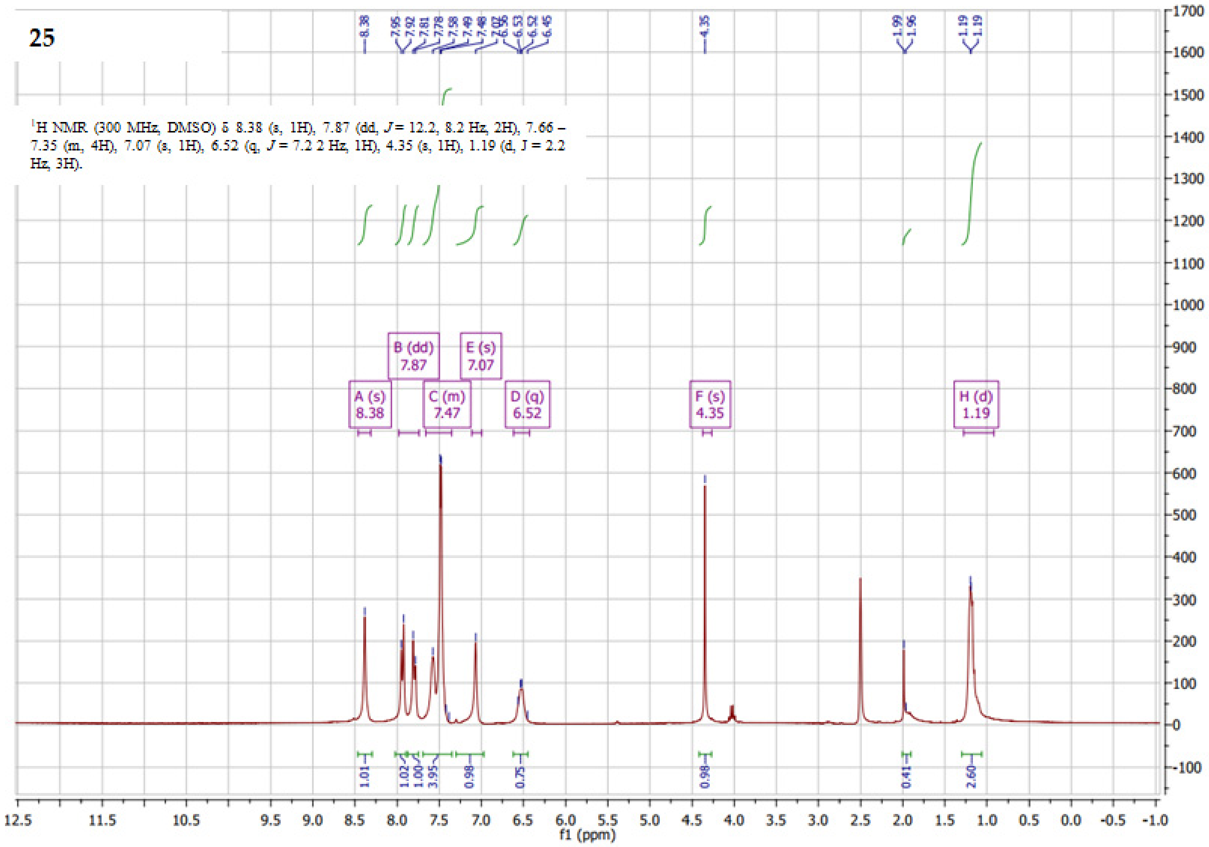

Figure S39. ^{1}HNMR spectrum of compound **25**

25

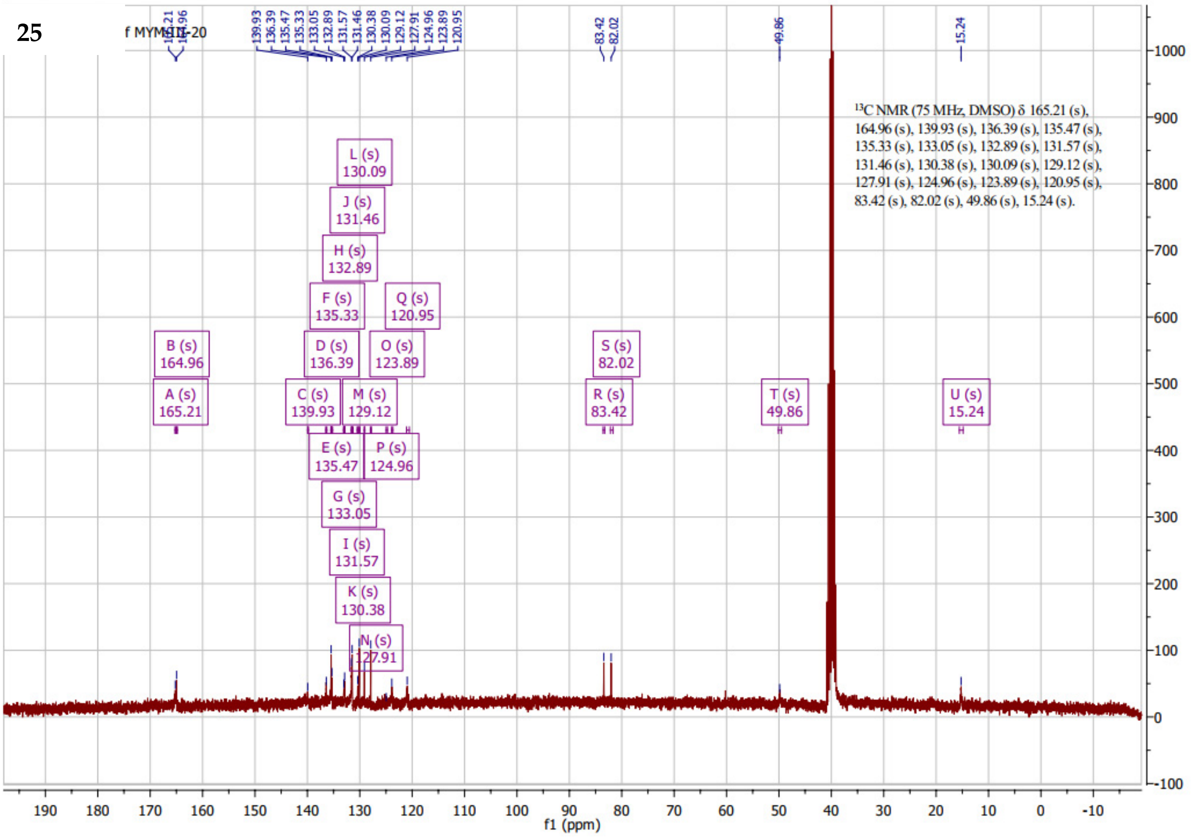

Figure S40. ^{13}CNMR spectrum of compound **25**

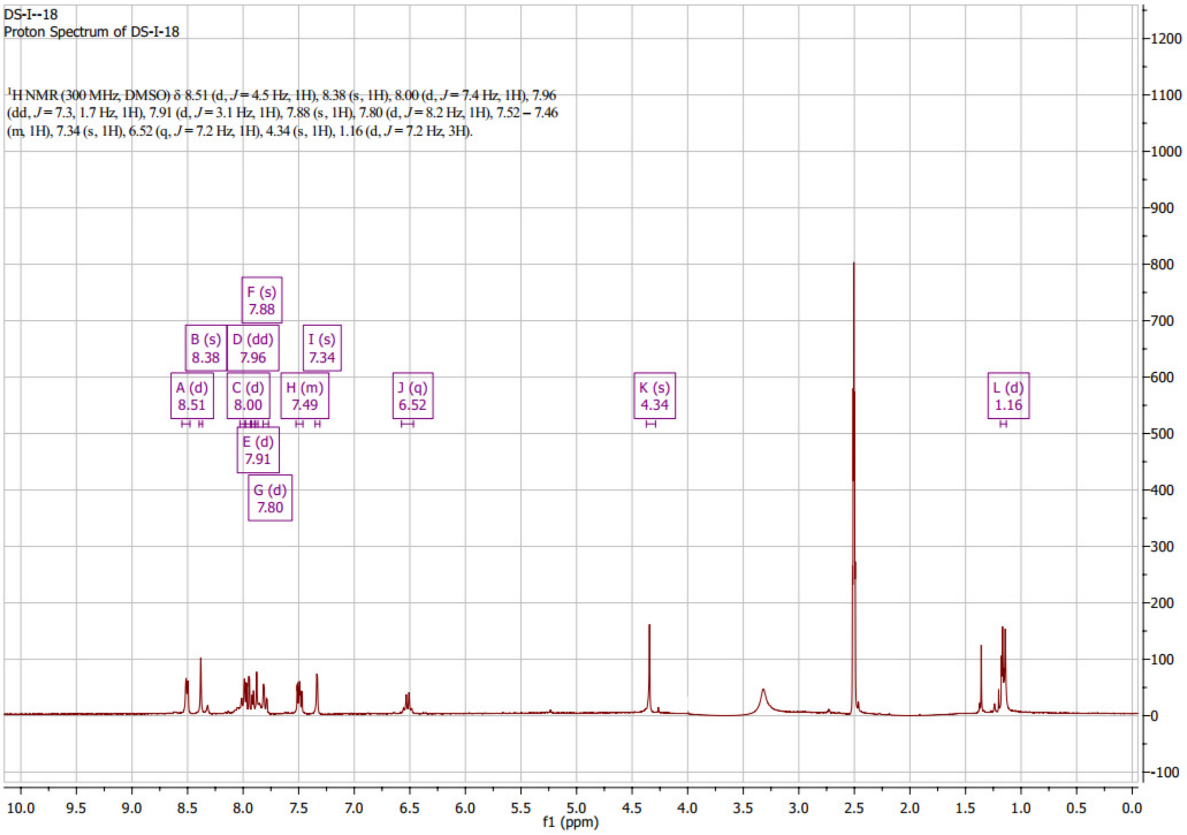

Figure S41. ^{1}HNMR spectrum of compound **26**

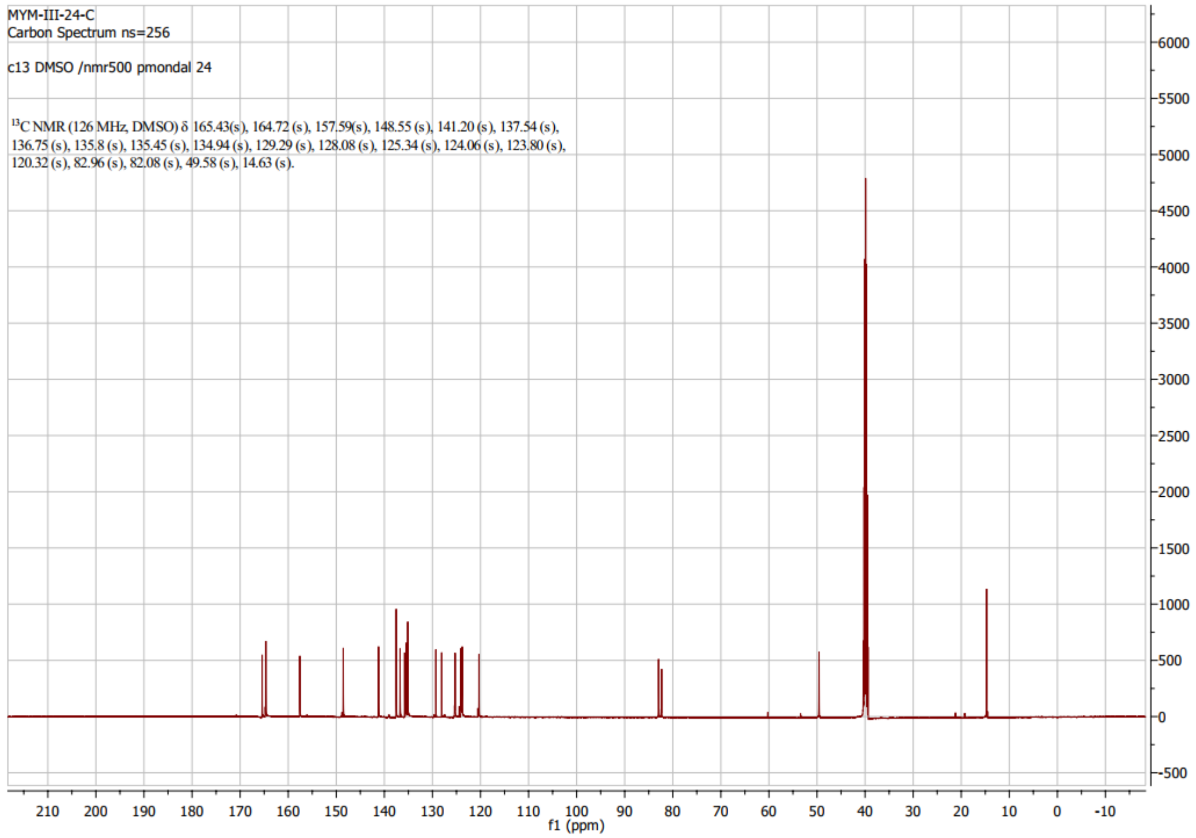

Figure S42. ^{13}CNMR spectrum of compound **26**

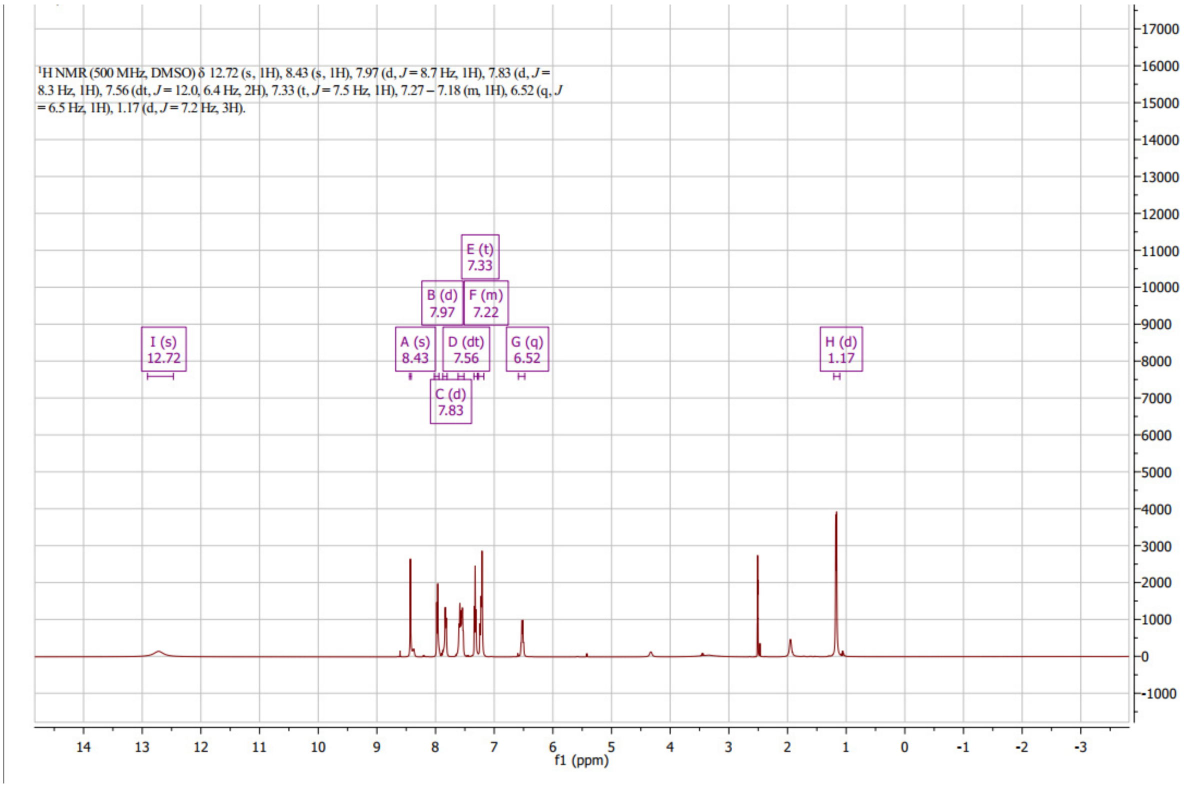

Figure S43. ^{1}HNMR spectrum of compound **28**

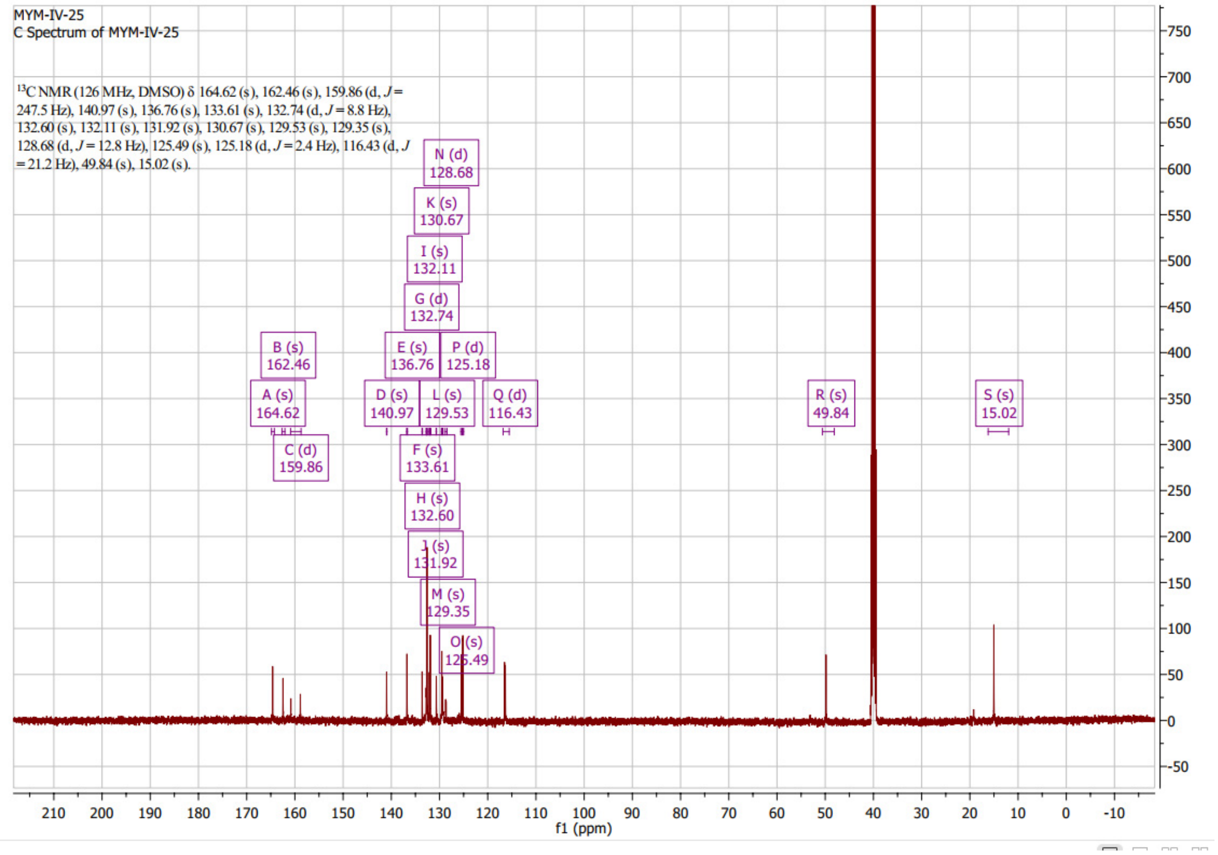

Figure S44. ^{13}CNMR spectrum of compound **28**

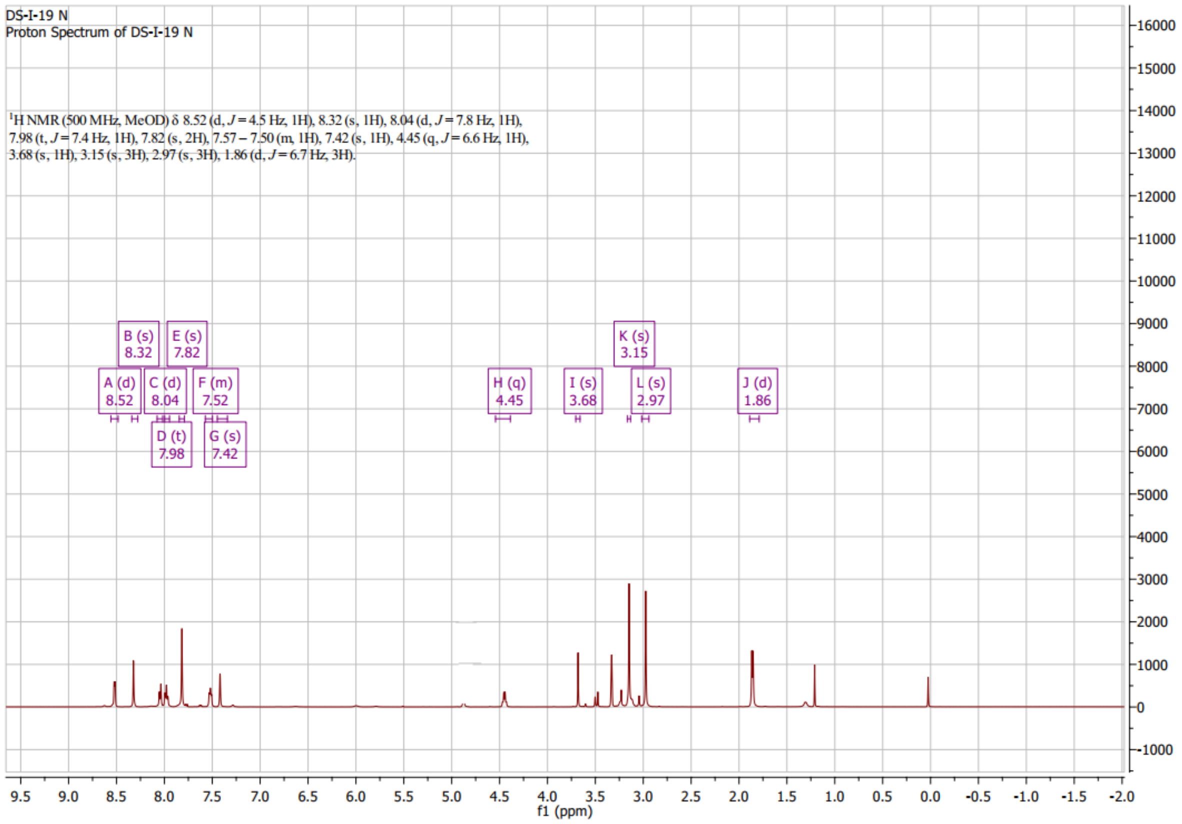

Figure S45. 1HNMR spectrum of compound **30**

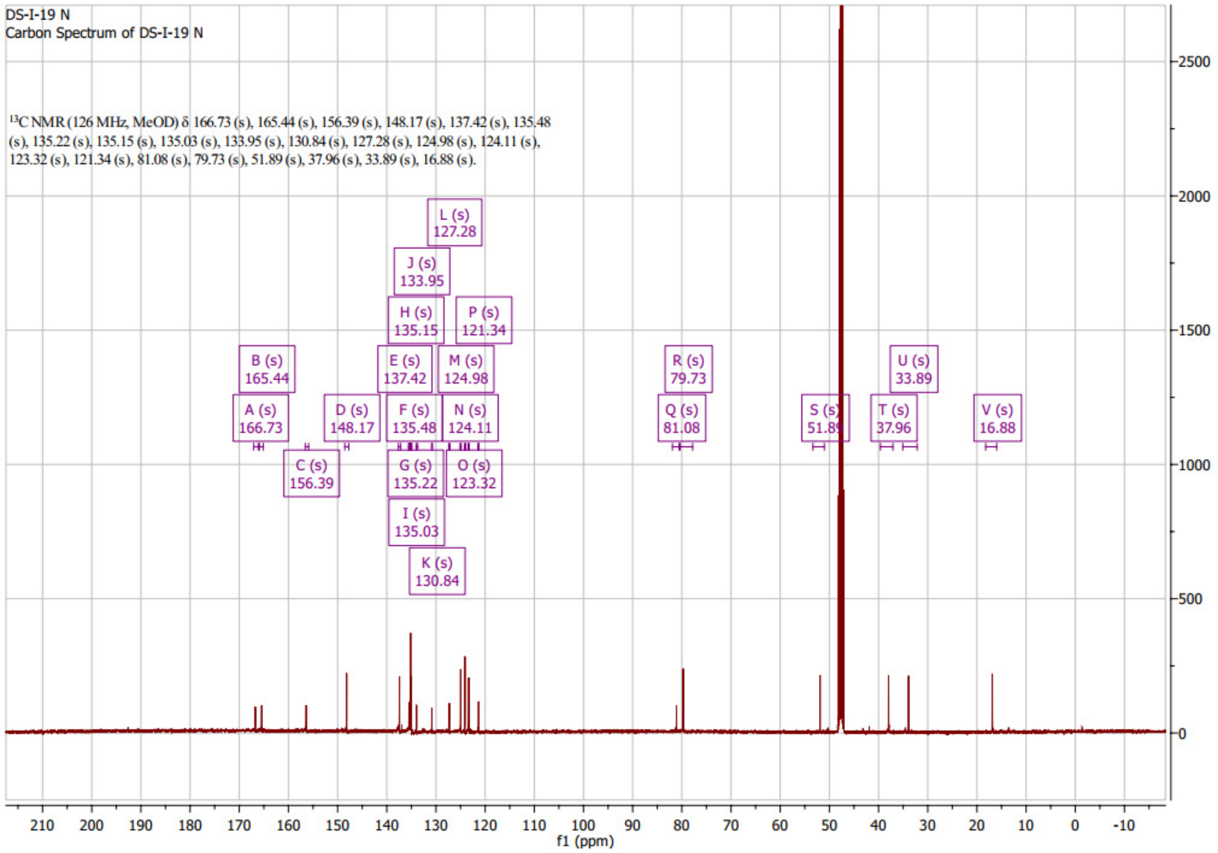

Figure S46. ^{13}CNMR spectrum of compound **30**

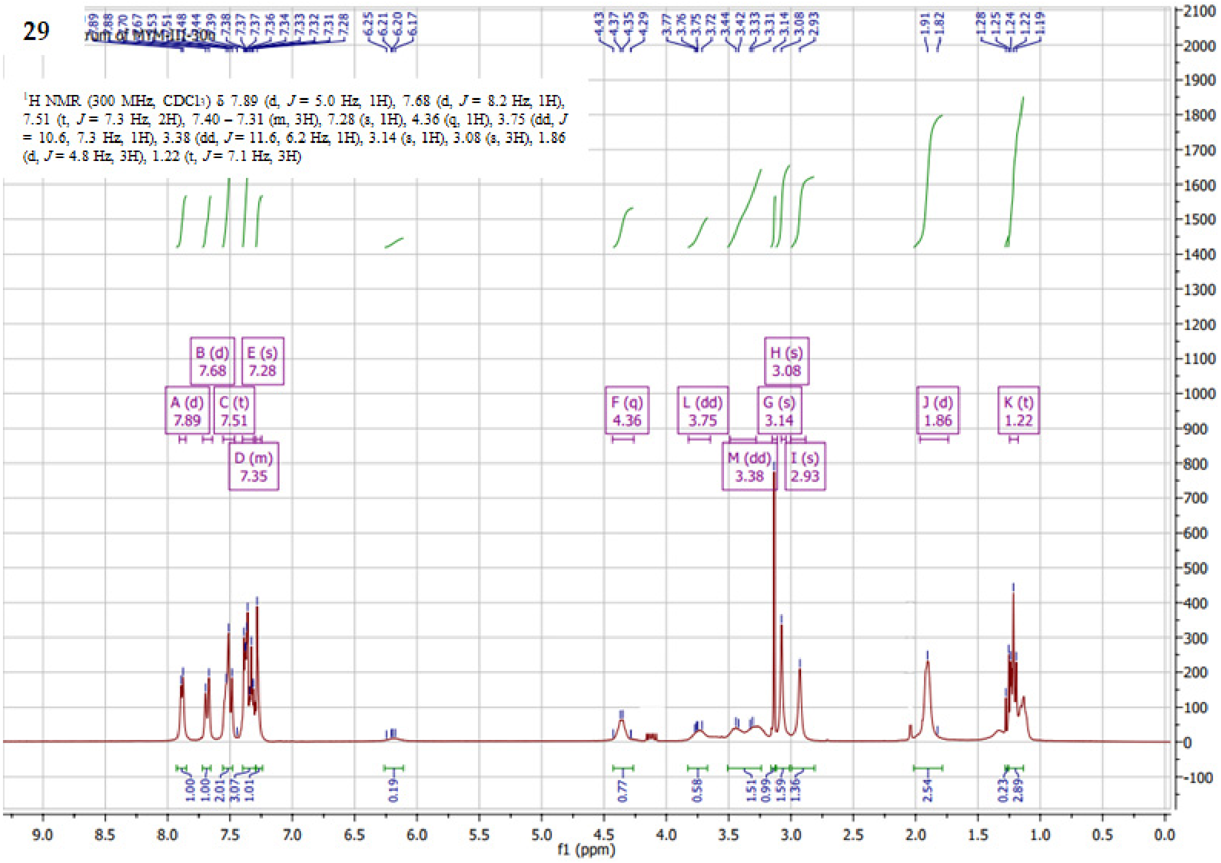

Figure S47. ^{1}HNMR spectrum of compound **29**

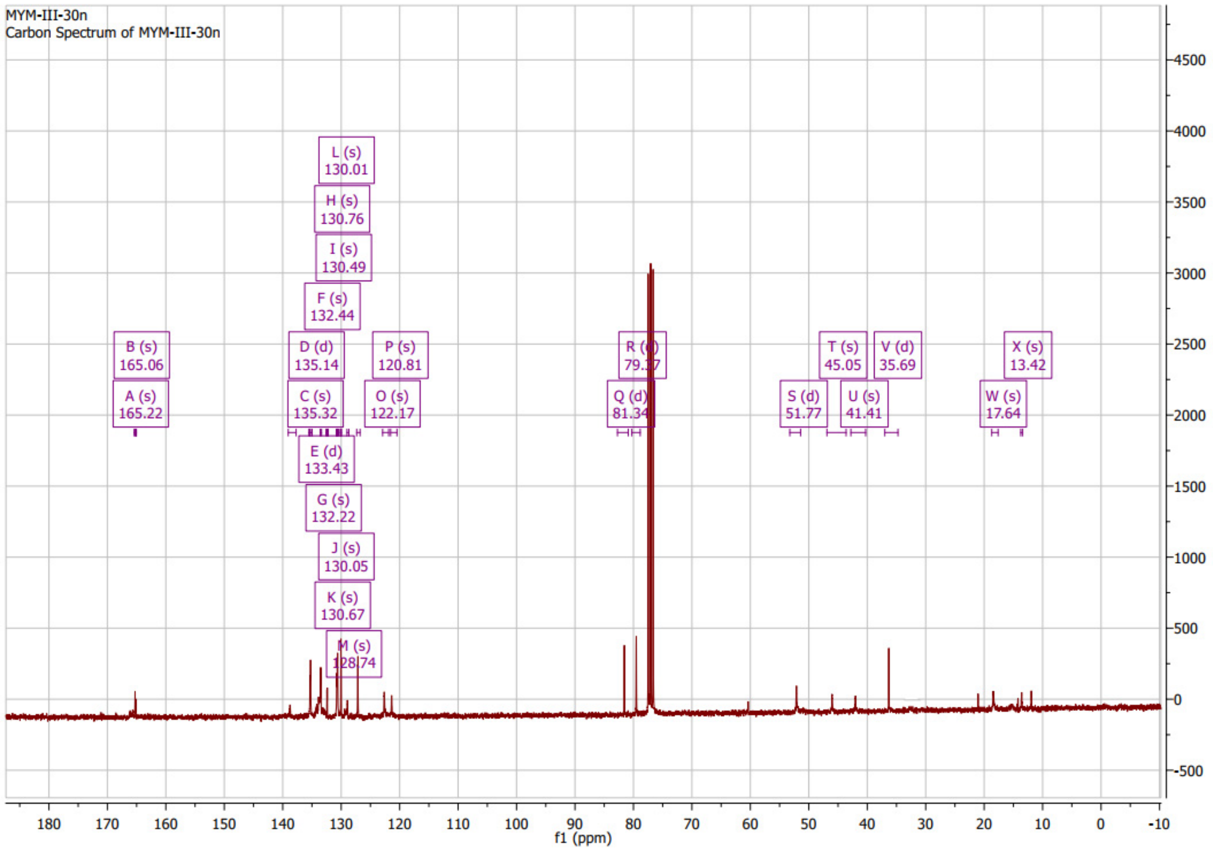

Figure S48. ^{13}CNMR spectrum of compound **29**

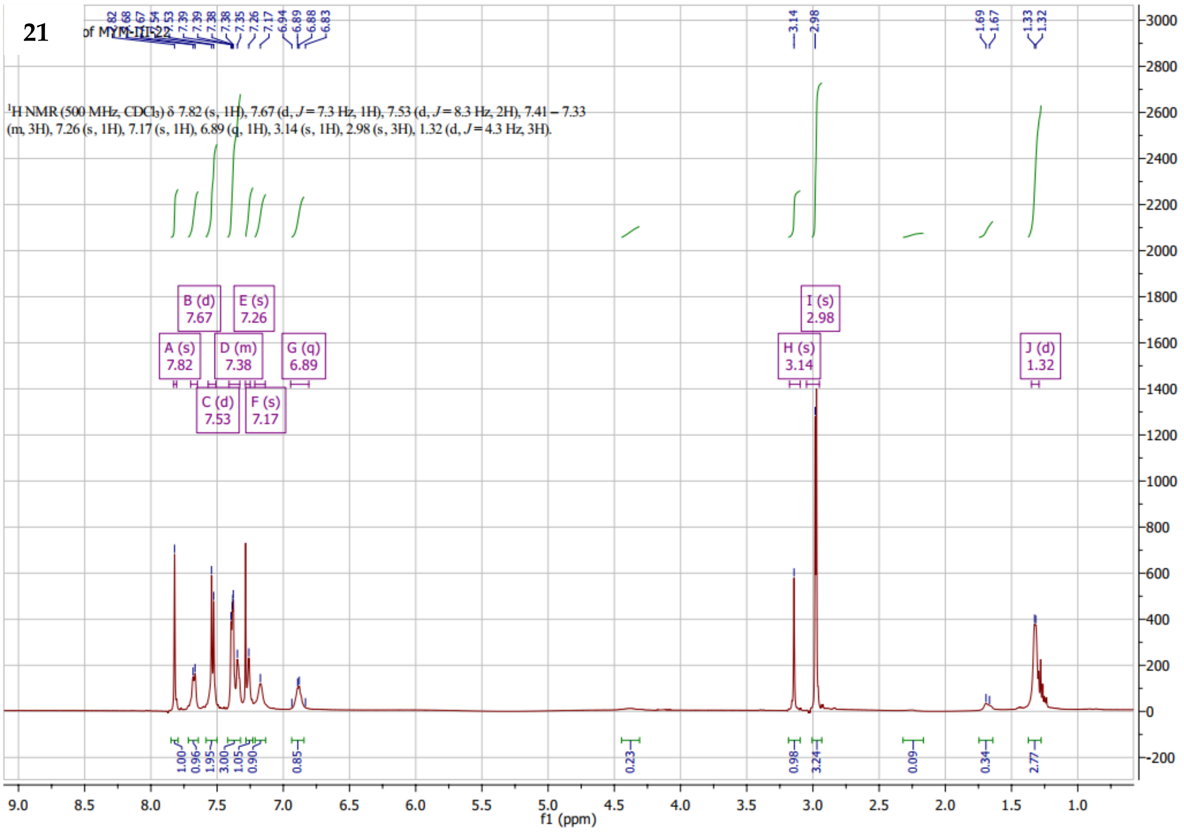

Figure S49. ¹HNMR spectrum of compound **21**

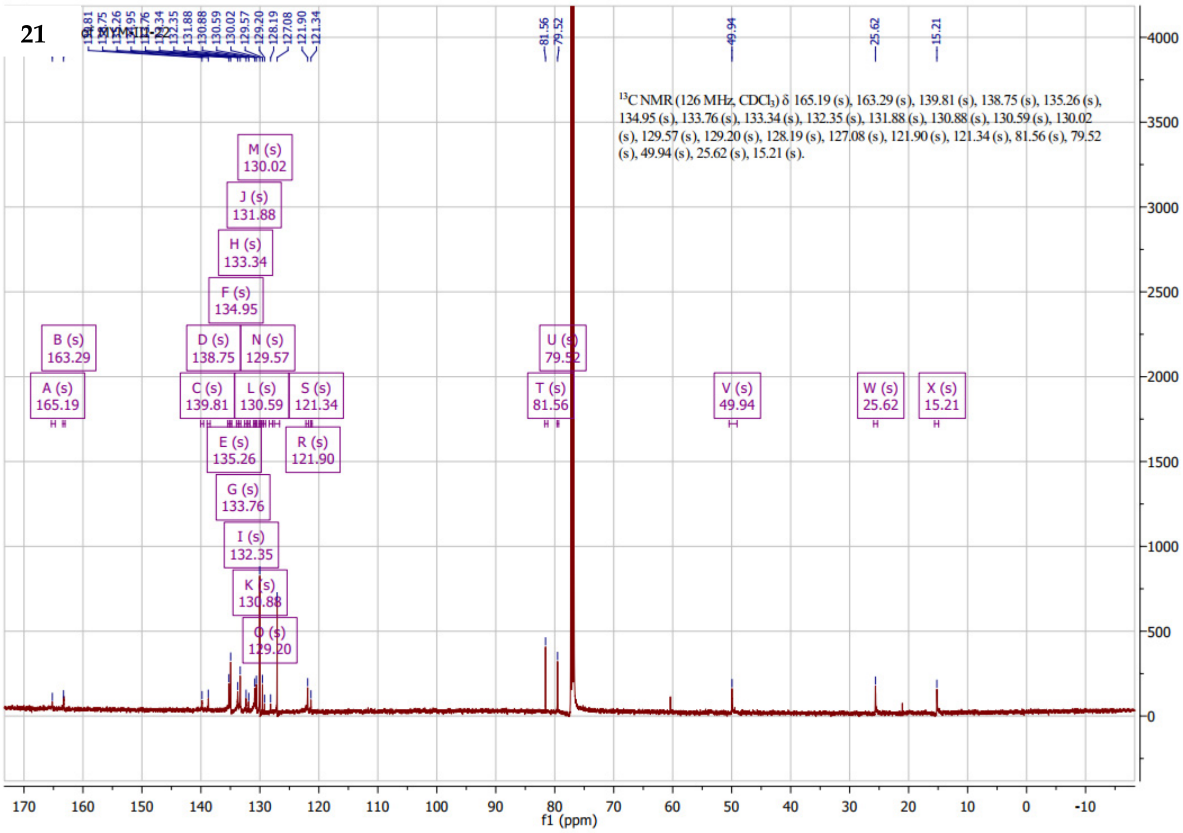

Figure S50. ^{13}CNMR spectrum of compound **21**

27

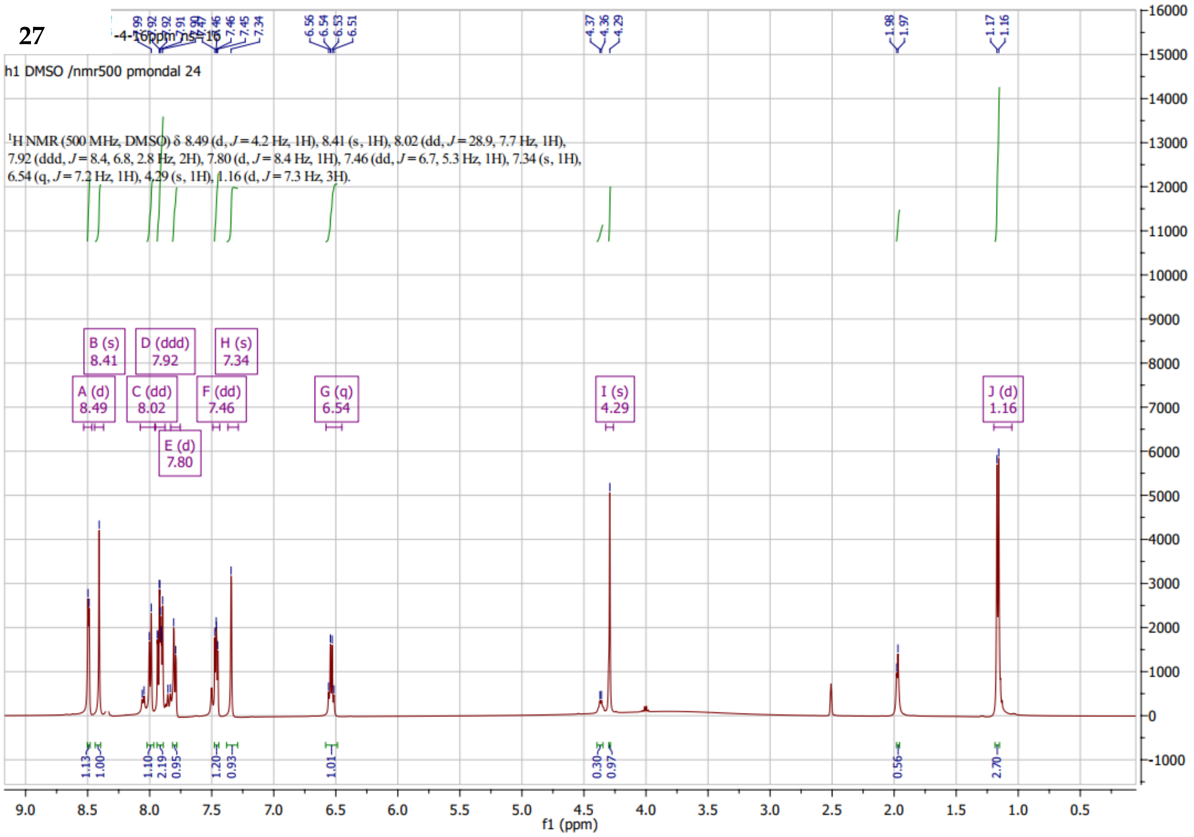

Figure S51. ¹HNMR spectrum of compound **27**

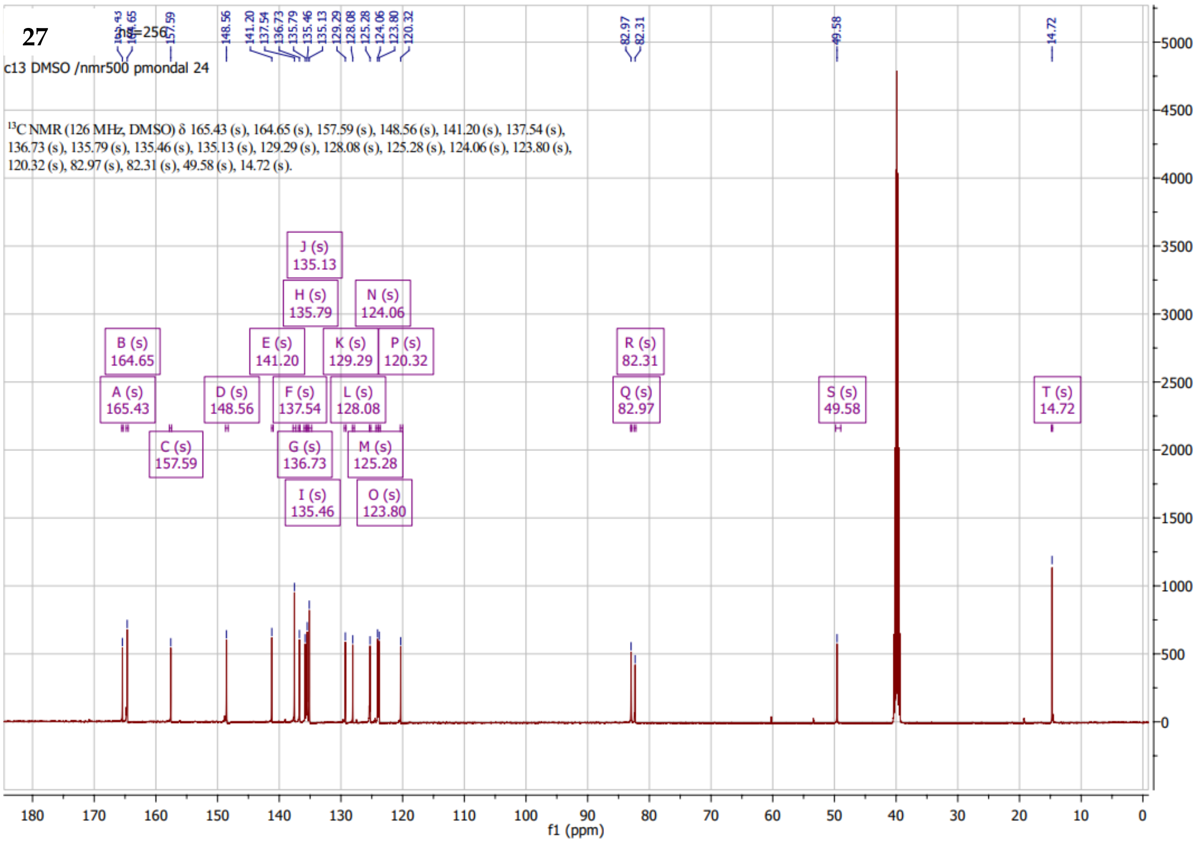

Figure S52. ^{13}CNMR spectrum of compound **51**

31

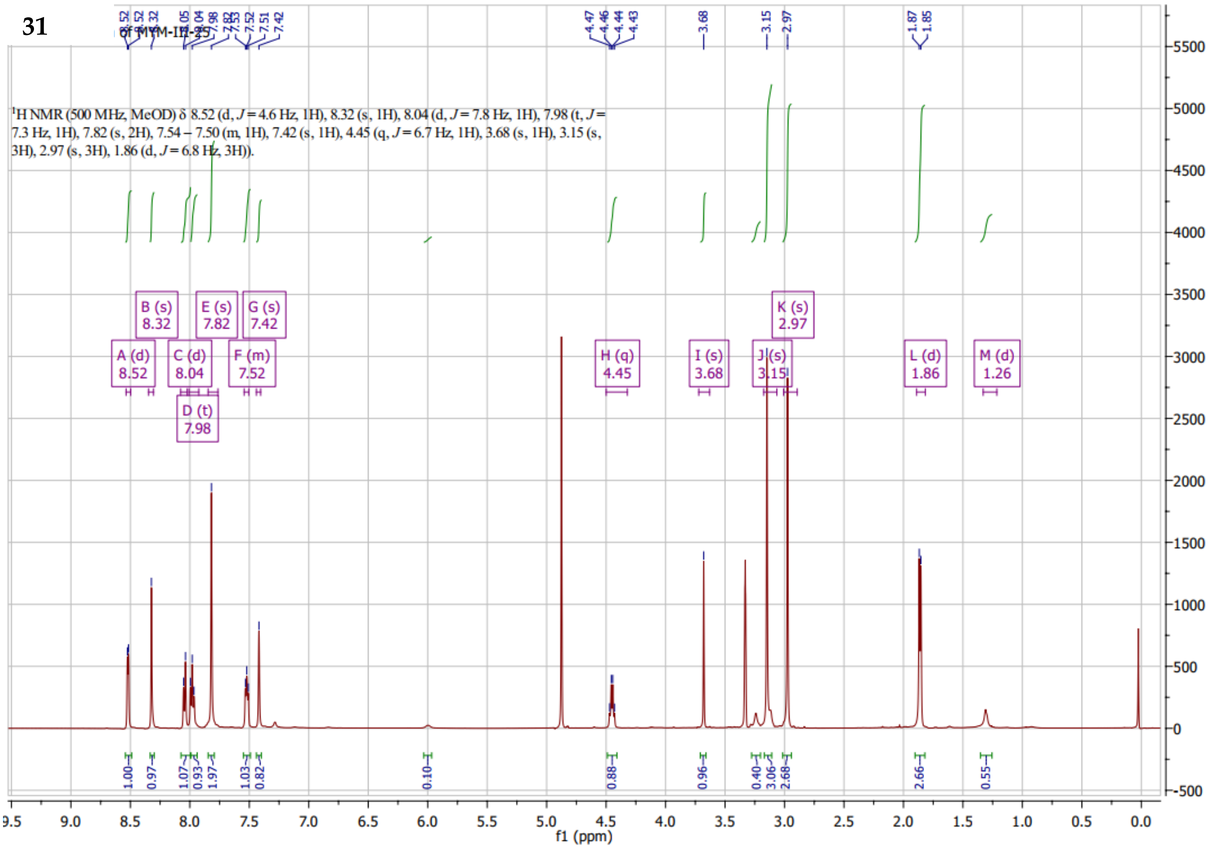

Figure S53. ^{1}HNMR spectrum of compound **31**

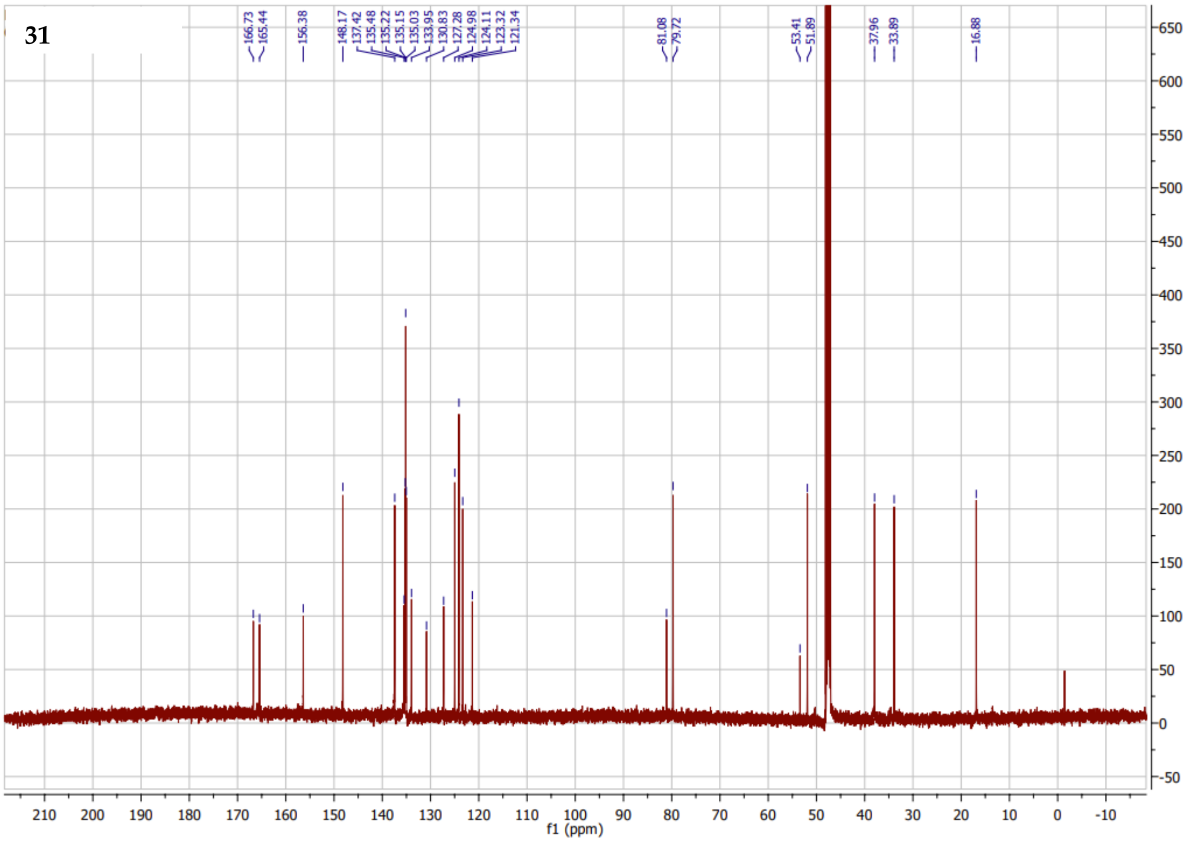

Figure S54. ^{13}CNMR spectrum of compound **31**

23

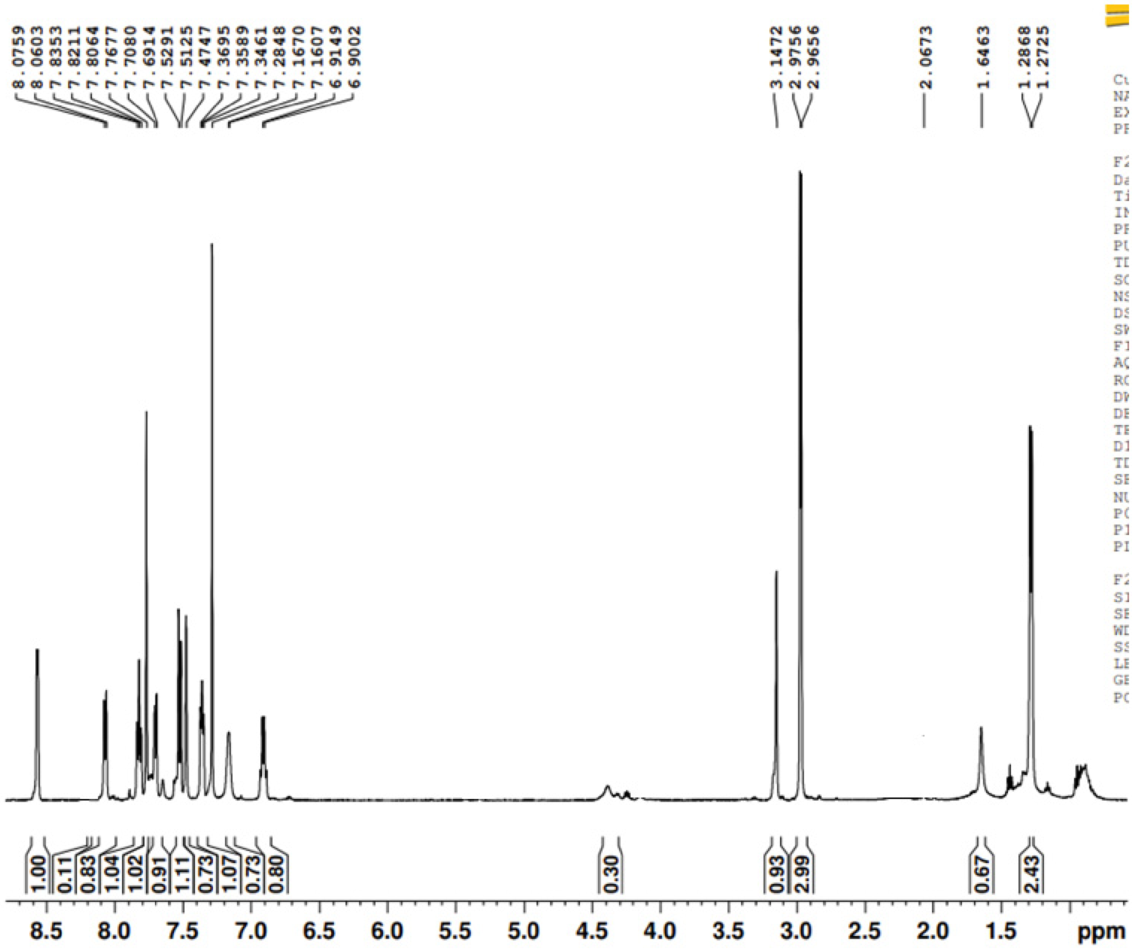

Figure S55. ^{1}HNMR spectrum of compound **23**

23

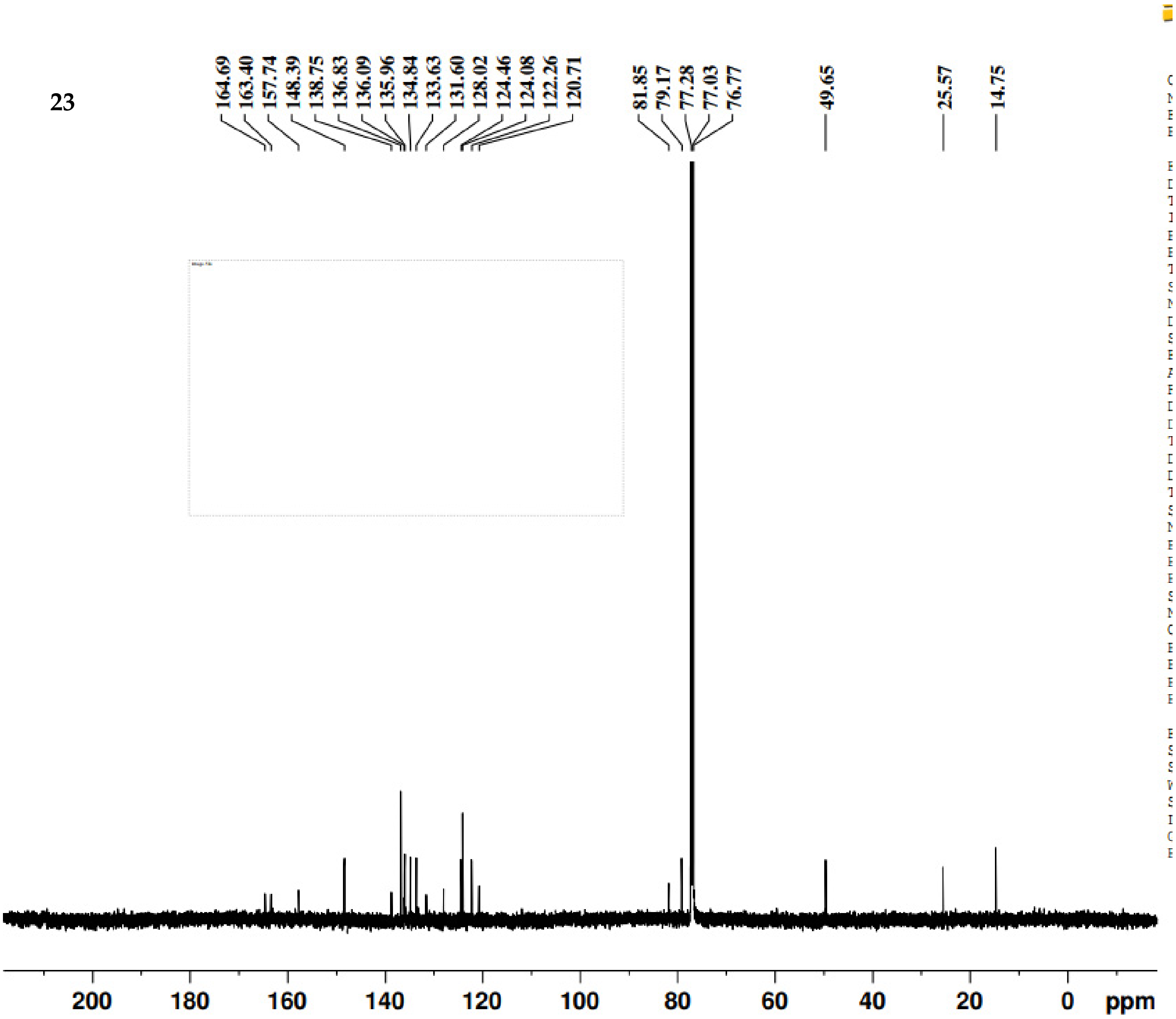

Figure S56. ^{13}CNMR spectrum of compound **23**

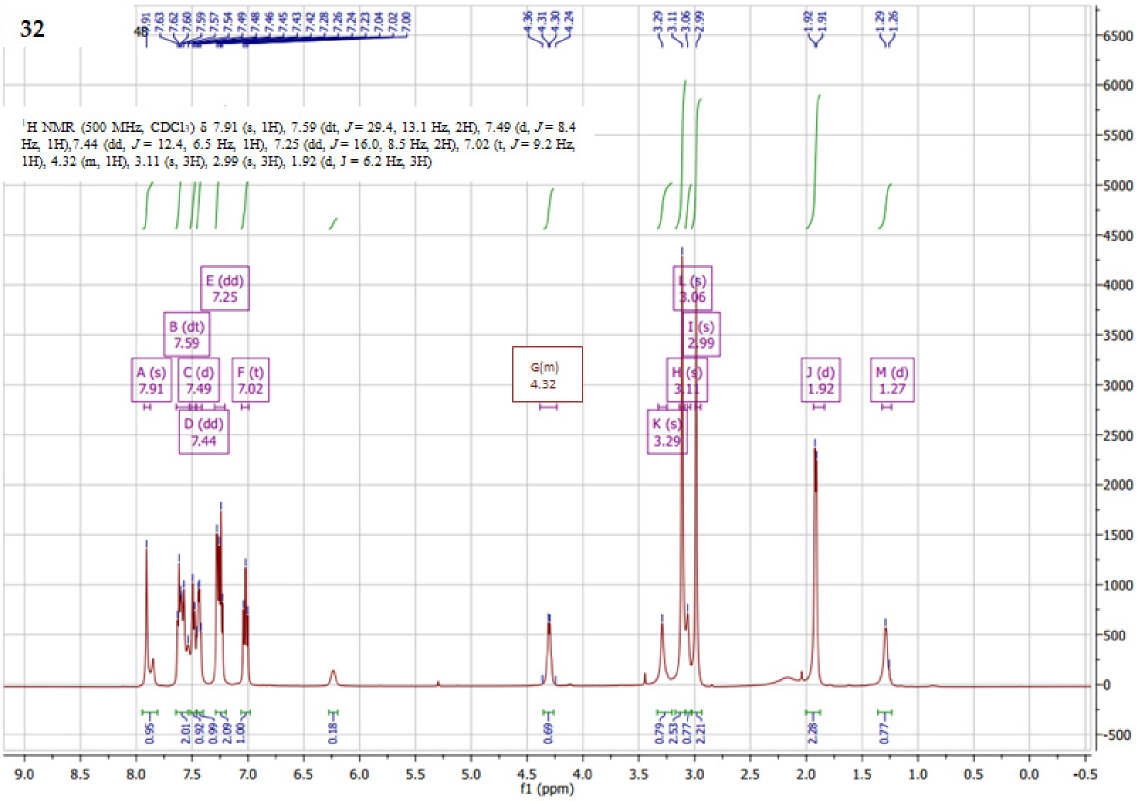

Figure S57. ¹HNMR spectrum of compound **32**

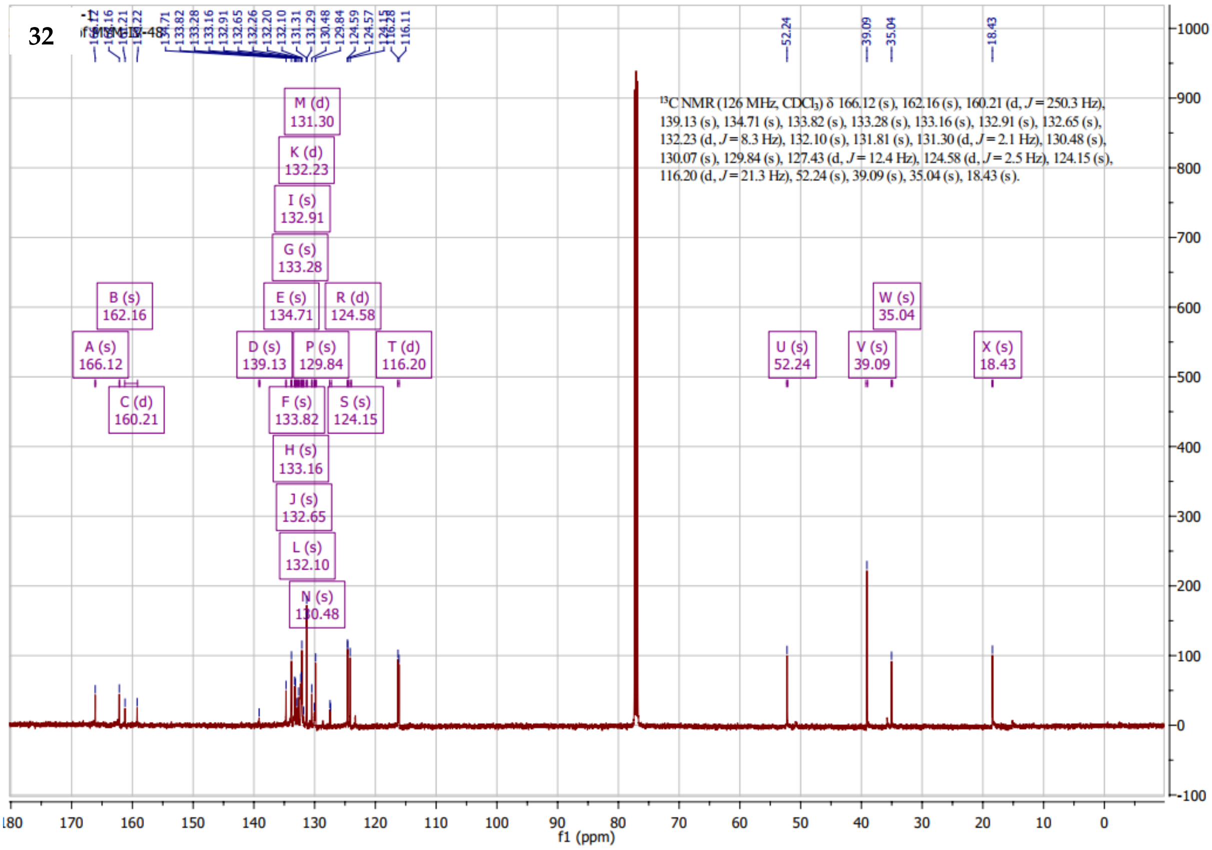

Figure S58. ^{13}CNMR spectrum of compound **32**

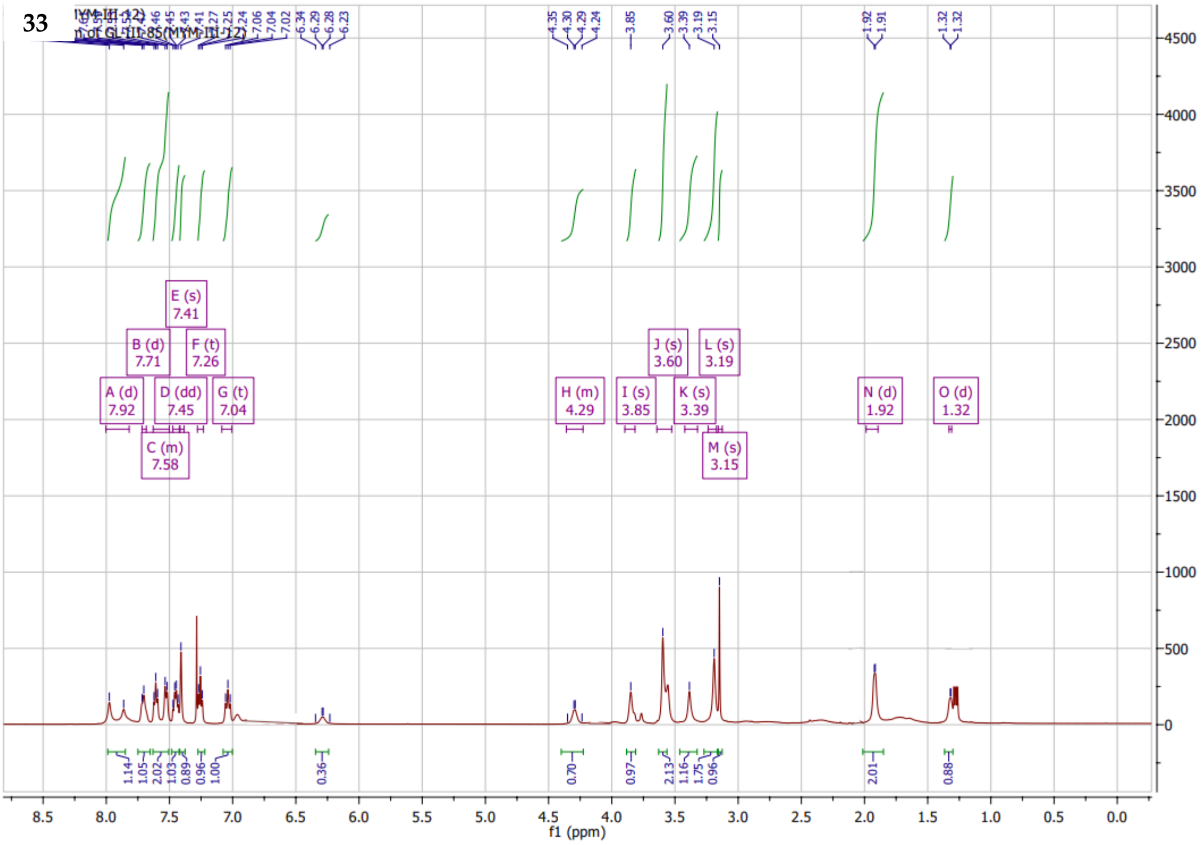

Figure S59. ^{1}HNMR spectrum of compound **33**

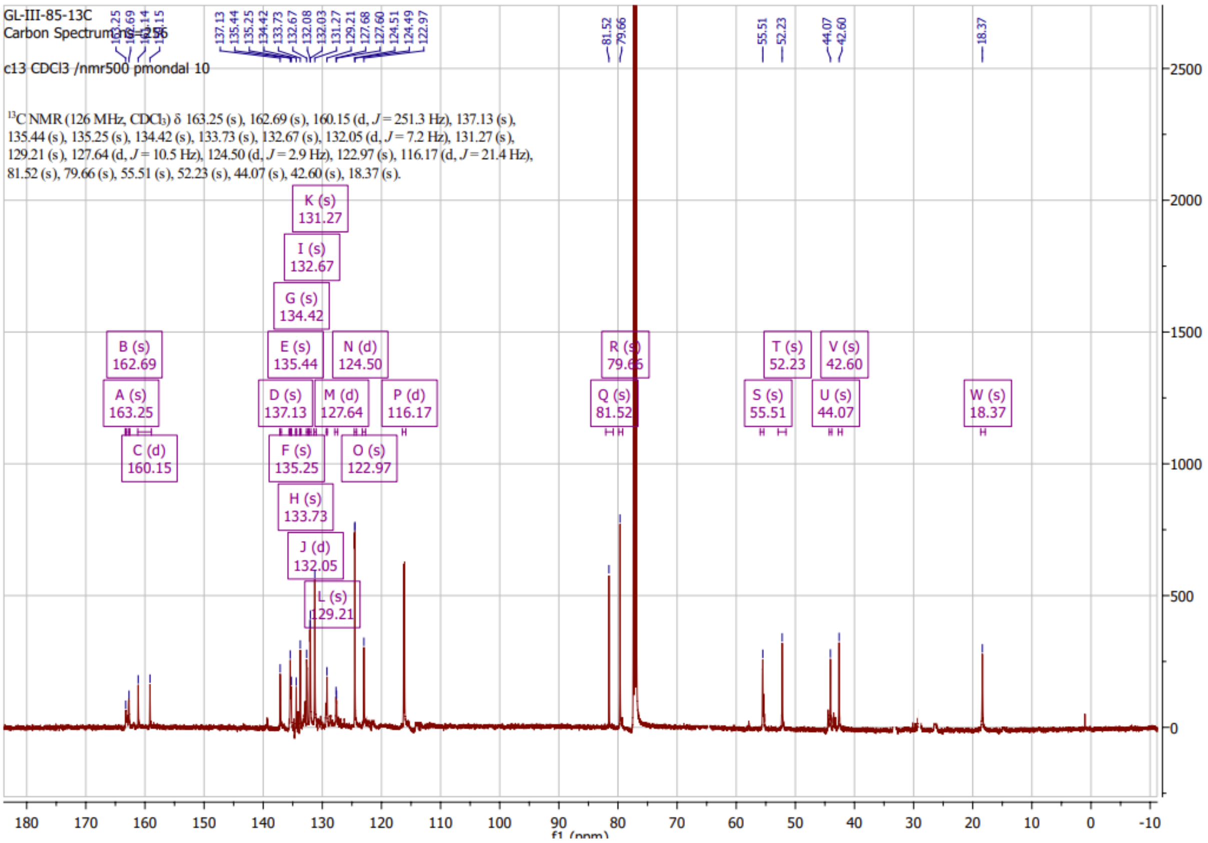

Figure S60. ^{13}CNMR spectrum of compound **33**

35

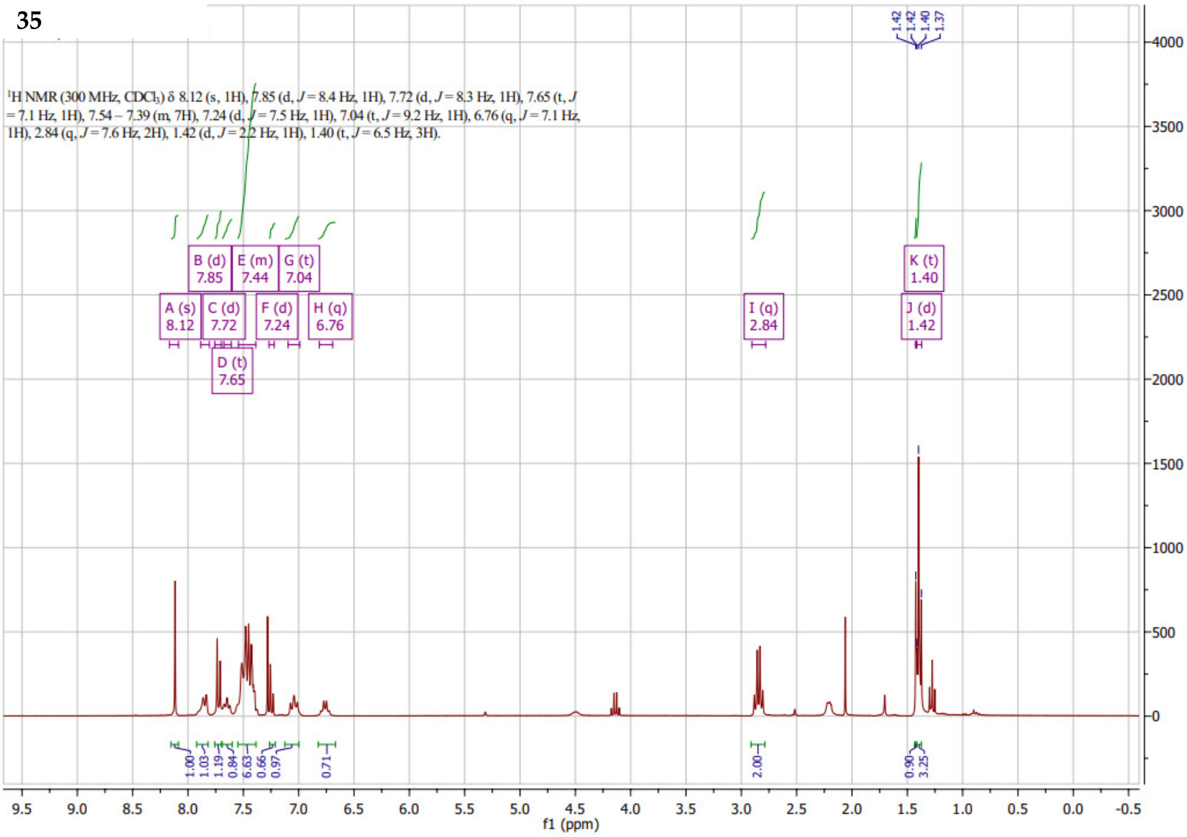

Figure S61. ^{1}HNMR spectrum of compound **35**

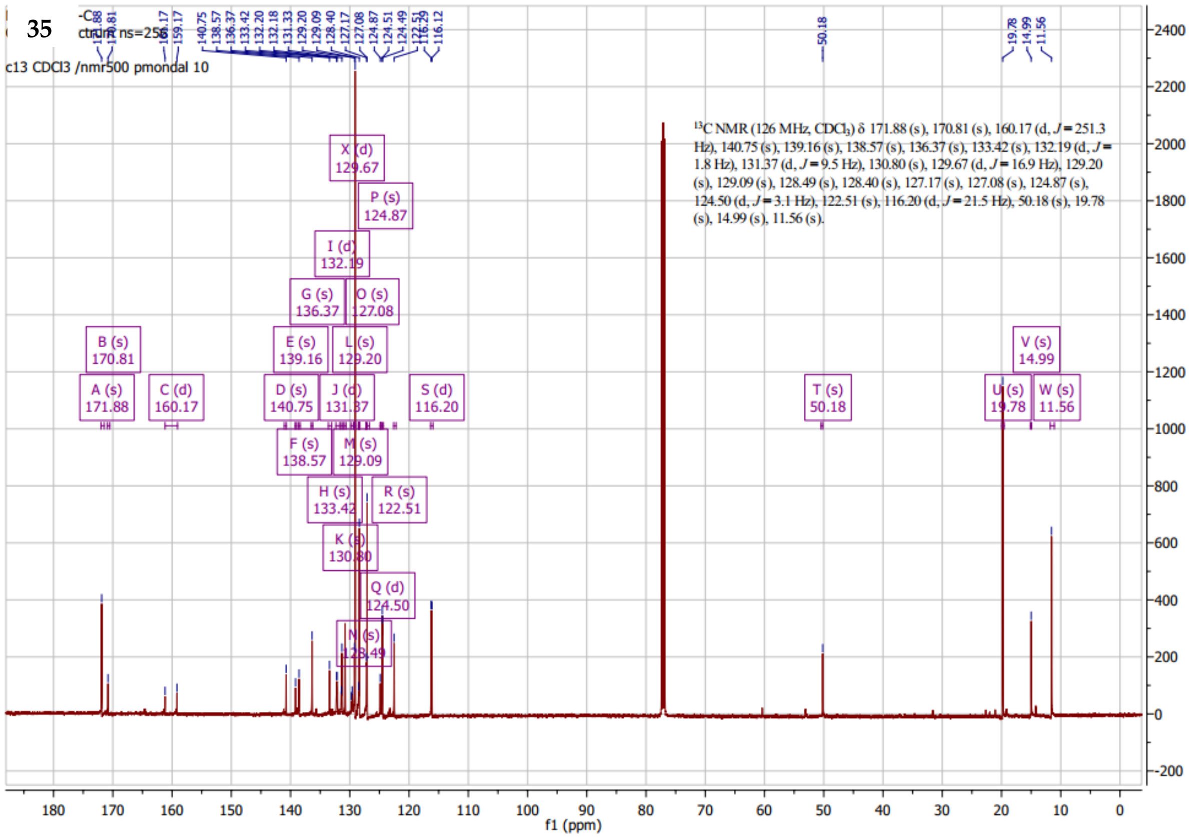

Figure S62. ^{13}CNMR spectrum of compound **35**

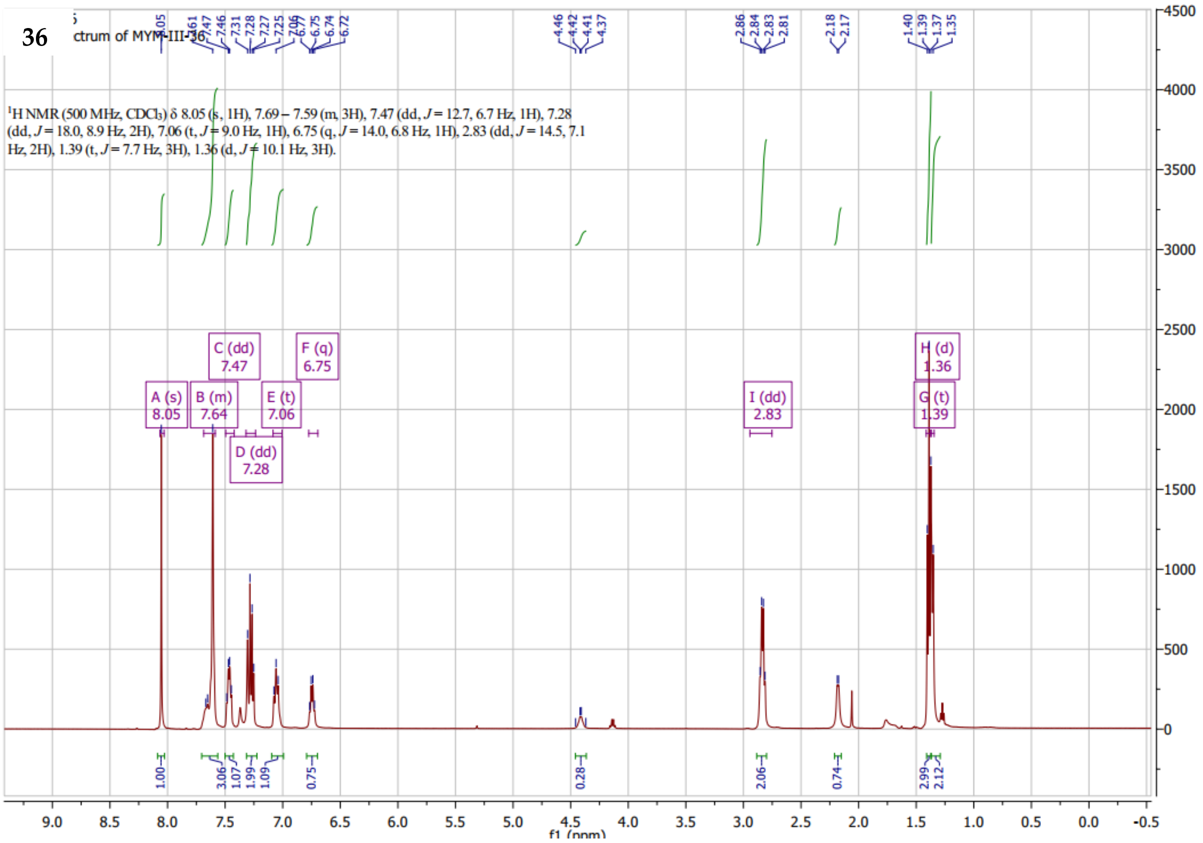

Figure S63. ^{1}HNMR spectrum of compound **36**

36

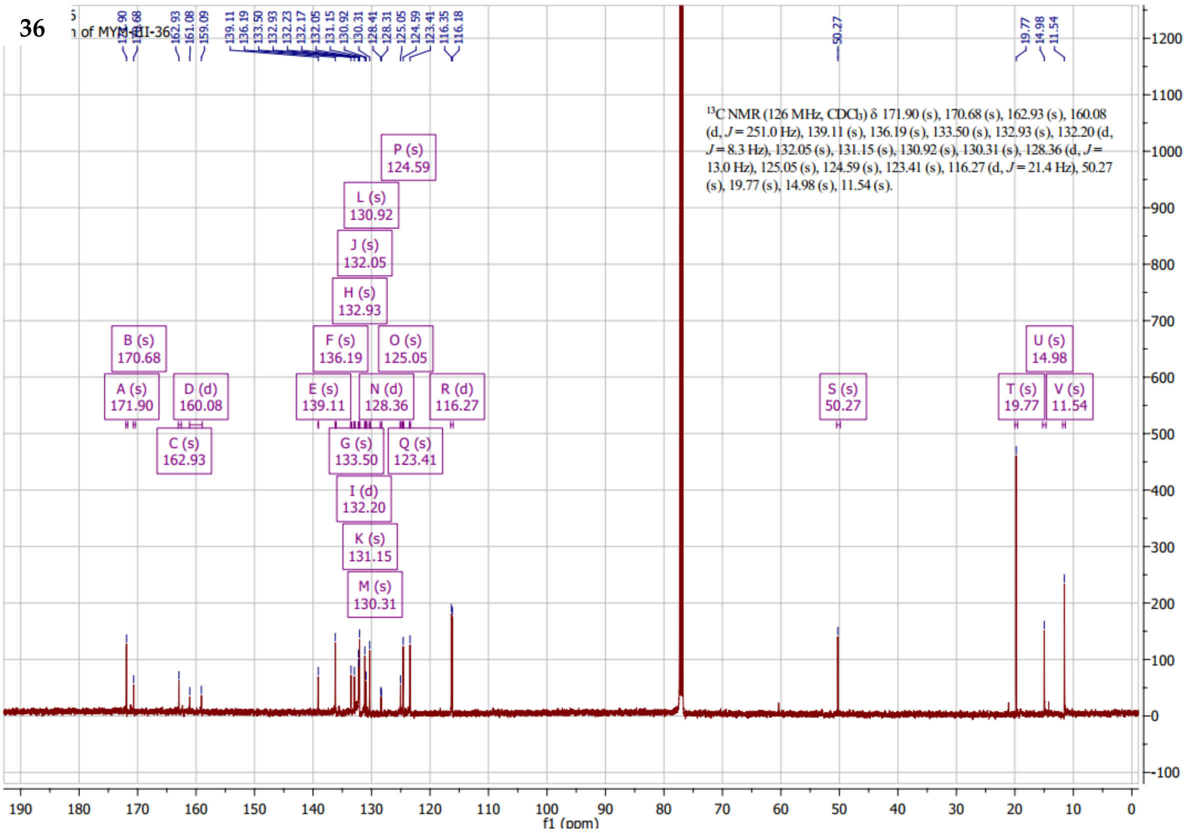

Figure S64. ^{13}CNMR spectrum of compound **36**

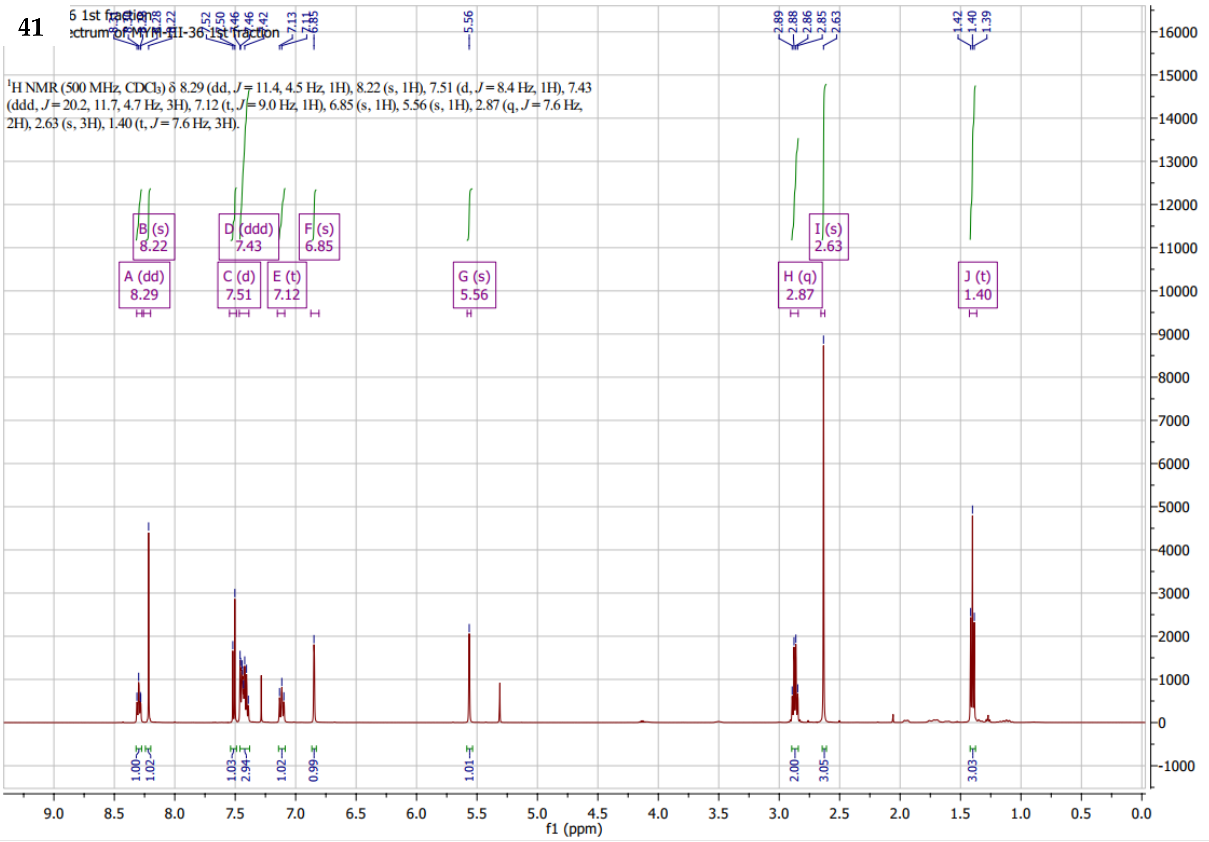

Figure S65. ^{1}HNMR spectrum of compound **41**

41

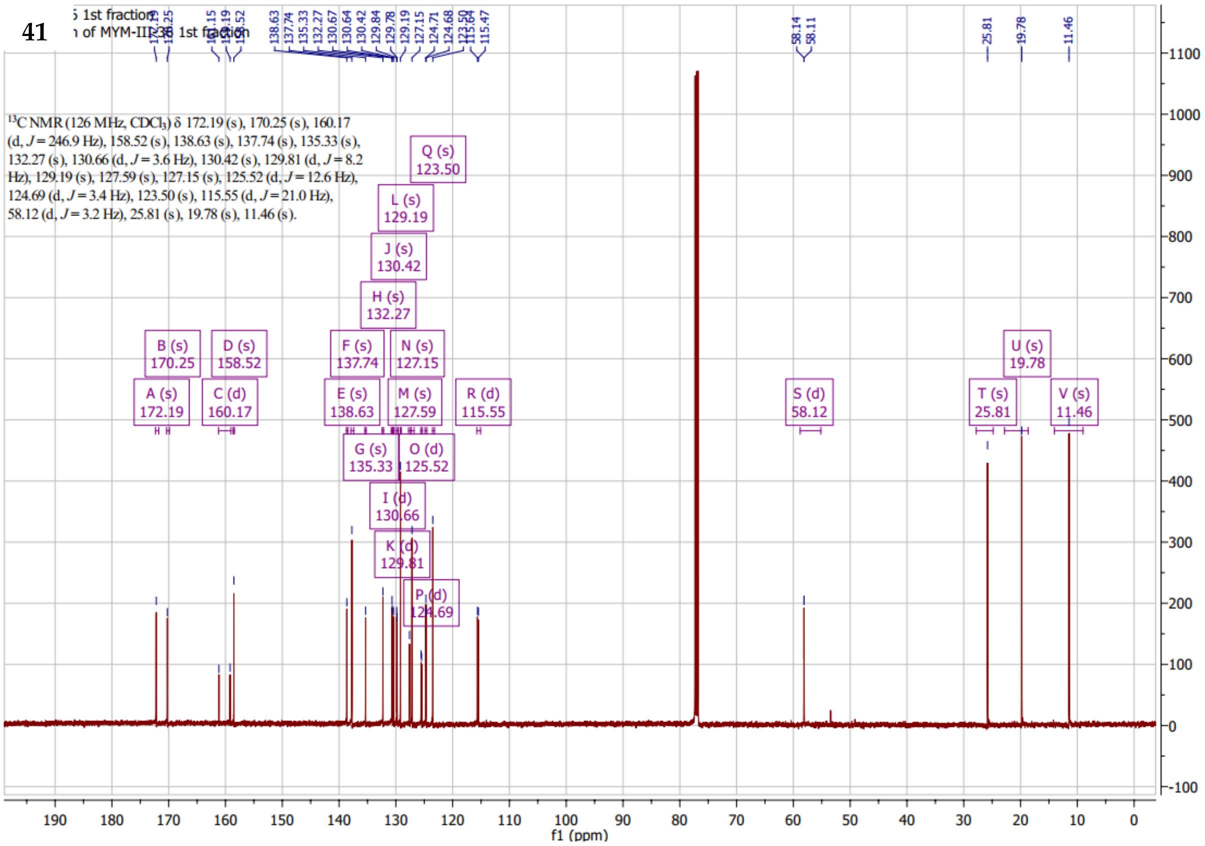

Figure S66. ^{13}CNMR spectrum of compound **41**

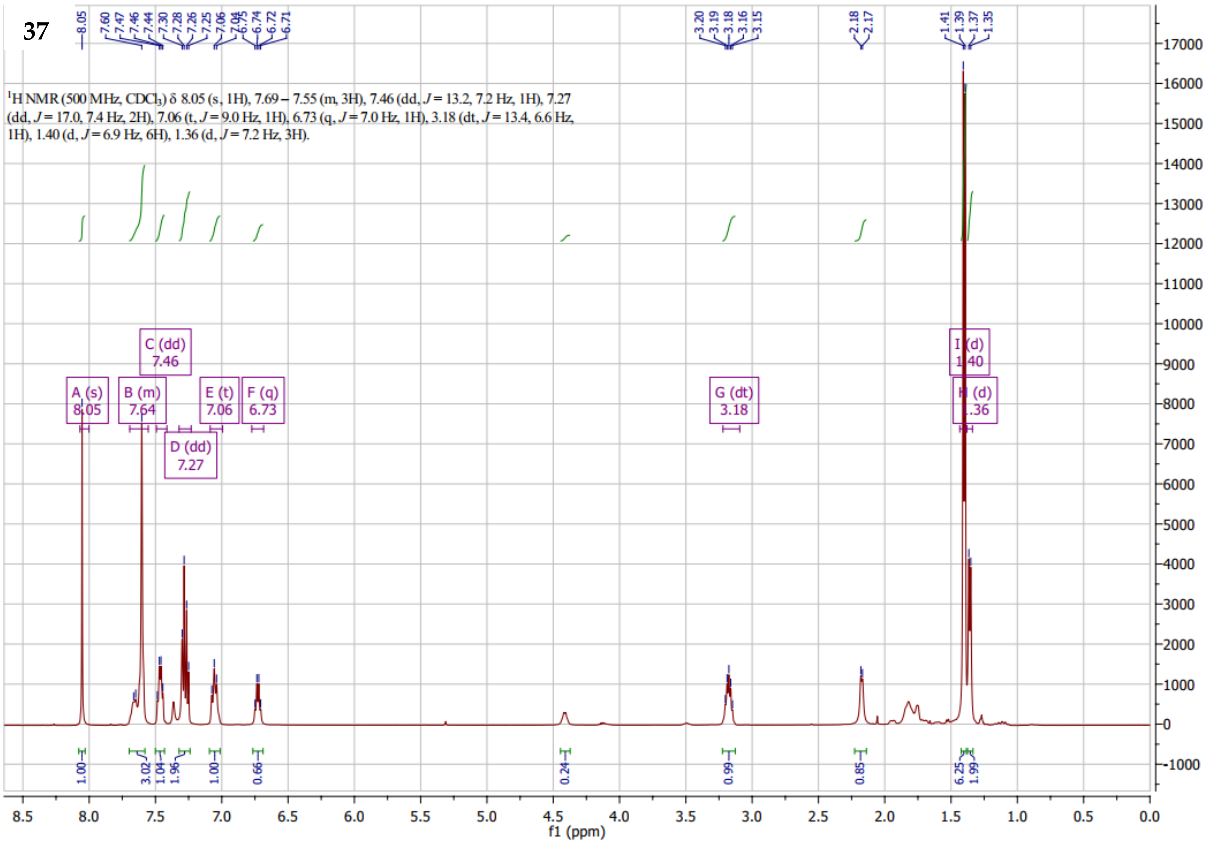

Figure S67. ^{1}HNMR spectrum of compound **37**

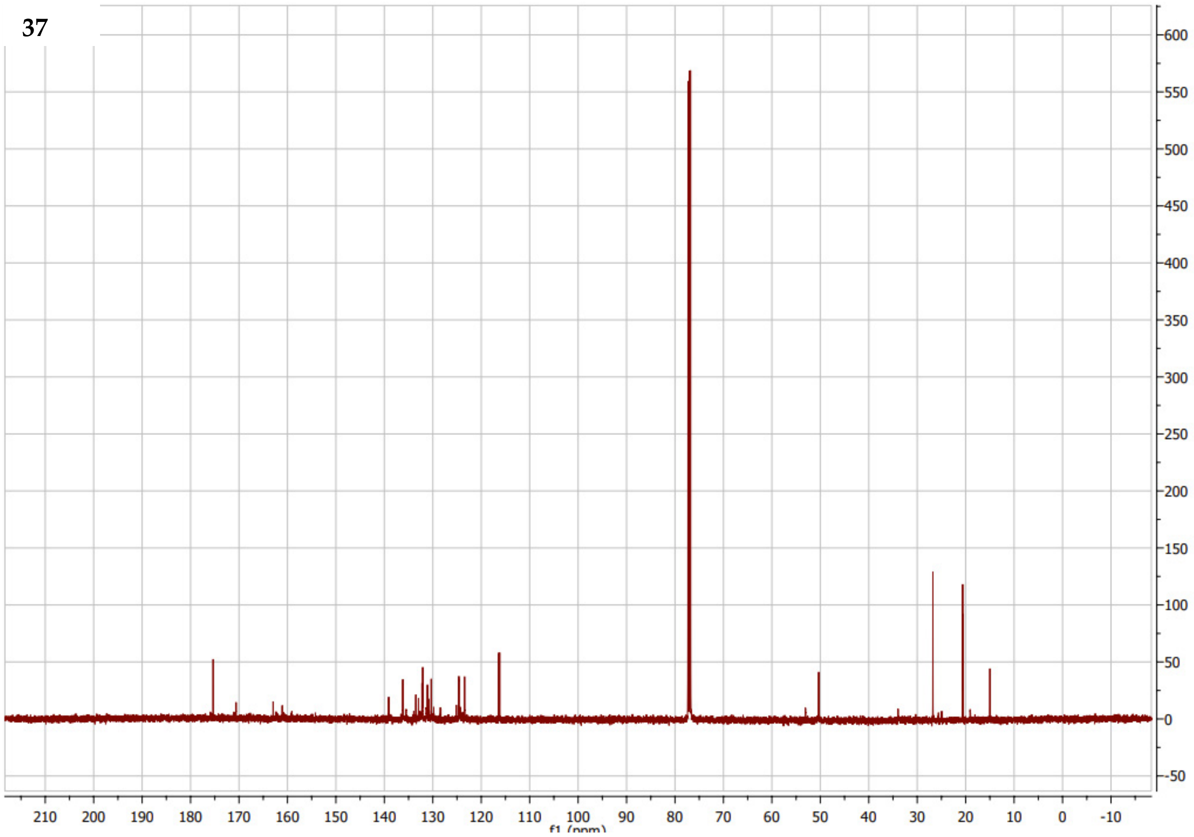

Figure S68. ^{13}CNMR spectrum of compound **37**

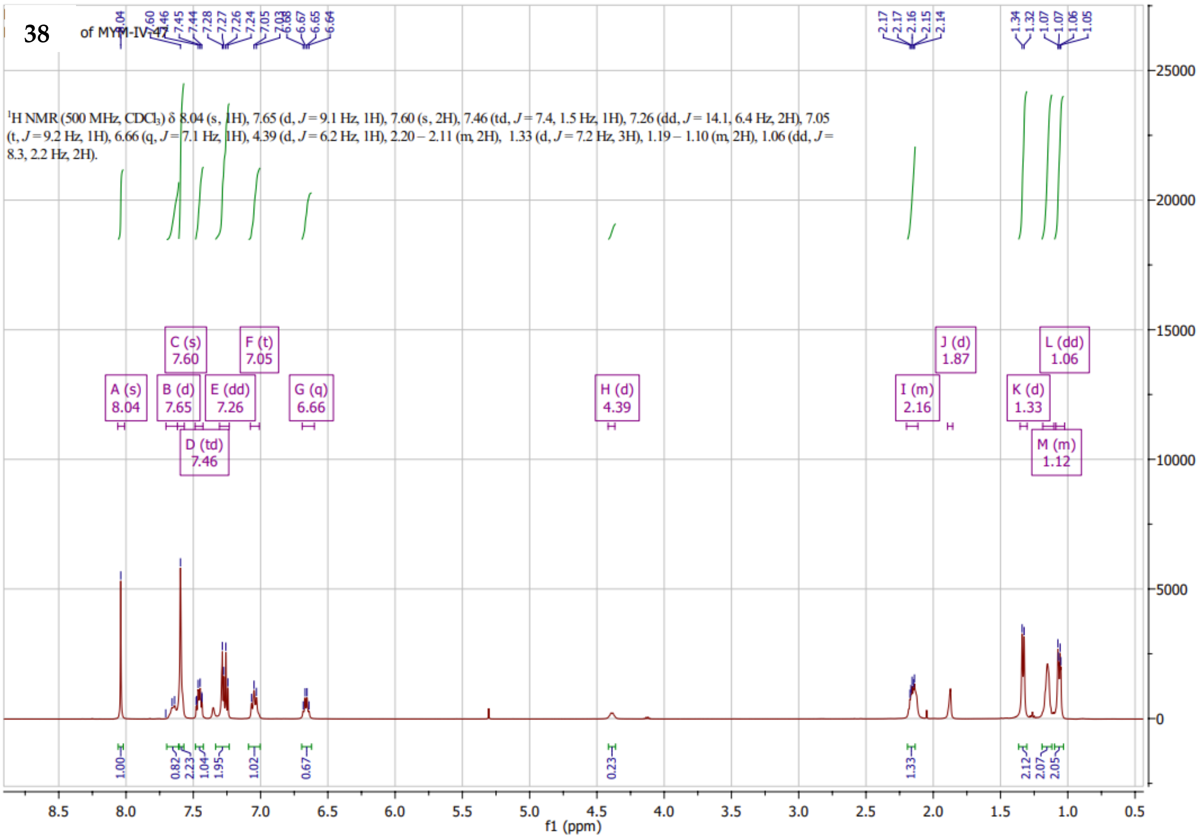

Figure S69. ¹HNMR spectrum of compound **38**

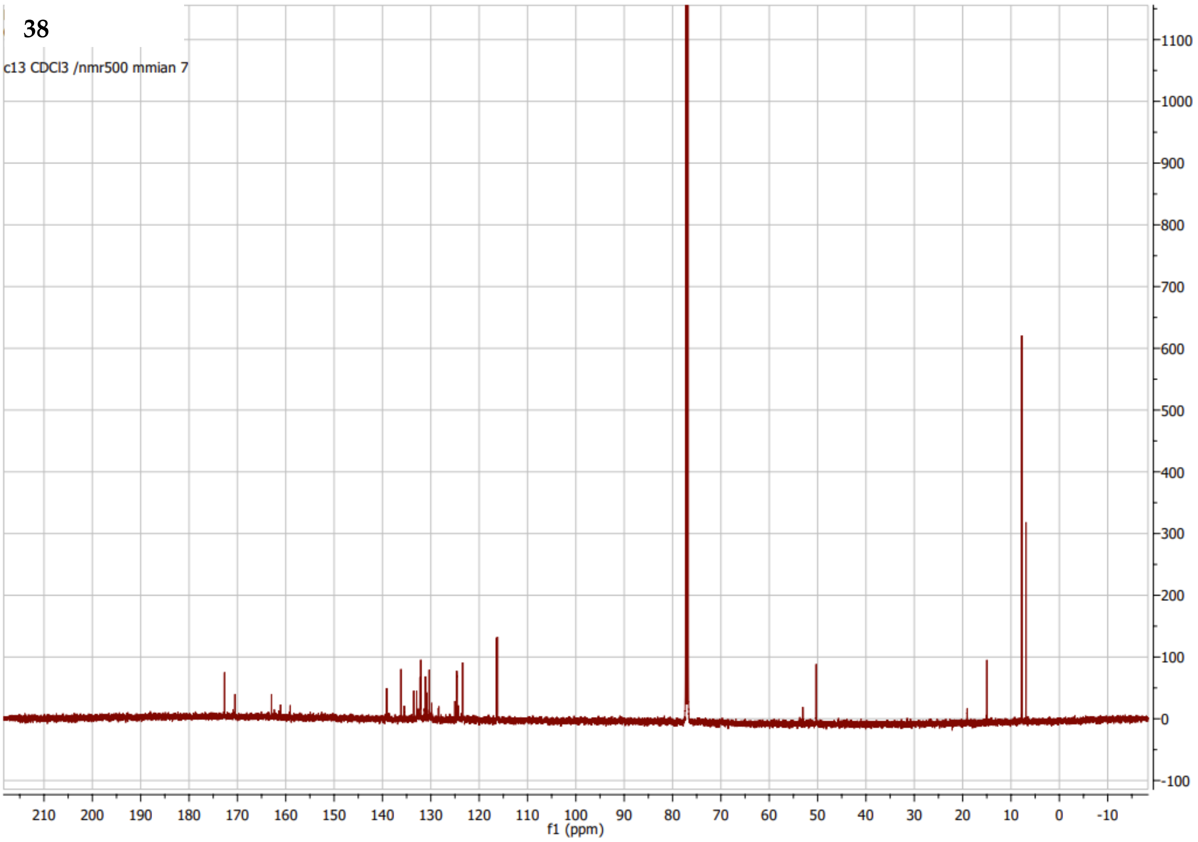

Figure S70. ^{13}CNMR spectrum of compound **38**

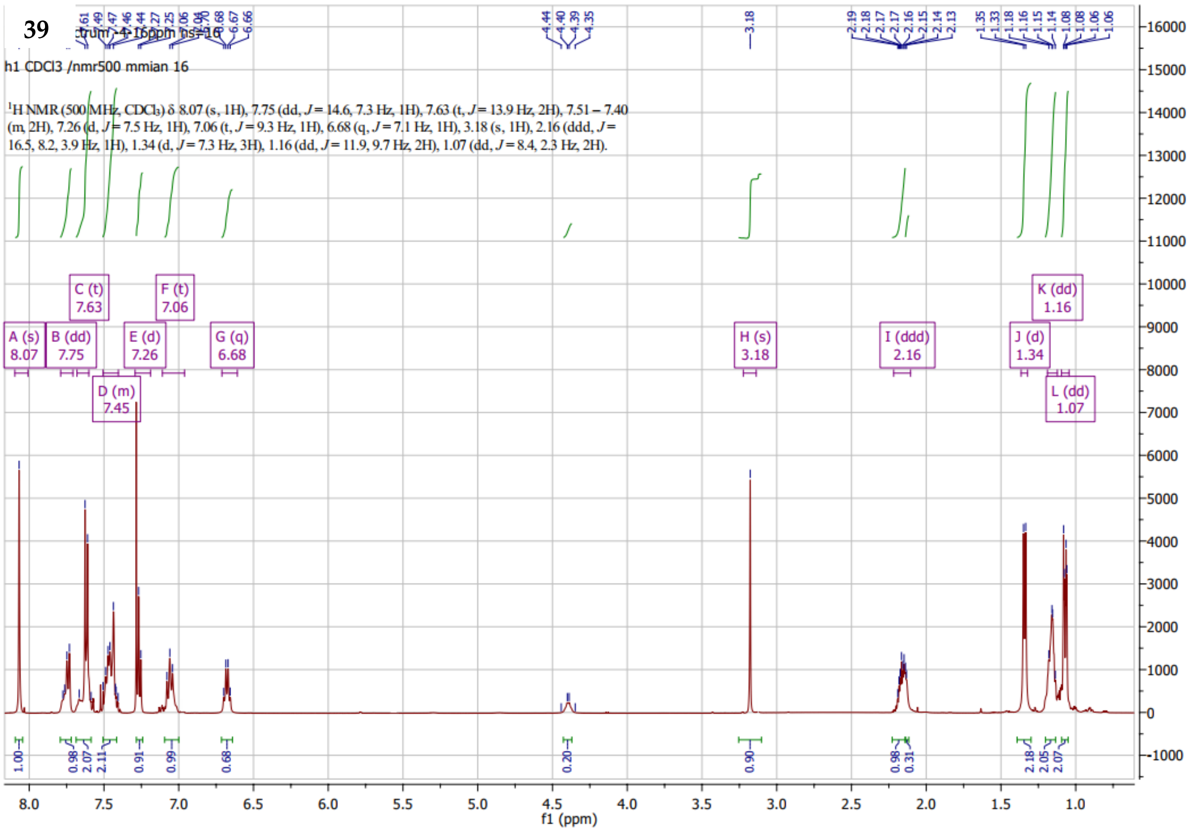

Figure S71. ¹HNMR spectrum of compound **39**

39

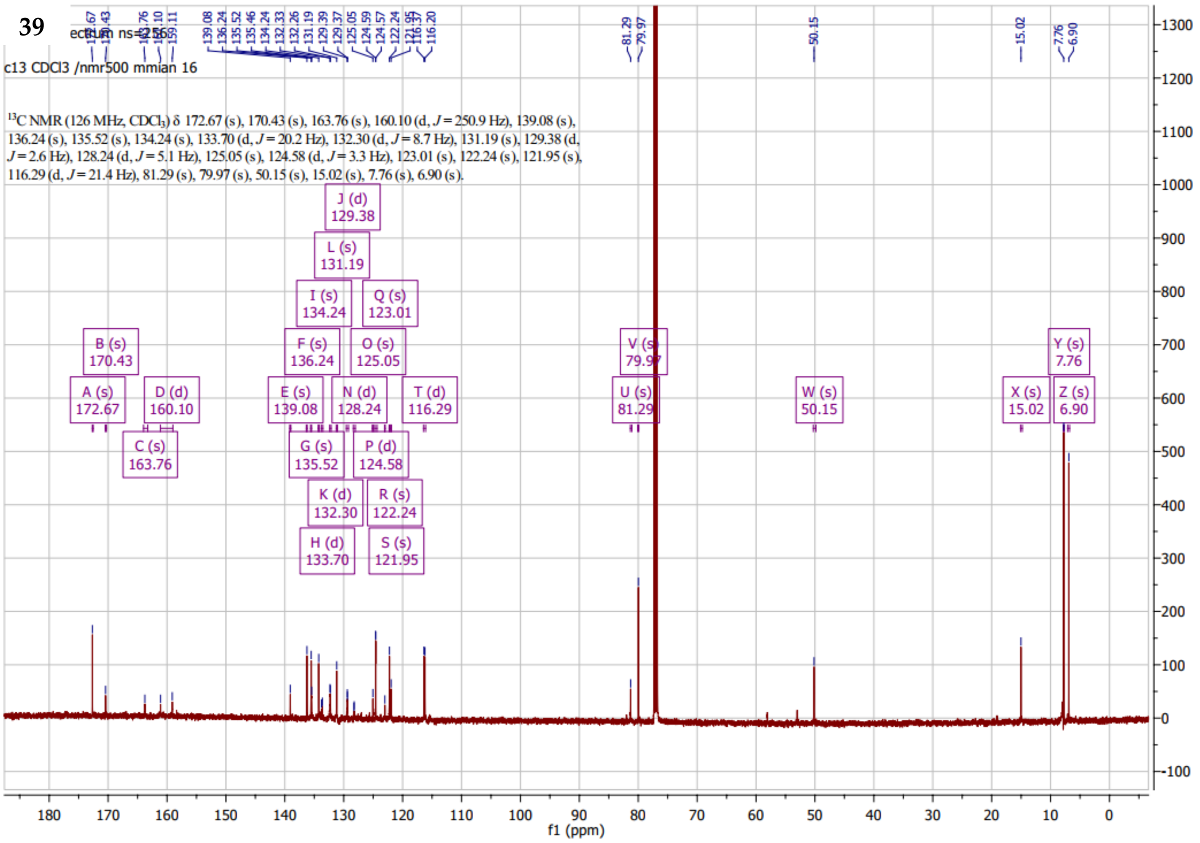

Figure S72. ^{13}CNMR spectrum of compound **39**

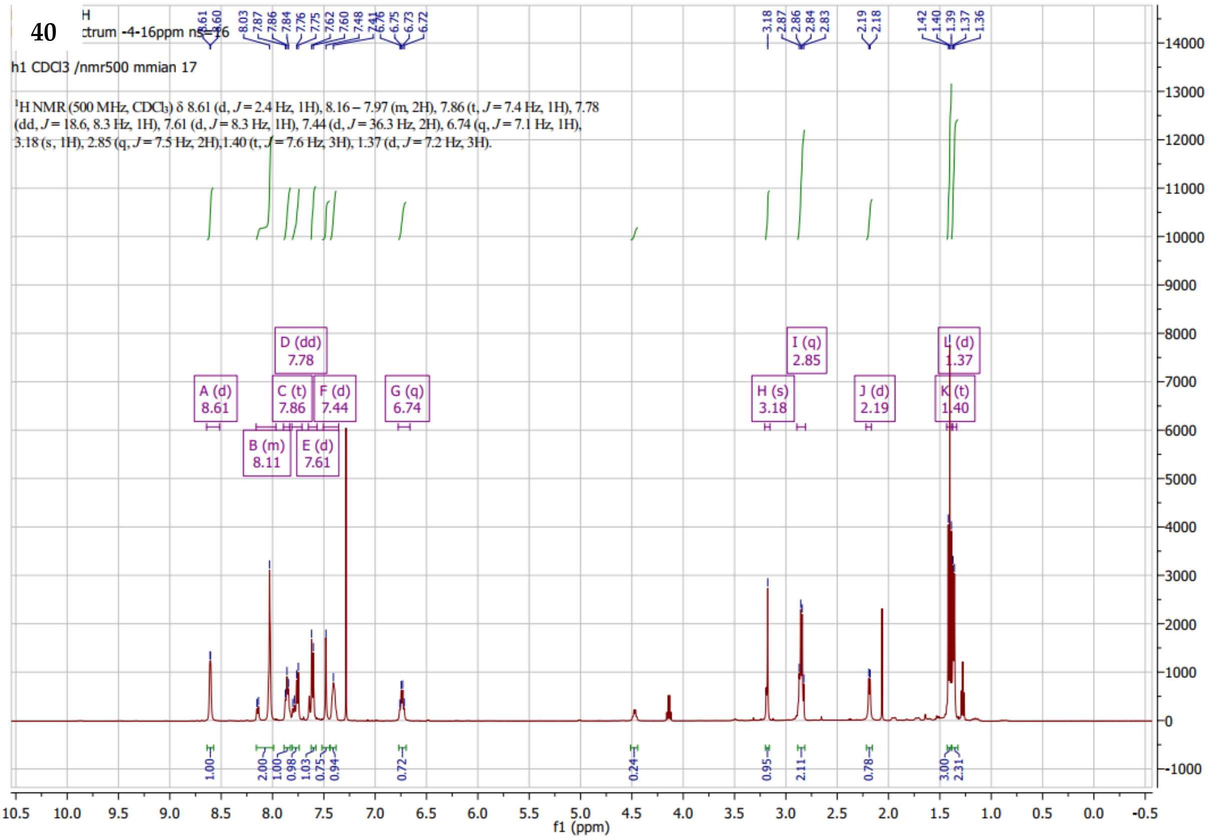

Figure S73. ^{1}HNMR spectrum of compound **40**

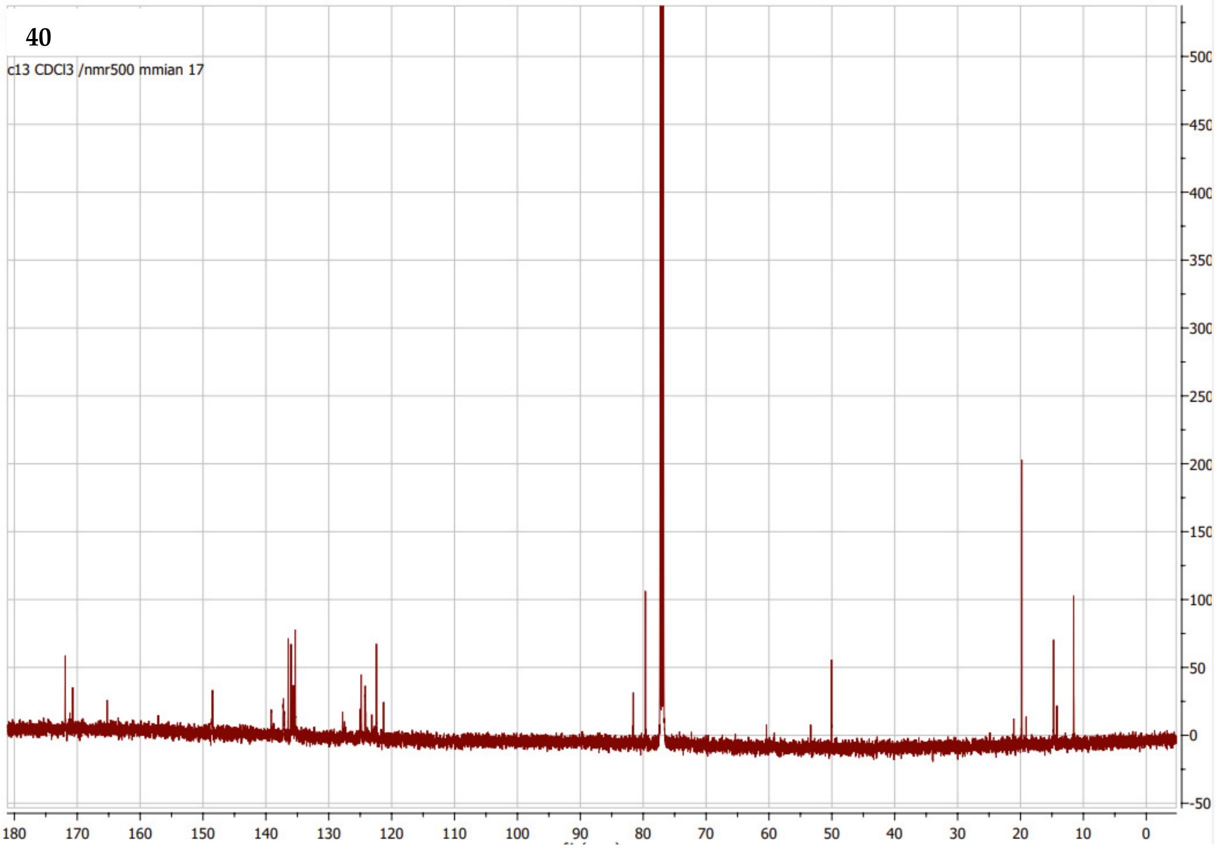

Figure S74. 13CNMR spectrum of compound **40**

43

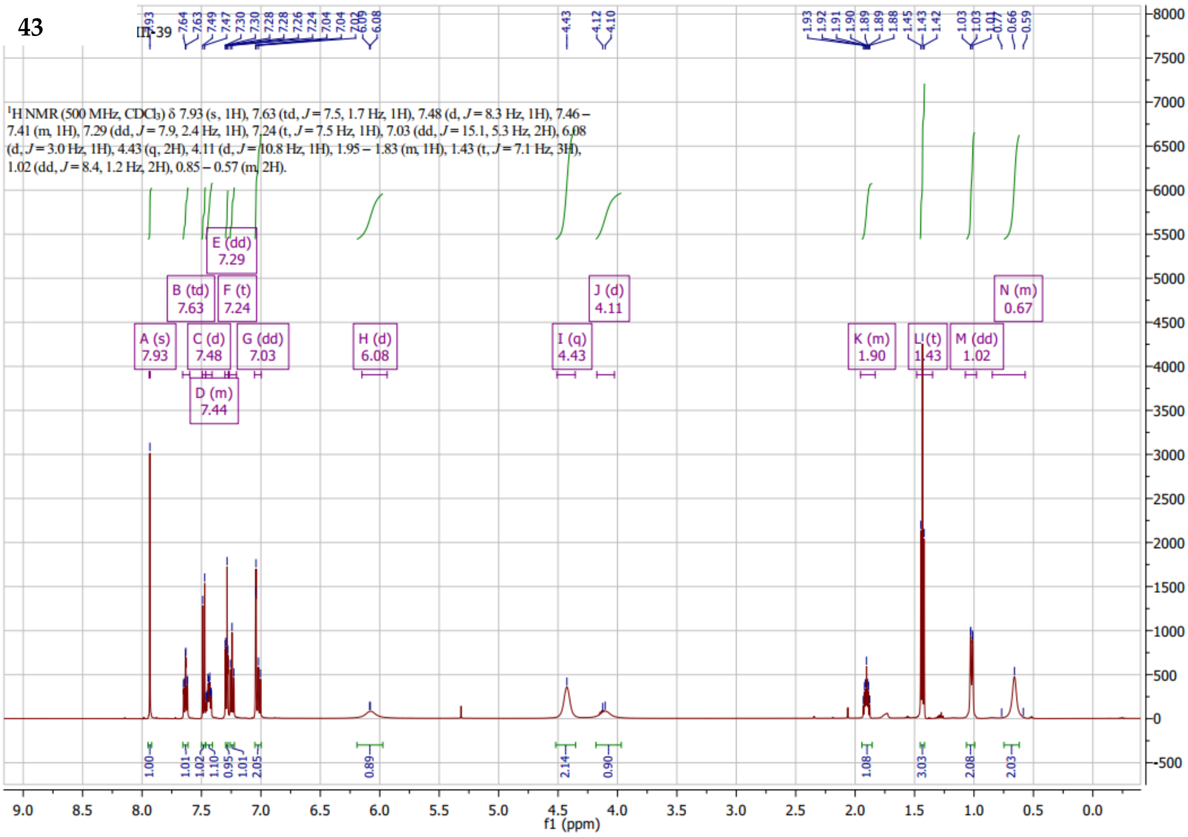

Figure S75. ^{1}HNMR spectrum of compound **43**

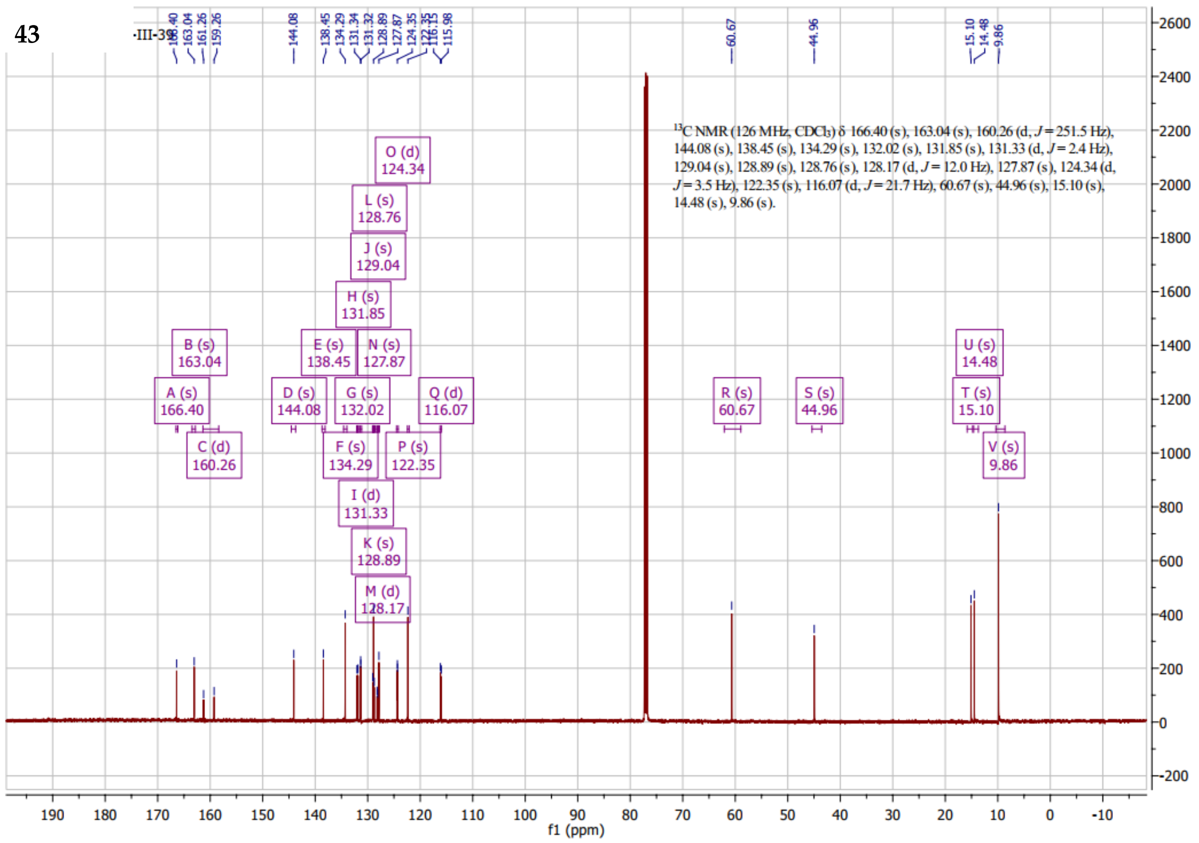

Figure S76. ^{13}CNMR spectrum of compound **43**

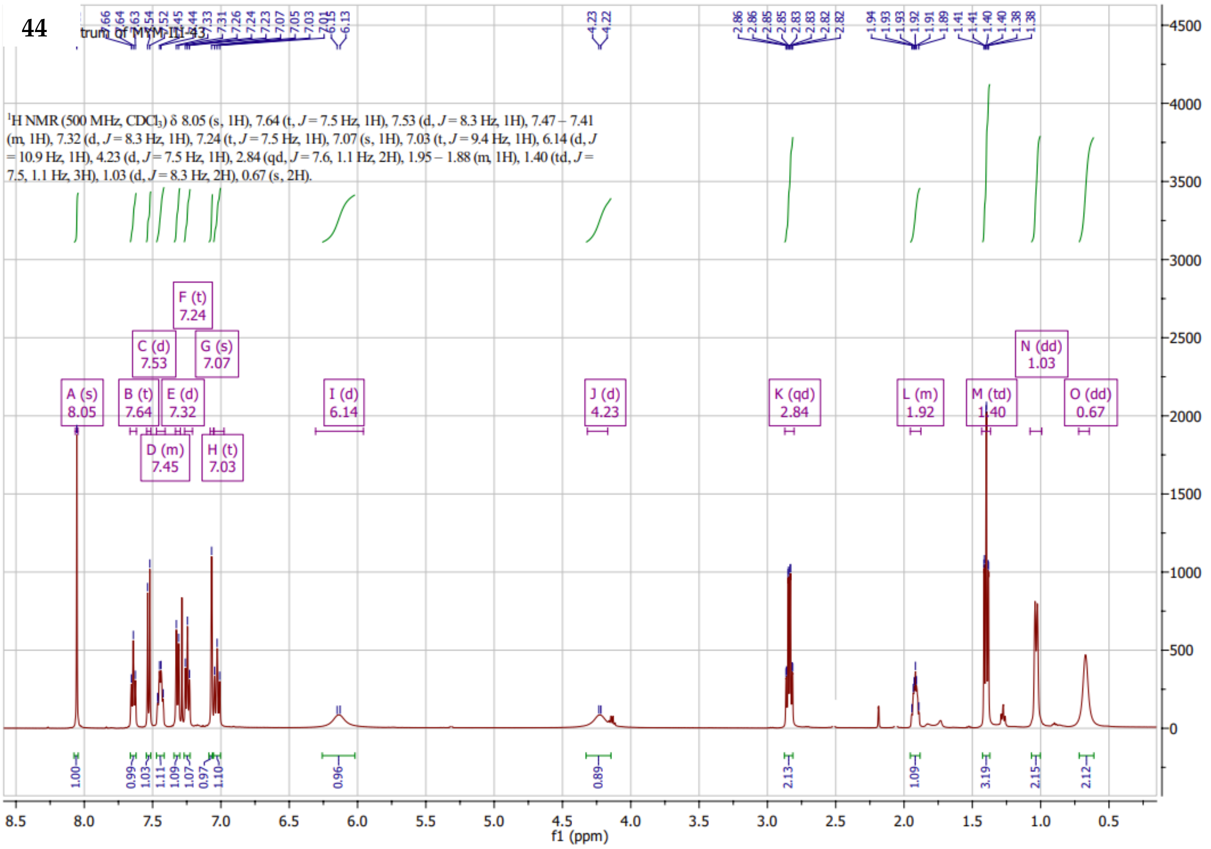

Figure S77. ^{1}HNMR spectrum of compound **44**

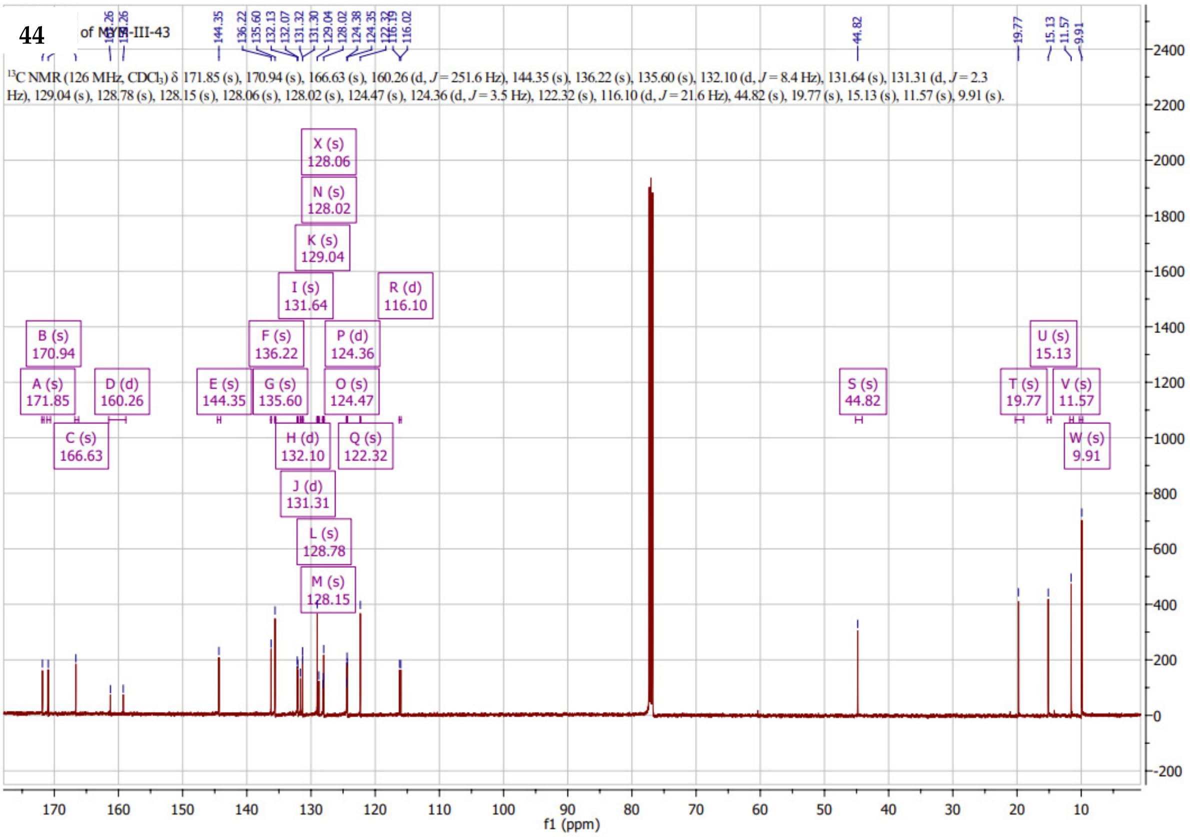

Figure S78. ^{13}CNMR spectrum of compound **44**

45

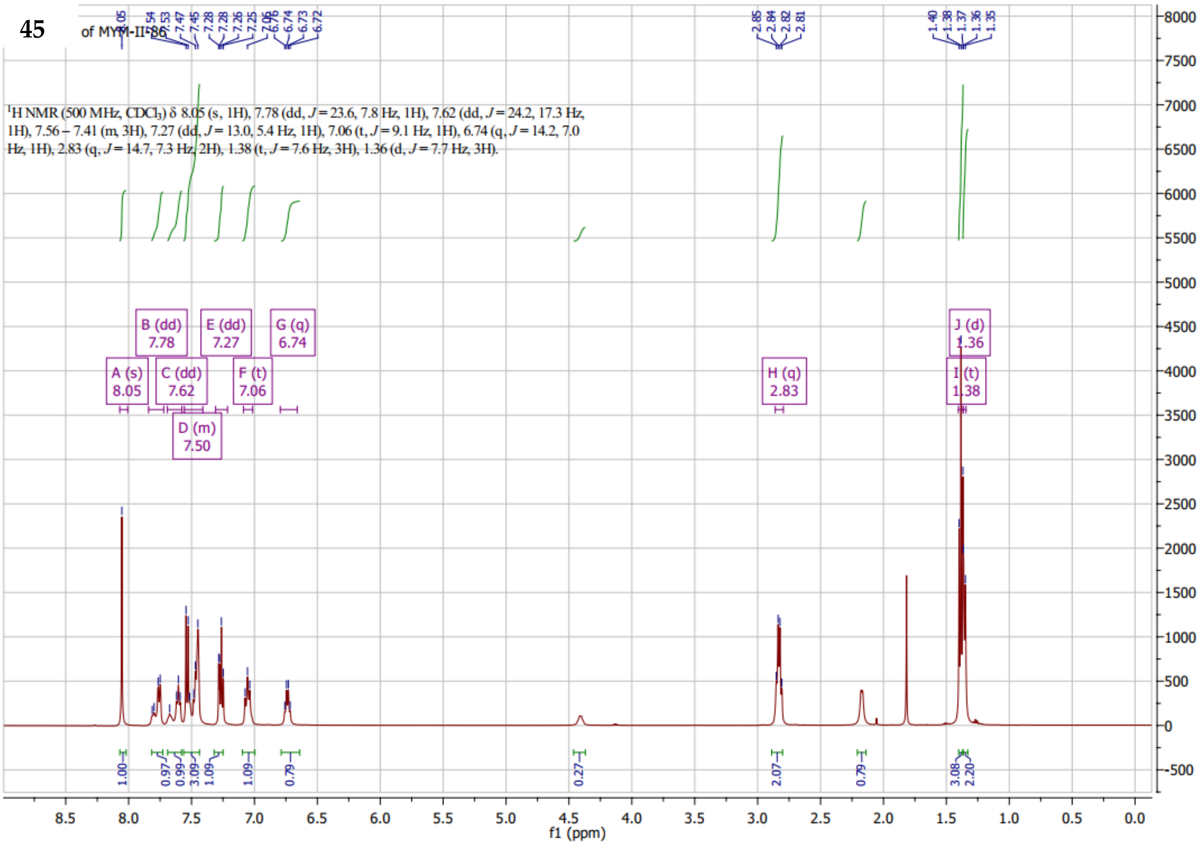

Figure S79. ¹HNMR spectrum of compound **45**

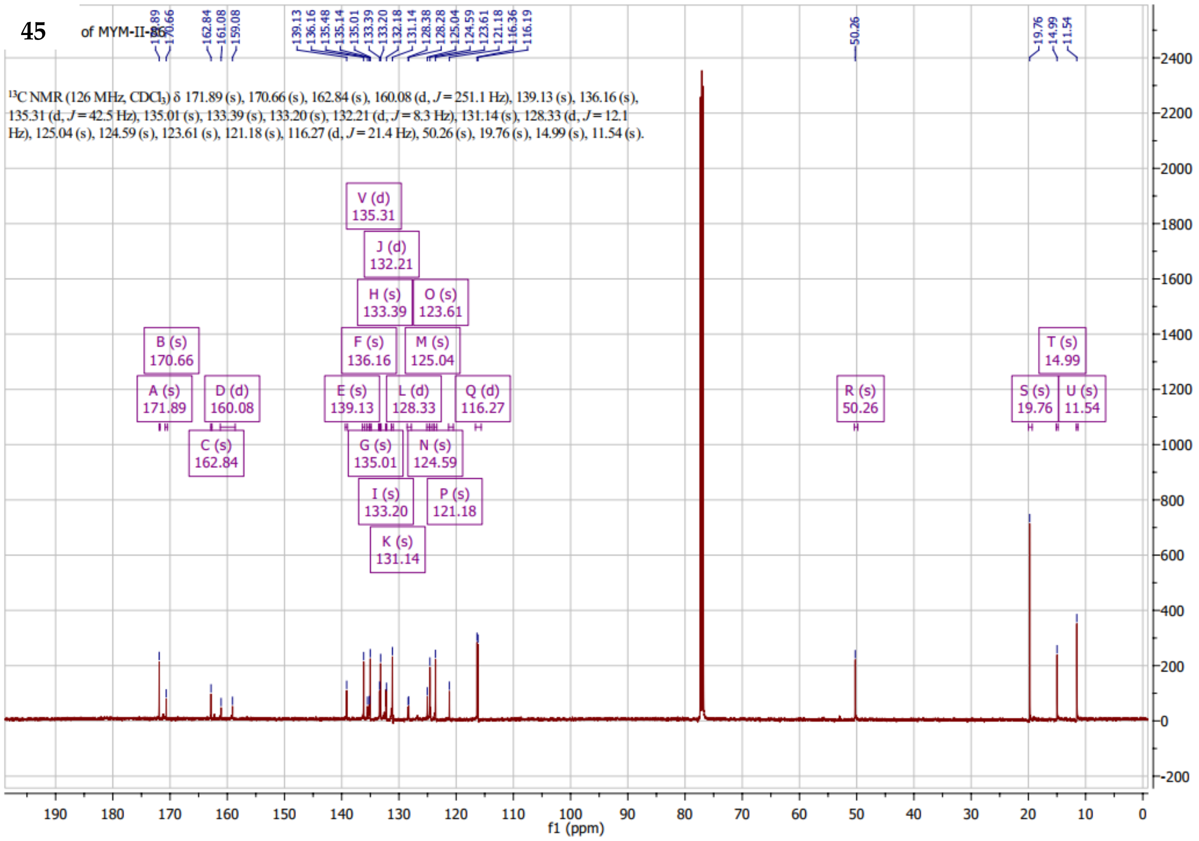

Figure S80. ¹³CNMR spectrum of compound **45**

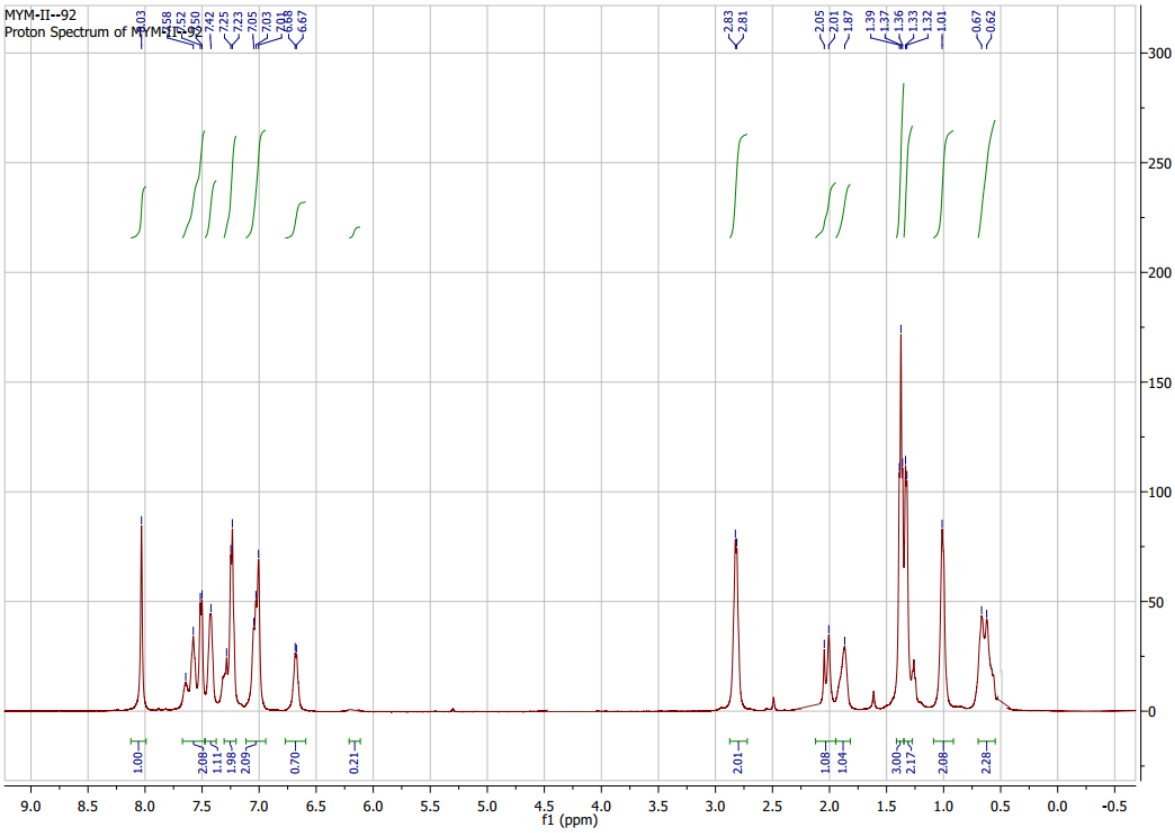

Figure S81. ^{1}HNMR spectrum of compound **46**

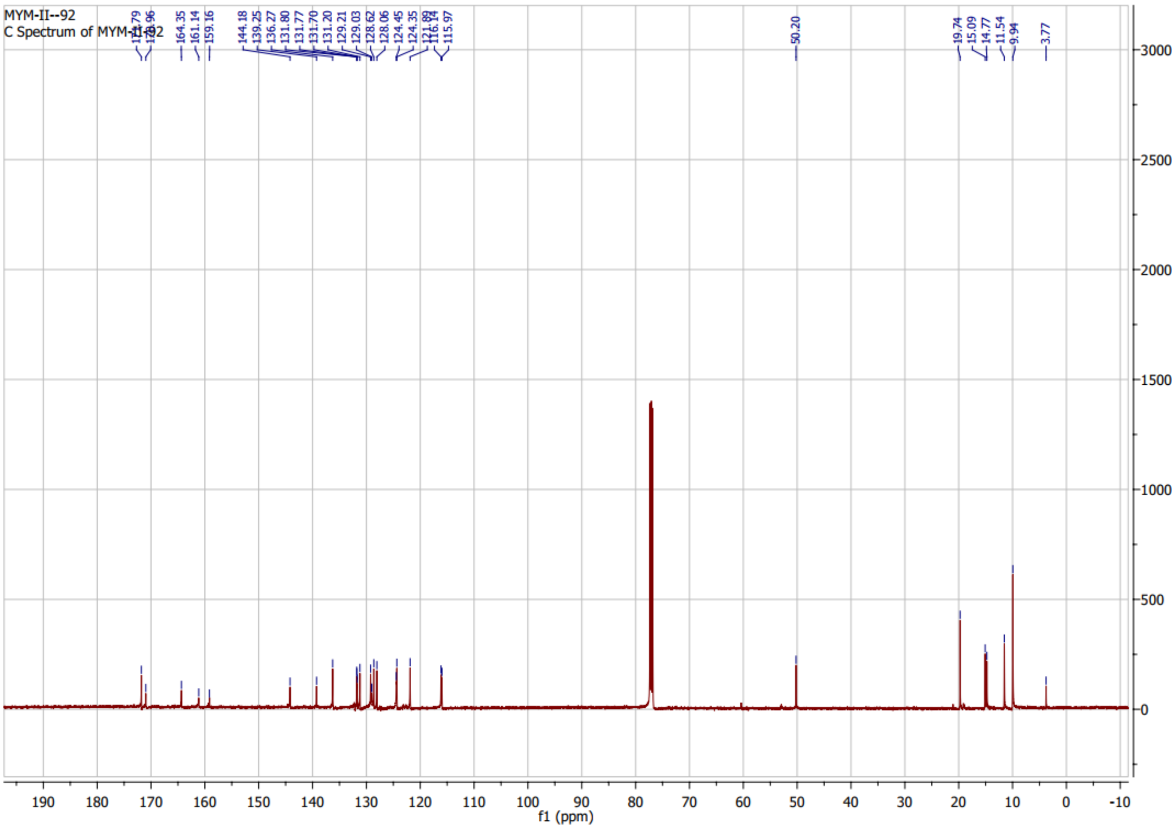

Figure S82. ^{13}CNMR spectrum of compound **46**

Data File: C:\LabSolutions\Data\Vilashini Rajaratnam\08052020 Analysis\MYM-III-32_08052020 HRMS Analysis_2_48.lcd

Elmt	Val.	Min	Max	Elmt	Val.	Min	Max	Elmt	Val.	Min	Max	Elmt	Val.	Min	Max	Use Adduct
H	1	10	25	N	3	0	5	Si	4	0	0	Br	1	0	0	H
2H	1	0	0	O	2	0	4	S	2	0	0	I	3	0	0	NH4
C	4	25	32	F	1	0	2	Cl	1	0	0					

Error Margin (ppm): 100
HC Ratio: 0.0 - 10.0
Max Isotopes: all
MSn Iso RI (%): 75.00

DBE Range: -100.0 - 1000.0
Apply N Rule: yes
Isotope RI (%): 1.00
MSn Logic Mode: AND

Electron Ions: both
Use MSn Info: yes
Isotope Res: 10000
Max Results: 10

Event#: 1 MS(E+) Ret. Time : 0.187 -> 0.253 - 1.600 -> 1.873 Scan# : 29 -> 39 - 241 -> 281

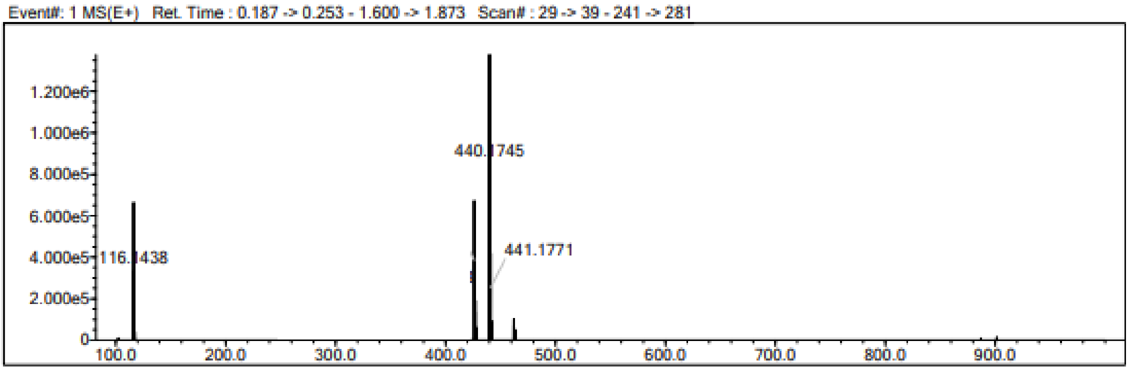

Measured region for 440.1745 m/z

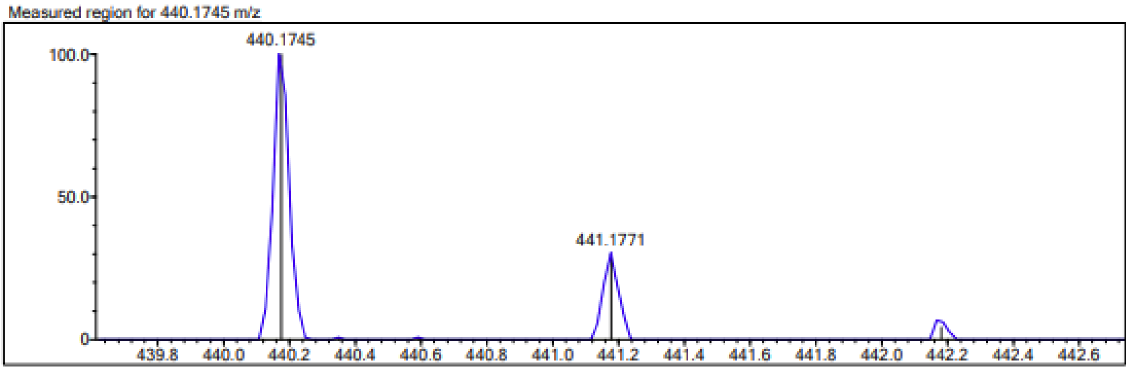

C27 H22 N3 O2 F [M+H]+ : Predicted region for 440.1769 m/z

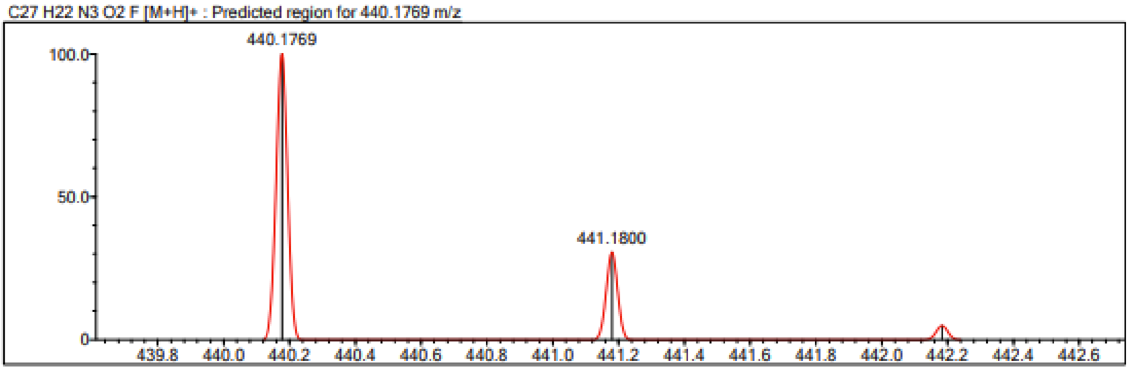

Rank	Score	Formula (M)	Ion	Meas. m/z	Pred. m/z	Df. (mDa)	Df. (ppm)	Iso	DBE
5	64.91	C27 H22 N3 O2 F	[M+H]+	440.1745	440.1769	-2.4	-5.45	75.92	18.0

Figure S83. HRMS spectrum of compound **3**

Data File: C:\LabSolutions\Data\Vilashini Rajaratnam\08052020 Analysis\MYM-III-37_08052020 HRMS Analysis_2_60.lcd

Elmt	Val.	Min	Max	Elmt	Val.	Min	Max	Elmt	Val.	Min	Max	Elmt	Val.	Min	Max	Use Adduct
H	1	8	25	N	3	0	5	Si	4	0	0	Br	1	0	0	H
2H	1	0	0	O	2	0	4	S	2	0	0	I	3	0	0	
C	4	20	25	F	1	0	2	Cl	1	0	2					

Error Margin (ppm):	100	DBE Range:	-100.0 - 1000.0	Electron Ions:	both
HC Ratio:	0.0 - 10.0	Apply N Rule:	yes	Use MSn Info:	yes
Max Isotopes:	all	Isotope RI (%):	1.00	Isotope Res:	10000
MSn Iso RI (%):	75.00	MSn Logic Mode:	AND	Max Results:	10

Event#: 2 MS(E-) Ret. Time : 0.107 -> 0.173 - 1.533 -> 1.890 Scan# : 18 -> 28 - 232 -> 286

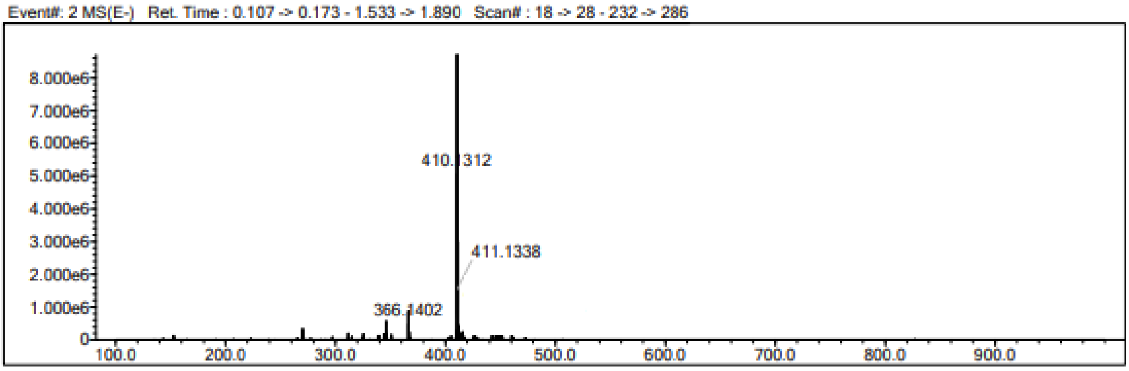

Measured region for 410.1312 m/z

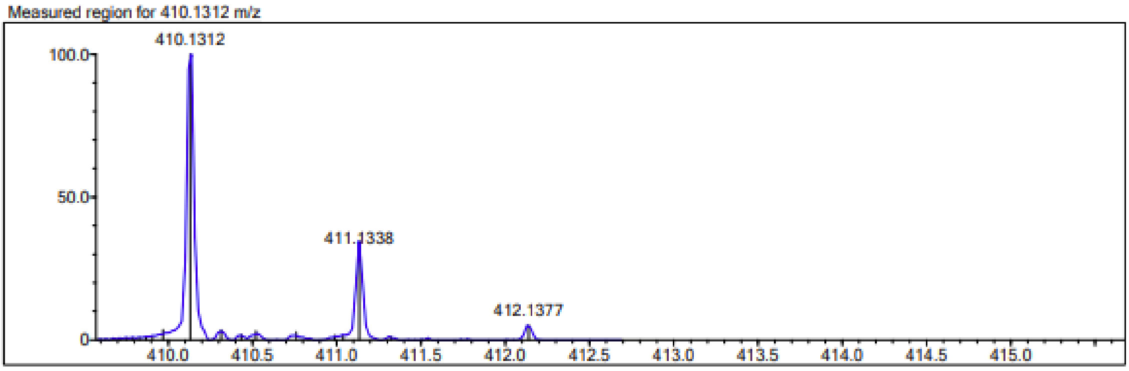

C25 H18 N3 O2 F [M-H]- : Predicted region for 410.1310 m/z

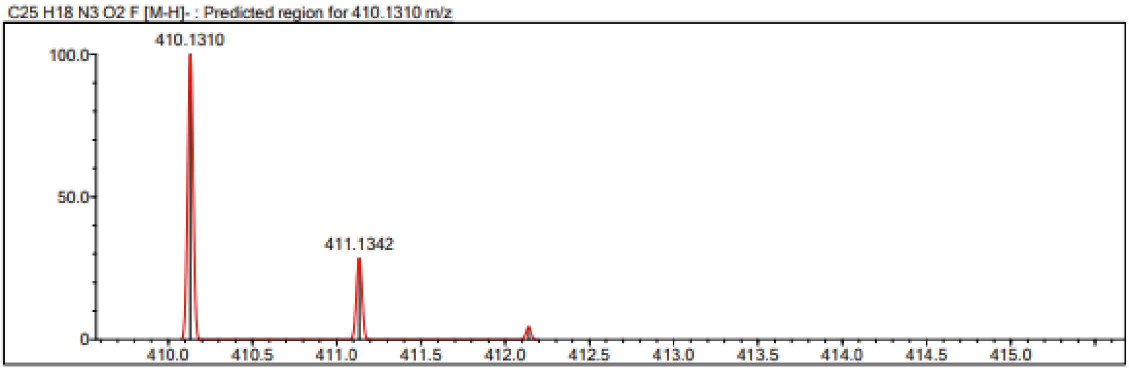

Rank	Score	Formula (M)	Ion	Meas. m/z	Pred. m/z	Df. (mDa)	Df. (ppm)	Iso	DBE
1	91.11	C25 H18 N3 O2 F	[M-H]-	410.1312	410.1310	0.2	0.49	91.11	18.0

Figure S84. HRMS spectrum of compound **4**

Data File: C:\LabSolutions\Data\Vilashini Rajaratnam\08052020 Analysis\MYM-III-41_08052020 HRMS Analysis_2_30.lcd

Elmt	Val.	Min	Max	Elmt	Val.	Min	Max	Elmt	Val.	Min	Max	Elmt	Val.	Min	Max	Use Adduct
H	1	15	25	N	3	2	5	Si	4	0	0	Br	1	0	0	H
2H	1	0	0	O	2	0	2	S	2	0	0	I	3	0	0	NH4
C	4	20	30	F	1	0	2	Cl	1	0	0					

Error Margin (ppm): 100
HC Ratio: 0.0 - 10.0
Max Isotopes: all
MSn Iso RI (%): 75.00

DBE Range: -100.0 - 1000.0
Apply N Rule: yes
Isotope RI (%): 1.00
MSn Logic Mode: AND

Electron Ions: both
Use MSn Info: yes
Isotope Res: 10000
Max Results: 10

Event#: 1 MS(E+) Ret. Time : 0.093 -> 0.107 - 1.640 -> 2.000 Scan# : 15 -> 17 - 247 -> 301

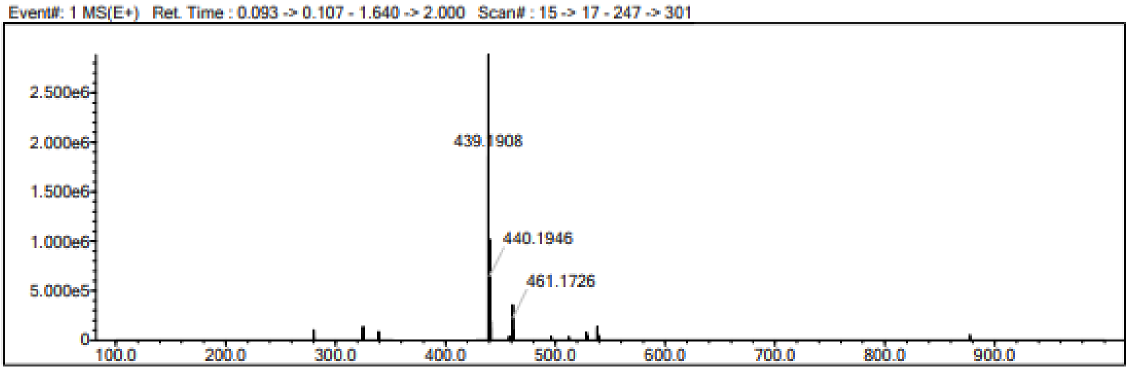

Measured region for 439.1908 m/z

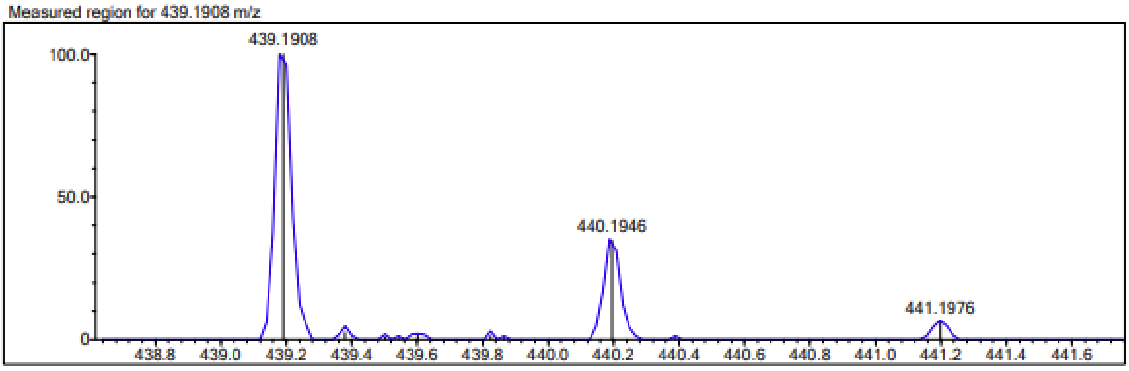

C27 H23 N4 O F [M+H]+ : Predicted region for 439.1929 m/z

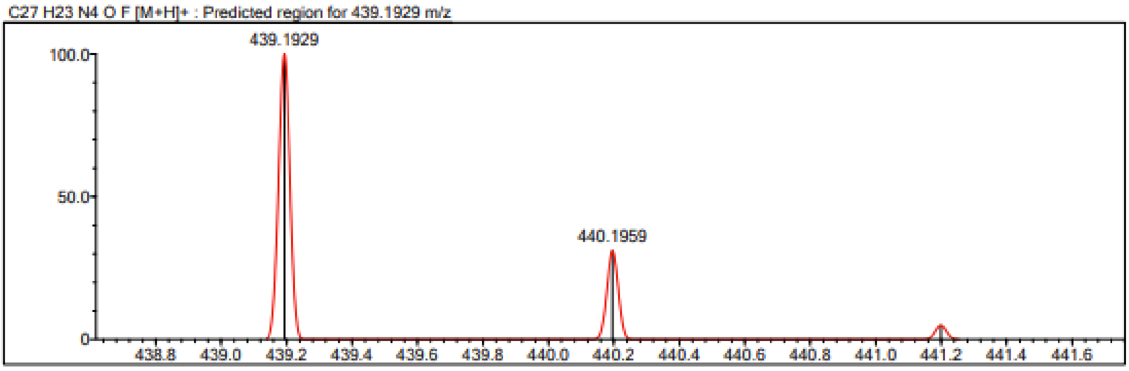

Rank	Score	Formula (M)	Ion	Meas. m/z	Pred. m/z	Df. (mDa)	Df. (ppm)	Iso	DBE
3	85.40	C27 H23 N4 O F	[M+H]+	439.1908	439.1929	-2.1	-4.78	94.31	18.0

Figure S85. HRMS spectrum of compound **5**

Scan positive no column.lcm

Formula Predictor Result	**C16 H12 N2 O Cl Br**
Mass	362.98957
Error Margin	20 ppm
DBE Range	0 - 1000
Electron Ions	Both configurations
HC Ratio	Not Used
Nitrogen Rule	Used

Score	Pred. (M)	Pred. m/z	Meas. m/z	Diff. (mDa)	Formulae (M)	Ion	Diff. (ppm)	Iso Score	DBE
99.63	361.98215	362.98943	362.98957	0.14	C16 H12 N2 O Cl Br	[M+H]+	0.386	99.76	11.0

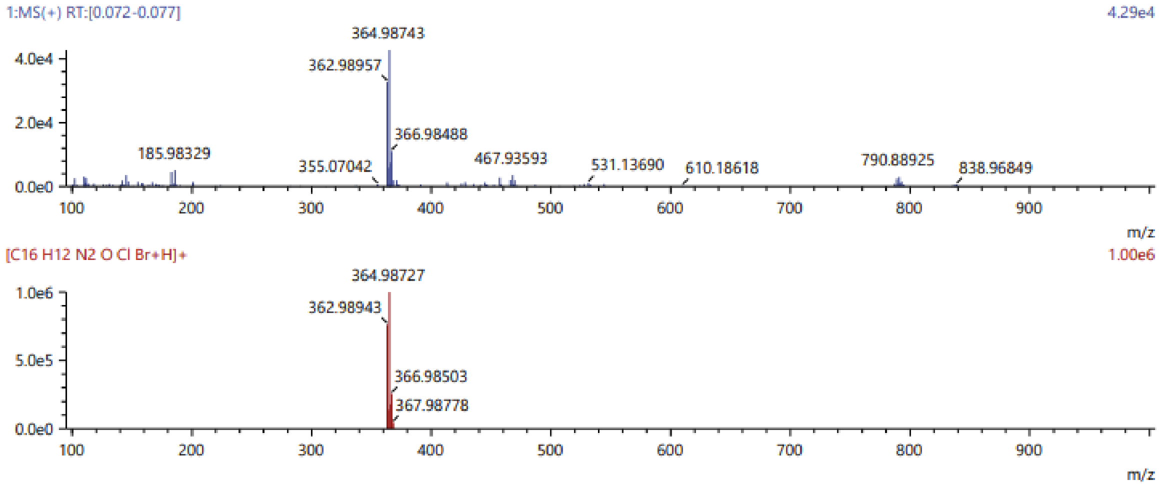

Figure S86. HRMS spectrum of compound 1**2**

HRMS_04262021.lcb

Formula Predictor Result	**C20 H22 N3 O4 Br**
Mass	448.08998
Error Margin	10 ppm
DBE Range	0 - 1000
Electron Ions	Both configurations
HC Ratio	0 - 3
Nitrogen Rule	Used

#	Score	Pred. (M)	Pred. m/z	Meas. m/z	Diff. (mDa)	Formulae (M)	Ion	Diff. (ppm)	Iso Score	DBE
48	40.00	447.07937	448.08665	448.08998	3.33	C20 H22 N3 O4 Br	[M+H]+	7.432	44.44	11.0

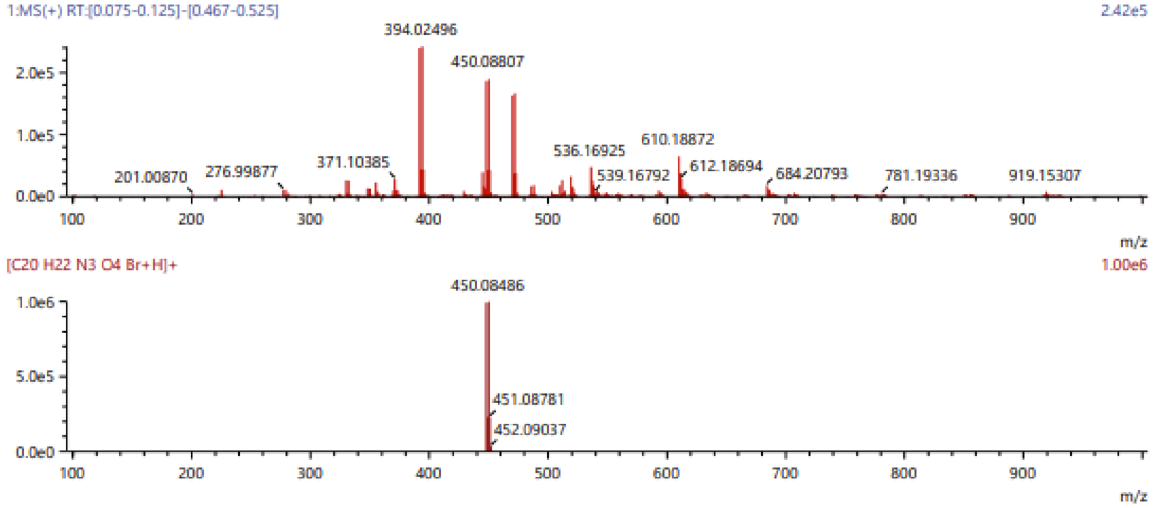

Figure S87. HRMS spectrum of compound 11

HRMS_04262021.lcb

Formula Predictor Result	C15 H12 N3 O Br
Mass	330.02586
Error Margin	10 ppm
DBE Range	0 - 1000
Electron Ions	Both configurations
HC Ratio	0 - 3
Nitrogen Rule	Used

#	Score	Pred. (M)	Pred. m/z	Meas. m/z	Diff. (mDa)	Formulae (M)	Ion	Diff. (ppm)	Iso Score	DBE
21	53.64	329.01637	330.02365	330.02586	2.21	C15 H12 N3 O Br	[M+H]+	6.696	59.60	11.0

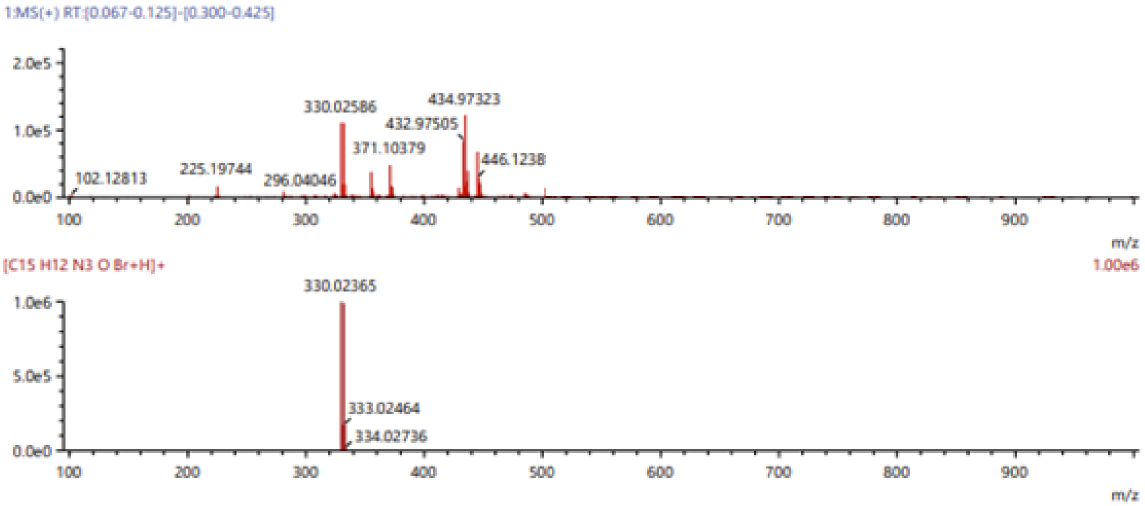

Figure S88. HRMS spectrum of compound 14

Scan positive no column.lcm

Formula Predictor Result	**C21 H17 N3 O2 Cl Br**
Mass	458.02682
Error Margin	20 ppm
DBE Range	0 - 1000
Electron Ions	Both configurations
HC Ratio	Not Used
Nitrogen Rule	Used

Score	Pred. (M)	Pred. m/z	Meas. m/z	Diff. (mDa)	Formulae (M)	Ion	Diff. (ppm)	Iso Score	DBE
99.21	457.01927	458.02654	458.02682	0.28	C21 H17 N3 O2 Cl Br	[M+H]+	0.611	99.41	14.0

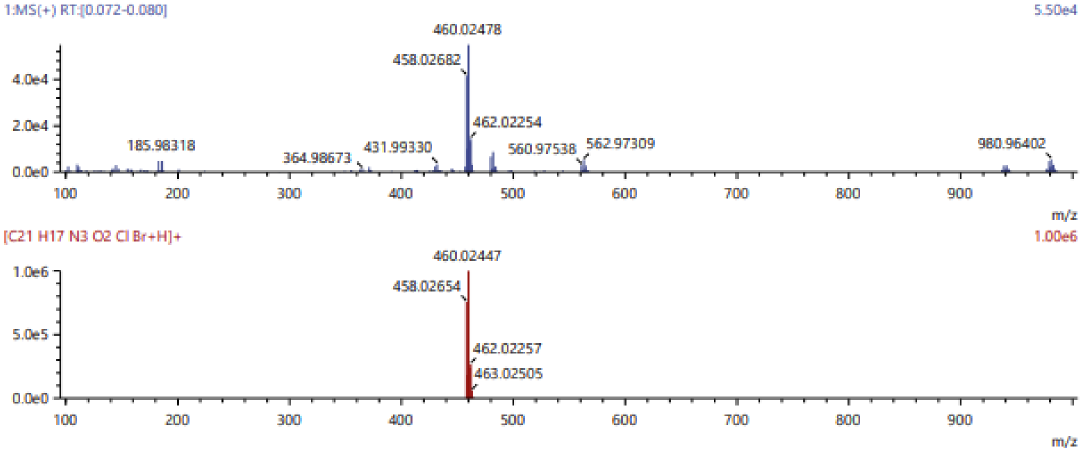

Figure S89. HRMS spectrum of compound 1**5**

HRMS_04262021.lcb

Formula Predictor Result	**C20 H17 N4 O2 Br**
Mass	425.064
Error Margin	10 ppm
DBE Range	0 - 1000
Electron Ions	Both configurations
HC Ratio	0 - 3
Nitrogen Rule	Used

#	Score	Pred. (M)	Pred. m/z	Meas. m/z	Diff. (mDa)	Formulae (M)	Ion	Diff. (ppm)	Iso Score	DBE
94	40.00	424.05349	425.06076	425.06400	3.24	C20 H17 N4 O2 Br	[M+H]+	7.622	44.44	14.0

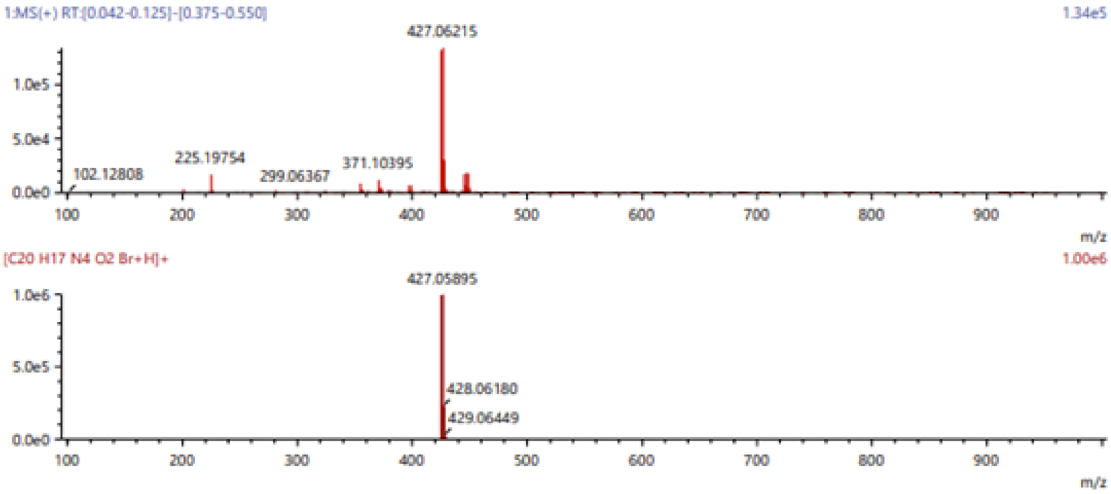

Figure S90. HRMS spectrum of compound 17

Scan positive no column.lcm

Formula Predictor Result	**C32 H38 N3 O2 Si Cl**
Mass	560.25004
Error Margin	20 ppm
DBE Range	0 - 1000
Electron Ions	Both configurations
HC Ratio	Not Used
Nitrogen Rule	Used

Score	Pred. (M)	Pred. m/z	Meas. m/z	Diff. (mDa)	Formulae (M)	Ion	Diff. (ppm)	Iso Score	DBE
95.82	559.24218	560.24946	560.25004	0.58	C32 H38 N3 O2 Si Cl	[M+H]+	1.035	95.88	15.0

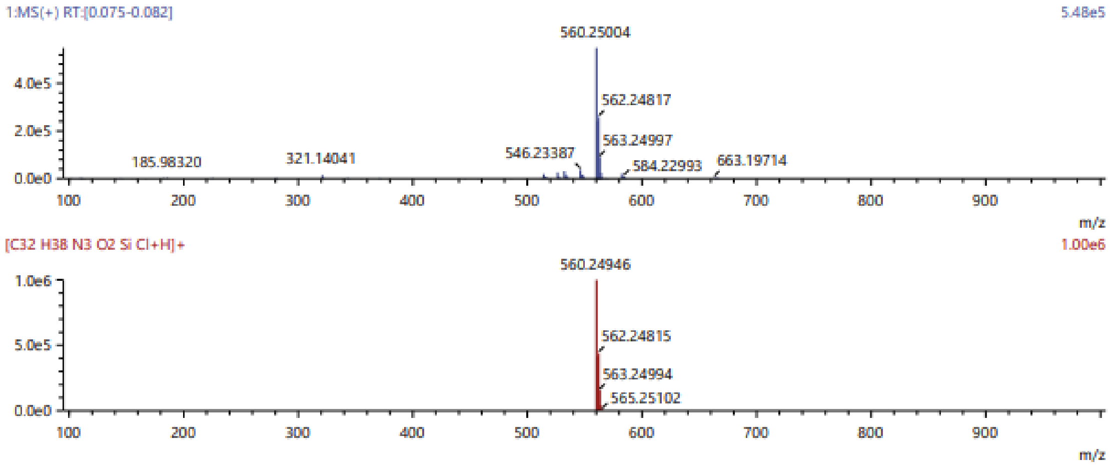

Figure S91. HRMS spectrum of compound 18

Data File: C:\LabSolutions\Data\Vilashini Rajaratnam\08052020 Analysis\MYM-III-16_08052020 HRMS Analysis_2_56.lcd

Elmt	Val.	Min	Max	Elmt	Val.	Min	Max	Elmt	Val.	Min	Max	Elmt	Val.	Min	Max	Use Adduct
H	1	8	25	N	3	0	5	Si	4	0	0	Br	1	0	0	H
2H	1	0	0	O	2	0	4	S	2	0	0	I	3	0	0	NH4
C	4	20	25	F	1	0	2	Cl	1	0	2					

Error Margin (ppm):	100	DBE Range:	-100.0 - 1000.0	Electron Ions:	both
HC Ratio:	0.0 - 10.0	Apply N Rule:	yes	Use MSn Info:	yes
Max Isotopes:	all	Isotope RI (%):	1.00	Isotope Res:	10000
MSn Iso RI (%):	75.00	MSn Logic Mode:	AND	Max Results:	10

Event#: 1 MS(E+) Ret. Time : 0.093 -> 0.120 - 1.627 -> 1.917 Scan# : 15 -> 19 - 245 -> 289

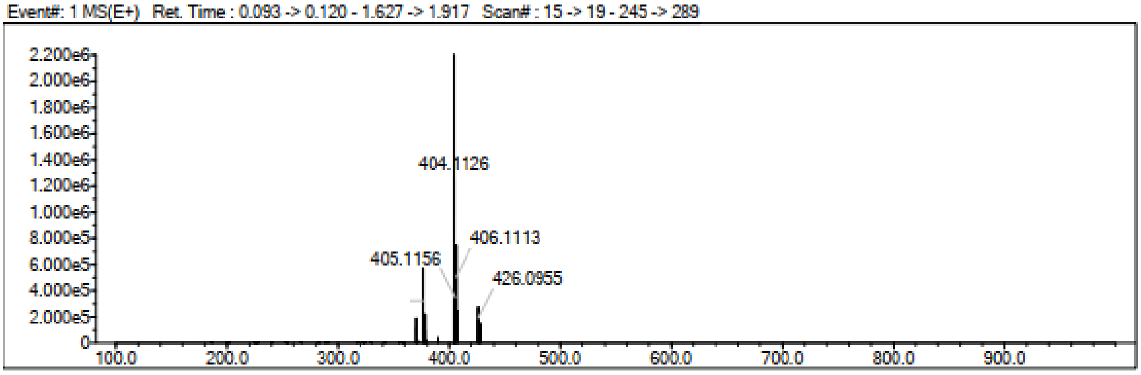

Measured region for 404.1126 m/z

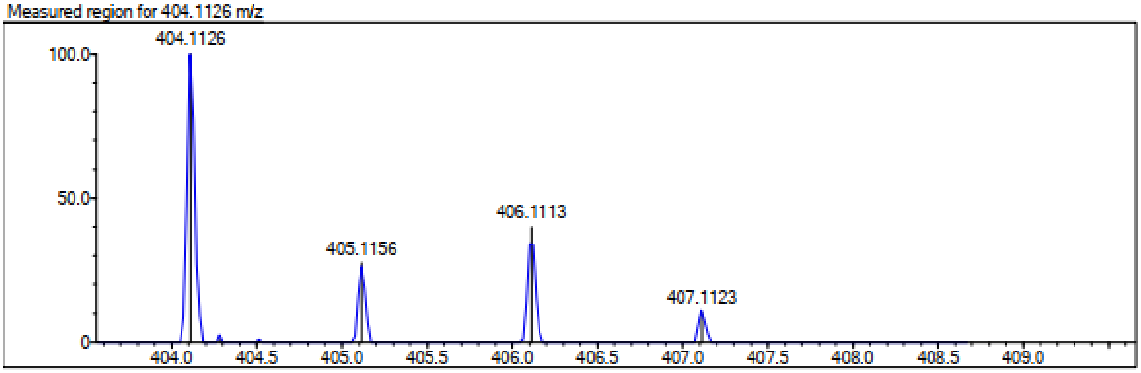

C23 H18 N3 O2 Cl [M+H]+ : Predicted region for 404.1160 m/z

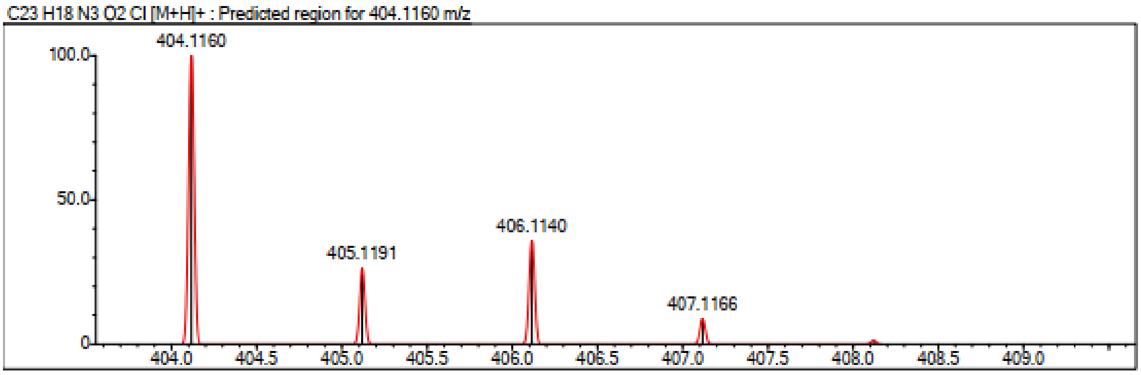

Rank	Score	Formula (M)	Ion	Meas. m/z	Pred. m/z	Df. (mDa)	Df. (ppm)	Iso	DBE
1	37.91	C23 H18 N3 O2 Cl	[M+H]+	404.1126	404.1160	-3.4	-8.41	67.82	16.0

Figure S92. HRMS spectrum of compound **18A**

HRMS_04262021.lcb

Formula Predictor Result	**C22 H18 N4 O2**
Mass	371.15321
Error Margin	10 ppm
DBE Range	0 - 1000
Electron Ions	Both configurations
HC Ratio	0 - 3
Nitrogen Rule	Used

#	Score	Pred. (M)	Pred. m/z	Meas. m/z	Diff. (mDa)	Formulae (M)	Ion	Diff. (ppm)	Iso Score	DBE
23	44.40	370.14298	371.15025	371.15321	2.96	C22 H18 N4 O2	[M+H]+	7.975	49.33	16.0

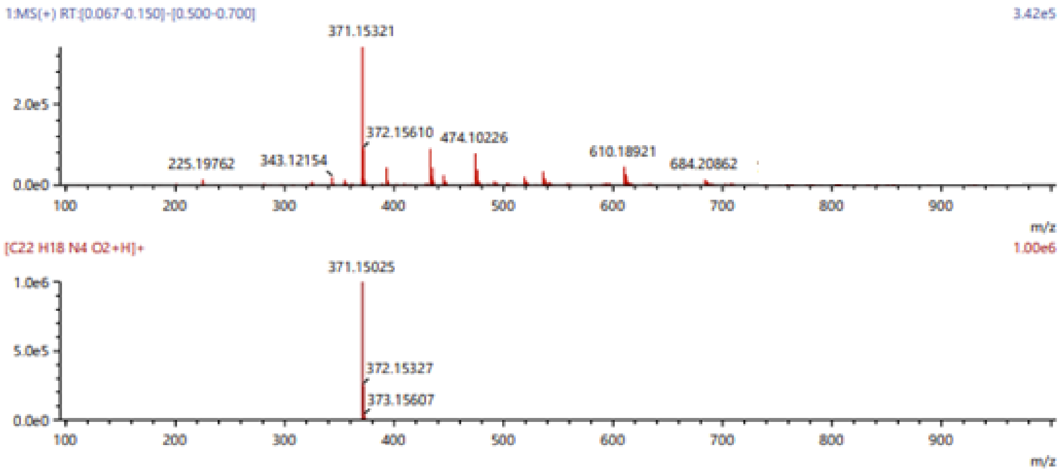

Figure S93. HRMS spectrum of compound 20**A**

Data File: C:\LabSolutions\Data\Vilashini Rajaratnam\08052020 Analysis\MYM-III-22_08052020 HRMS Analysis_2_50.lcd

Elmt	Val.	Min	Max	Elmt	Val.	Min	Max	Elmt	Val.	Min	Max	Elmt	Val.	Min	Max	Use Adduct
H	1	10	25	N	3	0	5	Si	4	0	0	Br	1	0	0	H
2H	1	0	0	O	2	0	4	S	2	0	0	I	3	0	0	NH4
C	4	20	25	F	1	0	0	Cl	1	0	2					

Error Margin (ppm): 100
HC Ratio: 0.0 - 10.0
Max Isotopes: all
MSn Iso RI (%): 75.00

DBE Range: -100.0 - 1000.0
Apply N Rule: yes
Isotope RI (%): 1.00
MSn Logic Mode: AND

Electron Ions: both
Use MSn Info: yes
Isotope Res: 10000
Max Results: 10

Event#: 1 MS(E+) Ret. Time : 0.093 -> 0.093 - 0.187 -> 0.205 Scan# : 15 -> 15 - 29 -> 31

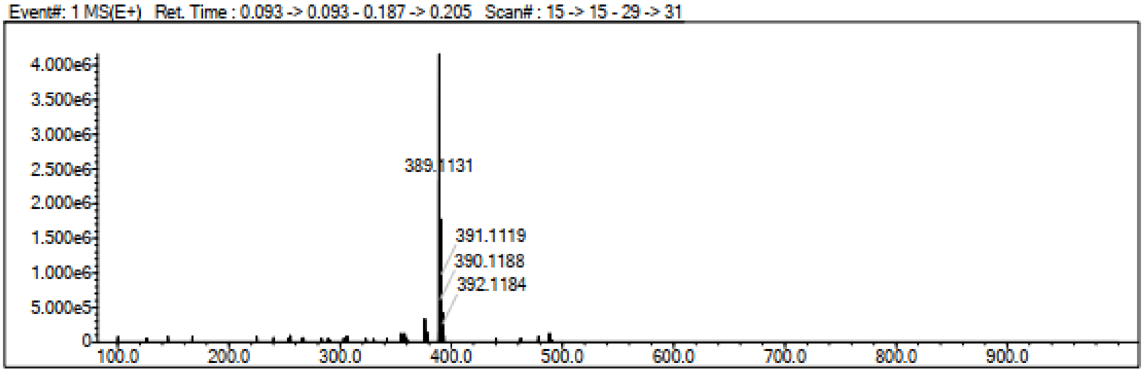

Measured region for 389.1131 m/z

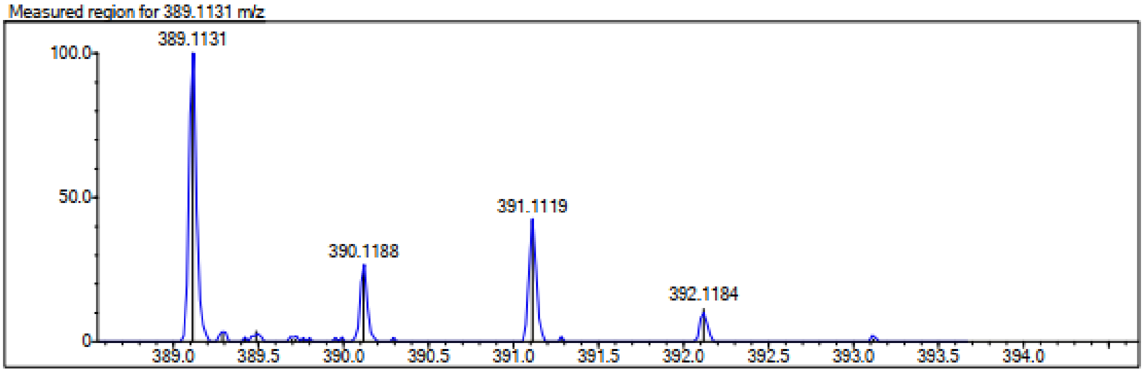

C22 H17 N4 O Cl [M+H]+ : Predicted region for 389.1164 m/z

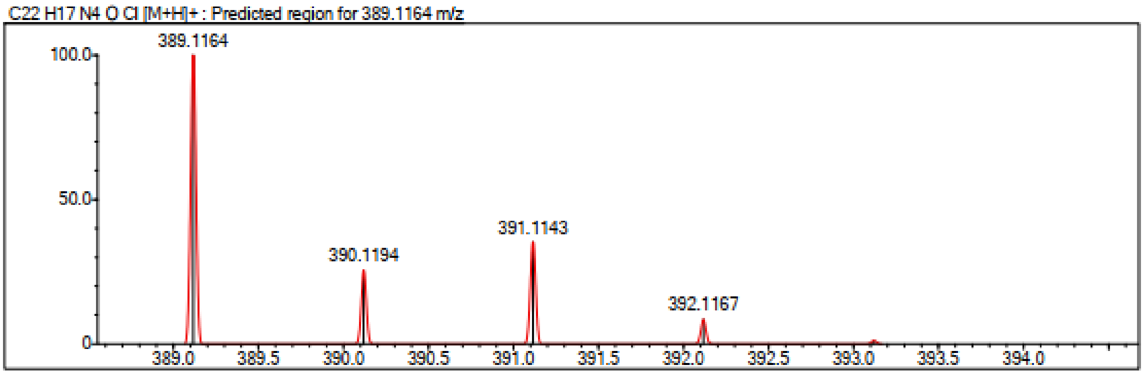

Rank	Score	Formula (M)	Ion	Meas. m/z	Pred. m/z	Df. (mDa)	Df. (ppm)	Iso	DBE
1	49.38	C22 H17 N4 O Cl	[M+H]+	389.1131	389.1164	-3.3	-8.48	89.46	16.0

Figure S94. HRMS spectrum of compound 21

Data File: C:\LabSolutions\Data\Vilashini Rajaratnam\08052020 Analysis\MYM-III-29_08052020 HRMS Analysis_2_58.lcd

Elmt	Val.	Min	Max	Elmt	Val.	Min	Max	Elmt	Val.	Min	Max	Elmt	Val.	Min	Max	Use Adduct
H	1	8	25	N	3	0	5	Si	4	0	0	Br	1	0	0	H
2H	1	0	0	O	2	0	4	S	2	0	0	I	3	0	0	NH4
C	4	20	25	F	1	0	0	Cl	1	0	2					

Error Margin (ppm): 100
HC Ratio: 0.0 - 10.0
Max Isotopes: all
MSn Iso RI (%): 75.00

DBE Range: -100.0 - 1000.0
Apply N Rule: yes
Isotope RI (%): 1.00
MSn Logic Mode: AND

Electron Ions: both
Use MSn Info: yes
Isotope Res: 10000
Max Results: 10

Event#: 1 MS(E+) Ret. Time : 0.387 -> 0.680 - 1.680 -> 1.858 Scan# : 59 -> 103 - 253 -> 279

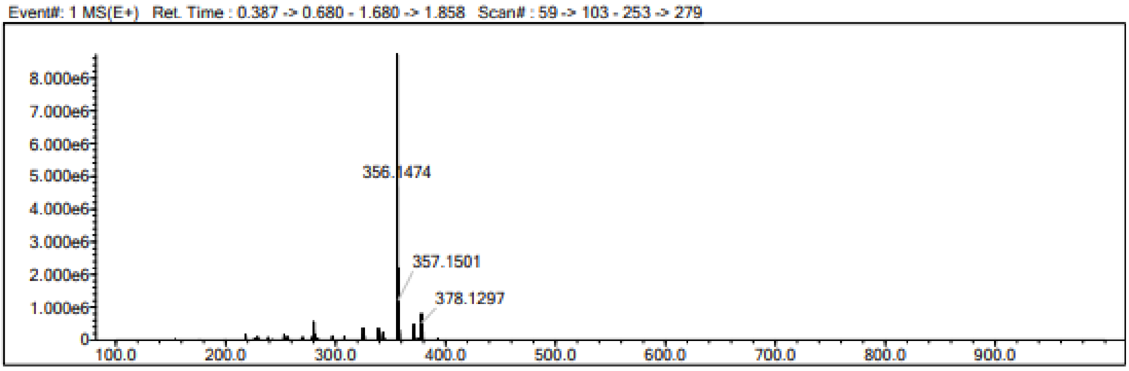

Measured region for 356.1474 m/z

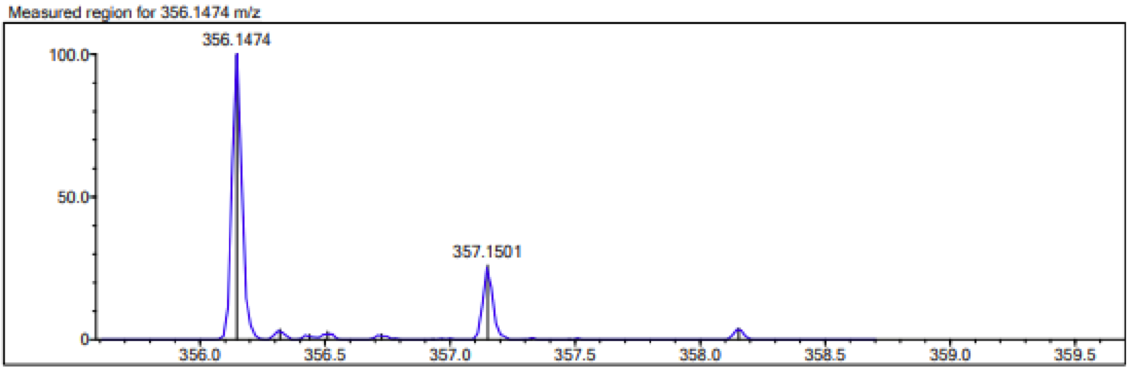

C21 H17 N5 O [M+H]+ : Predicted region for 356.1506 m/z

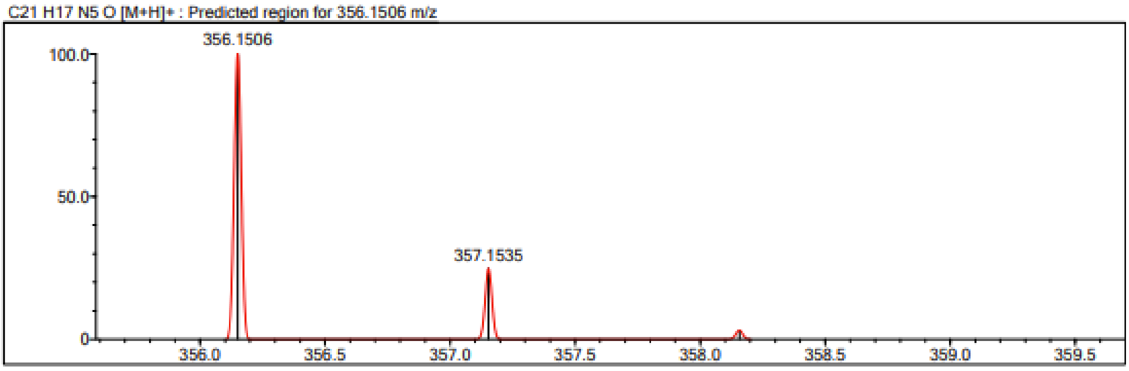

Rank	Score	Formula (M)	Ion	Meas. m/z	Pred. m/z	Df. (mDa)	Df. (ppm)	Iso	DBE
1	43.58	C21 H17 N5 O	[M+H]+	356.1474	356.1506	-3.2	-8.99	86.99	16.0

Figure S95. HRMS spectrum of compound 23

Scan positive no column.lcm

Formula Predictor Result	**C24 H21 N4 O Cl**
Mass	417.14797
Error Margin	20 ppm
DBE Range	0 - 1000
Electron Ions	Both configurations
HC Ratio	Not Used
Nitrogen Rule	Used

Score	Pred. (M)	Pred. m/z	Meas. m/z	Diff. (mDa)	Formulae (M)	Ion	Diff. (ppm)	Iso Score	DBE
99.43	416.14039	417.14767	417.14797	0.30	C24 H21 N4 O Cl	[M+H]+	0.719	99.71	16.0

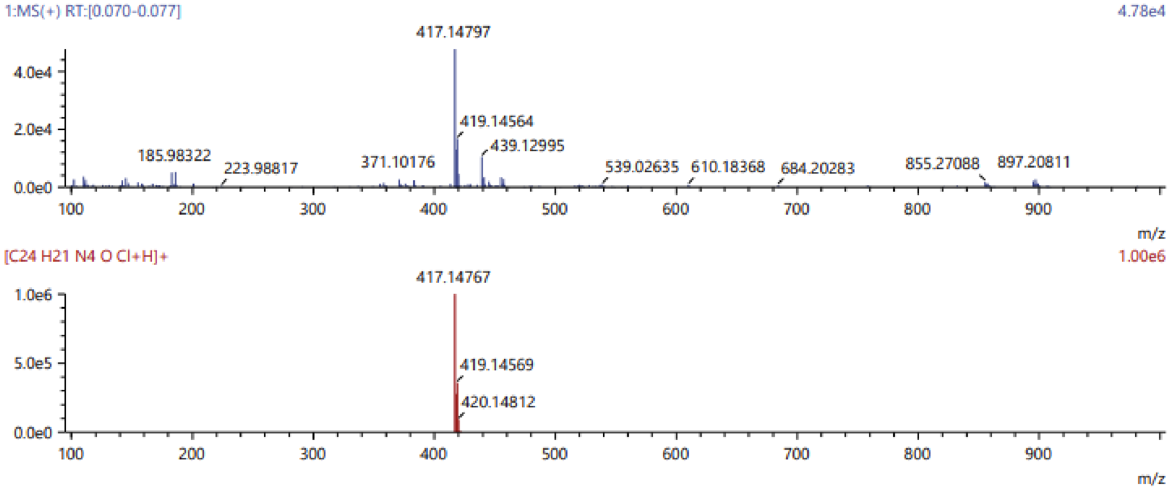

Figure S96. HRMS spectrum of compound 29

Data File: C:\LabSolutions\Data\Vilashini Rajaratnam\08052020 Analysis\MYM-III-25_08052020 HRMS Analysis_2_42.lcd

Elmt	Val.	Min	Max	Elmt	Val.	Min	Max	Elmt	Val.	Min	Max	Elmt	Val.	Min	Max	Use Adduct
H	1	10	25	N	3	0	5	Si	4	0	0	Br	1	0	0	H
2H	1	0	0	O	2	0	4	S	2	0	0	I	3	0	0	NH4
C	4	15	25	F	1	0	0	Cl	1	0	0					

Error Margin (ppm): 100
HC Ratio: 0.0 - 10.0
Max Isotopes: all
MSn Iso RI (%): 75.00

DBE Range: -100.0 - 1000.0
Apply N Rule: yes
Isotope RI (%): 1.00
MSn Logic Mode: AND

Electron Ions: both
Use MSn Info: yes
Isotope Res: 10000
Max Results: 10

Event#: 1 MS(E+) Ret. Time : 0.080 -> 0.107 - 1.200 -> 1.638 Scan# : 13 -> 17 - 181 -> 247

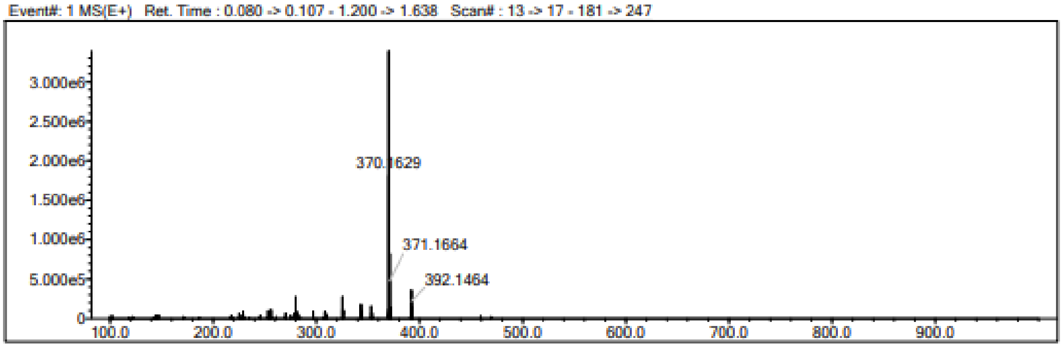

Measured region for 370.1629 m/z

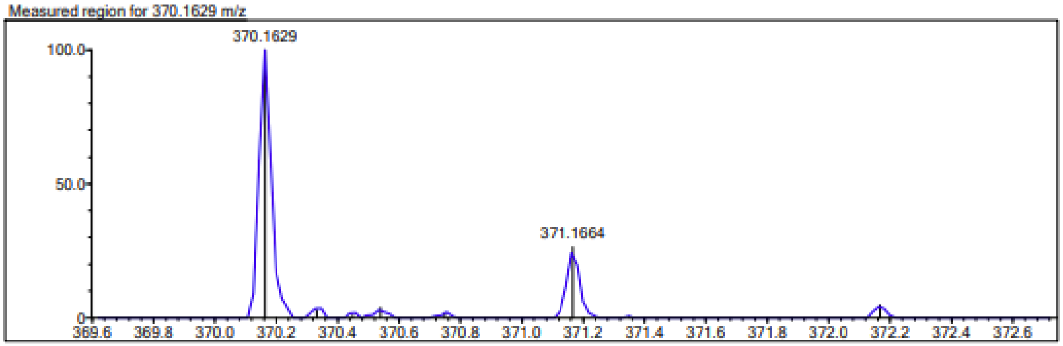

C22 H19 N5 O [M+H]+ : Predicted region for 370.1662 m/z

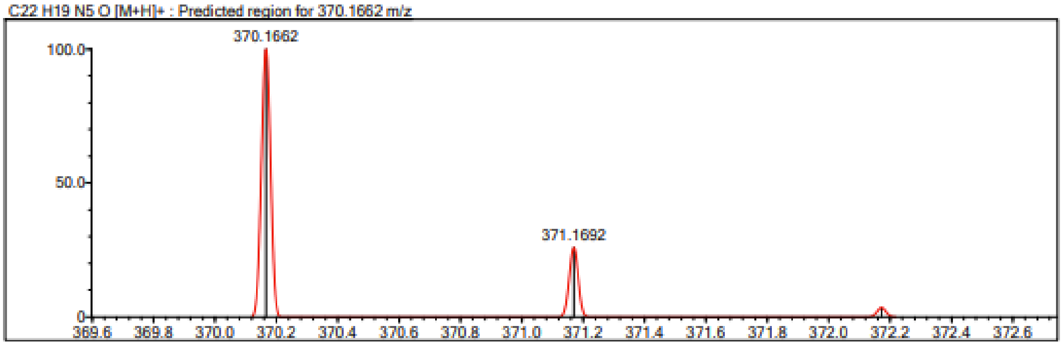

Rank	Score	Formula (M)	Ion	Meas. m/z	Pred. m/z	Df. (mDa)	Df. (ppm)	Iso	DBE
1	36.77	C22 H19 N5 O	[M+H]+	370.1629	370.1662	-3.3	-8.91	72.25	16.0

Figure S97. HRMS spectrum of compound 31

HRMS_04262021.lcb

Formula Predictor Result	**C21 H18 N4 O F Cl**
Mass	397.12599
Error Margin	10 ppm
DBE Range	0 - 1000
Electron Ions	Both configurations
HC Ratio	0 - 3
Nitrogen Rule	Used

#	Score	Pred. (M)	Pred. m/z	Meas. m/z	Diff. (mDa)	Formulae (M)	Ion	Diff. (ppm)	Iso Score	DBE
201	1.05	396.11532	397.12259	397.12599	3.40	C21 H18 N4 O F Cl	[M+H]+	8.562	1.16	14.0

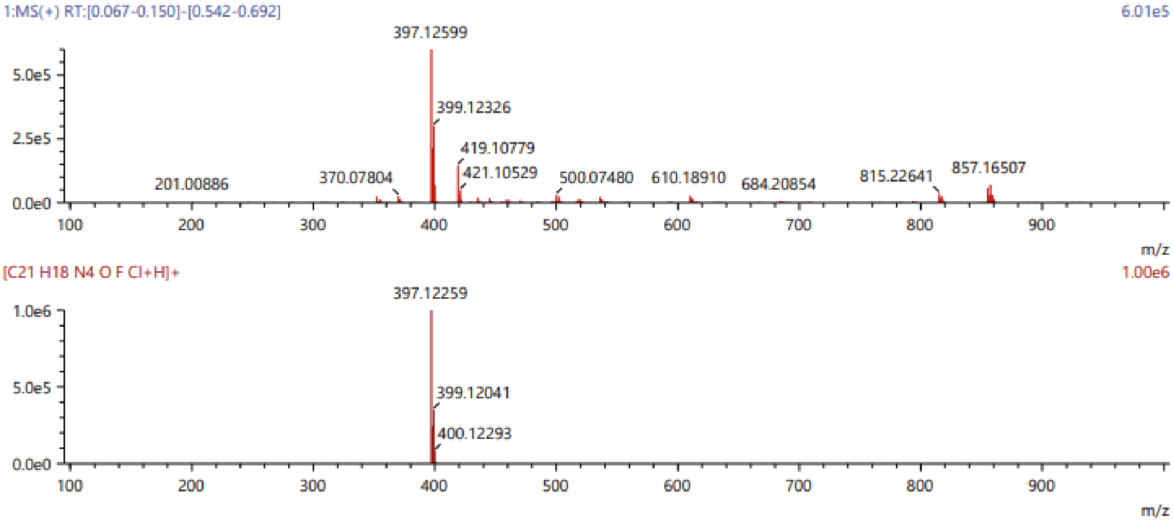

Figure S98. HRMS spectrum of compound 32

Scan positive no column.lcm

Formula Predictor Result	**C28 H22 N5 O F**
Mass	464.18855
Error Margin	20 ppm
DBE Range	0 - 1000
Electron Ions	Both configurations
HC Ratio	Not Used
Nitrogen Rule	Used

Score	Pred. (M)	Pred. m/z	Meas. m/z	Diff. (mDa)	Formulae (M)	Ion	Diff. (ppm)	Iso Score	DBE
98.76	463.18084	464.18811	464.18855	0.44	C28 H22 N5 O F	[M+H]+	0.948	99.10	20.0

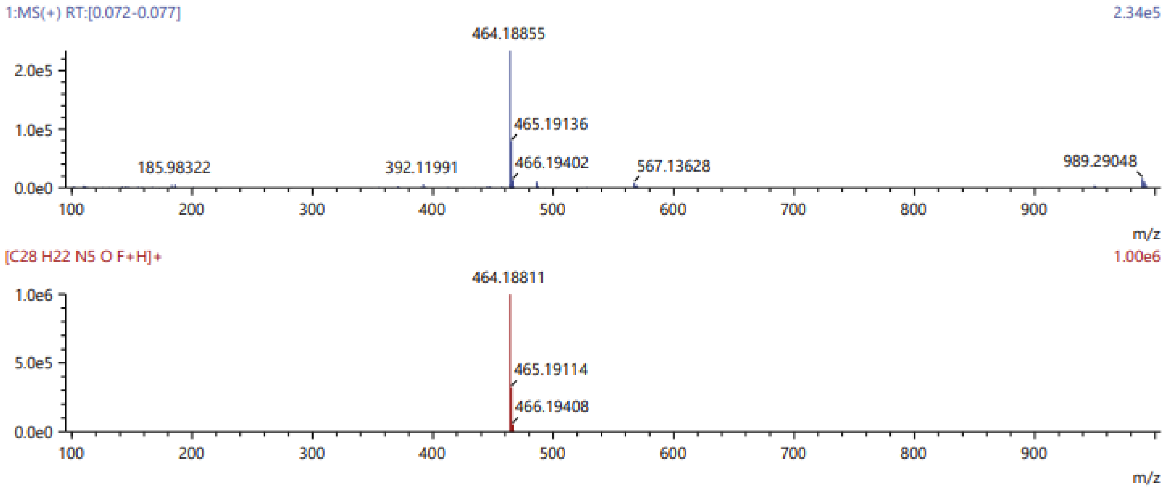

Figure S99. HRMS spectrum of compound 35

Data File: C:\LabSolutions\Data\Vilashini Rajaratnam\08052020 Analysis\MYM-III-36_08052020 HRMS Analysis_2_54.lcd

Elmt	Val.	Min	Max	Elmt	Val.	Min	Max	Elmt	Val.	Min	Max	Elmt	Val.	Min	Max	Use Adduct
H	1	8	25	N	3	0	5	Si	4	0	0	Br	1	0	0	H
2H	1	0	0	O	2	0	4	S	2	0	0	I	3	0	0	NH4
C	4	20	25	F	1	0	2	Cl	1	0	2					

Error Margin (ppm): 100
HC Ratio: 0.0 - 10.0
Max Isotopes: all
MSn Iso RI (%): 75.00

DBE Range: -100.0 - 1000.0
Apply N Rule: yes
Isotope RI (%): 1.00
MSn Logic Mode: AND

Electron Ions: both
Use MSn Info: yes
Isotope Res: 10000
Max Results: 10

Event#: 1 MS(E+) Ret. Time : 0.120 -> 0.240 - 1.387 -> 1.799 Scan# : 19 -> 37 - 209 -> 271

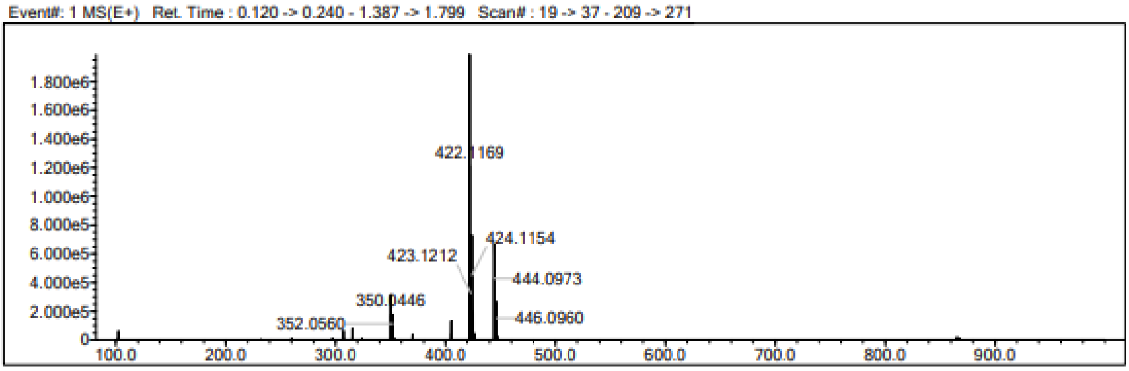

Measured region for 422.1169 m/z

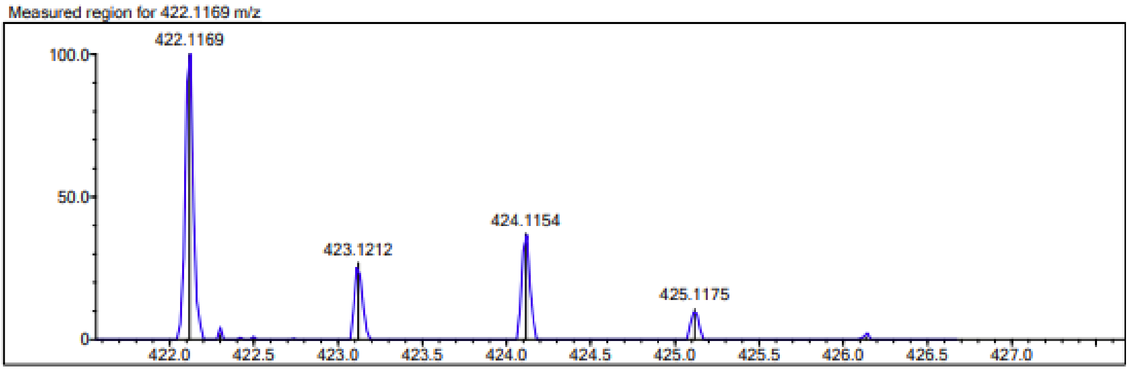

C22 H17 N5 O F Cl [M+H]+ : Predicted region for 422.1178 m/z

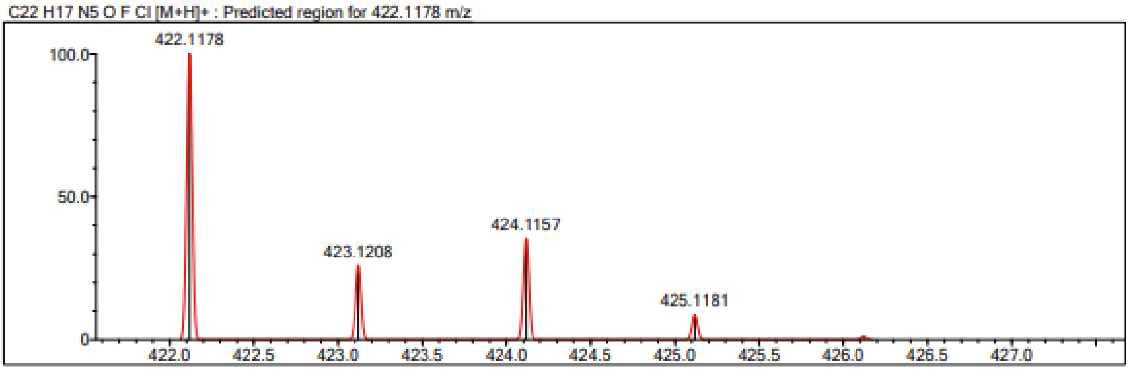

Rank	Score	Formula (M)	Ion	Meas. m/z	Pred. m/z	Df. (mDa)	Df. (ppm)	Iso	DBE
1	69.87	C22 H17 N5 O F Cl	[M+H]+	422.1169	422.1178	-0.9	-2.13	71.90	16.0

Figure S100. HRMS spectrum of compound 36

HRMS_04262021.lcb

Formula Predictor Result	**C23 H19 N5 O F Cl**
Mass	436.13746
Error Margin	10 ppm
DBE Range	0 - 1000
Electron Ions	Both configurations
HC Ratio	0 - 3
Nitrogen Rule	Used

#	Score	Pred. (M)	Pred. m/z	Meas. m/z	Diff. (mDa)	Formulae (M)	Ion	Diff. (ppm)	Iso Score	DBE
204	4.64	435.12622	436.13349	436.13746	3.97	C23 H19 N5 O F Cl	[M+H]+	9.103	5.16	16.0

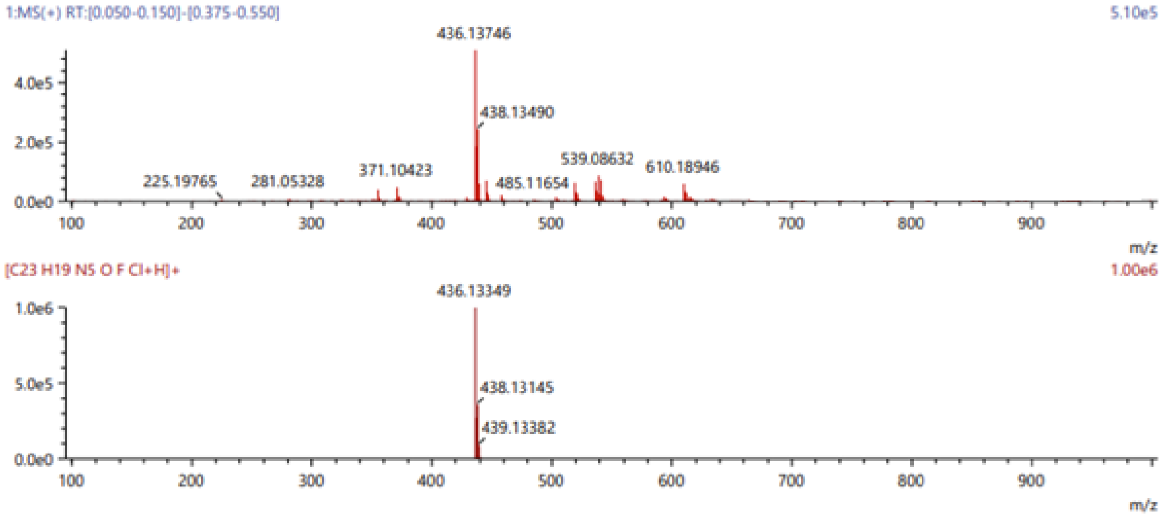

Figure S101. HRMS spectrum of compound 37

HRMS_04262021.lcb

Formula Predictor Result	**C23 H17 N5 O F Cl**
Mass	434.121
Error Margin	10 ppm
DBE Range	0 - 1000
Electron Ions	Both configurations
HC Ratio	0 - 3
Nitrogen Rule	Used

#	Score	Pred. (M)	Pred. m/z	Meas. m/z	Diff. (mDa)	Formulae (M)	Ion	Diff. (ppm)	Iso Score	DBE
89	5.44	433.11057	434.11784	434.12100	3.16	C23 H17 N5 O F Cl	[M+H]+	7.279	6.05	17.0

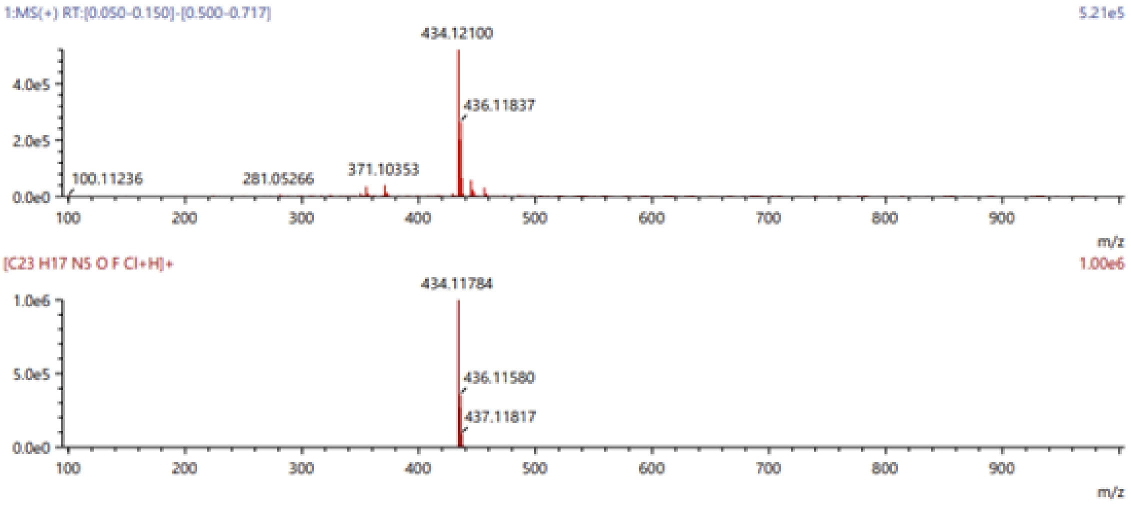

Figure S102. HRMS spectrum of compound 38

Scan positive no column.lcm

Formula Predictor Result	**C25 H18 N5 O F**
Mass	424.15731
Error Margin	20 ppm
DBE Range	0 - 1000
Electron Ions	Both configurations
HC Ratio	Not Used
Nitrogen Rule	Used

Score	Pred. (M)	Pred. m/z	Meas. m/z	Diff. (mDa)	Formulae (M)	Ion	Diff. (ppm)	Iso Score	DBE
97.30	423.14954	424.15681	424.15731	0.50	C25 H18 N5 O F	[M+H]+	1.179	97.61	19.0

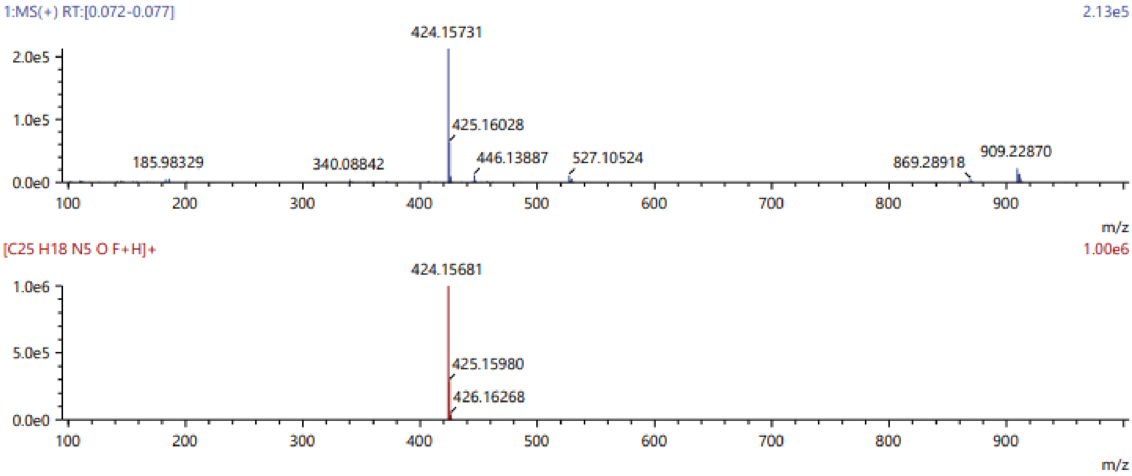

Figure S103. HRMS spectrum of compound 39

Scan positive no column.lcm

Formula Predictor Result	**C23 H18 N6 O**
Mass	395.16219
Error Margin	20 ppm
DBE Range	0 - 1000
Electron Ions	Both configurations
HC Ratio	Not Used
Nitrogen Rule	Used

Score	Pred. (M)	Pred. m/z	Meas. m/z	Diff. (mDa)	Formulae (M)	Ion	Diff. (ppm)	Iso Score	DBE
70.65	394.15421	395.16149	395.16219	0.70	C23 H18 N6 O	[M+H]+	1.771	68.41	18.0

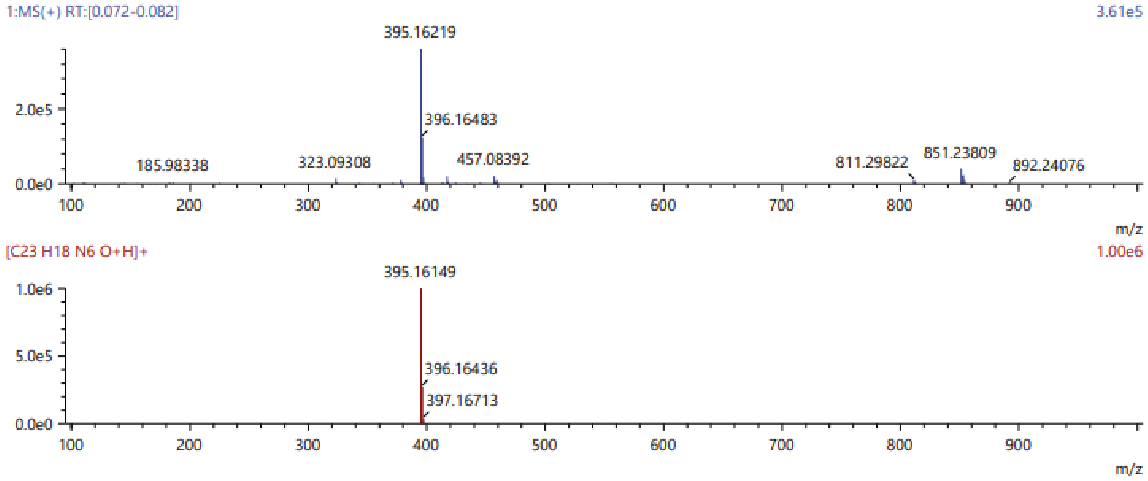

Figure S104. HRMS spectrum of compound 40

Data File: C:\LabSolutions\Data\Vilashini Rajaratnam\08052020 Analysis\MYM-III-36A_08052020 HRMS Analysis_2_36.lcd

Elmt	Val.	Min	Max	Elmt	Val.	Min	Max	Elmt	Val.	Min	Max	Elmt	Val.	Min	Max	Use Adduct
H	1	10	25	N	3	3	6	Si	4	0	0	Br	1	0	0	H
2H	1	0	0	O	2	0	4	S	2	0	0	I	3	0	0	NH4
C	4	20	25	F	1	0	2	Cl	1	0	2					

Error Margin (ppm): 100
HC Ratio: 0.0 - 10.0
Max Isotopes: all
MSn Iso RI (%): 75.00

DBE Range: -100.0 - 1000.0
Apply N Rule: yes
Isotope RI (%): 1.00
MSn Logic Mode: AND

Electron Ions: both
Use MSn Info: yes
Isotope Res: 10000
Max Results: 10

Event#: 1 MS(E+) Ret. Time : 0.107 -> 0.240 - 1.707 -> 1.869 Scan# : 17 -> 37 - 257 -> 281

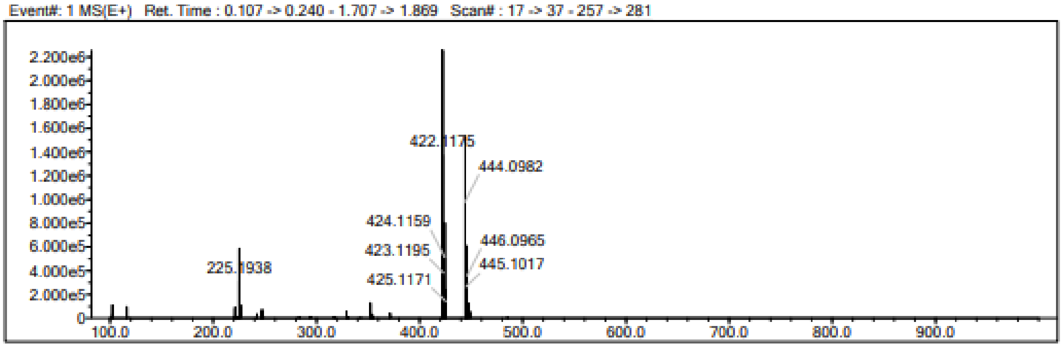

Measured region for 422.1175 m/z

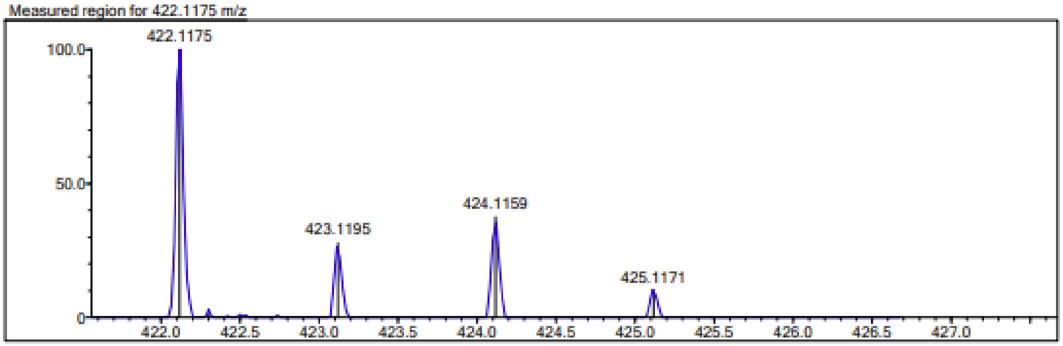

C22 H17 N5 O F Cl [M+H]+ : Predicted region for 422.1178 m/z

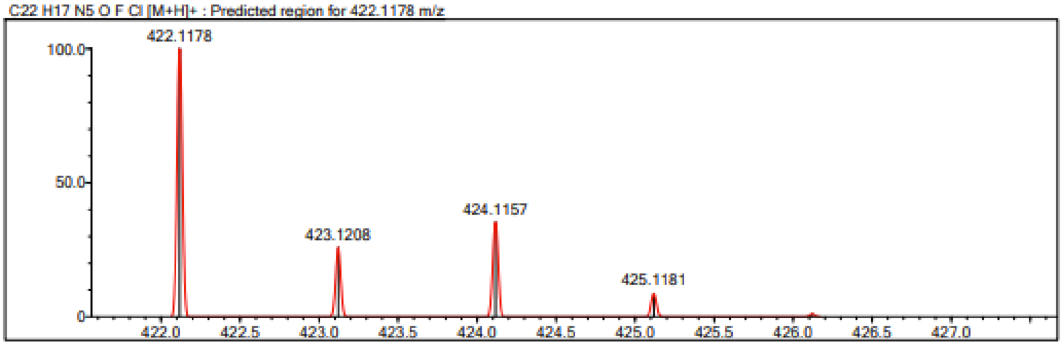

Rank	Score	Formula (M)	Ion	Meas. m/z	Pred. m/z	Df. (mDa)	Df. (ppm)	Iso	DBE
1	96.85	C22 H17 N5 O F Cl	[M+H]+	422.1175	422.1178	-0.3	-0.71	96.85	16.0

Figure S105. HRMS spectrum of compound 41

Scan positive no column.lcm

Formula Predictor Result	**C23 H20 N3 O2 F**
Mass	390.16178
Error Margin	20 ppm
DBE Range	0 - 1000
Electron Ions	Both configurations
HC Ratio	Not Used
Nitrogen Rule	Used

Score	Pred. (M)	Pred. m/z	Meas. m/z	Diff. (mDa)	Formulae (M)	Ion	Diff. (ppm)	Iso Score	DBE
96.51	389.15396	390.16123	390.16178	0.55	C23 H20 N3 O2 F	[M+H]+	1.410	96.88	15.0

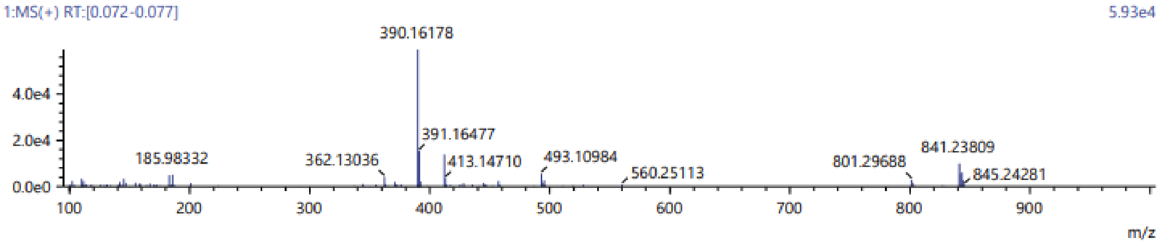

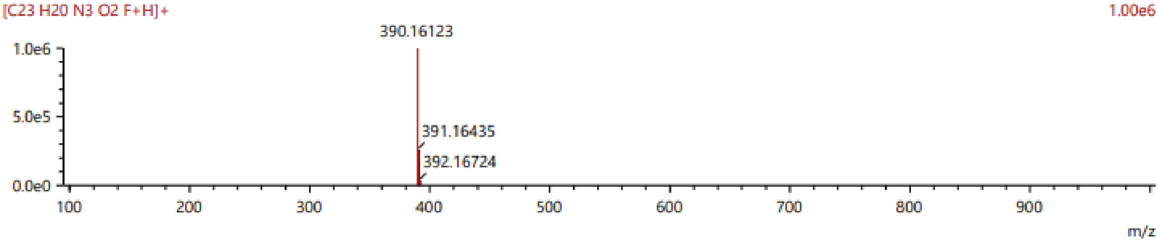

Figure S106. HRMS spectrum of compound 43

Data File: C:\LabSolutions\Data\Vilashini Rajaratnam\08052020 Analysis\MYM-III-43_08052020 HRMS Analysis_2_66.lcd

Elmt	Val.	Min	Max	Elmt	Val.	Min	Max	Elmt	Val.	Min	Max	Elmt	Val.	Min	Max	Use Adduct
H	1	8	25	N	3	0	5	Si	4	0	0	Br	1	0	0	H
2H	1	0	0	O	2	0	4	S	2	0	0	I	3	0	0	NH4
C	4	23	25	F	1	0	2	Cl	1	0	0					

Error Margin (ppm):	100	DBE Range:	-100.0 - 1000.0	Electron Ions:	both
HC Ratio:	0.0 - 10.0	Apply N Rule:	yes	Use MSn Info:	yes
Max Isotopes:	all	Isotope RI (%):	1.00	Isotope Res:	10000
MSn Iso RI (%):	75.00	MSn Logic Mode:	AND	Max Results:	10

Event#: 1 MS(E+) Ret. Time : 0.120 -> 0.133 - 1.360 -> 1.639 Scan# : 19 -> 21 - 205 -> 247

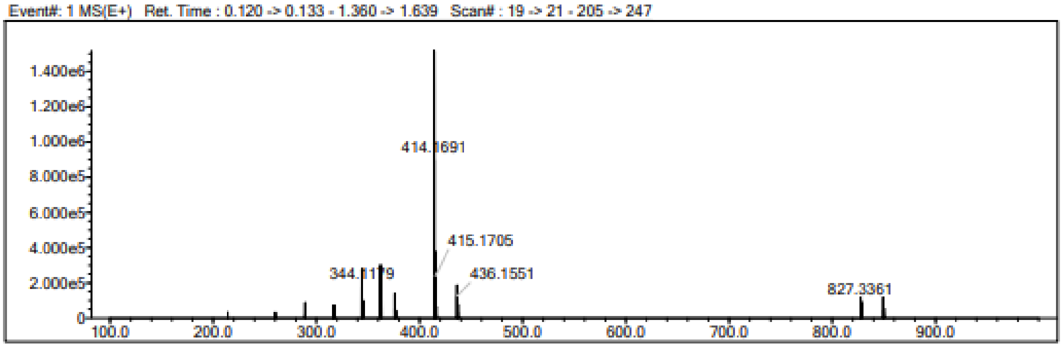

Measured region for 414.1691 m/z

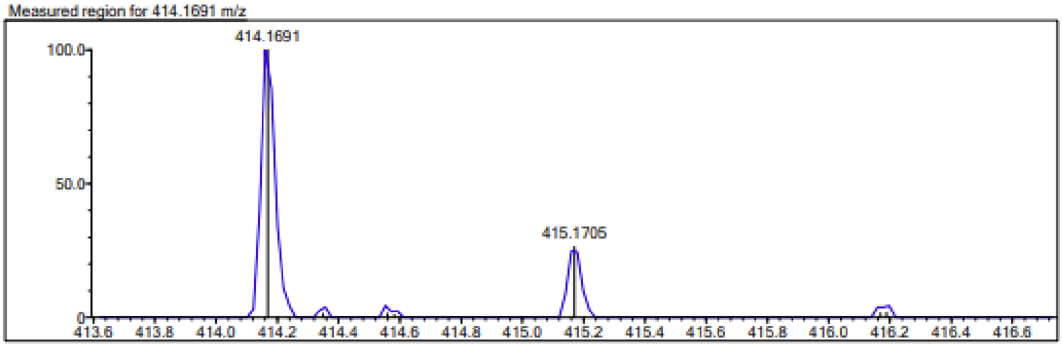

C24 H20 N5 O F [M+H]+ : Predicted region for 414.1725 m/z

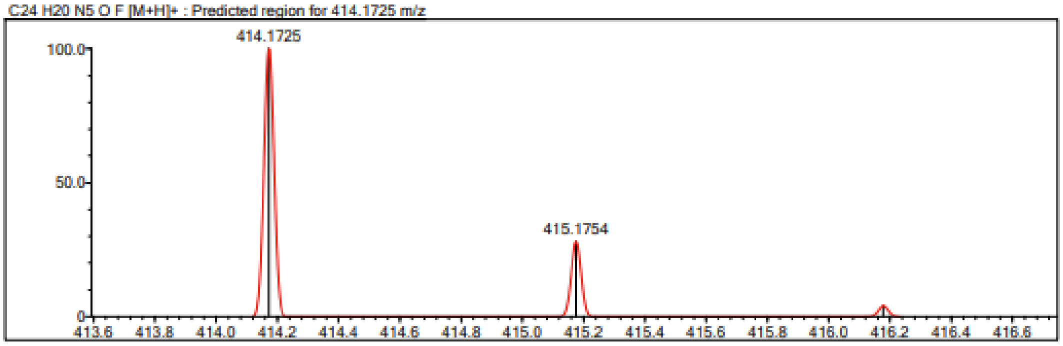

Rank	Score	Formula (M)	Ion	Meas. m/z	Pred. m/z	Df. (mDa)	Df. (ppm)	Iso	DBE
1	41.35	C24 H20 N5 O F	[M+H]+	414.1691	414.1725	-3.4	-8.21	71.41	17.0

Figure S107. HRMS spectrum of compound 44

Scan positive no column.lcm

Formula Predictor Result	**C22 H17 N5 O F Br**
Mass	466.06781
Error Margin	20 ppm
DBE Range	0 - 1000
Electron Ions	Both configurations
HC Ratio	Not Used
Nitrogen Rule	Used

Score	Pred. (M)	Pred. m/z	Meas. m/z	Diff. (mDa)	Formulae (M)	Ion	Diff. (ppm)	Iso Score	DBE
97.84	465.06005	466.06733	466.06781	0.48	C22 H17 N5 O F Br	[M+H]+	1.030	98.12	16.0

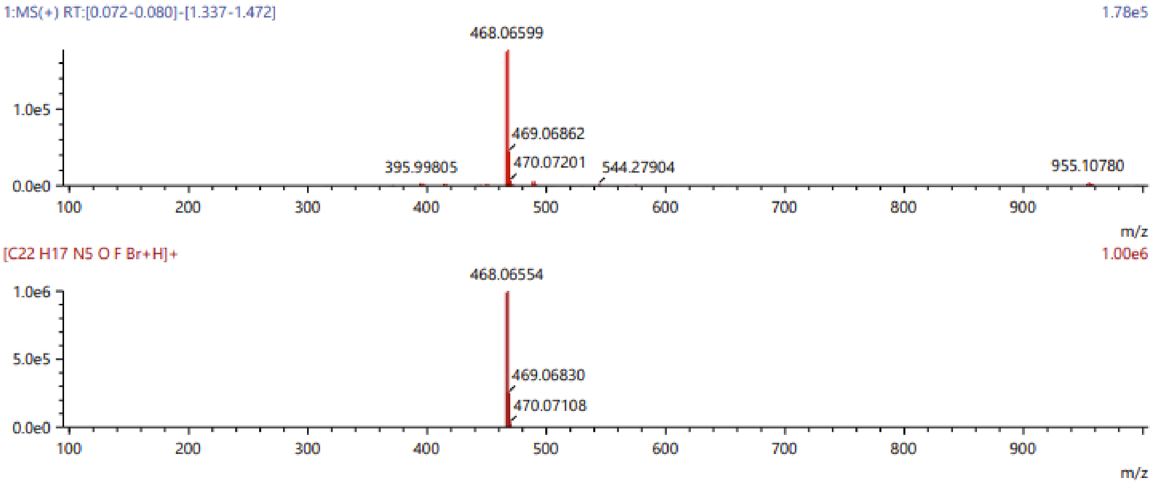

Figure S108. HRMS spectrum of compound 45

Scan positive no column.lcm

Formula Predictor Result	**C25 H22 N5 O F**
Mass	450.17001
Error Margin	20 ppm
DBE Range	0 - 1000
Electron Ions	Both configurations
HC Ratio	Not Used
Nitrogen Rule	Used

Score	Pred. (M)	Pred. m/z	Meas. m/z	Diff. (mDa)	Formulae (M)	Ion	Diff. (ppm)	Iso Score	DBE
99.62	427.18084	450.17006	450.17001	-0.05	C25 H22 N5 O F	[M+Na]+	-0.111	99.61	17.0

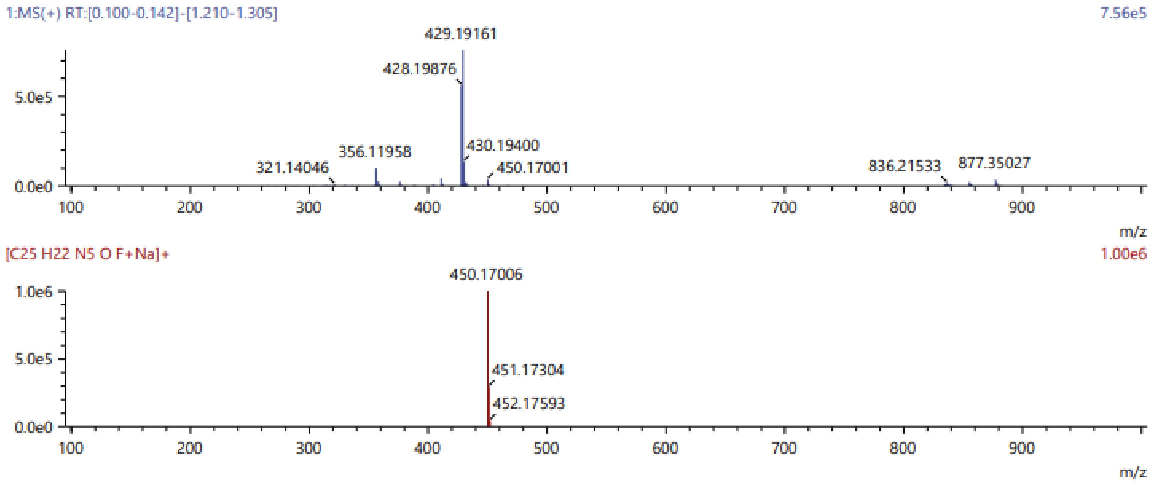

Figure S109. HRMS spectrum of compound 46

Table S1. PDSP raw data for compound **46**

56618	5-HT1A	41.67	12.85	17.44	-0.62	17.84
56618	5-HT1B	32.16	14.88	11.38	1.84	15.07
56618	5-HT1D	35.01	17.42	7.41	11.04	17.72
56618	5-HT1E	-29.59	-24.37	-15.55	-5.52	-18.76
56618	5-HT2A	35.97	23.12	22.29	13.11	23.62
56618	5-HT2B	31.86	29.27	20.26	18.9	25.07
56618	5-HT2C	12	9.57	-5.02	-2.45	3.53
56618	5-HT3	40.12	35.78	26.37	16.97	29.81
56618	5-HT4	11.92	3.63	-6.91	3.55	3.05
56618	5-HT5A	33.82	27.47	24.71	8.42	23.61
56618	5-HT6	83.88	69.33	68.31	64.47	71.5
56618	5-HT7A	14.64	2.74	-0.89	3.43	4.98

56618	A1	0	55.76	39.87	27.8	30.86
56618	A2A	11.92	25.58	28.91	42.57	27.25
56618	Alpha1A	41.63	20.29	32.01	33.26	31.8
56618	Alpha1B	4.54	3.83	12.41	16.45	9.31
56618	Alpha1D	39.75	36.97	26.33	36.48	34.88
56618	Alpha2A	36.33	40.9	19.51	20.16	29.23
56618	Alpha2B	48.66	43.55	49.15	35.47	44.21
56618	Alpha2C	15.81	10.01	15.97	5.68	11.87
56618	Beta1	20.92	1.96	-8.7	-119.77	-26.4
56618	Beta2	-2.62	-7.91	-10.62	-3.07	-6.06
56618	Beta3	57.95	16.53	25.32	24.64	31.11
56618	BZP Rat Brain Site	71.77	57.77	68.45	62.99	65.25
56618	D1	80.5	69.07	74.27	74.56	74.6
56618	D2	14.27	2.43	-0.72	2.93	4.73
56618	D3	37.5	28.76	26.33	18.69	27.82
56618	D4	16.07	7.58	9.13	9.79	10.64
56618	D5	45.19	53.75	52.46	53.23	51.16
56618	DAT	35.02	27.44	15.69	9.94	22.02
56618	DOR	79.82	59.28	62.35	67.91	67.34
56618	GABAA	17.75	14	2.76	43.57	19.52
56618	H1	43.99	34.04	30.03	26.85	33.73
56618	H2	12.6	18.16	-5.7	2.84	6.98
56618	H3	8.08	-3.75	1.32	-31.22	-6.39
56618	H4	39.21	-10.71	33.55	24.8	21.71
56618	KOR	96.52	89.95	88.57	90.25	91.32
56618	M1	6.32	-6.51	-2.24	5.86	0.86
56618	M2	27.96	-3.22	-4.96	3.13	5.73
56618	M3	32.45	7.28	7.28	-11.21	8.95
56618	M4	12.33	-10.89	7.34	-11.96	-0.8
56618	M5	7.45	5.14	5.69	4.49	5.69
56618	MOR	46.77	21.02	19.12	13.7	25.15

56618	NET	55.73	26.86	2.83	11.81	24.31
56618	PBR	93.92	89.21	76.55	85.84	86.38
56618	SERT	9.75	-7.56	-2.12	-13.19	-3.28
56618	Sigma 1	21.85	42.88	37.96	36.27	34.74
56618	Sigma 2	90.22	90.38	89.9	88.62	89.78
56618	AMPA	-1.98	-0.2	-4.66	10.08	0.81
56618	Kainate (Rat Brain)	0.23	44.74	48.05	59.82	38.21
56618	NMDA	58.88	55.54	68.38	81.35	66.04
56618	NR2B	23.96	37.16	42.43	27.96	32.88
56618	HCA2	-56.68	0	0	-12.17	-17.21
56618	Ca channel human	47.17	36.7	38.8	23.45	36.53
56618	HERG	10.37	2.96	4.52	-0.48	4.34
56618	M3D	20.44	4.33	22.86	-7.75	9.97
56618	M4 D	33.82	44.61	23.04	30.88	33.09
56618	NTS1	-33.07	-31.93	-28.3	-26.04	-29.84
56618	Alpha2Beta2	-10.24	-4.66	1.6	2.41	-2.72
56618	Alpha2Beta4	8.17	9.02	7.21	1.25	6.41
56618	Alpha3Beta2	7.24	11.92	-0.82	3.87	5.55
56618	Alpha3Beta4	7.69	9.95	15.9	8.14	10.42
56618	Alpha4Beta2	5.37	5.11	1.05	6.83	4.59
56618	Alpha4Beta2 (Rat Brain)	-9.32	-8.18	-4.36	-1.3	-5.79
56618	Alpha4Beta4	25.72	5.54	15.75	9.78	14.2
56618	Alpha7	-3.76	-1.05	10.98	12.11	4.57
56618	NOP	-0.58	-0.86	34.49	-3.05	7.5
56618	OT	17.13	3.73	9.76	15.12	11.44
56618	V1A	-0.84	3.42	-27.56	-1.96	-6.74
56618	V1B	5.34	-2.24	-14.97	22.55	2.67
56618	V2	50.54	36.03	55.13	51.82	48.38
56618	DAT	32.95	24.7	30.73	29.15	29.38

Table S2. PDSP raw data for compound **36**

56635	5-HT1A	9.9	2.18	-3.7	3.63	3
56635	5-HT1B	6.29	6.9	3.65	-2.18	3.67
56635	5-HT1D	2.57	2.23	-5.67	-3.75	-1.16
56635	5-HT1E	4.51	-16.27	10.65	22.23	5.28
56635	5-HT2A	17.49	13.36	3.25	10.22	11.08
56635	5-HT2B	16.86	5.06	-4.47	-3.56	3.47
56635	5-HT2C	32.31	8.38	13.78	1.32	13.95
56635	5-HT3	5.75	-10.13	-11.67	8.06	-2
56635	5-HT4	-16.5	5.23	-25.53	-12.36	-12.29
56635	5-HT5A	5.01	-7.92	-4.56	0.1	-1.84
56635	5-HT6	24.99	8.62	3.71	5.82	10.79
56635	5-HT7A	20.71	-9.1	-7.69	2.2	1.53
56635	A1	85.03	42.47	43.19	100.9	67.9
56635	A2A	2.99	-0.9	2.14	1.47	1.43
56635	Alpha1A	20.92	-10.32	-17.56	5.61	-0.34
56635	Alpha1B	4.7	-3.11	-3.11	-1.65	-0.79
56635	Alpha1D	-14.18	-25.01	0	-17.91	-14.28
56635	Alpha2A	-1.41	-16.94	-15.47	-23.65	-14.37
56635	Alpha2B	19.62	4.41	14.45	28.08	16.64
56635	Alpha2C	5	4.85	-8.01	3.87	1.43
56635	Beta1	-10.22	-19.98	1.73	-30.36	-14.71
56635	Beta2	-2.64	6.81	-4.45	10.97	2.67
56635	Beta3	27.35	-16.92	8.35	-11.76	1.76
56635	BZP Rat Brain Site	87.05	79.43	81.34	79.43	81.81
56635	D1	22.88	62.43	33.16	52.58	42.76
56635	D2	21.61	0.64	-5.1	-4.48	3.17
56635	D3	8.4	4.68	-9.54	16.33	4.97
56635	D4	11.94	-12.65	-13.68	-3.59	-4.5
56635	D5	6.28	-7.84	5.53	8.65	3.16
56635	DAT	26.68	0.07	30.23	-12.88	11.03
56635	DOR	58.12	32.3	36.37	36.16	40.74
56635	GABAA	3.49	7.34	9.27	32.39	13.12
56635	H1	17.27	1.23	2.71	9.59	7.7
56635	H2	5.37	4.17	12.15	0.74	5.61
56635	H3	10.58	2.61	-8.4	-8.02	-0.81
56635	H4	54.73	24.06	10.27	26.53	28.9
56635	KOR	100.77	97.38	94.76	92.76	96.42
56635	M1	6.3	-3.36	-13.2	9.19	-0.27
56635	M2	6.24	-8.59	-2.03	-10.11	-3.62
56635	M3	-2.49	-5.63	-2.66	-5.13	-3.98

56635	M4	7.16	-10.81	-8.67	-9.32	-5.41
56635	M5	-5.65	-16.63	-5.08	-15.25	-10.65
56635	MOR	24.21	16.03	24.72	11.82	19.2
56635	NET	7.83	-7.83	-3.74	-5.71	-2.36
56635	PBR	71.87	43.14	54.27	59.48	57.19
56635	SERT	20.32	11.37	-7.65	-11.7	3.09
56635	Sigma 1	36.17	46.3	49.27	52.24	46
56635	Sigma 2	82.1	82.1	80.31	81.11	81.41
56635	AMPA	-13.54	-29.63	-55.95	2.56	-24.14
56635	Kainate (Rat Brain)	42.53	28.57	42.09	58.23	42.86
56635	NMDA	70.17	53.9	47.1	51.23	55.6
56635	NR2B	27.05	11.95	1.69	17	14.42
56635	HCA2	-1.3	-78.41	-37.42	0	-29.28
56635	Ca channel human	41.73	47.6	-60.22	37.83	16.74
56635	HERG	22.33	13.02	-13.72	-26.24	-1.15
56635	M3D	-26.27	1.24	8.12	8.88	-2.01
56635	M4 D	34.63	20.53	21.54	29.6	26.58
56635	NTS1	-5.95	-6.62	-27.05	-27.95	-16.89
56635	Alpha2Beta2	-8.2	-9.56	-5.88	-3.16	-6.7
56635	Alpha2Beta4	5.51	25.53	6.47	5.94	10.86
56635	Alpha3Beta2	1.63	6.01	6.22	10.19	6.01
56635	Alpha3Beta4	17.53	17.26	14.28	13.38	15.61
56635	Alpha4Beta2	-4.54	0.03	0.16	-1.18	-1.38
56635	Alpha4Beta2 (Rat Brain)	-0.67	10.4	3.79	-0.54	3.25
56635	Alpha4Beta4	3.3	15.01	12.27	10.27	10.21
56635	Alpha7	0.75	-17.75	-20.15	-18.27	-13.86
56635	OT	24.5	14.11	12.44	7.75	14.7
56635	V1A	49	28.8	10.83	21.61	27.56
56635	V1B	16.11	20.58	22.42	32.41	22.88
56635	V2	46.87	48.52	-64.95	19.88	12.58

Table S3. PDSP raw data for compound **44**

56637	5-HT1A	47.32	38.83	30.83	27.45	36.11
56637	5-HT1B	74.11	58.65	56.14	59.45	62.09
56637	5-HT1D	79.81	69.2	64.07	56.68	67.44
56637	5-HT1E	14.05	1.45	14.39	17.12	11.75
56637	5-HT2A	26.16	17.15	15.66	25.73	21.18
56637	5-HT2B	21.97	20.95	14.25	16.75	18.48
56637	5-HT2C	36.53	26.33	22.9	13.09	24.71
56637	5-HT3	22.97	22.87	-0.99	6.42	12.82
56637	5-HT4	-9.91	-9.07	-10.01	-8.03	-9.26

56637	5-HT5A	23.38	-7.66	5.27	-0.68	5.08
56637	5-HT6	55.63	21.02	20.32	13.3	27.57
56637	5-HT7A	31.37	18.98	-14.59	1.1	9.22
56637	A1	106.67	35.26	38.86	12.17	48.24
56637	A2A	30.56	12.59	23.62	18.68	21.36
56637	Alpha1A	15.33	19.47	23.82	27.13	21.44
56637	Alpha1B	12.2	0.12	3.35	11.79	6.87
56637	Alpha1D	15.9	-2.22	0.02	-8.57	1.28
56637	Alpha2A	4.8	16.74	13.3	22.79	14.41
56637	Alpha2B	43.87	37.7	29.81	34.97	36.59
56637	Alpha2C	3.26	22.17	24.14	13.32	15.72
56637	Beta1	4.25	-10.85	-7.71	-6.45	-5.19
56637	Beta2	11.65	-2.08	7.15	-0.84	3.97
56637	Beta3	17.02	-5.12	-22.83	-5.12	-4.01
56637	BZP Rat Brain Site	99.43	98.48	95.15	93.01	96.52
56637	D1	42.29	46.06	58.52	51.42	49.57
56637	D2	2.51	2.01	-1.23	-0.61	0.67
56637	D3	19.82	16.68	8.87	18.89	16.07
56637	D4	12.8	-7.04	-6.44	-15.58	-4.07
56637	D5	16.4	4.65	12.53	2.66	9.06
56637	DAT	55.99	34.93	33.65	24.83	37.35
56637	DOR	87.4	75.4	84.35	78.25	81.35
56637	GABAA	11.2	11.2	20.35	4.94	11.92
56637	H1	36.69	12.29	8.78	7.43	16.3
56637	H2	16.18	0.99	-12.76	1.8	1.55
56637	H3	12.1	7.93	9.07	-0.05	7.26
56637	H4	35.38	25.5	3.89	17.47	20.56
56637	KOR	100	95.22	95.99	93.84	96.26
56637	M1	13.25	1.96	-8.15	30.04	9.28
56637	M2	-0.13	-22.08	-17.23	-15.05	-13.62
56637	M3	-2.66	2.29	-1.5	-0.52	-0.6
56637	M4	4.55	-6.34	-17.33	-0.57	-4.92
56637	M5	-1.53	-15.6	-24.06	-15.14	-14.08
56637	MOR	35.96	26.5	23.69	19.61	26.44
56637	NET	7.83	0.78	9.1	1.9	4.9
56637	PBR	75.11	87.93	71.03	50.04	71.03
56637	SERT	19.14	-5.37	8.41	6.63	7.2
56637	Sigma 1	36.35	56.08	71.27	49.79	53.37
56637	Sigma 2	76.08	68.62	76.43	78.77	74.98
56637	AMPA	20.54	-6.15	-21.49	-30.2	-9.33
56637	Kainate (Rat Brain)	26.83	47.33	38.17	57.36	42.42

56637	NMDA	51.47	19.67	0.73	8.25	20.03
56637	NR2B	23.78	32.48	27.26	3.43	21.74
56637	HCA2	12.59	0	-60.35	0	-11.94
56637	Ca channel human	0	57.04	42.39	47.27	36.68
56637	HERG	54.53	29.71	26.93	21.8	33.24
56637	M3D	19.58	0.48	7.35	19.58	11.75
56637	M4 D	34.63	38.66	33.63	36.65	35.89
56637	NTS1	-62.07	-69.47	-62.96	-54.21	-62.18
56637	Alpha2Beta2	-13.64	-15.14	-1.39	3.5	-6.67
56637	Alpha2Beta4	6.68	10.94	4.45	10.83	8.23
56637	Alpha3Beta2	-8.87	0.92	-3.67	11.52	-0.02
56637	Alpha3Beta4	3.09	10.31	8.05	8.78	7.56
56637	Alpha4Beta2	-0.41	0.16	-2.13	-5.43	-1.95
56637	Alpha4Beta2 (Rat Brain)	4.68	-4.36	-3.85	-0.41	-0.99
56637	Alpha4Beta4	12.52	13.26	11.77	4.79	10.59
56637	Alpha7	-22.03	-17.22	-26.1	-31.51	-24.22
56637	OT	19.47	15.79	0.71	25.5	15.37
56637	V1A	53.69	-3.65	16.42	18.61	21.27
56637	V1B	38.19	55.15	6.77	42.79	35.73
56637	V2	-5.1	44.66	38.05	33.28	27.72

Table S4. PDSP raw data for compound **35**

56638	5-HT1A	43.94	29.28	20.99	21.37	28.9	
56638	5-HT1B	17.81	12.79	10.95	4.75	11.58	
56638	5-HT1D	17.35	14.25	5.44	-10.41	6.66	
56638	5-HT1E	-2.3	2.47	9.96	13.37	5.88	
56638	5-HT2A	35.38	33.42	30.7	29.77	32.32	
56638	5-HT2B	17.2	15.27	13.46	9.26	13.8	
56638	5-HT2C	31.43	16.43	21.82	9.17	19.71	
56638	5-HT3	4.31	-18.02	-18.21	-5.41	-9.33	
56638	5-HT4	-2.2	-1.07	-6.34	7.68	-0.48	
56638	5-HT5A	33.99	17.69	6.31	20.02	19.5	
56638	5-HT6	34.12	4.88	10.73	-8.45	10.32	
56638	5-HT7A	28.55	10.2	-0.16	-6.12	8.12	
56638	A1	70.6	-63.57	42.47	59.06	27.14	
56638	A2A	16.4	20.1	15.64	27.23	19.84	
56638	Alpha1A	32.71	15.75	14.51	20.3	20.82	
56638	Alpha1B	11.58	10.43	15.22	-2.49	8.69	
56638	Alpha1D	17.21	0.77	-13.43	-6.14	-0.4	
56638	Alpha2A	41.75	31.29	40.28	24.59	34.48	
56638	Alpha2B	47.74	42.72	42.58	49.89	45.73	

56638	Alpha2C	43.58	35.1	23.15	24.59	31.61	
56638	Beta1	14.63	-9.59	-1.1	0	0.99	
56638	Beta2	0.17	8.5	-5.46	-1.86	0.34	
56638	Beta3	40.44	36.38	25.69	9.27	27.95	
56638	BZP Rat Brain Site	31.34	18.24	18.72	11.34	19.91	
56638	D1	25.92	36.79	27.37	26.79	29.22	
56638	D2	18.86	4.01	5.13	1.89	7.47	
56638	D3	20.64	11.78	16.91	15.63	16.24	
56638	D4	13.4	4.69	0.64	5.47	6.05	
56638	D5	38.77	19.9	25.9	9.65	23.56	
56638	DAT	70.5	41.62	46.74	38.91	49.44	
56638	DOR	58.53	55.07	50.19	47.14	52.73	Secondary
56638	GABAA	21.79	6.38	19.39	16.5	16.02	
56638	H1	19.3	12.15	10.53	0.15	10.53	
56638	H2	-3.14	-14.73	-11.45	-10.88	-10.05	
56638	H3	20.46	22.73	-0.43	-23.21	4.89	
56638	H4	80.67	55.35	45.27	37.44	54.68	Secondary
56638	KOR	35.89	40.51	36.97	38.51	37.97	
56638	M1	10.09	-9.86	-11.58	4.85	-1.63	
56638	M2	3.29	-18	-4.22	-12.86	-7.95	
56638	M3	2.62	3.61	17.79	14.98	9.75	
56638	M4	16.19	-14.72	-5.32	-12.77	-4.16	
56638	M5	-1.07	-5.76	-11.82	-10.91	-7.39	
56638	MOR	6.2	23.44	12.45	17.18	14.82	
56638	NET	22.5	5.85	3.31	9.24	10.23	
56638	PBR	70.18	60.74	88.91	67.22	71.76	Secondary
56638	SERT	32.15	25.99	10.86	-0.38	17.16	
56638	Sigma 1	44.55	41.06	48.4	47	45.25	
56638	Sigma 2	87.28	83.34	84.69	86.13	85.36	Secondary
56638	AMPA	-25.09	6.72	-20.92	16.57	-5.68	
56638	Kainate (Rat Brain)	35.11	49.95	48.2	0	33.32	
56638	NMDA	62.4	26.71	28.16	33.75	37.76	
56638	NR2B	30.27	31.35	18.17	19.44	24.81	
56638	HCA2	-46.45	0	-29.09	0.78	-18.69	
56638	Ca channel human	55.09	68.77	65.51	58.02	61.85	
56638	HERG	46.72	33.78	30.89	37.95	37.34	
56638	M3D	2.01	15.76	-10.98	11.17	4.49	
56638	M4 D	2.39	18.51	12.47	29.6	15.74	
56638	NTS1	-31.09	-37.37	-32.21	-29.74	-32.6	
56638	Alpha2Beta2	2.55	-1.67	7.45	8.4	4.18	
56638	Alpha2Beta4	8.06	21.16	3.17	10.3	10.67	

56638	Alpha3Beta2	10.8	3.67	0.61	6.62	5.43	
56638	Alpha3Beta4	11.93	13.11	14.37	8.59	12	
56638	Alpha4Beta2	2.89	3.4	-3.27	-0.22	0.7	
56638	Alpha4Beta2 (Rat Brain)	-3.85	-14.41	-12.12	-15.94	-11.58	
56638	Alpha4Beta4	21.23	12.76	26.46	11.27	17.93	
56638	Alpha7	-15.49	-24.82	-29.55	-22.86	-23.18	
56638	OT	19.67	-1.29	-5.76	12.46	6.27	
56638	V1A	31.09	-3.31	-35.35	23.17	3.9	
56638	V1B	15.18	22.55	12.95	33.2	20.97	
56638	V2	51.53	26.61	47.1	24.44	37.42	

Table S5. PDSP raw data for compound **33**

56642	5-HT1A	8.74	2.47	-3.03	1.31	2.37	
56642	5-HT1B	13.83	12.05	9.84	11.31	11.76	
56642	5-HT1D	-1.66	3.3	-7.87	-7.42	-3.41	
56642	5-HT1E	16.1	32.11	35.86	52.21	34.07	
56642	5-HT2A	21.44	2.27	12.05	6.86	10.66	
56642	5-HT2B	35.81	22.31	29.23	18.79	26.54	
56642	5-HT2C	38.78	15.25	14.27	3.87	18.04	
56642	5-HT3	12.29	-0.31	-4.16	-11.38	-0.89	
56642	5-HT4	1.38	-23.93	-20.26	-14.99	-14.45	
56642	5-HT5A	46.15	35.54	93.24	39.42	53.59	Secondary
56642	5-HT6	41.13	19.62	23.36	14.24	24.59	
56642	5-HT7A	7.06	2.35	2.98	4.86	4.31	
56642	A1	56.18	22.99	-41.21	65.55	25.88	
56642	A2A	11.36	13.83	-17.54	38.64	11.57	
56642	Alpha1A	-6.8	-6.59	-14.25	9.75	-4.47	
56642	Alpha1B	1.47	-15.82	-16.86	-30.2	-15.35	
56642	Alpha1D	26.37	-6.33	-20.53	14.78	3.57	
56642	Alpha2A	7.42	9.22	-3.37	10.69	5.99	
56642	Alpha2B	80.74	63.52	65.82	65.67	68.94	Secondary
56642	Alpha2C	19.52	-3.24	14	21.19	12.87	
56642	Beta1	-9.91	2.36	-33.82	-21.86	-15.81	
56642	Beta2	-13.56	12.44	-5.91	3.55	-0.87	
56642	Beta3	9.64	-28.36	-37.77	-20.98	-19.37	
56642	BZP Rat Brain Site	95.86	95.86	93.96	93.48	94.79	Secondary

56642	D1	-51.89	-43.63	-30.59	-15.52	-35.41	
56642	D2	4.88	-4.35	1.39	-0.86	0.27	
56642	D3	-5.11	3.04	-5.69	-6.51	-3.57	
56642	D4	4.78	-6.61	-6.61	-9.37	-4.45	
56642	D5	-12.59	-6.47	11.53	12.4	1.22	
56642	DAT	37.78	26.82	18.85	35.36	29.7	
56642	DOR	54.46	34.33	37.38	45.72	42.97	
56642	GABAA	8.87	-17.08	-6.01	-1.81	-4.01	
56642	H1	0.15	-13.33	-16.3	-12.12	-10.4	
56642	H2	-9.72	-8.44	3.92	8.55	-1.42	
56642	H3	20.84	6.03	-8.78	5.65	5.94	
56642	H4	16.03	-11.76	-5.38	4.71	0.9	
56642	KOR	61.01	59.47	50.84	42.98	53.58	Secondary
56642	M1	0.34	7.2	-11.04	-2.91	-1.6	
56642	M2	28.38	10.04	13.46	9.85	15.43	
56642	M3	18.61	-2.82	-2.49	-1.17	3.03	
56642	M4	0.92	-10.25	-10.91	7.07	-3.29	
56642	M5	3.96	-3.82	-1.64	-1.53	-0.76	
56642	MOR	-10.03	-4.15	-20.12	-10.41	-11.18	
56642	NET	9.94	-10.65	-13.19	-10.09	-6	
56642	PBR	19.05	21.17	27.79	21.03	22.26	
56642	SERT	-2.15	-17.87	-7.73	-9.34	-9.27	
56642	Sigma 1	44.73	39.66	56.43	46.82	46.91	
56642	Sigma 2	47.92	46.97	36.43	43.19	43.63	
56642	AMPA	-11.64	19.98	-0.66	23.38	7.77	
56642	Kainate (Rat Brain)	16.38	7.84	0	-10.51	3.43	
56642	NMDA	93.9	37.55	22.02	19.21	43.17	
56642	NR2B	7.74	9.21	1.69	-3.48	3.79	
56642	HCA2	24.4	-45.07	-22.84	4.25	-9.82	
56642	Ca channel human	53.46	30.01	38.48	34.24	39.05	
56642	HERG	5.22	2.65	-4.41	-5.38	-0.48	
56642	M3D	5.04	0.2	16.33	-2.22	4.84	
56642	M4 D	71.93	47.35	-54.33	43.99	27.24	
56642	NTS1	-23.43	-24.56	-3.48	-15.27	-16.69	
56642	Alpha2Beta2	-11.87	-5.07	-0.31	-6.56	-5.95	
56642	Alpha2Beta4	-6.1	10.41	4.55	10.41	4.82	
56642	Alpha3Beta2	6.01	-3.46	1.63	4.38	2.14	
56642	Alpha3Beta4	-6.11	1.92	-1.24	2.37	-0.77	
56642	Alpha4Beta2	-1.43	-3.72	-2.7	1.24	-1.65	
56642	Alpha4Beta2 (Rat Brain)	-13.27	-5.12	-3.85	-0.54	-5.7	
56642	Alpha4Beta4	0.31	4.05	9.03	-5.42	1.99	

56642	Alpha7	-24.82	-24.97	-13.31	-10.23	-18.33	
56642	OT	-1.64	-4.01	2.02	8.48	1.21	
56642	V1A	49.64	0.75	9.93	1.89	15.55	
56642	V1B	11.53	-15.35	5.18	4.25	1.4	
56642	V2	18.31	16.07	45.23	-0.76	19.71	
56642	OT	16.36	8.6	-1.33	9.09	8.18	

Table S6. PDSP raw data for compound **37**

58241	5-HT1A	0.59	-11.77	-7.22	-5.59	-6	
58241	5-HT1B	38.51	6.44	1.61	-5.05	10.38	
58241	5-HT1D	14.87	13.66	3.58	6.8	9.73	
58241	5-HT1E	-8.95	-8.38	-28.56	-13.21	-14.78	
58241	5-HT2A	30.94	-5	15.25	15.25	14.11	
58241	5-HT2B	22.71	5.83	11.01	-5.3	8.56	
58241	5-HT2C	13.76	22.18	18.86	15.29	17.52	
58241	5-HT3	16.08	15.35	12.75	9.67	13.46	
58241	5-HT4	59.46	53.25	55.58	23.76	48.01	
58241	5-HT5A	5.97	-1.77	-1.5	-13.52	-2.71	
58241	5-HT6	-2.14	0.23	-6.58	1.89	-1.65	
58241	5-HT7A	8.96	14.54	7.29	17.56	12.09	
58241	A1	54.35	49.56	51.96	53.05	52.23	Secondary
58241	A2A	11.51	-4.47	-20.52	5.99	-1.87	
58241	Alpha1A	-7.22	-8.11	-6.15	-12.03	-8.38	
58241	Alpha1B	19.29	-4.07	3.12	-1.27	4.27	
58241	Alpha1D	6.36	1	1.45	7.56	4.09	
58241	Alpha2A	55.48	59.26	9.83	-33.25	22.83	
58241	Alpha2B	28.43	15.45	21.06	9.16	18.53	
58241	Alpha2C	15.54	-4.47	3.37	-3.49	2.74	
58241	Beta1	19.91	30.9	23.34	-41.59	8.14	
58241	Beta2	9.36	12.57	-0.73	5.01	6.55	
58241	Beta3	53.5	29.27	42.47	58.91	46.04	
58241	AT2	26.17	12.95	2.17	6.84	12.03	
58241	BZP Rat Brain Site	77.51	76.86	72.85	69.06	74.07	Secondary
58241	D1	19.85	26.11	29.13	34.53	27.41	
58241	D2	5.94	3.76	9.3	53.79	18.2	
58241	D3	8.81	-7.31	-0.07	-4.23	-0.7	
58241	D4	-4.9	-5.51	-19.83	-9.95	-10.05	
58241	D5	10.17	14.2	19.89	23.92	17.05	
58241	DAT	76.9	53.6	23.82	-4.38	37.49	
58241	DOR	48.16	29.33	26.82	23.48	31.95	
58241	GABAA	54.54	25.88	31.61	14.41	31.61	

58241	H1	92.55	76.08	51.61	15.93	59.04	Secondary
58241	H2	15.56	0.82	4.67	-14.41	1.66	
58241	H3	12.16	-0.69	10.02	4.2	6.42	
58241	H4	19.23	3.36	-1.05	4.02	6.39	
58241	KOR	100.71	99.95	99.95	96.21	99.21	Secondary
58241	M1	-2.88	-1.88	3.13	-0.28	-0.48	
58241	M2	-10.33	-14.69	-23.15	-29.15	-19.33	
58241	M3	35.39	15.47	7.3	4.38	15.64	
58241	M4	7.31	-3.52	-6.18	-13.66	-4.01	
58241	M5	8.78	-9.29	-10.41	-8.58	-4.88	
58241	MOR	53.65	49.76	42.06	48.92	48.6	
58241	NET	34.46	12.69	3.83	-14.83	9.04	
58241	PBR	96.89	91.74	84.85	84.78	89.57	Secondary
58241	SERT	21.05	8.5	14.75	15.6	14.98	
58241	Sigma 1	3.11	7.05	-2.43	14.94	5.67	
58241	Sigma 2	82.32	75.18	68.66	78.51	76.17	Secondary
58241	AMPA	19.83	12.06	8.06	42.42	20.59	
58241	Kainate (Rat Brain)	38.76	47.75	55.03	0	35.39	
58241	NMDA	56.77	46.76	53.19	59.81	54.13	Secondary
58241	NR2B	49.28	34.29	42.3	11.98	34.46	
58241	HCA2	23.66	33.9	15.91	67.38	35.21	
58241	Ca channel human	29.4	-23.6	19.46	2.9	7.04	
58241	HERG	37.75	8.85	14.92	21.41	20.73	
58241	M3D	-14.82	-34.14	-19.21	-25.36	-23.38	
58241	M4 D	-9.72	-44.71	-47.63	23.33	-19.68	
58241	NTS1	-17.98	-28.9	-10.31	-7.99	-16.3	
58241	Alpha2Beta2	8.38	-1.08	5.01	0.84	3.29	
58241	Alpha2Beta4	4.76	-3.62	-22.34	-0.72	-5.48	
58241	Alpha3Beta2	9.39	-0.06	7.32	6.41	5.77	
58241	Alpha3Beta4	21.89	19.86	11.1	22.51	18.84	
58241	Alpha4Beta2	0.3	6.98	7.08	10.27	6.16	
58241	Alpha4Beta2 (Rat Brain)	-4.37	-3.77	4.13	1.58	-0.61	
58241	Alpha4Beta4	6.99	15.8	15.04	13.89	12.93	
58241	Alpha7	21.87	15.46	25.74	21.96	21.26	
58241	NOP	-9.85	-12.17	-12.48	-5.99	-10.12	
58241	OT	30.7	9.11	-11.94	14.24	10.53	
58241	V1A	-30.22	28.49	-20.75	-17.74	-10.06	
58241	V1B	-17.02	-26.43	-22.53	4.33	-15.41	
58241	V2	6.87	-45.17	-1.32	10.96	-7.17	
58241	PBR	88.17	86.9	90.14	86.9	88.03	
58241	SERT	-6.56	0.1	-4.87	7.89	-0.86	

58241	GABAA	5.95	-9.72	8.85	11.76	4.21	
58241	Oxytocin	6.9	9.38	25.3	3.67	11.31	

Table S7. PDSP raw data for compound **38**

58242	5-HT1A	-2.83	4.66	-7.06	-1.04	-1.57	
58242	5-HT1B	40.49	4.45	-8.74	-1.22	8.75	
58242	5-HT1D	20.51	8.01	-2.47	3.18	7.31	
58242	5-HT1E	-42.2	-26.86	-17.19	-3.55	-22.45	
58242	5-HT2A	32.62	1.08	5.8	6.81	11.58	
58242	5-HT2B	28.08	13.69	-6.07	4.29	10	
58242	5-HT2C	-16.37	24.48	5.59	15.03	7.18	
58242	5-HT3	18.11	11.86	13.16	10.89	13.51	
58242	5-HT4	47.82	31.52	46.27	29.19	38.7	
58242	5-HT5A	13.99	-7.64	-10.31	-7.38	-2.84	
58242	5-HT6	-6.3	-10.33	-12.97	4.12	-6.37	
58242	5-HT7A	15.32	2.26	10.75	19.23	11.89	
58242	A1	59.14	42.6	66.75	53.48	55.49	Secondary
58242	A2A	21.96	19.58	32.01	6.24	19.95	
58242	Alpha1A	3.48	15.95	-9.71	-3.12	1.65	
58242	Alpha1B	6.57	47.82	-6.37	-5	10.76	
58242	Alpha1D	-16.13	-7.79	10.39	2.19	-2.84	
58242	Alpha2A	0	-0.12	26.82	35.75	15.61	
58242	Alpha2B	25.37	15.06	20.16	9.73	17.58	
58242	Alpha2C	15.94	15.08	3.6	5.1	9.93	
58242	Beta1	9.43	22.14	2.73	5.48	9.95	
58242	Beta2	-4.12	2.92	7.44	0.75	1.75	
58242	Beta3	35.11	23.42	-14.01	52.42	24.24	
58242	AT2	20.9	35.21	10.54	29.91	24.14	
58242	BZP Rat Brain Site	91.8	88.01	85.84	87.47	88.28	Secondary
58242	D1	30.86	45.75	53.3	41.87	42.95	
58242	D2	4.92	8.42	22.57	51.31	21.81	
58242	D3	0.48	-2.42	-3.15	4.82	-0.07	
58242	D4	-4.1	-22.86	-2.08	15.27	-3.44	
58242	D5	14.43	4	15.38	22.5	14.08	
58242	DAT	75.5	33.98	37.84	12.61	39.98	
58242	DOR	56.31	32.89	36.86	44.81	42.72	
58242	GABAA	51.35	36.7	20.78	15.68	31.13	
58242	H1	93.02	74.48	50.19	16.03	58.43	Secondary
58242	H2	12.14	0.75	15.34	-0.99	6.81	
58242	H3	10.93	-11.39	3.29	10.02	3.21	
58242	H4	15.27	-8.32	13.5	0.5	5.24	

58242	KOR	100.63	99.52	99.01	96.29	98.86	Secondary
58242	M1	-9.1	4.34	6.14	-2.28	-0.23	
58242	M2	6.31	-2.42	-22.33	-20.15	-9.65	
58242	M3	32.04	12.62	21.53	10.95	19.29	
58242	M4	-6.37	-5.39	-6.47	-16.41	-8.66	
58242	M5	3.91	-11.63	-0.66	-12.34	-5.18	
58242	MOR	36.22	36.69	26.4	21.58	30.22	
58242	NET	23.95	-0.83	-6.58	-17.42	-0.22	
58242	PBR	94.09	93.33	88.79	90.53	91.69	Secondary
58242	SERT	26.28	8.89	3.6	13.5	13.07	
58242	Sigma 1	5.21	-9.33	-16.73	14.82	-1.51	
58242	Sigma 2	85.85	74.69	80.03	82.87	80.86	Secondary
58242	AMPA	18.42	15.83	17.71	33.72	21.42	
58242	Kainate (Rat Brain)	47.75	51.61	0	46.47	36.46	
58242	NMDA	48.19	61.95	72.86	77.86	65.22	Secondary
58242	NR2B	38.67	43.11	31.69	12.5	31.49	
58242	HCA2	-9.57	-8.86	11.66	2.36	-1.1	
58242	Ca channel human	31.06	40.99	-3.73	54.24	30.64	
58242	HERG	45.08	-8.53	-20.26	21.2	9.37	
58242	M3D	28.21	-4.28	0.99	2.74	6.92	
58242	M4 D	17.5	-35.97	-62.21	5.83	-18.71	
58242	NTS1	-26.34	-11.7	-10.08	-4.73	-13.21	
58242	Alpha2Beta2	3.73	0.36	-3.33	5.01	1.44	
58242	Alpha2Beta4	1.26	2.32	2.02	-3.46	0.54	
58242	Alpha3Beta2	-0.19	8.48	8.61	10.94	6.96	
58242	Alpha3Beta4	9.06	9.67	18.64	12.32	12.42	
58242	Alpha4Beta2	-5.09	8.58	1.4	1.8	1.67	
58242	Alpha4Beta2 (Rat Brain)	-2.07	-4.62	5.22	3.4	0.48	
58242	Alpha4Beta4	7.38	4.69	16.19	6.23	8.62	
58242	Alpha7	-12.65	11.33	22.66	24.86	11.55	
58242	NOP	-26.84	-15.26	-17.11	-8.77	-17	
58242	OT	23.68	13.43	1.01	-1.69	9.11	
58242	V1A	12.8	10	-6.13	0.54	4.3	
58242	V1B	-0.95	-15.64	8.24	-20.46	-7.2	
58242	V2	8.79	-3.25	-4.22	20.6	5.48	
58242	PBR	92.19	85.48	89.51	91.01	89.55	
58242	SERT	22.41	8.47	-7.27	5.11	7.18	
58242	GABAA	13.5	33.24	2.47	7.11	14.08	
58242	Oxytocin	5.11	-0.87	-5.36	8.6	1.87	

Table S8. PDSP raw data for compound **31**

56631	5-HT1A	-3.43	3.31	4.25	-6.05	-0.48
56631	5-HT1B	-0.03	-3.03	-1.34	3.34	-0.27
56631	5-HT1D	-14.08	-13.26	-20.9	-11.66	-14.98
56631	5-HT1E	20.53	18.82	17.46	15.76	18.14
56631	5-HT2A	-11.27	-17.43	-23.54	0.01	-13.06
56631	5-HT2B	-2.96	-7.92	-4.87	2.23	-3.38
56631	5-HT2C	-11.5	-27.44	-9.47	-0.96	-12.34
56631	5-HT3	-9.28	-1.3	-11.94	8.19	-3.58
56631	5-HT4	-10.77	-6.91	1.38	13.05	-0.81
56631	5-HT5A	-7.87	8.97	-20.57	-10.35	-7.46
56631	5-HT6	31.02	18.26	9.06	14.17	18.13
56631	5-HT7A	-22.1	-17.1	-7.44	8.08	-9.64
56631	A1	61.95	38.86	23.72	4.24	32.19
56631	A2A	-13.16	-16.68	-13.54	0.05	-10.83
56631	Alpha1A	-11.72	-9.83	-15.69	-4.6	-10.46
56631	Alpha1B	3.43	-7.17	24.62	23.21	11.02
56631	Alpha1D	-19.79	-23.39	5.56	13.25	-6.09
56631	Alpha2A	-10.86	-3.51	-14.45	0.24	-7.15
56631	Alpha2B	-6.41	9.42	-5.26	-1.46	-0.93
56631	Alpha2C	6.82	10.58	-9.43	22.1	7.52
56631	Beta1	-23.81	-22.03	-13.14	-6.03	-16.25
56631	Beta2	-5.21	-1.72	-2.84	9.55	-0.05
56631	Beta3	-7.14	-10.36	-21.85	12.64	-6.68
56631	BZP Rat Brain Site	82.68	82.21	83.16	86.24	83.57
56631	D1	-43.73	-33.49	-46.55	-42.1	-41.47
56631	D2	-12.82	2.43	6.58	17.67	3.47
56631	D3	0.97	-2.67	6.8	-0.85	1.06
56631	D4	-0.66	1.13	-0.75	-1.23	-0.38
56631	D5	-18.48	-28.59	4.86	-2.01	-11.06
56631	DAT	7.36	16.36	12.86	25.36	15.49
56631	DOR	10.34	10.14	16.85	-12.63	6.18
56631	GABAA	12.16	4.46	0.6	20.35	9.39
56631	H1	-16.58	-14.78	-8.28	10.11	-7.38
56631	H2	-2.83	-19.82	-20.42	-5.41	-12.12
56631	H3	-7.55	-22.35	-7.55	15.27	-5.55
56631	H4	-21.26	-6.08	-18.17	-5.05	-12.64
56631	KOR	2.48	-9.75	-7.45	-0.42	-3.79
56631	M1	-20.43	-21.25	-21.34	-17.07	-20.02
56631	M2	3.71	-1.11	14.29	-4.76	3.03
56631	M3	-23.31	-13.44	-3.56	-11.52	-12.96
56631	M4	21.25	-7.36	-9.61	8.51	3.2

56631	M5	-24.72	-21.53	-9.24	8.11	-11.85
56631	MOR	-3.34	-10.92	11.3	21.14	4.55
56631	NET	14.3	-4.77	5.46	25.9	10.22
56631	PBR	10.83	-2.5	0.73	8.68	4.44
56631	SERT	-4.98	1.45	3.41	16.27	4.04
56631	Sigma 1	30.33	35.08	25.07	20.67	27.79
56631	Sigma 2	-10.09	-15.53	-25.68	0.78	-12.63
56631	AMPA	-7.48	-22.25	-16	3.5	-10.56
56631	Kainate (Rat Brain)	15.49	5.02	39.04	36.42	23.99
56631	NMDA	24.04	33.26	9.47	2.43	17.3
56631	NR2B	-8.36	-5.05	-11.84	-15.45	-10.18
56631	HCA2	-46.12	10.47	49.7	69.32	20.84
56631	Ca channel human	27.64	9.85	-22.23	1.48	4.19
56631	HERG	-1.42	4.11	1.92	-0.8	0.95
56631	M3D	0.48	-7.93	8.88	24.93	6.59
56631	M4 D	54.79	54.79	57.81	59.82	56.8
56631	NTS1	15.15	-6.85	-3.25	-3.93	0.28
56631	Alpha2Beta2	-3.03	4.86	-7.11	-3.71	-2.25
56631	Alpha2Beta4	1.78	11.9	14.35	-1.73	6.58
56631	Alpha3Beta2	6.42	-5.1	9.38	3.57	3.57
56631	Alpha3Beta4	-2.05	2.91	-2.5	-0.34	-0.5
56631	Alpha4Beta2	-2.89	0.86	-1.3	-0.03	-0.84
56631	Alpha4Beta2 (Rat Brain)	5.06	-4.74	7.6	2	2.48
56631	Alpha4Beta4	7.53	8.78	-1.68	12.76	6.85
56631	Alpha7	2.03	-3.16	-6.92	-5.72	-3.44
56631	NOP	-7.16	-31.54	-7.98	-11.27	-14.49
56631	OT	17.8	13.11	26.84	36.89	23.66
56631	V1A	-35.64	8.59	19.59	21.84	3.6
56631	V1B	17.29	18.21	29.65	31.88	24.26
56631	V2	-4.36	20.24	20.79	24.83	15.38
56631	DAT	15.67	22.61	34.78	36.09	27.29

Table S9. PDSP raw data for compound **23**

56632	5-HT1A	0.78	7.61	7.71	-7.17	2.23
56632	5-HT1B	0.28	-6.39	0.16	-9.33	-3.82
56632	5-HT1D	-21.45	-12.05	-15.34	-11.72	-15.14
56632	5-HT1E	21.89	24.61	20.87	9.96	19.33
56632	5-HT2A	-1.5	16.28	2.8	-19.97	-0.6
56632	5-HT2B	-1.38	-9.72	-1.72	-0.48	-3.33
56632	5-HT2C	-12.71	-23.65	-1.91	-1.91	-10.05
56632	5-HT3	-17	-4.93	-1.65	-5.02	-7.15

56632	5-HT4	-0.47	-0.23	-2.81	-0.71	-1.06
56632	5-HT5A	-16.7	7.04	2.35	-3.73	-2.76
56632	5-HT6	16.21	6.77	19.28	30	18.07
56632	5-HT7A	-23.66	-17.96	-11.93	-0.37	-13.48
56632	A1	79.26	-37.6	-13.8	13.62	10.37
56632	A2A	-18.49	-2.9	-19.72	2.14	-9.74
56632	Alpha1A	7.74	2.72	-10.46	-21.13	-5.28
56632	Alpha1B	16.95	19.07	29.77	23.01	22.2
56632	Alpha1D	-13.41	-31.24	11.29	7.2	-6.54
56632	Alpha2A	3.18	12.16	7.76	0.08	5.8
56632	Alpha2B	-4.6	20.3	0.19	2.66	4.64
56632	Alpha2C	-10.74	5.02	6.9	-1.67	-0.12
56632	Beta1	-15.51	-19.96	-17.59	9.96	-10.78
56632	Beta2	-10.73	-6.9	1.55	-7.46	-5.89
56632	Beta3	-4.44	-33.35	5.54	34.78	0.63
56632	BZP Rat Brain Site	50.65	63.7	48.04	49.47	52.97
56632	D1	-57.83	-27.4	-24.58	-38.98	-37.2
56632	D2	-14.46	-8.16	8.35	-4.88	-4.79
56632	D3	17.48	6.31	6.19	-0.73	7.31
56632	D4	-1.56	10.85	2.44	3.42	3.79
56632	D5	-25.48	-16.02	-3.18	-19.78	-16.12
56632	DAT	0.86	-66.97	0.69	16.02	-12.35
56632	DOR	24.17	21.73	11.97	1.8	14.92
56632	GABAA	20.35	16.98	25.17	30.46	23.24
56632	H1	-12.15	0.43	-9.52	11.08	-2.54
56632	H2	-1.31	-67.52	-1.66	-22.54	-23.26
56632	H3	0	0	-35.45	-15.58	-12.76
56632	H4	-33.87	0	-24.86	-8.39	-16.78
56632	KOR	20.83	35.97	29.24	21.44	26.87
56632	M1	-19.89	-2.42	-7.33	-18.25	-11.97
56632	M2	-17.08	4.48	-7.36	-4.67	-6.16
56632	M3	9.82	8.55	-5.47	-12.48	0.11
56632	M4	12.92	-25.68	28.5	-4.03	2.93
56632	M5	-24.5	3.39	-17.47	-15.61	-13.55
56632	MOR	1.33	4.23	22.4	-7.01	5.24
56632	NET	-5.46	8.63	-12.64	19.96	2.62
56632	PBR	45.84	-20.82	47.19	67.26	34.87
56632	SERT	-21.49	-7.83	7.52	-6.58	-7.1
56632	Sigma 1	47.97	58.99	40.67	25.07	43.18
56632	Sigma 2	9.17	7.49	9.89	8.85	8.85
56632	AMPA	-3.69	-63.15	-2.18	0.09	-17.23

56632	Kainate (Rat Brain)	7.63	36.86	53	50.82	37.08
56632	NMDA	59.73	98.33	94.2	104.4	89.17
56632	NR2B	-1.4	-18.79	-22.97	-47.59	-22.69
56632	HCA2	-20.47	2.92	13.49	64.79	15.18
56632	Ca channel human	10.2	6.36	-4.8	47.86	14.91
56632	HERG	0.25	-9.25	-5.18	-2.78	-4.24
56632	M3D	2.01	-2.58	23.4	13.47	9.08
56632	M4 D	38.66	62.85	44.71	55.79	50.5
56632	NTS1	-8.87	-5.72	-11.56	2.58	-5.89
56632	Alpha2Beta2	-3.84	-3.71	-10.1	2.96	-3.67
56632	Alpha2Beta4	-2.9	3.38	8.7	8.81	4.5
56632	Alpha3Beta2	12.43	2.24	8.56	5.3	7.13
56632	Alpha3Beta4	1.47	-1.06	1.65	5.89	1.99
56632	Alpha4Beta2	-7.97	-1.49	-0.98	-6.64	-4.27
56632	Alpha4Beta2 (Rat Brain)	10.15	-0.41	-2.07	3.15	2.71
56632	Alpha4Beta4	2.55	11.27	4.3	-0.44	4.42
56632	Alpha7	-7.82	-12.11	4.59	-5.79	-5.28
56632	OT	48.95	36.89	51.3	52.3	47.36
56632	V1A	60.01	5.22	5.22	31.04	25.37
56632	V1B	-88.55	22.02	21.36	28.46	-4.18
56632	V2	-23.64	10.33	15.65	38.6	10.24
56632	DAT	18.94	0.61	29.28	22.48	17.83

Table S10. PDSP raw data for compound **29**

56633	5-HT1A	-5.3	-6.89	1.16	0.22	-2.7	
56633	5-HT1B	-8.45	-9.33	-5.77	7.58	-3.99	
56633	5-HT1D	-24.86	-10.18	-6.22	-11.06	-13.08	
56633	5-HT1E	17.12	15.42	18.14	26.32	19.25	
56633	5-HT2A	1.23	4.56	-8.2	-13.13	-3.89	
56633	5-HT2B	3.01	102.86	0.87	13.16	29.98	
56633	5-HT2C	-30.41	-12.71	-2.18	2.41	-10.72	
56633	5-HT3	-5.38	-7.42	-2.89	-0.59	-4.07	
56633	5-HT4	-1.2	-6.59	-16.81	0.17	-6.11	
56633	5-HT5A	-8.7	-4	-16.43	14.22	-3.73	
56633	5-HT6	0.38	18.77	21.83	18.77	14.94	
56633	5-HT7A	-3.13	-9.68	-1.75	5.67	-2.22	
56633	A1	10.01	0	-54.19	18.67	-6.38	
56633	A2A	-2.8	-1.76	-33.41	-2.99	-10.24	
56633	Alpha1A	1.26	12.34	12.55	-6.28	4.97	
56633	Alpha1B	25.94	22.3	15.14	15.84	19.81	

56633	Alpha1D	-17.34	-25.68	-1.96	-2.62	-11.9	
56633	Alpha2A	-1.71	18.69	7.76	7.1	7.96	
56633	Alpha2B	-2.45	11.56	9.58	10.24	7.23	
56633	Alpha2C	8.62	22.83	38.02	10.09	19.89	
56633	Beta1	4.04	-11.66	13.51	28.92	8.7	
56633	Beta2	-2.96	-11.74	-5.55	-8.02	-7.07	
56633	Beta3	-0.38	8.75	10.1	20.41	9.72	
56633	BZP Rat Brain Site	86.48	88.14	83.87	91.46	87.49	Secondary
56633	D1	-18.94	-12.56	-44.92	-49.67	-31.52	
56633	D2	3.94	-3.12	-9.67	2.55	-1.58	
56633	D3	15.9	12.74	14.2	8.98	12.96	
56633	D4	-7.36	-1.48	5.95	8.32	1.36	
56633	D5	-24.06	-20.42	-9.66	-14.72	-17.22	
56633	DAT	-22.98	-16.14	-18.39	-1.23	-14.69	
56633	DOR	42.87	35.55	42.47	37.18	39.52	
56633	GABAA	1.08	35.76	26.61	39.61	25.77	
56633	H1	-29.99	7.48	-3.99	18.55	-1.99	
56633	H2	-2.72	-17.31	35.4	-16.5	-0.28	
56633	H3	-31.64	-34.18	-49.39	-2.91	-29.53	
56633	H4	-36.44	-28.72	-30.27	9.36	-21.52	
56633	KOR	32.76	82.45	83.52	77.71	69.11	Secondary
56633	M1	12.14	8.41	23.33	-24.07	4.95	
56633	M2	7.17	4.48	6.88	-6.4	3.03	
56633	M3	-26.82	-10.57	-1.33	3.13	-8.9	
56633	M4	10.27	14.68	-10.79	5.47	4.91	
56633	M5	-27.13	-20.11	-11.43	-11.32	-17.5	
56633	MOR	0	17.23	16.22	-4.23	7.31	
56633	NET	-1.73	-13.74	-18.44	23.41	-2.63	
56633	PBR	-8.02	-39	-12.74	16.08	-10.92	
56633	SERT	0.2	-2.66	8.41	18.32	6.07	
56633	Sigma 1	29.01	34.08	30.41	49.09	35.65	
56633	Sigma 2	7.49	19.48	3.5	24.28	13.69	
56633	AMPA	30.2	-31.91	-36.07	24.52	-3.32	
56633	Kainate (Rat Brain)	11.12	12	-5.02	49.07	16.79	
56633	NMDA	75.51	113.14	110.71	92.75	98.03	Secondary
56633	NR2B	-2.35	-13.71	-28.19	-12.05	-14.08	
56633	HCA2	9.12	-72.85	1.48	-42.98	-26.31	
56633	Ca channel human	19.62	22.76	19.27	29.03	22.67	
56633	HERG	-10.71	-11.54	3.38	12.66	-1.55	
56633	M3D	5.83	-3.34	13.47	11.17	6.78	
56633	M4 D	39.67	54.79	44.71	45.72	46.22	

56633	NTS1	-23.46	-8.64	-30.19	-15.82	-19.53	
56633	Alpha2Beta2	-8.06	-0.03	1.73	-5.2	-2.89	
56633	Alpha2Beta4	-5.99	5.08	10.19	2.95	3.06	
56633	Alpha3Beta2	-4.59	7.54	-0.41	11.52	3.52	
56633	Alpha3Beta4	1.2	-3.32	1.92	0.29	0.02	
56633	Alpha4Beta2	-4.22	3.97	-2.51	-1.87	-1.16	
56633	Alpha4Beta2 (Rat Brain)	-0.54	-2.2	-0.67	3.53	0.03	
56633	Alpha4Beta4	-9.4	-2.18	-5.67	4.05	-3.3	
56633	Alpha7	-17	-5.56	-0.98	15.34	-2.05	
56633	OT	28.52	42.25	33.88	62.69	41.84	
56633	V1A	0.28	4.55	4.77	26.1	8.93	
56633	V1B	6.64	5.98	1.51	29.91	11.01	
56633	V2	38.42	-5.28	20.79	33.83	21.94	
56633	DAT	1.92	16.06	-4.37	8.07	5.42	

Table S11. PDSP raw data for compound **5**

56642	5-HT1A	8.74	2.47	-3.03	1.31	2.37	
56642	5-HT1B	13.83	12.05	9.84	11.31	11.76	
56642	5-HT1D	-1.66	3.3	-7.87	-7.42	-3.41	
56642	5-HT1E	16.1	32.11	35.86	52.21	34.07	
56642	5-HT2A	21.44	2.27	12.05	6.86	10.66	
56642	5-HT2B	35.81	22.31	29.23	18.79	26.54	
56642	5-HT2C	38.78	15.25	14.27	3.87	18.04	
56642	5-HT3	12.29	-0.31	-4.16	-11.38	-0.89	
56642	5-HT4	1.38	-23.93	-20.26	-14.99	-14.45	
56642	5-HT5A	46.15	35.54	93.24	39.42	53.59	Secondary
56642	5-HT6	41.13	19.62	23.36	14.24	24.59	
56642	5-HT7A	7.06	2.35	2.98	4.86	4.31	
56642	A1	56.18	22.99	-41.21	65.55	25.88	
56642	A2A	11.36	13.83	-17.54	38.64	11.57	
56642	Alpha1A	-6.8	-6.59	-14.25	9.75	-4.47	
56642	Alpha1B	1.47	-15.82	-16.86	-30.2	-15.35	
56642	Alpha1D	26.37	-6.33	-20.53	14.78	3.57	
56642	Alpha2A	7.42	9.22	-3.37	10.69	5.99	
56642	Alpha2B	80.74	63.52	65.82	65.67	68.94	Secondary
56642	Alpha2C	19.52	-3.24	14	21.19	12.87	
56642	Beta1	-9.91	2.36	-33.82	-21.86	-15.81	
56642	Beta2	-13.56	12.44	-5.91	3.55	-0.87	
56642	Beta3	9.64	-28.36	-37.77	-20.98	-19.37	

56642	BZP Rat Brain Site	95.86	95.86	93.96	93.48	94.79	Secondary
56642	D1	-51.89	-43.63	-30.59	-15.52	-35.41	
56642	D2	4.88	-4.35	1.39	-0.86	0.27	
56642	D3	-5.11	3.04	-5.69	-6.51	-3.57	
56642	D4	4.78	-6.61	-6.61	-9.37	-4.45	
56642	D5	-12.59	-6.47	11.53	12.4	1.22	
56642	DAT	37.78	26.82	18.85	35.36	29.7	
56642	DOR	54.46	34.33	37.38	45.72	42.97	
56642	GABAA	8.87	-17.08	-6.01	-1.81	-4.01	
56642	H1	0.15	-13.33	-16.3	-12.12	-10.4	
56642	H2	-9.72	-8.44	3.92	8.55	-1.42	
56642	H3	20.84	6.03	-8.78	5.65	5.94	
56642	H4	16.03	-11.76	-5.38	4.71	0.9	
56642	KOR	61.01	59.47	50.84	42.98	53.58	Secondary
56642	M1	0.34	7.2	-11.04	-2.91	-1.6	
56642	M2	28.38	10.04	13.46	9.85	15.43	
56642	M3	18.61	-2.82	-2.49	-1.17	3.03	
56642	M4	0.92	-10.25	-10.91	7.07	-3.29	
56642	M5	3.96	-3.82	-1.64	-1.53	-0.76	
56642	MOR	-10.03	-4.15	-20.12	-10.41	-11.18	
56642	NET	9.94	-10.65	-13.19	-10.09	-6	
56642	PBR	19.05	21.17	27.79	21.03	22.26	
56642	SERT	-2.15	-17.87	-7.73	-9.34	-9.27	
56642	Sigma 1	44.73	39.66	56.43	46.82	46.91	
56642	Sigma 2	47.92	46.97	36.43	43.19	43.63	
56642	AMPA	-11.64	19.98	-0.66	23.38	7.77	
56642	Kainate (Rat Brain)	16.38	7.84	0	-10.51	3.43	
56642	NMDA	93.9	37.55	22.02	19.21	43.17	
56642	NR2B	7.74	9.21	1.69	-3.48	3.79	
56642	HCA2	24.4	-45.07	-22.84	4.25	-9.82	
56642	Ca channel human	53.46	30.01	38.48	34.24	39.05	
56642	HERG	5.22	2.65	-4.41	-5.38	-0.48	
56642	M3D	5.04	0.2	16.33	-2.22	4.84	
56642	M4 D	71.93	47.35	-54.33	43.99	27.24	
56642	NTS1	-23.43	-24.56	-3.48	-15.27	-16.69	
56642	Alpha2Beta2	-11.87	-5.07	-0.31	-6.56	-5.95	
56642	Alpha2Beta4	-6.1	10.41	4.55	10.41	4.82	
56642	Alpha3Beta2	6.01	-3.46	1.63	4.38	2.14	
56642	Alpha3Beta4	-6.11	1.92	-1.24	2.37	-0.77	
56642	Alpha4Beta2	-1.43	-3.72	-2.7	1.24	-1.65	
56642	Alpha4Beta2 (Rat Brain)	-13.27	-5.12	-3.85	-0.54	-5.7	

56642	Alpha4Beta4	0.31	4.05	9.03	-5.42	1.99	
56642	Alpha7	-24.82	-24.97	-13.31	-10.23	-18.33	
56642	OT	-1.64	-4.01	2.02	8.48	1.21	
56642	V1A	49.64	0.75	9.93	1.89	15.55	
56642	V1B	11.53	-15.35	5.18	4.25	1.4	
56642	V2	18.31	16.07	45.23	-0.76	19.71	
56642	OT	16.36	8.6	-1.33	9.09	8.18	

Table S12. PDSP raw data for compound **21**

56628	5-HT1A	9.86	10.14	4.06	-4.55	4.88	
56628	5-HT1B	-0.78	-4.4	-1.03	4.65	-0.39	
56628	5-HT1D	-7.1	-6	-9.79	-13.2	-9.02	
56628	5-HT1E	9.62	2.47	21.55	-2.64	7.75	
56628	5-HT2A	11.98	-13.42	-12.54	-17.62	-7.9	
56628	5-HT2B	-2.17	-9.83	-12.31	4.03	-5.07	
56628	5-HT2C	-8.8	-18.12	-20.41	-9.47	-14.2	
56628	5-HT3	0.39	-1.56	3.76	8.81	2.85	
56628	5-HT4	-3.61	-5.46	10.39	14.66	4	
56628	5-HT5A	2.35	8.14	-10.63	11.18	2.76	
56628	5-HT6	-3.96	-7.53	-13.66	-7.79	-8.24	
56628	5-HT7A	-17.62	-0.54	-20.72	-10.37	-12.31	
56628	A1	-11.6	-35.11	-28.75	22.08	-13.35	
56628	A2A	-40.16	-8.13	-1	-4.7	-13.5	
56628	Alpha1A	-1.05	-16.74	-6.07	2.09	-5.44	
56628	Alpha1B	-10.7	-22.91	-11	17.96	-6.66	
56628	Alpha1D	-8.51	-19.96	-13.09	19.46	-5.53	
56628	Alpha2A	-5.96	6.12	-12	7.43	-1.1	
56628	Alpha2B	-12.68	2.66	-6.41	6.62	-2.45	
56628	Alpha2C	2.25	-4.94	-13.27	10.41	-1.39	
56628	Beta1	18.55	12.33	-0.41	21.81	13.07	
56628	Beta2	-4.2	-0.59	2.11	1.55	-0.28	
56628	Beta3	19.06	-4.61	8.58	19.74	10.69	
56628	BZP Rat Brain Site	91.46	96.68	95.49	96.2	94.96	Secondary
56628	D1	2.88	15.79	19.2	0	9.47	
56628	D2	-3.37	-5.01	-10.93	2.55	-4.19	
56628	D3	25.61	-2.43	-5.58	7.16	6.19	
56628	D4	6.69	-3.44	5.87	-0.66	2.12	
56628	D5	7.07	11.22	25.61	4.73	12.16	
56628	DAT	6.27	7.52	-11.23	4.36	1.73	
56628	DOR	25.87	36.05	27.41	23.76	28.27	
56628	GABAA	-11.92	20.83	21.79	20.35	12.76	

56628	H1	-21.14	-13.95	-7.87	4.17	-9.7	
56628	H2	16.15	-11.2	1.48	-14.7	-2.07	
56628	H3	7.24	16.96	1.74	-6.71	4.81	
56628	H4	8.33	-1.45	-14.31	-10.2	-4.41	
56628	KOR	77.56	72.82	65.94	61.96	69.57	Secondary
56628	M1	-3.69	-23.35	-7.33	1.22	-8.29	
56628	M2	6.88	-20.45	11.89	2.55	0.22	
56628	M3	-1.33	-43.71	-8.66	2.18	-12.88	
56628	M4	-19.41	-29.99	-10.3	-7.95	-16.91	
56628	M5	-12.97	-9.13	-1.44	-8.58	-8.03	
56628	MOR	-5.62	-4.35	-12.81	8.52	-3.57	
56628	NET	-16.92	-8.63	-0.9	16.64	-2.45	
56628	PBR	-5.87	17.3	4.23	7.06	5.68	
56628	SERT	-0.16	19.48	17.96	-0.33	9.24	
56628	Sigma 1	38.3	50.34	50.17	39.49	44.58	
56628	Sigma 2	-21.6	-20.88	-12.81	59.45	1.04	
56628	AMPA	-9.94	-77.92	-32.66	-7.29	-31.95	
56628	Kainate (Rat Brain)	8.07	-32.5	-5.02	11.12	-4.58	
56628	NMDA	53.17	88.86	94.93	71.62	77.15	Secondary
56628	NR2B	2.87	-27.14	-13.01	2	-8.82	
56628	HCA2	8.96	27.82	18.01	88.18	35.74	
56628	Ca channel human	18.57	-18.05	10.55	28.33	9.85	
56628	HERG	-23.54	-4.66	6.92	-1.63	-5.73	
56628	M3D	23.4	-7.16	-7.93	-6.4	0.48	
56628	M4 D	36.65	-4.66	-9.7	-17.76	1.13	
56628	NTS1	-29.52	-21.66	-29.07	-20.76	-25.25	
56628	Alpha2Beta2	12.48	-0.71	3.23	0.51	3.88	
56628	Alpha2Beta4	4.98	5.62	13.81	6.25	7.67	
56628	Alpha3Beta2	2.04	6.11	-4.28	8.97	3.21	
56628	Alpha3Beta4	12.56	7.24	10.4	15.09	11.32	
56628	Alpha4Beta2	3.4	5.24	8.93	1.94	4.88	
56628	Alpha4Beta2 (Rat Brain)	12.69	-9.07	-3.72	2.9	0.7	
56628	Alpha4Beta4	18.24	21.23	13.01	5.29	14.44	
56628	Alpha7	-3.38	-18.5	-10.08	-11.43	-10.85	
56628	NOP	6.27	10.65	-3.87	-13.46	-0.1	
56628	OT	9.09	-10.34	-12.69	11.43	-0.63	
56628	V1A	43.61	51.92	35.31	9.71	35.14	
56628	V1B	44.9	48.32	49.76	40.03	45.75	
56628	V2	53.48	50.54	31.26	36.58	42.97	
56628	DAT	30.73	7.28	1.92	26.27	16.55	

Synthesis of compound **34** (Compound **34** in this manuscript is compound **1** in the publised thesis).

Li,G. "Design and Synthesis of Achiral and Chiral Imidazodiazepine (IMDZ) GABA(A)R Subtype Selective Ligands for the Treatment of CNS Disorders, as well as Asthma" (2019). Theses and Dissertations. 2093. https://dc.uwm.edu/etd/2093.

Synthetic scheme can be found on page 29 of the thesis

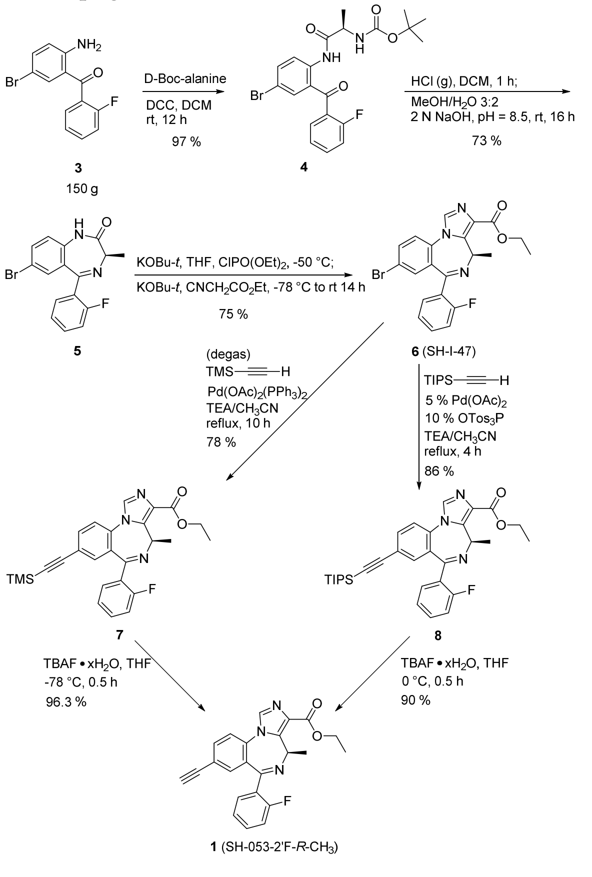

Experimental can be found on page 200 on the thesis

Ethyl (*R*)-8-ethynyl-6-(2-fluorophenyl)-4-methyl-4*H*-benzo[*f*]imidazo[1,5-*a*][1,4] diazepine-3-carboxylate (SH-053-2'F-*R*-CH_3, 1)

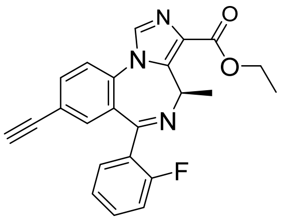

1 (SH-053-2'F-*R*-CH_3)

The intermediate **7** (44.4 g, 96.6 mmol) was dissolved in THF (400 mL) and cooled to -78 °C. This was treated with tetrabutylammonium fluoride hydrate (1 M solution in THF, 145 mmol), and this was followed by water (45 mL). The reaction mixture was stirred until the starting material was consumed as

indicated by TLC (silica gel), about 1 h. The reaction mixture was allowed to warm to rt, and water (200 mL) was slowly added. The solution was extracted with EtOAc and the organic extracts were combined, washed with brine, dried (Na_2SO_4), and the solvent was removed under reduced pressure. The residue which resulted was purified by a wash column (silica gel, EtOAc/hexanes 1:1) to afford pure ethyl ester **1** as a white powder (36 g, 96.3 %). The deprotection of the triisopropylsilyl analog **8** (27.2 g, 50.0 mmol) followed the same procedure as the deprotection of the trimethylsilyl analog **7** to produce the same product ethyl ester **1** (17.4 g, 89.8 %): **[α]** $^{26}{}_{D}$ = +20.9 (c 0.89, EtOAc); **^{1}H NMR** (300 MHz, $CDCl_3$) δ 7.93 (s, 1H), 7.69 (d, *J* = 8.1 Hz, 1H), 7.57 (t, *J* = 9.6 Hz, 2H), 7.48 – 7.36 (m, 2H), 7.24 (t, *J* = 7.5 Hz, 1H), 7.02 (t, *J* = 9.3 Hz, 1H), 6.69 (q, *J* = 7.1 Hz, 1H), 4.54 – 4.27 (m, 2H), 3.15 (s, 1H), 1.40 (t, *J* = 7.1 Hz, 3H), 1.27 (d, *J* = 7.2 Hz, 3H). **^{13}C NMR** (75 MHz, $CDCl_3$) δ 163.20 (s), 162.91 (s), 160.08 (d, $^1J_{C\text{-}F}$ = 252.0 Hz), 141.64 (s), 135.17 (s), 134.86 (s), 134.49 (s), 133.88 (s), 131.93 (d, $^3J_{C\text{-}F}$ = 8.2 Hz), 131.17 (s), 129.59 (s), 129.50 (s), 128.64 (d, $^3J_{C\text{-}F}$ = 12.6 Hz), 124.47 (d, $^4J_{C\text{-}F}$ = 3.3 Hz), 122.23 (s), 121.62 (s), 116.16 (d, $^2J_{C\text{-}F}$ = 21.5 Hz), 81.40 (s), 79.78 (s), 60.75 (s), 50.07 (s), 14.85 (s), 14.41 (s); **HRMS** (ESI/IT-TOF) m/z: $[M + H]^+$ Calcd for $C_{23}H_{19}FN_3O_2$ 388.1456; found 388.1439.

Synthesis of compound 24. (Compound **24** in this manuscript is compound **100** in the publised thesis).
Li,G. "Design and Synthesis of Achiral and Chiral Imidazodiazepine (IMDZ) GABA(A)R Subtype Selective Ligands for the Treatment of CNS Disorders, as well as Asthma" (2019). Theses and Dissertations. 2093. https://dc.uwm.edu/etd/2093.

Scheme can be found on page 338

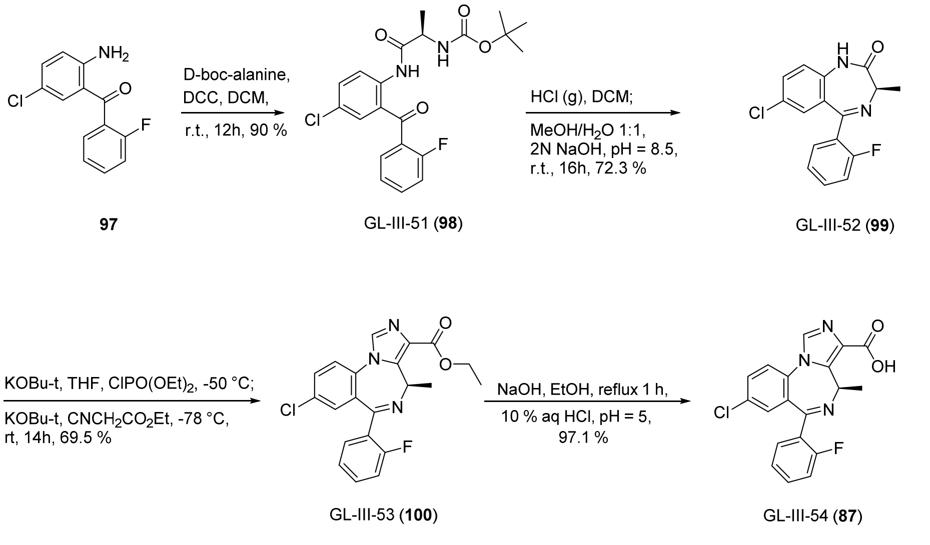

Experimental can be found on page 383

2.1.1.1. (*R*)-Ethyl-8-chloro-6-(2-fluorophenyl)-4-methyl-4*H*-benzo[*f*]imidazo[1,5-*a*][1,4]diazepine-3-carboxylate (GL-III-53, 100)

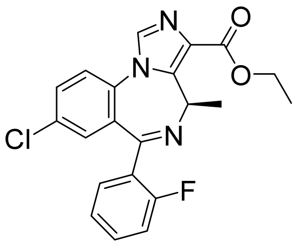

GL-III-53 (**100**)

The amide GL-III-52 **99** (25.0 g, 82.5 mmol) was dissolved in dry THF (300 mL), and cooled to -50 °C using a dry ice bath, after which potassium *t*-butoxide (12.0 g, 107.3 mmol) was added in one portion. The reaction mixture was stirred until it reached 0 °C and then stirred for 0.5 h at 0 °C. The mixture was then cooled to -50 °C, after which diethyl chlorophosphate (21.9 g, 125.0 mmol) was added dropwise with an addition funnel. The dry ice bath was removed to allow the temperature to rise to 0 °C, after which it was allowed to stir for 2 h with an ice-water bath. The solution was then cooled to -78 °C with a dry-ice bath and ethyl isocyanoacetate (14.1 g, 125.0 mmol) was added, and this was immediately followed by a second portion of potassium *t*-butoxide (12.0 g, 107.3 mmol). This solution was allowed to stir overnight during which period it was allowed to warm to rt. The reaction progress was completed after 14 h on analysis by TLC (silica gel, EtOAc/hexanes, 1:1). The reaction mixture was quenched by the addition of a cold saturated aq solution of $NaHCO_3$ (500 mL) and extracted with EtOAc. The organic layers were combined; washed with brine (2 x 200 mL), and dried (Na_2SO_4). The solvent was removed under reduced pressure to obtain a dark brown solid residue. The solid was washed with Et_2O/EtOAc (9:1) to remove most of the impurities and the solid was further recrystallized from EtOAc and hexane (1:3), and this was followed by washing the solid with cold Et_2O to afford the majority of the pure ethyl ester. Alternative recrystallization method: the residue was dissolved in MTBE and the mixture was heated to 50 °C, after which the solvent was removed under reduced pressure to 1/3 of its original volume to obtain the precipitates. The solid was filtered off and washed with cold Et_2O. The remaining filtrate was combined and purified by flash chromatography to obtain additional ethyl ester (silica gel, EtOAc/ hexanes 3/2) as

an off-white solid **100** (22.8 g, 69.5 % yield): **^{1}H NMR** (500 MHz, $CDCl_3$) δ 7.95 (s, 1H), 7.62 – 7.53 (m, 3H), 7.44 (td, *J* = 7.4, 1.4 Hz, 1H), 7.28 – 7.20 (m, 2H), 7.03 (t, *J* = 9.2 Hz, 1H), 6.69 (q, *J* = 7.2 Hz, 1H), 4.48 – 4.20 (m, 2H), 1.40 (t, *J* = 7.1 Hz, 3H), 1.27 (d, *J* = 7.3 Hz, 3H); **^{13}C NMR** (126 MHz, $CDCl_3$) δ 162.87, 161.07, 159.08, 141.49, 134.91, 133.29, 133.17, 132.19, 132.13, 132.00, 131.20, 130.82, 130.14, 129.46, 128.38, 128.28, 124.59, 124.56, 123.57, 116.31, 116.14, 60.81, 50.08, 14.86, 14.43; **HRMS** (ESI/IT-TOF) m/z: $[M + H]^+$ Calcd for $C_{21}H_{18}ClFN_3O_2$ 398.1066; found 398.1064.

Synthesis of compound **42**: compound **42** in this manuscript is the compound **153** in scheme

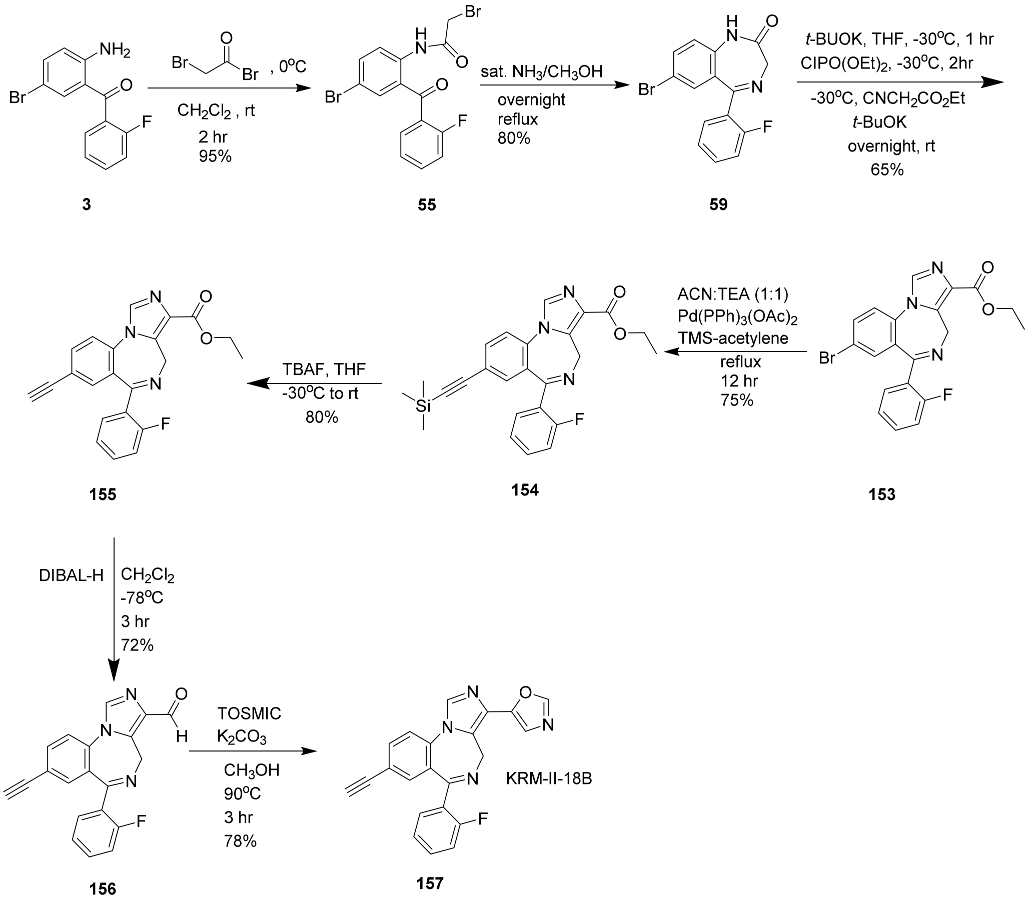

Ethyl 8-bromo-6-(2-fluorophenyl)-4H-benzo[f]imidazo[1,5-a][1,4]diazepine-3-carboxylate (153)

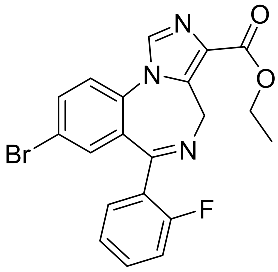

153

The imidazodiazepine intermediate, 7-bromo-5-(2-fluorophenyl)-1,3-dihydro-2H-benzo[e][1,4]diazepin-2-one (**59**, 25 g, 75.03 mmol) was dissolved in anhydrous tetrahydrofuran (150 mL) and cooled to −30 °C using a dry ice/IPA bath. Solid potassium t-butoxide (12.6 g, 112.55 mmol) was added in one portion once to the reaction mixture after which the mixture was stirred for 30 min at 0°C. Then diethyl chlorophosphate (16.26 mL, 112.55 mmol) was added dropwise to the reaction mixture over a 20 min period at −40 to −30 °C. Upon completion of the addition, the reaction mixture was allowed to stir until consumption of starting material as confirmed on silica gel TLC (ethyl acetate). At that point, ethyl isocyanoacetate (12.3 mL, 112.55 mmol) was then added dropwise to the reaction mixture at −50 to −40 °C for a period of 15 min followed by addition of potassium t-butoxide solution (12.6 g, 112.55 mmol) . The reaction mixture was then allowed to warm to rt and stirred for an additional 12 h at which point the reaction was deemed complete by analysis by TLC (silica gel and ethyl acetate). The reaction mixture was quenched with a saturated aq solution of sodium bicarbonate (100 mL) and diluted with ethyl acetate (200mL). The biphasic mixture, which resulted, was allowed to stand for 5 min and the layers were separated. The organic layer was separated and the aq layer was extracted with ethyl acetate (100 mL x 2). The combined organic layers were washed with 5 saturated solution of aq sodium chloride (2x 200 mL) and dried (Na2SO4). The solvents were removed under reduced pressure and the gummy residue was purified by silica gel column chromatography (EtOAc: Hexane = 3:7 to 6:4). The appropriate fractions were collected and the solvents were evaporated under reduced pressure. The residue was dried under vacuum to afford the product **153** as a light brown colored powder (20.8 g, 65%). R_f = 0.4 (EtOAc-hexanes,

1:1). ^{1}**H NMR** (500 MHz, CDCl3) δ 7.97 (s, 1H), 7.68 (dd, J = 67.8, 6.8 Hz, 2H), 7.50 (d, J = 8.2 Hz, 1H), 7.44 (d, J = 7.9 Hz, 2H), 7.21 (d, J = 4.5 Hz, 1H), 7.02 – 6.92 (m, 1H), 6.06 (d, J = 1.2 Hz, 1H), 4.37 (q, 2H), 4.07 (d, J = 0.4 Hz, 1H), 1.38 (t, J = 4.1 Hz, 3H). ^{13}**C NMR** (126 MHz, CDCl3) δ 164.98 (s), 162.77 (s), 160.16 (d, $^{1}J_{C\text{-}F}$ = 251.5 Hz), 138.31 (s), 135.16 (s), 134.36 (s), 133.27 (s), 133.13 (s), 132.49 (d, $^{4}J_{C\text{-}F}$ = 8.3 Hz), 131.24 (s), 130.52 (s), 129.38 (s), 127.37 (d, $^{3}J_{C\text{-}F}$ = 11.9 Hz), 124.55 (s), 124.28 (s), 121.23 (s), 116.24 (d, $^{2}J_{C\text{-}F}$ = 21.4 Hz), 60.80 (s), 44.87 (s), 14.44 (s). ^{19}**F NMR** (471 MHz, CDCl3) δ -112.10 (s). **HRMS** (LCMS-IT-TOF) Calc. for $C_{20}H_{15}N_{3}O_{2}FBr$ $(M + H)^{+}$ 428.0404, found 428.0405.
